# Nickel-catalyzed alkyl-arylation of 3,3,3-trifluoropropene

**DOI:** 10.1038/s42004-022-00659-7

**Published:** 2022-03-22

**Authors:** Chang Xu, Ming-Kuan Wang, Shu Zhang, Xingang Zhang

**Affiliations:** 1 Key Laboratory of Organofluorine Chemistry, Center for Excellence in Molecular Synthesis, Shanghai Institute of Organic Chemistry, University of Chinese Academy of Sciences, Chinese Academy of Sciences, 345 Lingling Road, Shanghai, 200032 China; 2 The Yangtze Delta Region Institute (Huzhou), University of Electronic Science and Technology of China, Huzhou, 313001 China

**Keywords:** Synthetic chemistry methodology, Synthetic chemistry methodology, Homogeneous catalysis

## Abstract

Owing to the versatile synthetic utility of its carbon-carbon double bond, low-cost industrial chemical 3,3,3-trifluoropropene (TFP) represents one of the most straightforward and cost-efficient precursors to prepare trifluoromethylated compounds. However, only limited methods for the efficient transformations of TFP have been reported so far. Here, we report a nickel-catalyzed dicarbofunctionalization of TFP. The reaction uses inexpensive NiCl_2_·6H_2_O as the catalyst and 4,4’-biMeO-bpy and PCy_2_Ph as the ligands, allowing the alkyl-arylation of TFP with a variety of tertiary alkyl iodides and arylzinc reagents in high efficiency. This nickel-catalyzed process overcomes the previous challenges by suppressing β-H and β-F eliminations from TFP, rendering this strategy effective for the transformations of TFP into medicinal interest trifluoromethylated compounds.

## Abstract (web summary)

3,3,3-Trifluoropropene is a cost-efficient precursor for the preparation of trifluoromethylated compounds, but limited methods for its efficient transformation have been reported. Here, nickel-catalyzed alkyl-arylation of 3,3,3-trifluoropropene with tertiary alkyl iodides and arylzinc reagents is achieved with high efficiency.

## Introduction

**Fig. 1:**
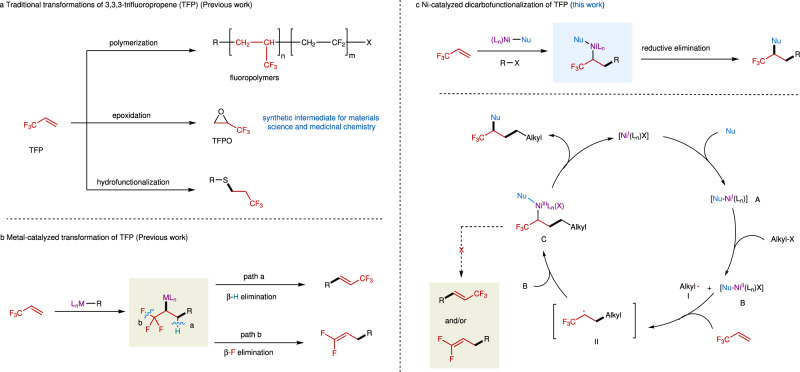
Transformations of 3,3,3-trifluoropropene. **a** Traditional transformations of TFP. **b** Previous work, metal-catalyzed transformation of TFP. **c** This work, Ni-catalyzed dicarbofunctionalization of TFP.

3,3,3-Trifluoropropene (TFP) is an low-cost industrial chemical used for the production of fluorinated polymers^1–5^ and refrigerants^6,7^. For practical applications, the functionalizations of the carbon-carbon double bond (C = C) in TFP are straightforward and cost-efficient strategies to prepare diversified trifluoromethylated compounds that are of great interests in pharmaceuticals and agrochemicals, due to the unique properties of the trifluoromethyl (CF_3_) group^8–11^. However, only limited methods for the transformations of TFP have been reported so far. Generally, these methods mainly focus either on the mono-functionalization of TFP, such as hydrofunctionalization^12–16^, or on the simple functionalization of TFP that produces limited types of products, such as polymerization^3,5,17,18^ and expoxidation^19,20^ (Fig. 1a). From the standpoint of synthetic efficiency, the dicarbofunctionalization of TFP would be a promising strategy to prepare trifluoromethylated compounds with multiple substitutes, enabling efficient construction of molecular complexity and structural diversity by forming two new C-C bonds in a one-pot reaction.

Recently, the nickel-catalyzed three-component dicarbofunctionalization of alkenes has received increasing attention^21–26^. However, the application of this process to the dicarbofunctionalization of TFP remains challenging, due to the undesired β-hydride (β-H)^27–29^ and β-fluoride (β-F)^29–31^ eliminations from the alkyl nickel intermediate (Fig. 1b). One general and effective strategy to suppress the β-H elimination in this process is using chelating group to form a stable nickellacycle intermediate^32–36^. While, the absence of chelating group on TFP renders it difficult to apply this strategy. It has also been demonstrated that the metal-mediated transformation of TFP is prone to defluorination driven by the formation of a strong metal-fluoride (M-F) bond^37–42^. To date, only a few examples of nickel-catalyzed transformations of TFP derivatives have been reported^29–31^, but they all undergo a defluorinative process that leads to the formation of *gem*-difluoroalkenes. As such, the development of a new catalytic system to overcome these challenges is imperative.

In our efforts to develop nickel-catalyzed dicarbofunctionalization of alkenes, we have established a nickel-catalyzed tandem radical process for dicarbofunctionalization of enamides through a chelation assisted strategy^32^. In this catalytic process, a highly active nickel(III) species bearing a chelated substrate was proposed as the key intermediate to suppress the β-hydride elimination. We envisioned the feasibility of nickel-catalyzed dicarbofunctionalization of TFP via a radical pathway in the absence of chelating group. Our design is based on the hypothesis that the nickel(III) species may enable the rate of reductive elimination step faster than those of undesired β-H and β-F eliminations. Following this hypothesis (Fig. 1c)^43–45^, the reaction may be induced by a nickel(I) complex [Nu-Ni^I^(L_n_)] (**A**) that can be derived from the transmetallation of a nucleophile with a nickel(I) species. Complex **A** reacts with an aliphatic electrophile to generate an alkyl radical (**I**) and nickel(II) [Nu-Ni^II^(L_n_)X] (**B**) via a single electron transfer (SET) pathway. Subsequently, **I** undergoes a radical addition with TFP to form a new alkyl radical (**II**), followed by combination with **B** to produce the key intermediate [Nu-Ni^III^(L_n_)(X)-alkyl] (**C**). Finally, reductive elimination of **C** delivers the dicarbofunctionalized product.

Herein, we report a nickel catalyzed dicarbofunctionalization of TFP. This nickel-catalyzed process allows alkyl-arylation of TFP with a variety of arylzinc reagents and tertiary alkyl iodides, providing a series of trifluoromethyl-substituted secondary alkanes with moderate to good yields. The advantage of this protocol is the rapid construction of complex trifluoromethylated compounds by using inexpensive industrial chemical TFP and low-cost nickel catalyst. The successful suppression of defluorinative side reaction in this approach paves a new way to practical application of TFP, and even more fluorinated olefins, in the synthesis of trifluoromethylated compounds of medicinal interest.

## Results and discussion

### Optimization of Ni-catalyzed alkyl-arylation of TFP

To test our hypothesis, tertiary alkyl iodide **2a** and arylzinc reagent **3a** were chosen as the model substrates for dicarbofunctionalization of TFP **1** (Table 1, see also Supplementary Tables 1–9). We found that the combination of NiCl_2_·DME (5 mol%) with bipyridine (bpy, **L1**) in DMA at 25 °C afforded dicarbofunctionalized product **4a** in 60% yield along with small amount of Heck-type side product **6a** (11% yield) (entry 1). Notably, no defluorinated side product **7a** was observed under these reaction conditions. Further examination of different ligands showed that electron-rich ligand 4,4’-diMeO-bpy (**L3**) could improve the yield to 65% (entry 3); while, other ligands, such as electron-deficient and bulky bpy-based ligands as well as 1,10-phenanthroline derivatives, led to lower yields with observation of **7a** in some cases (entries 2 and 4, see also Supplementary Table 1). Switching NiCl_2_·DME to monodentate phosphine ligand chelated nickel(II) salts benefited the reaction efficiency (entries 5–8). NiCl_2_(PPh_3_)_2_ provided **4a** in 68% yield (entry 5) and a higher yield (75%) was obtained with [*trans*-NiCl_2_(PCy_2_Ph)_2_] (**Ni-2**)^46^ as the catalyst (entry 7). Most importantly, **Ni-2** completely suppressed the formation of side products **6a** and **7a**. Control experiments showed that the absence of **L3** only led to *gem*-difluoroalkene **7a** and no product was obtained without nickel catalyst (entries 8–9). These results clearly demonstrate that nickel and ligand are essential in promoting the reaction, and the use of **L3** benefits the inhibition of β-F elimination. Encouraged by these results, we found that the combination of inexpensive NiCl_2_·6H_2_O (5 mol%) with PCy_2_Ph (**P1**) (5 mol%) could afford **4a** in 82% isolated yield (entry 12). Every possible permutation of a metal from Ni(NO_3_)_2_·6H_2_O and NiCl_2_·6H_2_O with a ligand from PCyPh_2_ and PCy_3_ was also applicable to the reaction, but relatively lower yields were obtained (entries 10, 11, 13 and 14). The absence of **P1** dramatically decreased the yield (52%) (entry 15). We also tested pyridine-based co-ligands, such as 4-dimethylaminopyridine (DMAP) and 4-trifluoromethylpyridine, but they all provided lower yields (see also Supplementary Table 8). Interestingly, the use of dimethoxyethane (DME) as the cosolvent (DMA/DME = 1:2, v/v) without phosphine ligand benefited the reaction efficiency as well, but a lower yield (73%) was obtained (entry 16, see also Supplementary Table 9). These results demonstrate that the additional monodentate phosphine ligand is critical to the reaction efficiency. This bidentate ligand plus monodentate ligand strategy ([2 + 1] ligand system)^45,47,48^ provides a good opportunity to fine tune the reaction conditions without need to prepare nickel complex, thus demonstrating the synthetic simplicity of this catalytic system.

**Table 1:** Representative Results for Optimization of Ni-Catalyzed Alkyl-Arylation of TFP^a^.


Entry	[Ni]	L	additive (*x*)	Yield [%]^b^		
				4a	6a	7a
1	NiCl_2_ ∙ DME	**L1**	--	60	11	ND
2	NiCl_2_ ∙ DME	**L2**	--	50	10	ND
3	NiCl_2_ ∙ DME	**L3**	--	65	9	ND
4	NiCl_2_ ∙ DME	**L4**	--	43	10	ND
5	NiCl_2_(PPh_3_)_2_	**L3**	--	68	5	9
6	**Ni-1**	**L3**	--	73	5	ND
7	**Ni-2**	**L3**	--	75	ND	ND
8	**Ni-2**	**--**	--	ND	ND	47
9	--	**L3**	--	ND	ND	ND
10	Ni(NO_3_)_2_ ∙ 6H_2_O	**L3**	PCy_2_Ph (10)	73	ND	ND
11	NiCl_2_ ∙ 6H_2_O	**L3**	PCy_2_Ph (10)	77	ND	ND
12	NiCl_2_ ∙ 6H_2_O	**L3**	PCy_2_Ph (5)	84 (82)	ND	ND
13	NiCl_2_ ∙ 6H_2_O	**L3**	PCyPh_2_ (5)	66	ND	7
14	NiCl_2_ ∙ 6H_2_O	**L3**	PCy_3_ (5)	72	ND	ND
15	NiCl_2_ ∙ 6H_2_O	**L3**	--	52	ND	ND
16^c^	NiCl_2_ ∙ 6H_2_O	**L3**	--	73	4	5

*ND* not detected.

^a^Reaction conditions (unless otherwise specified): **1** (2.0 equiv, 1 M in DMA, 0.8 mL), **2a** (0.4 mmol, 1.0 equiv), **3a** (1.5 equiv in THF), and DMA (2 mL).

^b^Determined by ^19^F NMR using benzotrifluoride as an internal standard; the number given in the parentheses is the isolated yield.

^c^DMA + DME (2 mL, DMA/DME = 1:2, v/v) were used.

### Ni-catalyzed alkyl-arylation of TFP

**Fig. 2:**
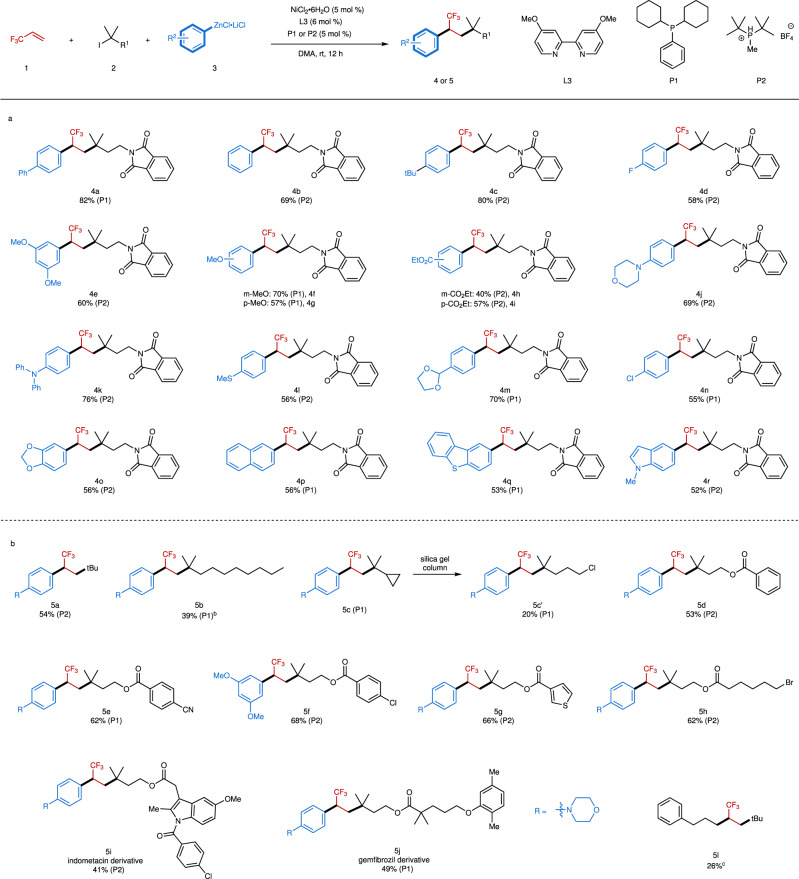
Scope of the Nickel-catalyzed alkyl-arylation of TFP. ^a^Reaction conditions (unless otherwise specified): **1** (0.8 mmol, 2.0 equiv), **2** (0.4 mmol, 1.0 equiv), **3** (1.5 equiv in THF), and DMA (2 mL). Isolated yields are given. The phosphine ligands used for the substrates were enclosed in the parentheses. ^b^Reaction was conducted at 0 °C. ^c^Reaction was conducted without **L3** and phosphine ligand, and 5 mol% NiCl_2_·DME was used. **a** Substrate scope of arylzinc reagents with **2a**. **b** Substrate scope of alkyl iodides.

With the optimized reaction conditions in hand, the substrate scope of the reaction was examined. We found that the arylzinc reagent bearing an electron-withdrawing group, such as 4-fluorophenylzinc, provided a low yield (41%). This limitation can be addressed by using (*t*-Bu_2_MeP)∙HBF_4_ (**P2**) as the alternative ligand, providing corresponding product **4d** in 58% isolated yield (Supplementary Table 10). **P2** was also applicable to the model reaction, with 75% yield obtained (Supplementary Table 7). Similarly, the use of DME as the cosolvent (DMA/DME = 1/3, v/v) without **P2** could also improve the yield (from 41% to 50%), but with lower activity than **P2** (Supplementary Table 11). Thereby, both phosphine ligands **P1** and **P2** were chosen as the co-ligands for the reaction and the representative results were illustrated in Fig. 2.

Generally, both electron-rich and electron-deficient arylzinc reagents were suitable substrates, providing the corresponding products **4** with high efficiency (Fig. 2a, Supplementary Fig. 2). Specifically, **P1** was more suitable for electron-neutral substrates, and **P2** was more suitable for the substrates bearing electron-withdrawing or electron-donating groups. However, this feature was not absolute, in some cases, **P1** provided higher yields for electron-rich and electron-deficient arylzinc reagents (**4f**, **4g**, **4m**, **4n**). The reaction exhibited good functional groups tolerance. Various important functional moieties, such as ester (**4h**, **4i**), morpholine (**4j**), thioether (**4l**), 1,3-dioxolane (**4m**), and even aryl chloride (**4n**), were all compatible with the reaction conditions. Heteroarylzinc reagents including dibenzothiophene (**4q**) and indole (**4r**) derived substrates were also applicable to the reaction, demonstrating the generality of this approach.

In addition to arylzinc reagents, a series of tertiary alkyl iodides were also tested (Fig. 2b, Supplementary Fig. 1). *tert*-Butyl iodide (**5a**) and the substrate bearing a long carbon-chain, such as *n*-octyl (**5b**), underwent the reaction smoothly. Even cyclopropyl-containing substrate could also produce corresponding product **5c**. Unfortunately, **5c** was unstable upon purification with silica gel chromatography, and a ring-opening product **5c’** was obtained with dichloromethane as the eluent. The aliphatic chain bearing benzoyl (**5d**), cyano (**5e**), aryl chloride (**5f**), thiophene (**5g**), and alkyl bromide (**5h**) moieties showed good tolerance to the reaction. Remarkably, the pharmaceutical derived substrates, such as indomethacin- and gemfibrozil-containing tertiary alkyl iodides were competent coupling partners, providing **5i** and **5j** in synthetically useful yields. The alkylzinc reagent was also tested, leading to **5l** in 26% yield. However, tertiary acyclic bromides and secondary alkyl halides, including benzylic iodides and bromides, were not applicable to the reaction.

**Fig. 3:**
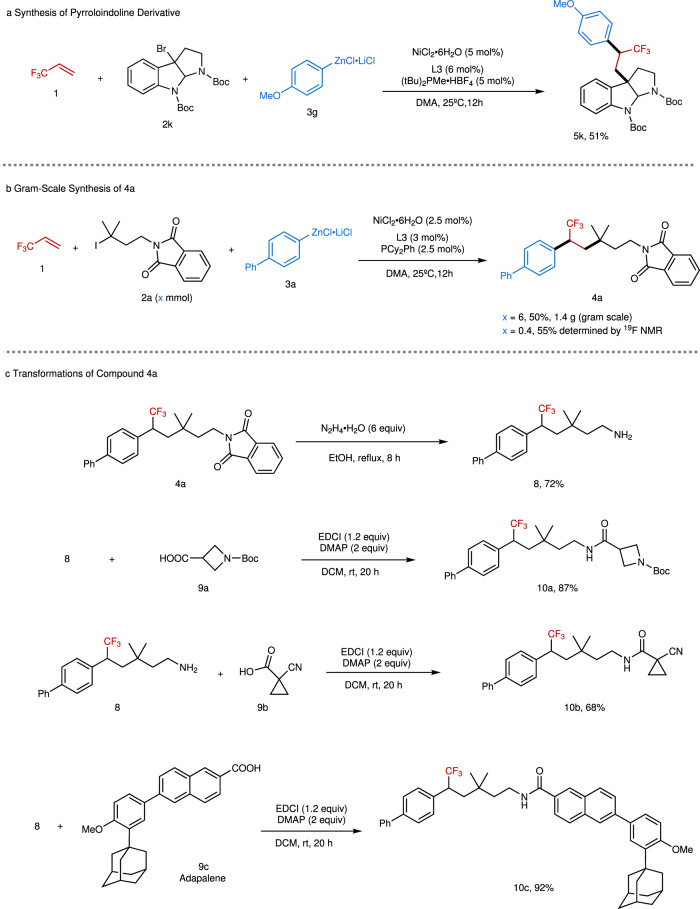
Synthetic applications. **a** Synthesis of pyrroloindoline derivative. **b** Gram-scale synthesis of **4a**. **c** Transformations of compound **4a**.

To demonstrate the synthetic utility of this approach further, pyrroloindoline derivative **5k** was prepared. As shown in Fig. 3a, treatment of TFP with tertiary benzyl bromide **2k** and arylzinc reagent **3g** afforded **5k** in 51% yield. Since pyrroloindoline derivatives show significant bioactivities as a cholinesterase inhibitor^17^ and CF_3_ has important applications in medicinal chemistry^3^, the present nickel-catalyzed process may offer good potential for discovery of new bioactive compounds. The reaction can also be scaled up, as demonstrated by the gram-scale synthesis of **4a** with 2.5 mol% catalyst loading, which provided a comparable yield (50% isolated yield) with the small-scale reaction (55% yield, determined by ^19^F NMR) (Fig. 3b). Although a lower yield was obtained, this approach trades off the overall yield loss from the multi-step synthesis of **4a**. Deprotection of **4a** provided free amine **8** in good yield, which can serve as a versatile building block for various transformations (Fig. 3c). For instance, condensation of **8** with a series of carboxylic acids, including azetidine- (**9a**) and cyclopropane-containing substrates (**9b**) and adapalene (**9c**) used for the treatment of acne vulgaris, provided the corresponding products **10a**–**10c** in high efficiency. Because of its synthetic simplicity that can rapidly construct complex trifluoromethylated compounds by forging two new C-C bonds in one-pot reaction, this approach provides a facile route for application of TFP in medicinal chemistry.

**Fig. 4:**
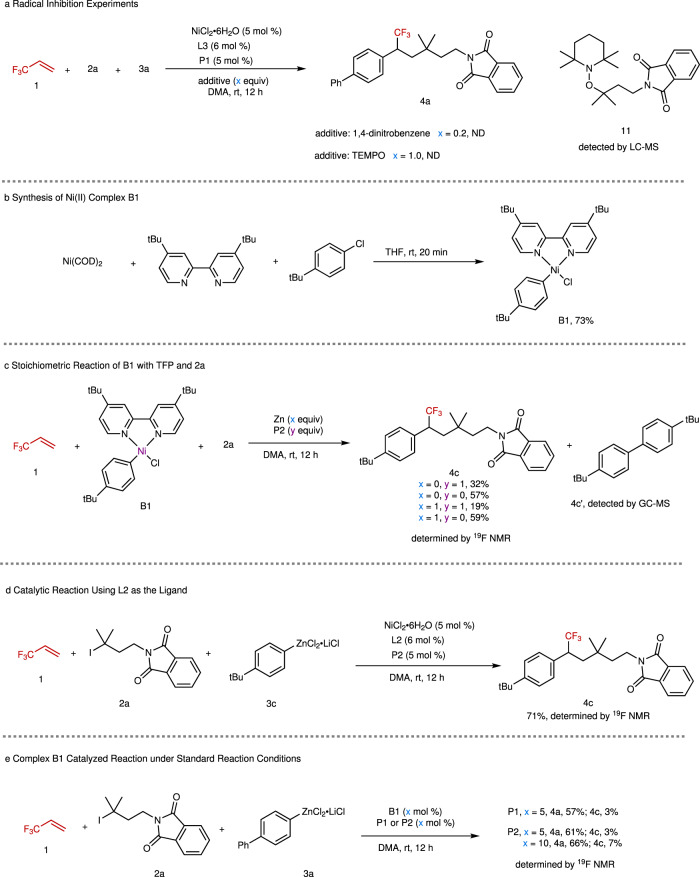
Mechanistic studies. **a** Radical inhibition experiments with 1,4-dinitrobenzene and TEMPO. **b** Synthesis of arylnickel(II) complex **B1**. **c** Stoichiometric reactions of **B1** with TFP and **2a**. **d** Catalytic reaction using **L2** as the ligand. **e** Standard reaction catalyzed by complex **B1**.

**Fig. 5:**
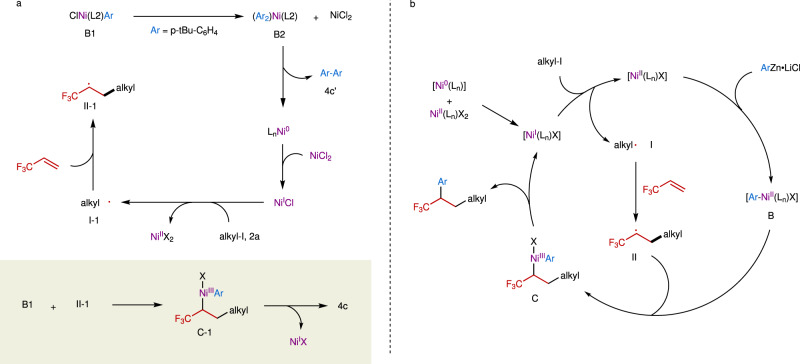
Proposed reaction mechanisms. **a** Proposed mechanism for the formation of **4c** from the reaction of **B1** with **1** and **2a**. **b** Proposed mechanism for the reaction.

To gain mechanistic insight into the reaction, several experiments were conducted (Fig. 4). Radical inhibition experiments with 1,4-dinitrobenzene and TEMPO as the radical inhibitors^49,50^ indicated that a radical was involved in the reaction (Fig. 4a). A radical trapping product **11** by TEMPO in the reaction, observed by LC-MS (Supplementary Fig. 3), further confirmed that an alkyl radical was involved in the reaction. We also prepared arylnickel(II) complex [*p*-*t*Bu-C_6_H_4_-Ni^II^(**L2**)Br] (**B1**) by reaction of Ni(COD)_2_ with 1-chloro-4-(1,1-dimethylethyl)benzene to identify the role of phosphine ligand and the active arylnickel species in the reaction (Fig. 4b)^51^. We used **L2** instead of **L3** as the supporting ligand because of difficulty in accessing [*p*-*t*Bu-C_6_H_4_-Ni^II^(**L3**)Br] (**B1’**). Given that nickel(II) can be reduced into nickel(I) by zinc^52,53^ several control experiments by using zinc to reduce **B1** were also conducted (Fig. 4c). We found that the absence of phosphine ligand with or without reductant zinc afforded higher yields of **4c** (57% without Zn; 59% with Zn) than those with **P2** (32% without Zn; 19% with Zn) (Fig. 4c). Particularly, using zinc without **P2** provided **4c** in a yield three times higher than that with **P2**. These results are in sharp contrast to the catalytic reactions. We surmised that the phosphine ligand is soft and more susceptible to leaving the hard nickel center, compared with the hard bpy-based ligands, which may benefit the formation of an active precatalyst or protect the active nickel species from decomposition^54^. Here the phosphine ligand is likely to play a similar coordination role as DME, even with higher activity. The exact role of the phosphine ligand still remains elusive at this stage. Comparable yields of **4c** were obtained in the absence of **P2** with or without zinc (59% with Zn; 57% without Zn), indicating that the arylnickel(II) species is likely involved in the reaction. The involvement of this nickel species in the catalytic cycle are also supported by several facts: the catalytic reaction using **L2** as the ligand provided **4c** efficiently (Fig. 4d), and complex **B1** could serve as a good catalyst for the reaction of **1** with **2a** and **3a** under standard reaction conditions, in which 3–7% yield of **4c** that originated from **B1** was observed (Fig. 4e). Since all the control experiments illustrated in Fig. 4c provided biaryl side product **4c’**, we surmised that a diarylnickel(II) complex **B2** and a NiCl_2_ were formed between two arylnickel(II) species, which underwent reductive elimination to produce the biaryl product and a Ni(0) (Fig. 5a)^55^. Comproportionation of Ni(0) with NiCl_2_ would generate a Ni^I^Cl species^56^. The resulting active nickel(I) species underwent SET reduction of tertiary alkyl iodide **2a** to afford alkyl radical **I-1**, which was subsequently trapped by TFP to produce a new alkyl radical **II-1**. Combination of arylnickle(II) complex **B1** with the newly formed alkyl radical **II-1** would generate a key intermediate aryl(alkyl)nickel(III) species **C1**. Finally, reductive elimination of **Cl** provided product **4c** and regenerate Ni(I)X.

On the basis of aforementioned results and previous reports^23,34^, a possible reaction mechanism was proposed (Fig. 5b). The reaction was initiated by the SET reduction of alkyl iodide with Ni(I)X to generate an alkyl radical (**I**) and Ni(II)X_2_ species, in which Ni(I)X was generated by the comproportionation of Ni(II)X_2_ with in-situ generated Ni(0)^56^. Subsequently, alkyl radical (**I**) was trapped by TFP to form a new alkyl radical (**II**). Meanwhile, transmetallation of arylzinc reagent with Ni(II)X_2_ provided arylnickel(II) complex **B**. Combination of **B** with **II** produced the key intermediate nickel(III) species **C**, which underwent reductive elimination to afford the dicarbofunctionalized product and regenerate Ni(I)X. However, the possible pathway illustrated in Fig. 1c still cannot be ruled out.

In conclusion, we have developed a nickel-catalyzed dicarbofunctionalization of industrial chemical TFP. The use of [2 + 1] ligand system overcomes the previous challenges by suppressing β-H and β-F eliminations of TFP, allowing alkyl-arylation of TFP with a variety of tertiary alkyl iodides and arylzinc reagents in high efficiency. The reaction exhibits good functional group tolerance, including pharmaceuticals, providing an efficient route for application of TFP in rapid synthesis of complex trifluoromethylated compounds that are of interests in medicinal chemistry. Most importantly, the successful suppression of β-F elimination side reaction paves a new way for diversified transformation of TFP, which may prompt the research on using industry relevant fluoroolefins as the starting materials for the synthesis of valuable fluorinated structures.

## Methods

### General procedure for the Ni-catalyzed alkylarylation of 3,3,3-trifluoropropene 1 with tertiary alkyl Iodides 2 and arylzinc reagents 3

To a 25 mL of Schlenck tube were added 4,4’-diMeO-2,2’-bpy (6 mol%), NiCl_2_ ∙ 6H_2_O (5 mol%) and the monodentate phosphine ligand (PCy_2_Ph or P^*t*^Bu_2_Me ∙ HBF_4_, 5 mol%). The tube was evacuated and backfilled with argon for 3 times, then tertiary alkyl iodide **2** (0.4 mmol, 1.0 equiv) and TFP solution (1 M in DMA, 0.8 mmol, 2.0 equiv) were added under Ar. The resulting mixture was stirred for 20 min at room temperature, and the corresponding arylzinc reagent **3** (0.6 mmol, 1.5 equiv) was added slowly within a period of 5 min, and the tube was sealed with a Teflon cap. After stirring for 12 h at room temperature, the reaction mixture was quenched with aqueous NH_4_Cl solution and diluted with EtOAc. The reaction mixture was filtered through a pad of Celite, and the filtrate was extracted with EtOAc and washed with brine. The organic layer was dried over Na_2_SO_4_, filtered and concentrated. The residue was purified with silica gel chromatography to give the corresponding products **4** or **5**. Isolated yields are based on the average of two runs under identical conditions.

## Supplementary information

Supplementary Information

## Data Availability

The authors declare that all the data supporting the findings of this study are available within the paper and its Supplementary Information Files. For characterization data for all new compounds see Supplementary Methods and Supplementary Note, for all NMR spectra see Supplementary Figs. 4–119.
