# Supplementary material for: Nickel-catalyzed alkyl-arylation of 3,3,3-trifluoropropene
Source: Commun Chem. 2022 Mar 22;5:41. doi: 10.1038/s42004-022-00659-7 (PMC9814099; doi:10.1038/s42004-022-00659-7)
Supplement: Supplementary file 1 — Supplementary Information [file 42004_2022_659_MOESM1_ESM.pdf]

Nickel-Catalyzed Alkyl-arylation of 3,3,3-Trifluoropropene

Chang Xu, Ming-Kuan Wang, Shu Zhang and Xingang Zhang*

*Corresponding author. Email: xgzhang@mail.sioc.ac.cn

Table of Contents

Supplementary Methods

1. Materials and Methods....S2

2. Preparation of Tertiary Alkyl Iodides and Bromides S3

3. General Procedure for the Preparation of Arylzinc Reagents from Aryl Bromides S6

4. Optimization of Ni-Catalyzed Akylarylation of 3,3,3-Trifluoropropene **1** with Tertiary Alkyl Iodide **2a** and Arylzinc Reagent **3a**....S8

5. General Procedure for the Ni-Catalyzed Alkylarylation of 3,3,3-Trifluoropropene **1** with Tertiary Alkyl Iodides **2** and Arylzinc Reagents **3** S16

6. Characterization Data for Compounds **4** and **5**....S17

7. Transformations of Compound **4a** S30

8. Mechanistic Studies S33

Supplementary Note

Supplementary Note 1: Characterization Spectra forTertiary Alkyl Iodides **2** S36

Supplementary Note 2: Characterization Spectra for Compounds **4** and **5** S43

Supplementary Note 3: Characterization Spectra for Compounds **8-10**....S88

Supplementary References....S94

Supplementary Methods

1. Materials and Methods

General Information: ^{1}H NMR and ^{13}C NMR spectra were recorded on a Bruker AM400, AM500 or Agilent 400 spectrometer and are calibrated using residual undeuterated solvent ($CHCl_3$ at 7.26 ppm ^{1}H NMR, 77.00 ppm ^{13}C NMR; CD_3OD at 3.34 ppm ^{1}H NMR, 49.86 ppm ^{13}C NMR). ^{19}F NMR was recorded on a Bruker AM400 spectrometer ($CFCl_3$ as an external standard and low field is positive). Chemical shifts (δ) are reported in ppm, and coupling constants (*J*) are in Hertz (Hz). The following abbreviations were used to explain the multiplicities: s = singlet, d = doublet, t = triplet, q = quartet, m = multiplet, br = broad. NMR yield was determined by ^{19}F NMR using fluorobenzene as an internal standard before working up the reaction.

Materials: All reagents were used as received from commercial sources and used without further purification. Superdry solvents, DMF, DMA and MeCN were purchased form commercial sources. 1,4-Dioxane, THF, DCM and tolunene were taken from solvent purification system (PureSolv MD5, inert technology) and stored in 500 mL storage flasks with high vacuum valve. *trans*-$NiCl_2(PCy_2Ph)_2$ and $NiCl_2{\cdot}6H_2O$ were purchased from Strem chemicals and used as received. 4,4'-diMeO-2,2'-bpy was purchased from Aladdin Chemicals, PCy_2Ph (**P1**) and $P^tBu_2Me{\cdot}HBF_4$ (**P2**) were purchased from Adamas-beta Chemicals, and all used as received. 3,3,3-Trifluoropropene (TFP) **1** was purchased from Shangfluoro chemicals and used as received.

Preparation of 3,3,3-Trifluoropropene (TFP) Stock Solution

Anhydrous DMA (300 mL) was added to a 500 mL Schlenk flask under argon atmosphere (Ar). TFP gas was then slowly bubbled through DMA with stirring until the total volume of the solution reaches the maximum (generally 2 hours). The concentration of the TFP stock solution was determined by ^{19}F NMR using benzotrifluoride as an internal standard (generally 0.9 ~ 1.0 mol/L). This solution could be stored at room temperature for one month without obvious loss of TFP.

2. Preparation of Tertiary Alkyl Iodides and Bromides

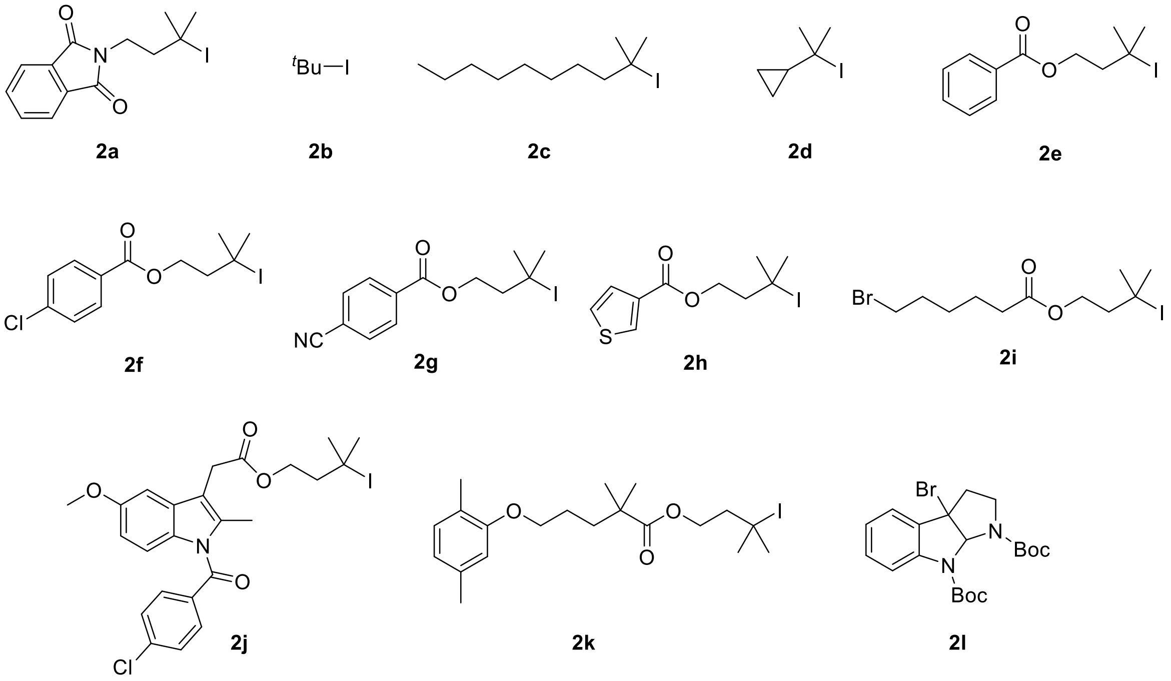

Supplementary Figure 1 Structure of Tertiary Alkyl Iodides and Bromides

Tertiary alkyl halide **2b** was purchased from commercial source. Compounds **2c**[1], **2e**[2] **2d**[3] and **2l**[4] are known compounds and were prepared according to the literatures.

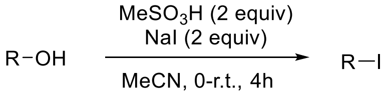

The general procedure for the preparation of tertiary alkyl halides is according to the literature.[5] To a 500 mL round-bottom flask were added tertiary alcohol (1 equiv) and MeCN (0.2 M for tertiary alcohol) with stirring. The clear solution was cooled to 0 °C with an ice-water bath, and NaI (2 equiv) was added in one portion. $MeSO_3H$ (2.0 equiv) was added dropwise to the solution under vigorous stirring with precipitate formed. The reaction was stirred for 4 h at room temperature. After the consumption of the alcohol monitored by TLC, the reaction was concentrated and diluted with Et_2O, washed by water, saturated $NaHCO_3$, saturated $Na_2S_2O_3$, and brine, dried over Na_2SO_4, and concentrated on a rotary evaporator. The residue was purified by flash column chromatography to give the corresponding tertiary alkyl iodide. The iodides were unstable and would slowly become deteriorated during column chromatography. The product must be stored at -20 °C away from light to prevent its decomposition.

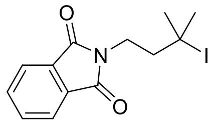

2-(3-Iodo-3-methylbutyl)isoindoline-1,3-dione (2a). The product (13.7 mmol scale, 3.7 g, 79% yield) as a white solid (m.p. 88.4 – 89.7 °C) was purified with silica gel chromatography (Hexane/Ethyl Acetate = 5/1). ^{1}H NMR (400 MHz, $CDCl_3$) δ 7.87 – 7.81 (m, 2H), 7.74 – 7.67 (m, 2H), 3.94 – 3.87 (m, 2H), 2.03 – 1.98 (m, 8H). ^{13}C NMR (101 MHz, $CDCl_3$) δ 168.1, 133.9, 132.1, 123.2, 47.5, 45.2, 38.0, 37.7. MS (DART): m/z (%) 344.0 ($[M+H]^+$), 361.0 ($[M+NH_4]^+$, 100). HRMS (DART): Calculated for $C_{13}H_{15}INO_2$ ($[M+H]^+$): 344.0142; Found: 344.0139.

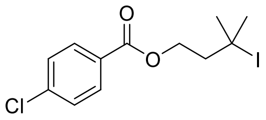

3-Iodo-3-methylbutyl 4-chlorobenzoate (2f). The product (34 mmol scale, 8.0 g, 67% yield) as a yellow oil was purified with silica gel chromatography (Hexane/Ethyl Acetate = 15/1). ^{1}H NMR (400 MHz, $CDCl_3$) δ 7.94 (d, J = 8.5 Hz, 2H), 7.38 (d, J = 8.5 Hz, 2H), 4.53 (t, J = 6.8 Hz, 2H), 2.14 (t, J = 6.8 Hz, 2H), 1.99 (s, 6H). ^{13}C NMR (101 MHz, $CDCl_3$) δ 165.5, 139.4, 130.9, 128.7, 128.5, 65.2, 48.0, 45.76, 38.5. MS (DART): m/z (%) 353.0 ($[M+H]^+$), 370.0 ($[M+NH_4]^+$, 100). HRMS (DART): Calculated for $C_{12}H_{15}ClIO_2$ ($[M+H]^+$): 352.9800; Found: 352.9799.

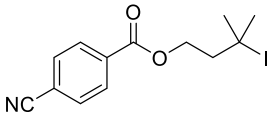

3-Iodo-3-methylbutyl 4-cyanobenzoate (2g). The product (46 mmol scale, 9.5 g, 60% yield) as a yellow solid (m.p. 60.3 – 61.5 °C) was purified with silica gel chromatography (Hexane/Ethyl Acetate = 5/1). ^{1}H NMR (400 MHz, $CDCl_3$) δ 8.11 (d, J = 8.2 Hz, 2H), 7.73 (d, J = 8.2 Hz, 2H), 4.57 (t, J = 6.8 Hz, 2H), 2.15 (t, J = 6.8 Hz, 2H), 2.00 (s, 6H). ^{13}C NMR (126 MHz, $CDCl_3$) δ 164.9, 134.0, 132.4, 130.2, 118.0, 116.6, 66.0, 48.0, 45.5, 38.6. MS (FI): m/z (%) 216 ($[M-HI]^+$), 130, 69 (100). HRMS (FI): Calculated for $C_{13}H_{13}NO_2$ ($[M-HI]^+$): 215.0941; Found: 215.0940.

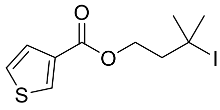

3-Iodo-3-methylbutyl thiophene-3-carboxylate (2h). The product (31.7 mmol scale, 5.0 g, 49% yield) as a yellow oil was purified with silica gel chromatography (Hexane/Ethyl Acetate = 10/1). ^{1}H NMR (400 MHz, $CDCl_3$) δ 8.08 (d, J = 3.0 Hz, 1H), 7.50 (d, J = 4.8 Hz, 1H), 7.29 (dd, J = 5.1 Hz, 3.1 Hz, 1H), 4.49 (t, J = 6.8 Hz, 2H), 2.13 (t, J = 6.8 Hz, 2H), 2.00 (s, 6H). ^{13}C NMR (101 MHz, $CDCl_3$) δ 162.4, 133.4, 132.7, 127.2, 126.0, 64.6, 48.0,

46.1, 38.4. MS (DART): m/z (%) 325.0 ([M+H]$^+$), 342.0 ([M+NH$_4$]$^+$, 100). HRMS (DART): Calculated for $C_{10}H_{14}IO_2S$ ([M+H]$^+$): 324.9754; Found: 324.9750.

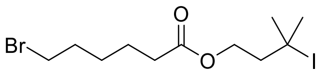

3-Iodo-3-methylbutyl 6-bromohexanoate (2i). The product (45 mmol scale, 10.0 g, 57% yield) as a yellow oil was purified with silica gel chromatography (Hexane/Ethyl Acetate = 20/1). ^{1}H NMR (400 MHz, $CDCl_3$) δ 4.29 (t, *J* = 7.0 Hz, 2H), 3.40 (t, *J* = 6.7 Hz, 2H), 2.31 (t, *J* = 7.4 Hz, 2H), 2.00 (t, *J* = 7.0 Hz, 2H), 1.96 (s, 6H), 1.90 – 1.82 (m, 2H), 1.69 – 1.59 (m, 2H), 1.51 – 1.41 (m, 2H). ^{13}C NMR (101 MHz, $CDCl_3$) δ 173.2, 64.4, 47.9, 46.0, 38.4, 34.0, 33.4, 32.3, 27.5, 23.9. MS (DART): m/z (%) 408.0 ([M+NH$_4$]$^+$, 100). HRMS (DART): Calculated for $C_{11}H_{21}BrIO_2$ ([M+H]$^+$): 390.9764; Found: 390.9763.

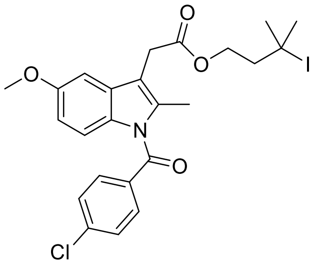

3-Iodo-3-methylbutyl 2-(1-(4-chlorobenzoyl)-5-methoxy-2-methyl-1H-indol-3-yl)acetate (2j). The product (20 mmol scale, 4.0 g, 37% yield) as a yellow viscous oil was purified with silica gel chromatography (Hexane/Ethyl Acetate = 5/1). ^{1}H NMR (400 MHz, $CDCl_3$) δ 7.65 (d, *J* = 8.5 Hz, 2H), 7.46 (d, *J* = 8.5 Hz, 2H), 6.95 (d, *J* = 2.4 Hz, 1H), 6.86 (d, *J* = 9.0 Hz, 1H), 6.66 (dd, *J* = 9.0 Hz, 2.4 Hz, 1H), 4.33 (t, *J* = 6.9 Hz, 2H), 3.83 (s, 3H), 3.66 (s, 2H), 2.38 (s, 3H), 2.00 (t, *J* = 6.9 Hz, 2H), 1.91 (s, 6H). ^{13}C NMR (101 MHz, $CDCl_3$) δ 170.5, 168.1, 155.9, 139.1, 135.8, 133.7, 131.0, 130.6, 130.4, 129.0, 114.8, 112.3, 111.5, 101.1, 65.0, 55.6, 47.8, 45.8, 38.2, 30.2, 13.3. MS (DART): m/z (%) 554.1 ([M+H]$^+$), 571.1 ([M+NH$_4$]$^+$, 100). HRMS (DART): Calculated for $C_{24}H_{26}ClINO_4$ ([M+H]$^+$): 554.0590; Found: 554.0579.

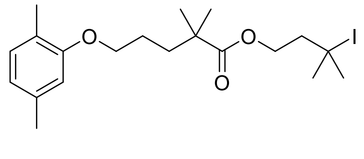

3-Iodo-3-methylbutyl 5-(2,5-dimethylphenoxy)-2,2-dimethylpentanoate (2k). The product (27 mmol scale, 6.0 g, 50% yield) as a yellow viscous oil was purified with silica gel chromatography (Hexane/Ethyl Acetate = 10/1). ^{1}H NMR (400 MHz, $CDCl_3$) δ 7.03 (d, *J* = 7.4 Hz, 1H), 6.69 (d, *J* = 7.5 Hz, 1H), 6.64 (s, 1H), 4.33 (t, *J* = 6.8 Hz, 2H), 3.97 – 3.91 (m, 2H), 2.34 (s, 3H), 2.22 (s, 3H), 2.04 (t, *J* = 6.8 Hz, 2H), 2.00 (s, 6H), 1.80 – 7.13 (m, 4H), 1.26 (s, 6H). ^{13}C NMR (101 MHz, $CDCl_3$) δ 177.6, 156.9, 136.4, 130.3, 123.5, 120.8, 67.8, 64.6, 48.1, 46.3, 42.0, 38.5, 37.1, 25.24,

25.20, 21.5, 15.9. MS (DART): m/z (%) 319.2 ([M-I]$^+$), 447.1 ([M+H]$^+$, 100), 464.2 ([M+NH$_4$]$^+$). HRMS (DART): Calculated for $C_{20}H_{32}IO_3$ ([M+H]$^+$): 447.1391; Found: 447.1387.

3. General Procedure for the Preparation of Arylzinc Reagents from Aryl Bromides

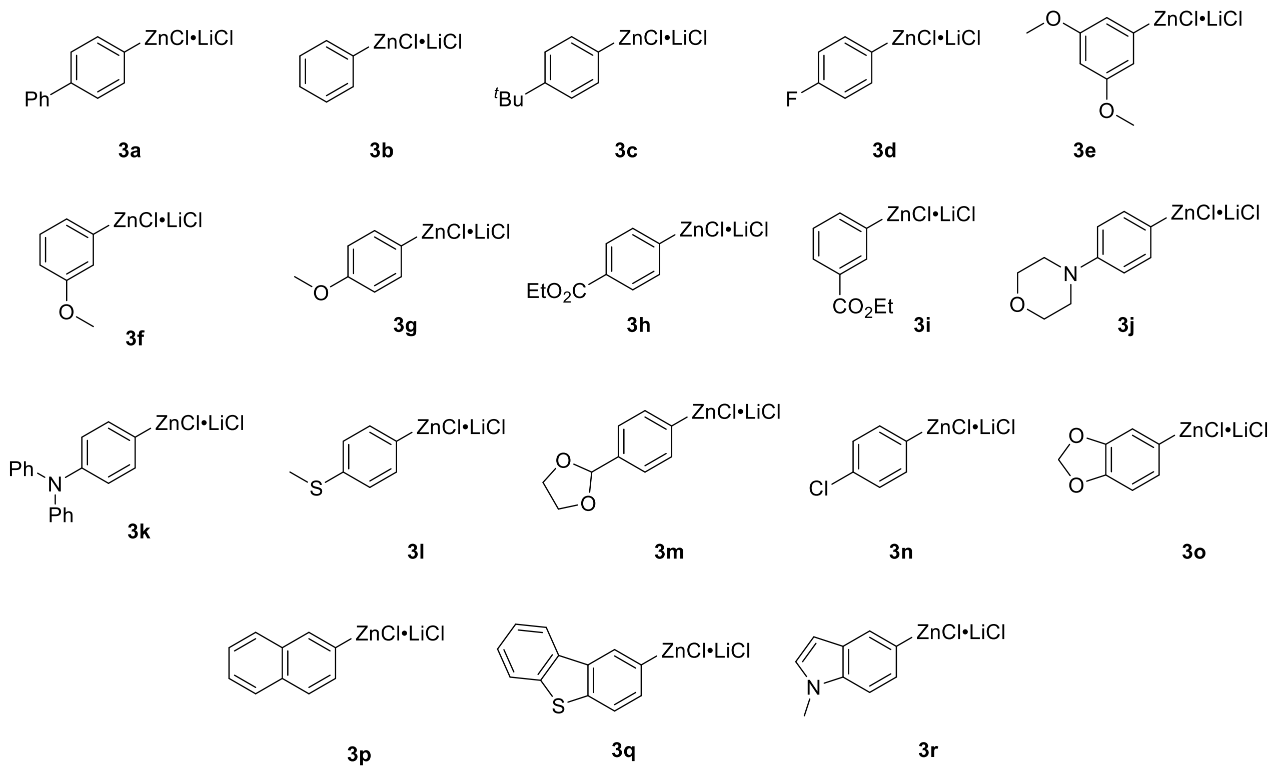

Supplementary Figure 2 Structure of Arylzinc Reagents

All arylzinc reagents are known compounds[6–8] and were prepared from the corresponding aryl bromides or iodides using Knochel's method. **3a-3f**, **3i-3q** were prepared by magnesium insertion-transmetallation process (**Method A**). **3g** and **3h** were prepared by iodine-magnesium exchange followed by transmetallation (**Method B**).

Method A

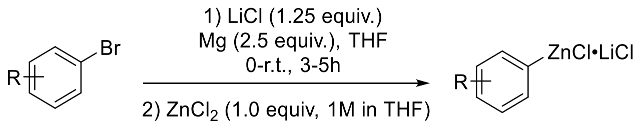

The synthesis of arylzinc reagents was according to the literature.[9] To a dry 100 mL Schlenk flask were added magnesium turnings (3 g, 125 mmol, 2.5 equiv) and LiCl (2.7 g, 62.5 mmol, 1.25 equiv).

The flask was evacuated and heated with a heatgun. The flask was evacuated again and backfilled with argon for 3 times. Upon cooling, tetrahydrofuran (anhydrous, 50 mL) was added, and the mixture was stirred vigorously for 5 min. DIBAL-H (1.0 M in hexane, 0.5 mL, 0.01 equiv) was added via syringe, and the mixture was stirred vigorously for 5 min. The flask was then cooled to 0 °C with an ice/water bath, and aryl bromide (50 mmol, 1.0 equiv) was added via a syringe. After 10 minutes, the ice/water bath was removed, and the mixture was stirred at room temperature for 3-5 h until all aryl bromide was consumed (the reaction was monitored by GC). After the reaction was completed, the resulting solution of Grignard reagent ArMgBr•LiCl was titrated with I_2 according to Knochel's method[10] to afford Grignard reagents with concentration typically ranging 0.3-0.5 M in THF. To a separate oven-dried 100 mL Schlenk tube, $ZnCl_2$ (1.0 M in THF) (***Note:*** the loading amount of $ZnCl_2$ (1 equiv) depends on the amount of Grignard reagents used for the reaction) was added. To this tube ArMgBr•LiCl was transferred via a syringe with stirring. After stirring for at least 10 min, the solution of ArZnCl•LiCl was titrated with I_2 according to Knochel's method. The Schlenk tube was sealed with a Teflon cap and the resulting aryl zinc reagents can be stored under argon at room temperature for several weeks.

Method B

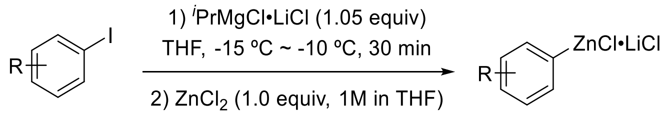

The procedure was a modification to the literature.[11] To a 50 mL Schlenk flask was added a THF solution of ^{i}PrMgCl·LiCl (1.5 M. 1.05 equiv) under Ar. The flask was cooled to -15 °C and a solution of aryl iodide (1 equiv) in THF was added dropwise. After complete addition, the temperature was raised to -10 °C and the reaction mixture was stirred for 30 min at this temperature. Then the resulting solution of Grignard reagent ArMgCl•LiCl was titrated with I_2 according to Knochel's method. To a separate oven-dried 100 mL Schlenk tube, $ZnCl_2$ (1.0 M in THF) (***Note:*** the loading amount of $ZnCl_2$ (1 equiv) depends on the amount of Grignard reagents used for the reaction) was added. To this tube ArMgBr•LiCl was transferred via a syringe with stirring. After stirring for at least 10 min, the solution of ArZnCl•LiCl was titrated with I_2 according to Knochel's method. The Schlenk tube was sealed with a Teflon cap and the resulting aryl zinc reagents can be stored under argon at room temperature for several weeks.

4. Optimization of Ni-Catalyzed Akylarylation of 3,3,3-Trifluoropropene 1 with Tertiary Alkyl Iodide 2a and Arylzinc Reagent 3a

To a 25 mL of Schlenck tube were added 4,4'-diMeO-2,2'-bpy (6 mol%), $NiCl_2 \cdot 6H_2O$ (5 mol%) and the monodentate phosphine ligand (PCy_2Ph or $t$$Bu_2MeP \cdot HBF_4$, 5 mol%). The tube was evacuated and backfilled with argon for 3 times, then tertiary alkyl iodide **2a** (0.4 mmol, 1.0 equiv) and TFP solution (1 M in DMA, 0.8 mmol, 2.0 equiv) were added under Ar. The resulting mixture was stirred for 20 min at room temperature, and the corresponding arylzinc reagent **3a** (0.6 mmol, 1.5 equiv) was added slowly within a period of 5 min, and the tube was sealed with a Teflon cap. After stirring for 12 h at room temperature, the reaction mixture was quenched with aqueous NH_4Cl solution and diluted with EtOAc. The reaction mixture was filtered through a pad of Celite, and the filtrate was extracted with EtOAc and washed with brine. The organic layer was dried over Na_2SO_4, filtered and concentrated. The residue was purified with silica gel chromatography to give the corresponding products **4a**. Isolated yields are based on the average of two runs under identical conditions.

Supplementary Table 1. Ligand effect on the nickel catalyzed alkylarylation of TFP **1** with alkyl iodide **2a** and arylzinc reagent **3a**[a]

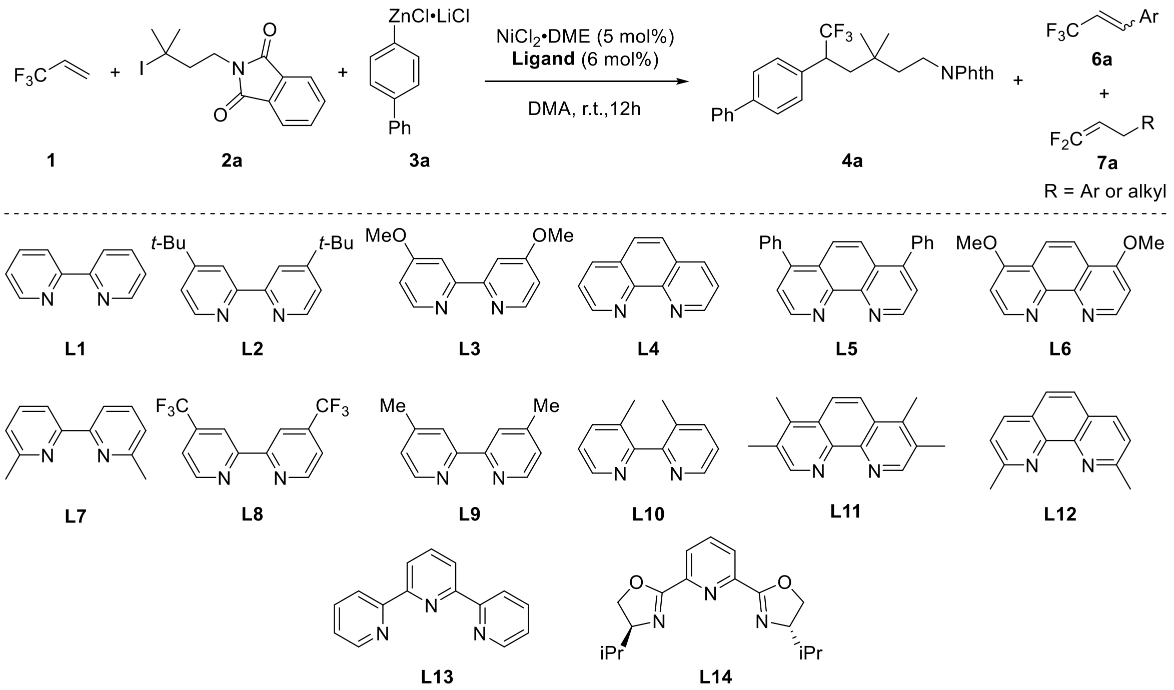

Entry	Ligand	Yield [%][b]		
		**6a**	**4a**	**7a**
1	**L1**	11	60	ND
3	**L2**	10	50	ND
4	**L3**	9	65	ND
8	**L4**	10	43	ND
9	**L5**	7	40	16
10	**L6**	10	22	6
7	**L7**	ND	8	28
2	**L8**	2	16	ND
5	**L9**	9	55	12
6	**L10**	11	60	14
11	**L11**	9	51	ND
12	**L12**	8	31	8
13	**L13**	15	ND	14
14	**L14**	11	ND	11

[a]Reaction conditions (unless otherwise specified): **2a** (0.4 mmol, 1.0 equiv), **1** (2.0 equiv, 1 M in DMA, 0.8 mL), **3a** (1.5 equiv), and DMA (2 mL). [b]Determined by ^{19}F NMR using fluorobenzene as an internal standard.

Supplementary Table 2. Screening of the nickel catalysts for alkylarylation of TFP **1** with alkyl iodide **2a** and arylzinc reagent **3a**[a]

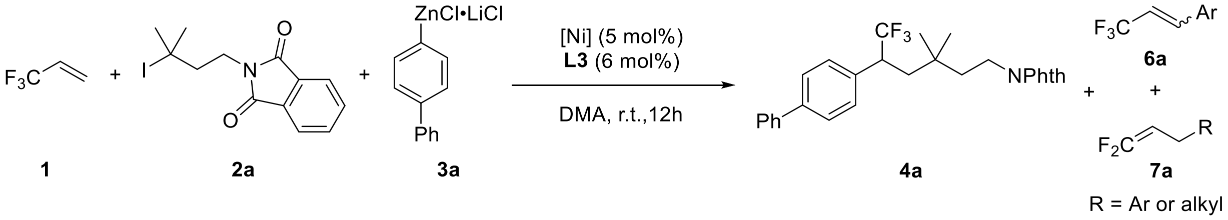

Entry	[Ni]	Yield [%][b]		
		**6a**	**4a**	**7a**
1	$NiCl_2$·DME	9	65	ND
2	$NiCl_2$	11	34	ND
3	$NiBr_2$·DME	11	60	ND
4	$NiBr_2$	10	22	ND
5	NiI_2	12	49	ND
6	$Ni(acac)_2$	10	60	9
7	$NiCl_2(PPh_3)_2$	5	68	9
8	$NiBr_2(PPh_3)_2$	4	68	11
9	$NiCl_2$(dppe)	6	19	5
10	$NiCl_2$(dppf)	ND	21	ND
11	*trans*-$NiPhCl(PPh_3)_2$ (**Ni-1**)	5	73	ND
12	*trans*-$NiCl_2(PCy_2Ph)_2$ (**Ni-2**)	ND	75	ND
13	$Ni(TMHD)_2$	12	65	ND
14	$Ni(COD)_2$	11	65	ND

[a]Reaction conditions (unless otherwise specified): **2a** (0.4 mmol, 1.0 equiv), **1** (2.0 equiv, 1 M in DMA, 0.8 mL), **3a** (1.5 equiv), and DMA (2 mL). [b]Determined by ^{19}F NMR using fluorobenzene as an internal standard.

Supplementary Table 3. Solvent effects on the nickel catalysts for alkylarylation of TFP **1** with alkyl iodide **2a** and arylzinc reagent **3a**[a]

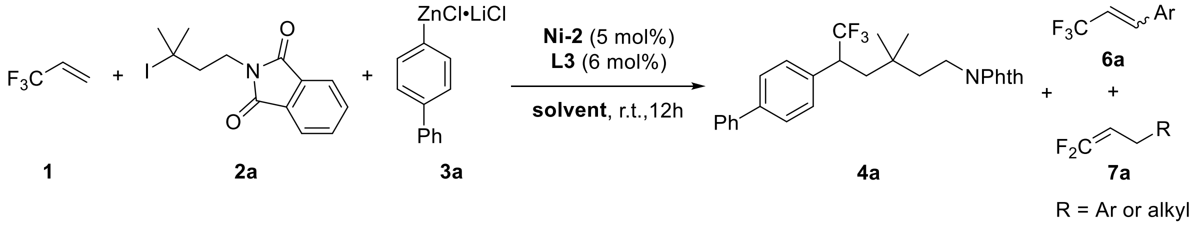

Entry	Solvent	Yield [%][b]		
		**6a**	**4a**	**7a**
1	DMA	ND	75	ND
2	DMF	ND	44	ND
3	THF	ND	5	14
4	dioxane	ND	11	34
5	Toluene	ND	ND	12
6	MeCN	ND	ND	18

[a]Reaction conditions (unless otherwise specified): **2a** (0.4 mmol, 1.0 equiv), **1** (2.0 equiv, 1 M in DMA, 0.8 mL), **3a** (1.5 equiv), and Solvent (2 mL). [b]Determined by ^{19}F NMR using fluorobenzene as an internal standard.

Supplementary Table 4. Control experiments[a]

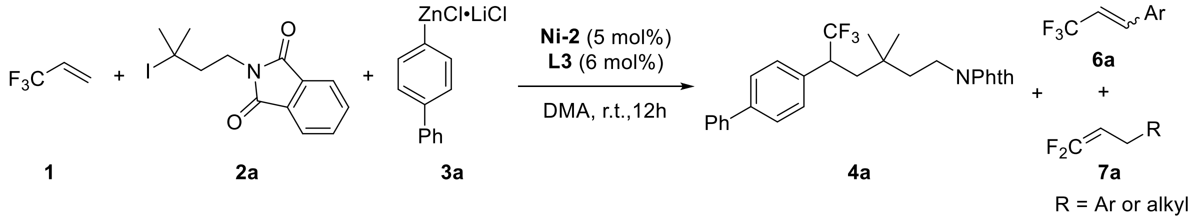

Entry	[Ni]	L3	Yield [%][b]		
			**6a**	**4a**	**7a**
1	**Ni-2**	**L3**	ND	75	ND
2	none	**L3**	ND	ND	ND
3	**Ni-2**	none	ND	ND	47
4	none	none	ND	ND	ND

[a]Reaction conditions (unless otherwise specified): **2a** (0.4 mmol, 1.0 equiv), **1** (2.0 equiv, 1 M in DMA, 0.8 mL), **3a** (1.5 equiv), and DMA (2 mL). [b]Determined by ^{19}F NMR using fluorobenzene as an internal standard.

Supplementary Table 5. Screening of the nickel catalysts in the presence of phosphine ligand for nickel-catalyzed alkylarylation of TFP **1** with alkyl iodide **2a** and arylzinc reagent **3a**[a]

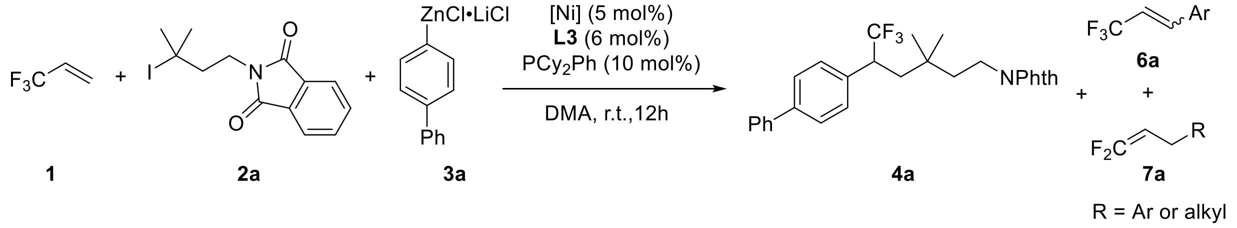

Entry	[Ni]	Yield [%][b]		
		**6a**	**4a**	**7a**
1	$Ni(acac)_2$	ND	64	8
2	$Ni(NO_3)_2 \cdot 6H_2O$	ND	73	ND
3	$NiClO_4 \cdot H_2O$	ND	74	4
4	$NiBr_2 \cdot 3H_2O$	ND	74	10
5	$NiCl_2 \cdot 6H_2O$	ND	77	ND
6	$Ni(OTf)_2$	ND	43	6

[a]Reaction conditions (unless otherwise specified): **2a** (0.4 mmol, 1.0 equiv), **1** (2.0 equiv, 1 M in DMA, 0.8 mL), **3a** (1.5 equiv), and DMA (2 mL). [b]Determined by ^{19}F NMR using fluorobenzene as an internal standard.

Supplementary Table 6. Screening of the loading amount of PCy_2Ph for the nickel-catalyzed alkylarylation of TFP **1** with alkyl iodide **2a** and arylzinc reagent **3a**[a]

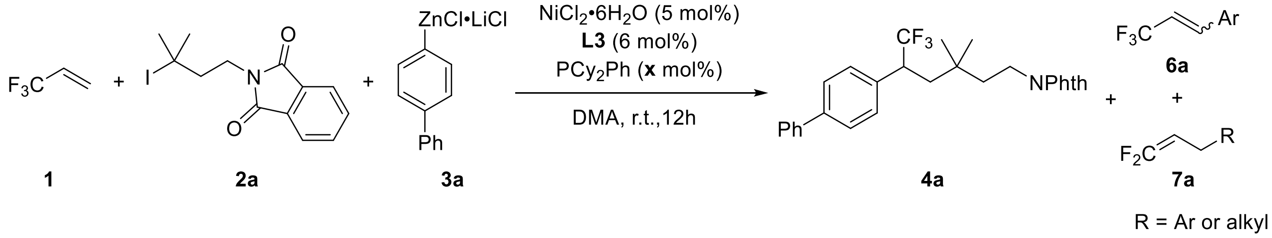

Entry	x (mol%)	Yield [%][b]		
		**6a**	**4a**	**7a**
1	2.5	ND	78	ND
2	5	ND	84	ND
3	10	ND	77	ND
4	15	ND	81	ND
5	20	ND	81	ND

[a]Reaction conditions (unless otherwise specified): **2a** (0.4 mmol, 1.0 equiv), **1** (2.0 equiv, 1 M in DMA, 0.8 mL), **3a** (1.5 equiv), and DMA (2 mL). [b]Determined by ^{19}F NMR using fluorobenzene as an internal standard.

Supplementary Table 7. Effects of the phosphine ligands on the nickel-catalyzed alkylarylation of TFP **1** with alkyl iodide **2a** and arylzinc reagent **3a**[a]

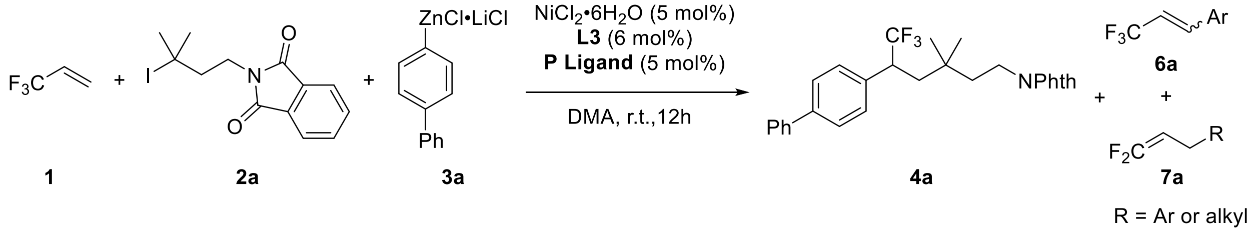

Entry	P Ligand	Yield [%][b]		
		**6a**	**4a**	**7a**
1	PCy_2Ph	ND	84 (82)	ND
2	P(Ad$_2$*n*-Bu)·HI	ND	67	8
3	P(*t*-Bu$_2$Ph)·HBF_4	ND	56	5
4	P(2-MeO-Ph)$_3$	ND	50	6
5	P(4-MeO-Ph)$_3$	ND	69	9
6	P(2-Me-Ph)$_3$	ND	52	6
7	P(4-Me-Ph)$_3$	ND	66	8
8	P(*t*-Bu$_2$Me)·HBF_4	ND	75	5
9	PCy_3	ND	72	ND
10	$PCyPh_2$	ND	66	7
11	$AsPh_3$	ND	50	6
12	none	ND	52	ND

[a]Reaction conditions (unless otherwise specified): **2a** (0.4 mmol, 1.0 equiv), **1** (2.0 equiv, 1 M in DMA, 0.8 mL), **3a** (1.5 equiv), and DMA (2 mL). [b]Determined by ^{19}F NMR using fluorobenzene as an internal standard.

Supplementary Table 8. Effects of the pyridine-based ligands on the nickel-catalyzed alkylarylation of TFP **1** with alkyl iodide **2a** and arylzinc reagent **3a**[a]

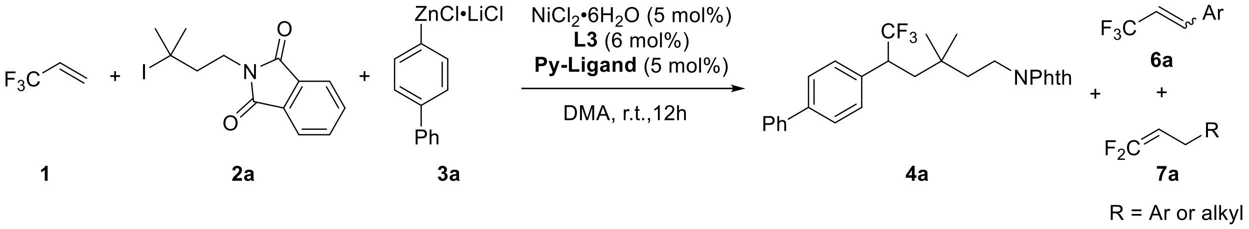

Entry	Py-Ligand	Yield [%][b]	
		**4a**	**7a**
1	4-MeO-Py	56	4
2	4-Ph-Py	51	6
3	4-CF_3-Py	52	6
4	DMAP	55	5

[a]Reaction conditions (unless otherwise specified): **2a** (0.4 mmol, 1.0 equiv), **1** (2.0 equiv, 1 M in DMA, 0.8 mL), **3a** (1.5 equiv), and DMA (2 mL). [b]Determined by ^{19}F NMR using fluorobenzene as an internal standard.

Supplementary Table 9. Effects of DME on the nickel-catalyzed alkylarylation of TFP **1** with alkyl iodide **2a** and arylzinc reagent **3a**[a]

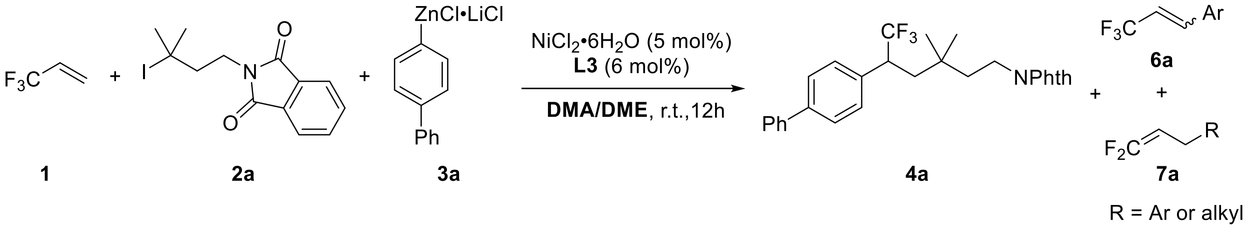

Entry	DMA/DME (v/v)	Yield [%][b]	
		**4a**	**7a**
1	10:1	54	2
2	5:1	56	2
3	2:1	60	3
4	1:1	66	2
5	1:2	73	5
6	1:3	71	5

[a]Reaction conditions (unless otherwise specified): **2a** (0.4 mmol, 1.0 equiv), **1** (2.0 equiv, 1 M in DMA, 0.8 mL), **3a** (1.5 equiv), and DMA+DME (2 mL). [b]Determined by ^{19}F NMR using fluorobenzene as an internal standard.

Supplementary Table 10. Effects of the phosphine ligands on the nickel-catalyzed alkylarylation of TFP **1** with alkyl iodide **2a** and electron-deficient arylzinc reagent **3d**[a]

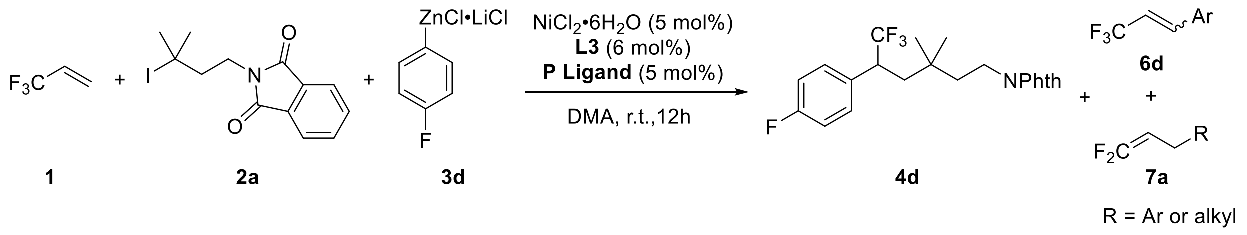

Entry	P Ligand	Yield [%][b]		
		**6d**	**4d**	**7a**
1	PCy_2Ph	ND	41	5
2	P(Ad_2*n*-Bu)·HI	ND	54	4
3	P(*t*-Bu_2Ph)·HBF_4	ND	27	3
4	$P(2\text{-MeO-Ph})_3$	ND	25	4
5	$P(4\text{-MeO-Ph})_3$	ND	41	2
6	$P(2\text{-Me-Ph})_3$	ND	26	4
7	$P(4\text{-Me-Ph})_3$	ND	50	4
8	P(*t*-Bu_2Me)·HBF_4	ND	60 (58)	ND
9	PCy_3	ND	56	4
10	$PCyPh_2$	ND	46	3

[a]Reaction conditions (unless otherwise specified): **2a** (0.4 mmol, 1.0 equiv), **1** (2.0 equiv, 1 M in DMA, 0.8 mL), **3d** (1.5 equiv), and DMA (2 mL). [b]Determined by ^{19}F NMR using fluorobenzene as an internal standard.

Supplementary Table 11. Effects of DME on the nickel-catalyzed alkylarylation of TFP **1** with alkyl iodide **2a** and electron-deficient arylzinc reagent **3d**[a]

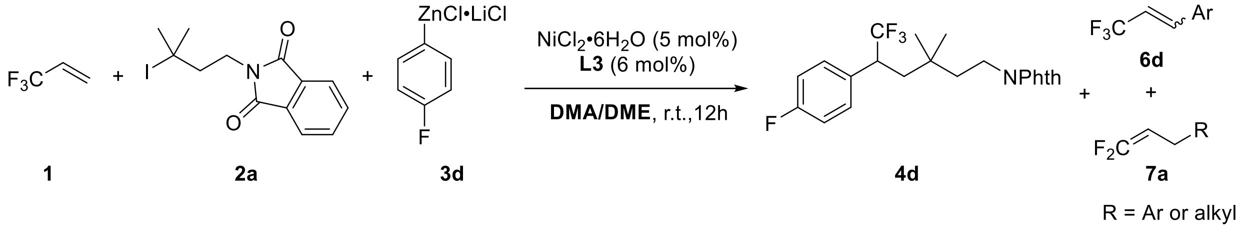

Entry	DMA/DME (v/v)	Yield [%][b]	
		**4d**	**7a**
1	10:1	30	3
2	5:1	31	3
3	2:1	30	3
4	1:1	34	3
5	1:2	48	9
6	1:3	50	11

[a]Reaction conditions (unless otherwise specified): **2a** (0.4 mmol, 1.0 equiv), **1** (2.0 equiv, 1 M in DMA, 0.8 mL), **3d** (1.5 equiv), and DMA+DME (2 mL). [b]Determined by ^{19}F NMR using fluorobenzene as an internal standard.

5. General Procedure for the Ni-Catalyzed Alkylarylation of 3,3,3-Trifluoropropene 1 with Tertiary Alkyl Iodides 2 and Arylzinc Reagents 3

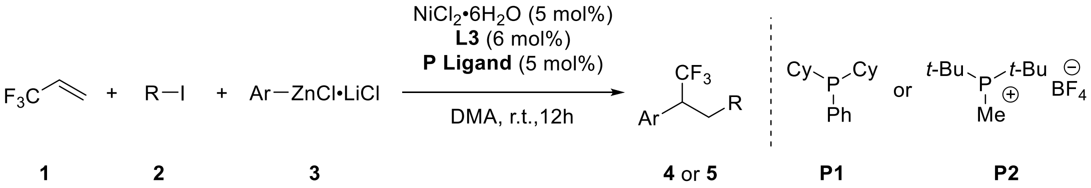

To a 25 mL of Schlenck tube were added 4,4'-diMeO-2,2'-bpy (6 mol%), $NiCl_2\cdot 6H_2O$ (5 mol%) and the monodentate phosphine ligand (PCy_2Ph or $P^tBu_2Me\cdot HBF_4$, 5 mol%). The tube was evacuated and backfilled with argon for 3 times, then tertiary alkyl iodide **2** (0.4 mmol, 1.0 equiv) and TFP solution (1 M in DMA, 0.8 mmol, 2.0 equiv) were added under Ar. The resulting mixture was stirred for 20 min at room temperature, and the corresponding arylzinc reagent **3** (0.6 mmol, 1.5 equiv) was added slowly within a period of 5 min, and the tube was sealed with a Teflon cap. After stirring for 12 h at room temperature, the reaction mixture was quenched with aqueous NH_4Cl solution and diluted with EtOAc. The reaction mixture was filtered through a pad of Celite, and the filtrate was extracted with

EtOAc and washed with brine. The organic layer was dried over Na_2SO_4, filtered and concentrated. The residue was purified with silica gel chromatography to give the corresponding products **4** or **5.** Isolated yields are based on the average of two runs under identical conditions.

6. Characterization Data for Compounds 4 and 5

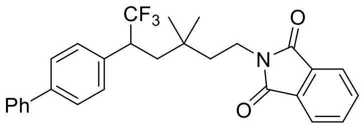

2-(5-([1,1'-Biphenyl]-4-yl)-6,6,6-trifluoro-3,3-dimethylhexyl)isoindoline-1,3-dione (4a). The product (standard reaction condtions: 153 mg, 82% yield using **P1;** using 2.5 mol% nickel catalyst on 0.4 mmol scale: 55% determined by ^{19}F NMR) as a yellow solid (m.p. 108.4 – 109.7 °C) was purified with silica gel chromatography (Hexane/Ethyl Acetate = 10/1). ^{1}H NMR (400 MHz, $CDCl_3$) δ 7.88 – 7.77 (m, 2H), 7.74 – 7.65 (m, 2H), 7.63 – 7.52 (m, 4H), 7.49 – 7.39 (m, 4H) 7.37 – 7.30 (m, 1H), 3.75 – 3.60 (m, 2H), 3.59 – 3.46 (m, 1H), 2.17 – 1.97 (m, 2H), 1.69 – 1.58 (m, 1H), 1.58 – 1.46 (m, 1H), 0.91 (s, 3H), 0.87 (s, 3H). ^{19}F NMR (376 MHz, $CDCl_3$) δ -70.1 (d, J = 9.6 Hz, 3F). ^{13}C NMR (101 MHz, $CDCl_3$) δ 168.1, 140.7, 140.3, 135.3, 133.8, 132.0, 129.7, 128.7, 127.3, 127.2, 127.1 (q, J = 281.3 Hz), 126.9, 123.0, 46.0 (q, J = 26.4 Hz), 39.9, 39.8, 33.8, 32.8, 27.3, 27.1. MS (DART): m/z (%) 466.2 ($[M+H]^+$), 483.2 ($[M+NH_4]^+$, 100). HRMS (DART): Calculated for $C_{28}H_{27}F_3NO_2$ ($[M+H]^+$): 466.1988; Found: 466.1985.

Gram-scale synthesis of compound 4a

To a 100 mL of Schlenck tube were added 4,4'-diMeO-2,2'-bpy (3 mol%), $NiCl_2·6H_2O$ (2.5 mol%) and the monodentate phosphine ligand PCy_2Ph (2.5 mol%). The tube was evacuated and backfilled with argon for 3 times, then tertiary alkyl iodide **2a** (6 mmol, 1.0 equiv) and TFP solution (1 M in DMA, 25 mL) were added under Ar. The resulting mixture was stirred for 20 min at room temperature, and the corresponding arylzinc reagent **3a** (9 mmol, 1.5 equiv) was added slowly within a period of 5 min, and the tube was sealed with a Teflon cap. After stirring for 12 h at room temperature, the reaction mixture was quenched with aqueous NH_4Cl solution and diluted with EtOAc. The reaction mixture was filtered through a pad of Celite, and the filtrate was extracted with EtOAc and washed with brine. The organic layer was dried over Na_2SO_4, filtered and concentrated. The residue was purified with

silica gel chromatography (Hexane/Ethyl Acetate = 15/1) to give compound **4a** as a yellow solid (1.4 g, 50% yield).

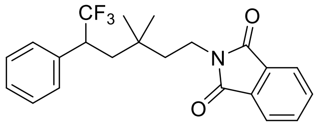

2-(6,6,6-Trifluoro-3,3-dimethyl-5-phenylhexyl)isoindoline-1,3-dione (4b). The product (107 mg, 69% yield using **P2**) as a yellow oil was purified with silica gel chromatography (Hexane/Ethyl Acetate = 10/1). ^{1}H NMR (400 MHz, $CDCl_3$) δ 7.83 – 7.77 (m, 2H), 7.69 – 7.63 (m, 2H), 7.37 – 7.23 (m, 5H), 3.68 – 3.56 (m, 2H), 3.51 – 3.38 (m, 1H), 2.04 – 1.95 (m, 2H), 1.63 – 1.53 (m, 1H), 1.53 – 1.44 (m, 1H), 0.83 (s, 3H), 0.79 (s, 3H). ^{19}F NMR (376 MHz, $CDCl_3$) δ -70.2 (d, *J* = 9.8 Hz, 3F). ^{13}C NMR (101 MHz, $CDCl_3$) δ 168.1, 136.3, 133.8, 132.0, 129.3, 128.5, 127.9, 127.1 (q, *J* = 281.3 Hz) 123.0, 46.3 (q, *J* = 26.3 Hz), 39.9, 39.8, 33.8, 32.7, 27.3, 27.0. MS (DART): m/z (%) 390.2 ($[M+H]^+$), 407.2 ($[M+NH_4]^+$, 100). HRMS (DART): Calculated for $C_{22}H_{23}F_3NO_2$ ($[M+H]^+$): 390.1675; Found: 390.1673.

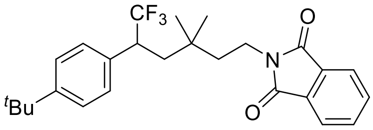

2-(5-(4-(*tert*-Butyl)phenyl)-6,6,6-trifluoro-3,3-dimethylhexyl)isoindoline-1,3-dione (4c). The product (standard reaction conditions: 142.6 mg, 80% yield using **P2**; using **L2** instead of **L3** as the ligand: 71% determined by ^{19}F NMR) as a white solid (m.p. 114.8 – 116.2°C) was purified with silica gel chromatography (Hexane/Ethyl Acetate = 15/1). ^{1}H NMR (400 MHz, $CDCl_3$) δ 7.76 – 7.70 (m, 2H), 7.62 – 7.55 (m, 2H), 7.28 (d, *J* = 8.3 Hz, 2H), 7.23 (d, *J* = 8.2 Hz, 2H), 3.64 – 3.52 (m, 2H), 3.47 – 3.34 (m, 1H), 2.00 – 1.92 (m, 2H), 1.58 – 1.47 (m, 1H), 1.47 – 1.35 (m, 1H), 1.21 (s, 9H), 0.82 (s, 3H), 0.79 (s, 3H). ^{19}F NMR (376 MHz, $CDCl_3$) δ -70.1 (d, *J* = 9.9 Hz, 3F). ^{13}C NMR (126 MHz, $CDCl_3$) δ 168.0, 150.9, 133.8, 133.2, 132.2, 129.0, 127.3 (q, *J* = 280.1 Hz), 125.5, 123.0, 46.0 (q, *J* = 26.1 Hz), 40.1, 39.9, 34.4, 33.9, 32.8, 31.2, 27.1, 27.1. MS (ESI): 468.2 ($[M+Na]^+$). HRMS (ESI): Calculated for $C_{26}H_{30}F_3NNaO_2$ ($[[M+Na]^+]$): 468.2121; Found: 468.21208.

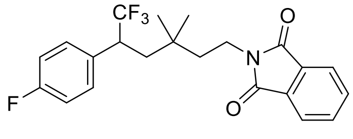

2-(6,6,6-Trifluoro-5-(4-fluorophenyl)-3,3-dimethylhexyl)isoindoline-1,3-dione (4d). The product (94 mg, 58% yield using **P2**) as a yellow oil was purified with silica gel chromatography (Hexane/Ethyl Acetate = 15/1). ^{1}H NMR (400 MHz, $CDCl_3$) δ 7.86 – 7.79 (m, 2H),

7.73 – 7.66 (m, 2H), 7.36 – 7.28 (m, 2H), 7.02 (t, *J* = 8.5 Hz, 2H), 3.70 – 3.56 (m, 2H), 3.53 – 3.40 (m, 1H), 2.05 – 1.89 (m, 2H), 1.64 – 1.43 (m, 3H), 0.85 (s, 3H), 0.80 (s, 3H). ^{19}F NMR (376 MHz, $CDCl_3$) δ -70.5 (d, *J* = 9.7 Hz, 3F), -114.1 – -114.2 (m, 1F). ^{13}C NMR (101 MHz, $CDCl_3$) δ 168.1, 162.4 (q, *J* = 248.1 Hz), 133.9, 132.0, 130.9 (d, *J* = 8.1 Hz), 126.9 (q, *J* = 281.79 Hz), 123.1, 115.6 (d, *J* = 21.5 Hz), 45.6 (q, *J* = 26.6 Hz), 40.0, 39.7, 33.8, 32.7, 27.3, 27.1. MS (DART): m/z (%) 408.2 ($[M+H]^+$, 100), 425.2 ($[M+NH_4]^+$). HRMS (DART): Calculated for $C_{22}H_{22}F_4NO_2$ ($[M+H]^+$): 408.1581; Found: 408.1577.

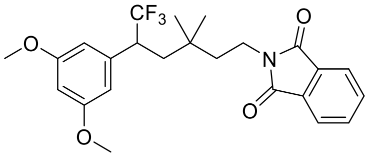

2-(5-(3,5-Dimethoxyphenyl)-6,6,6-trifluoro-3,3-dimethylhexyl)isoindoline-1,3-dione (4e). The product (108 mg, 60% yield using **P2**) as a yellow oil was purified with silica gel chromatography (Hexane/Ethyl Acetate = 5/1). ^{1}H NMR (400 MHz, $CDCl_3$) δ 7.83 – 7.76 (m, 2H), 7.70 – 7.63 (m, 2H), 6.49 (s, 2H), 6.37 (s, 1H), 3.77 (s, 6H), 3.68 – 3.56 (m, 2H), 3.45 – 3.30 (m, 1H), 1.96 (d, *J* = 6.0 Hz, 2H), 1.67 – 1.45 (m, 2H), 0.87 (s, 3H), 0.84 (s, 3H). ^{19}F NMR (376 MHz, $CDCl_3$) δ -70.0 (d, *J* = 9.7 Hz, 3F). ^{13}C NMR (101 MHz, $CDCl_3$) δ 168.1, 160.6, 138.5, 133.8, 132.0, 127.0 (q, *J* = 281.6 Hz), 123.0, 107.6, 99.5, 55.2, 46.5 (q, *J* = 26.4 Hz), 39.9, 39.8, 33.8, 32.7, 27.2, 26.9. MS (DART): m/z (%) 450.2 ($[M+H]^+$, 100), 467.2 ($[M+NH_4]^+$). HRMS (DART): Calculated for $C_{24}H_{27}F_3NO_4$ ($[M+H]^+$): 450.1887; Found: 450.1884.

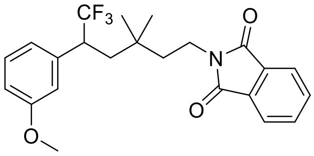

2-(6,6,6-Trifluoro-5-(3-methoxyphenyl)-3,3-dimethylhexyl)isoindoline-1,3-dione (4f). The product (117 mg, 70% yield using **P1**) as a yellow oil was purified with silica gel chromatography (Hexane/Ethyl Acetate = 10/1). ^{1}H NMR (400 MHz, $CDCl_3$) δ 7.83 – 7.76 (m, 2H), 7.69 – 7.63 (m, 2H), 7.23 (t, *J* = 7.9 Hz, 1H), 6.97 – 6.87 (m, 2H), 6.81 (dd, *J* = 8.1 Hz, 2.2 Hz, 1H), 3.78 (s, 3H), 3.67 – 3.57 (m, 2H), 3.51 – 3.36 (m, 1H), 1.99 (d, *J* = 5.9 Hz, 2H), 1.66 – 1.44 (m, 2H), 0.85 (s, 3H), 0.82 (s, 3H). ^{19}F NMR (376 MHz, $CDCl_3$) δ -70.1 (d, *J* = 9.7 Hz, 3F). ^{13}C NMR (101 MHz, $CDCl_3$) δ 168.1, 159.5, 137.8, 133.8, 132.0, 129.5, 127.0 (q, J = 281.2 Hz), 123.0, 121.7, 115.2, 113.0, 55.0, 46.3 (q, *J* = 26.4 Hz), 39.9, 39.8, 33.8, 32.7, 27.2, 26.9. MS (DART): m/z (%) 420.2 ($[M+H]^+$), 437.2 ($[M+NH_4]^+$, 100). HRMS (DART): Calculated for $C_{23}H_{25}F_3NO_3$ ($[M+H]^+$): 420.1781; Found: 420.1176.

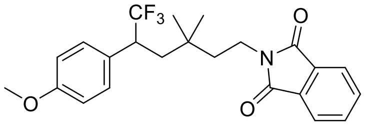

2-(6,6,6-Trifluoro-5-(4-methoxyphenyl)-3,3-dimethylhexyl) isoindoline-1,3-dione (4g). The product (96 mg, 57% yield using **P1**) as a white solid (m.p. 88.7 – 89.9 °C) was purified with silica gel chromatography (Hexane/Ethyl Acetate = 10/1). ^{1}H NMR (400 MHz, $CDCl_3$) δ 7.86 – 7.76 (m, 2H), 7.71 – 7.62 (m, 2H), 7.25 (d, *J* = 8.5 Hz, 2H), 6.85 (d, *J* = 8.3 Hz, 2H), 3.75 (s, 3H), 3.68 – 3.56 (m, 2H), 3.48 – 3.33 (m, 1H), 2.02 – 1.89 (m, 2H), 1.66 – 1.40 (m, 2H), 0.84 (s, 3H), 0.80 (s, 3H). ^{19}F NMR (376 MHz, $CDCl_3$) δ -70.6 (d, *J* = 9.8 Hz). ^{13}C NMR (101 MHz, $CDCl_3$) δ 168.1, 159.2, 133.8, 132.0, 130.2, 128.1, 127.2 (q, *J* = 281.3 Hz), 123.0, 113.9, 55.0, 45.4 (q, *J* = 26.5 Hz), 39.8, 33.8, 32.6, 27.3, 27.0. MS (DART): m/z (%) 420.2 ($[M+H]^+$), 437.2 ($[M+NH_4]^+$, 100). HRMS (DART): Calculated for $C_{23}H_{25}F_3NO_3$ ($[M+H]^+$): 420.1781; Found: 420.1778.

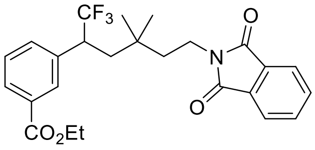

Ethyl 3-(6-(1,3-dioxoisoindolin-2-yl)-1,1,1-trifluoro-4,4-dimethylhexan-2-yl)benzoate (4h). The product (74 mg, 40% yield using **P2**) as a colorless oil was purified with silica gel chromatography (Hexane/Ethyl Acetate = 10/1). ^{1}H NMR (400 MHz, $CDCl_3$) δ 8.04 – 7.94 (m, 2H), 7.82 – 7.76 (m, 2H), 7.69 – 7.63 (m, 2H), 7.54 (d, *J* = 7.7 Hz, 1H), 7.40 (t, *J* = 7.7 Hz, 1H), 4.35 (q, *J* = 7.1 Hz, 2H), 3.68 – 3.58 (m, 2H), 3.57 – 3.45 (m, 1H), 2.07 – 1.97 (m, 2H), 1.63 – 1.43 (m, 2H), 1.37 (t, *J* = 7.1 Hz, 3H), 0.82 (s, 3H), 0.79 (s, 3H). ^{19}F NMR (376 MHz, $CDCl_3$) δ -70.2 (d, *J* = 9.6 Hz). ^{13}C NMR (101 MHz, $CDCl_3$) δ 168.0, 166.0, 136.8, 133.8, 133.6, 132.0, 130.9, 130.4, 129.2, 128.7, 126.8 (q, *J* = 281.3 Hz), 123.0, 61.0, 46.2 (q, J = 26.7 Hz), 39.9, 39.9, 33.8, 32.8, 27.3, 26.9, 14.2. MS (DART): m/z (%) 462.2 ($[M+H]^+$, 100), 479.2 ($[M+NH_4]^+$); HRMS (DART): Calculated for $C_{25}H_{27}F_3NO_4$ ($[M+H]^+$): 462.1887; Found: 462.1879.

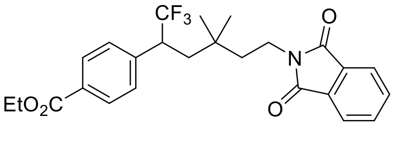

Ethyl 4-(6-(1,3-dioxoisoindolin-2-yl)-1,1,1-trifluoro-4,4-dimethylhexan-2-yl)benzoate (4i). The product (105 mg, 57% yield using **P2**) as a colorless oil was purified with silica gel chromatography (Hexane/Ethyl Acetate = 10/1). ^{1}H NMR (400 MHz, $CDCl_3$) δ 7.98 (d, *J* = 8.2 Hz, 2H), 7.79 – 7.73 (m, 2H), 7.67 – 7.60 (m, 2H), 7.41 (d, *J* = 8.2 Hz, 2H), 4.31 (q, *J* = 7.1 Hz, 2H), 3.68 – 3.46 (m, 3H), 2.06 – 1.92 (m, 2H), 1.62 – 1.40 (m, 2H), 1.33 (t, *J* = 7.1 Hz, 3H), 0.81 (s, 3H), 0.75

(s, 3H). ^{19}F NMR (376 MHz, $CDCl_3$) δ -70.0 (d, *J* = 9.6 Hz). ^{13}C NMR (101 MHz, $CDCl_3$) δ 168.0, 165.9, 141.2, 133.7, 132.0, 130.2, 129.7, 129.3, 126.7 (q, *J* = 282.2 Hz), 123.0, 60.8, 46.3 (q, *J* = 26.5 Hz), 39.8, 39.7, 33.7, 32.7, 27.2, 27.0, 14.1. MS (DART): m/z (%) 408.1 (100), 462.2 ($[M+H]^+$). HRMS (DART): Calculated for $C_{25}H_{27}F_3NO_4$ ($[M+H]^+$): 462.1887; Found: 462.1886.

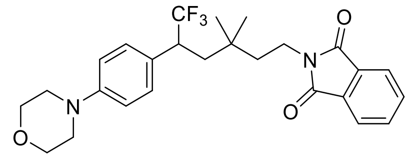

2-(6,6,6-Trifluoro-3,3-dimethyl-5-(4-morpholinophenyl)hexyl)isoindoline-1,3-dione (4j). The product (131 mg, 69% yield using **P2**) as a yellow solid (m.p. 115.5 – 117.6 °C) was purified with silica gel chromatography (Hexane/Ethyl Acetate = 5/1). ^{1}H NMR (400 MHz, $CDCl_3$) δ 7.83 – 7.75 (m, 2H), 7.70 – 7.61 (m, 2H), 7.21 (d, *J* = 8.3 Hz, 2H), 6.83 (d, *J* = 8.3 Hz, 2H), 3.85 – 3.74 (m, 4H), 3.68 – 3.53 (m, 2H), 3.44 – 3.28 (m, 1H), 3.15 – 3.05 (m, 4H), 1.99 – 1.87 (m, 2H), 1.64 – 1.38 (m, 2H), 0.84 (s, 3H), 0.80 (s, 3H). ^{19}F NMR (376 MHz, $CDCl_3$) δ -70.5 (d, *J* = 9.8 Hz). ^{13}C NMR (101 MHz, $CDCl_3$) δ 168.0, 150.6, 133.7, 131.9, 129.9, 127.2 (q, *J* = 281.2 Hz), 127.0, 122.9, 115.2, 66.6, 48.7, 45.3 (q, *J* = 26.4 Hz), 39.7, 39.7, 33.8, 32.6, 27.2, 27.0. MS (DART): m/z (%) 475.2 ($[M+H]^+$, 100). HRMS (DART): Calculated for $C_{26}H_{30}F_3N_2O_3$ ($[M+H]^+$): 475.2203; Found: 475.2197.

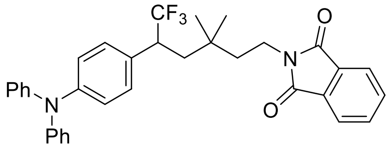

2-(5-(4-(Diphenylamino)phenyl)-6,6,6-trifluoro-3,3-dimethylhexyl)isoindoline-1,3-dione (4k). The product (169 mg, 76% yield using **P2**) as a dark green oil was purified with silica gel chromatography (Hexane/Ethyl Acetate = 10/1). ^{1}H NMR (400 MHz, $CDCl_3$) δ 7.85 – 7.77 (m, 2H), 7.70 – 7.62 (m, 2H), 7.25 – 7.15 (m, 6H), 7.09 – 6.94 (m, 8H), 3.73 – 3.56 (m, 2H), 3.50 – 3.33 (m, 1H), 2.04 – 1.91 (m, 2H), 1.68 – 1.45 (m, 2H), 0.91 (s, 3H), 0.88 (s, 3H). ^{19}F NMR (376 MHz, $CDCl_3$) δ -70.2 (d, *J* = 9.8 Hz). ^{13}C NMR (101 MHz, $CDCl_3$) δ 168.0, 147.4, 133.7, 132.1, 130.0, 129.7, 129.14, 127.1 (q, *J* = 281.2 Hz), 124.4, 123.1, 123.0 122.9, 45.6 (q, *J* = 26.5 Hz), 40.0, 39.8, 33.9, 32.7, 27.1, 27.1. MS (DART): m/z (%) 557.2 ($[M+H]^+$, 100). HRMS (DART): Calculated for $C_{34}H_{32}F_3N_2O_2$ ($[M+H]^+$): 557.2410; Found: 557.2396.

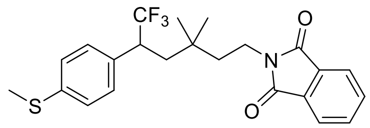

2-(6,6,6-Trifluoro-3,3-dimethyl-5-(4-(methylthio)phenyl)hexyl)isoindoline-1,3-dione (4l). The product (97 mg, 56% yield using **P2**) as a yellow solid (m.p. 88.9 – 90.4 °C) was purified with silica gel chromatography (Hexane/Ethyl Acetate = 5/1). ^{1}H NMR (400 MHz, $CDCl_3$) δ 7.81 – 7.74 (m, 2H), 7.68 – 7.61 (m, 2H), 7.24 (d, *J* = 8.4 Hz, 2H), 7.17 (d, *J* = 8.4 Hz, 2H), 3.67 – 3.54 (m, 2H), 3.48 – 3.34 (m, 1H), 2.41 (s, 3H), 2.02 – 1.92 (m, 2H), 1.61 – 1.41 (m, 2H), 0.83 (s, 3H), 0.79 (s, 3H). ^{19}F NMR (376 MHz, $CDCl_3$) δ -70.3 (d, *J* = 9.7 Hz). ^{13}C NMR (101 MHz, $CDCl_3$) δ 168.0, 138.4, 133.7, 132.8, 132.0, 129.6, 127.0 (q, *J* = 281.4 Hz), 126.3, 122.9, 45.7 (q, *J* = 26.4 Hz), 39.7, 33.8, 32.6, 27.2, 27.0, 15.3. MS (DART): m/z (%) 436.2 ($[M+H]^+$), 453.2 ($[M+NH_4]^+$, 100). HRMS (DART): Calculated for $C_{23}H_{25}F_3NO_2S$ ($[M+H]^+$); 436.1553; Found: 436.1549.

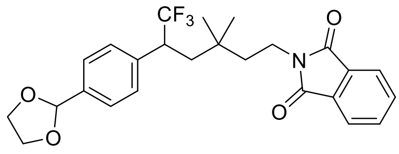

2-(5-(4-(1,3-Dioxolan-2-yl)phenyl)-6,6,6-trifluoro-3,3-dimethylhexyl)isoindoline-1,3-dione (4m). The product (129 mg, 70% yield using **P1**) as a colorless oil was purified with silica gel chromatography (Hexane/Ethyl Acetate = 10/1). (***Note***: this compound is prone to deterioration during silica gel chromatography) ^{1}H NMR (400 MHz, $CDCl_3$) δ 7.83 – 7.77 (m, 2H), 7.70 – 7.64 (m, 2H), 7.45 (d, *J* = 8.2 Hz, 2H), 7.36 (d, *J* = 8.1 Hz, 2H), 5.77 (s, 1H), 4.12 – 4.06 (m, 2H), 4.02 – 3.96 (m, 2H), 3.67 – 3.58 (m, 2H), 3.54 – 3.40 (m, 1H), 2.05 – 1.97 (m, 2H), 1.64 – 1.43 (m, 2H), 0.83 (s, 3H), 0.79 (s, 3H). ^{19}F NMR (376 MHz, $CDCl_3$) δ -70.1 (d, *J* = 9.8 Hz). ^{13}C NMR (101 MHz, $CDCl_3$) δ 168.1, 137.7, 137.3, 133.8, 132.0, 129.3, 126.9 (q, *J* = 281.3 Hz), 126.7, 123.0, 103.2, 65.2, 46.1 (q, J = 26.4 Hz), 39.9, 39.8, 33.8, 32.7, 27.3, 27.0. MS (DART): m/z (%) 462.2 ($[M+H]^+$, 100), 479.2 ($[M+NH_4]^+$). HRMS (DART): Calculated for $C_{25}H_{27}F_3NO_4$ ($[M+H]^+$): 462.1887; Found: 462.1882.

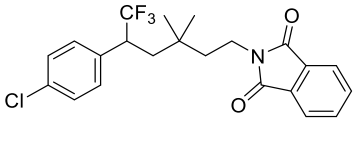

2-(5-(4-Chlorophenyl)-6,6,6-trifluoro-3,3-dimethylhexyl)isoindoline-1,3-dione (4n). The product (93 mg, 55% yield using **P1**) as a white solid (m.p. 75.2 – 76.6 °C) was purified with silica gel chromatography (Hexane/Ethyl Acetate = 15/1). ^{1}H NMR (400 MHz, $CDCl_3$) δ 7.83 – 7.76 (m, 2H), 7.70 – 7.63 (m, 2H), 7.31 – 7.26 (m, 4H), 3.69 – 3.54 (m, 2H), 3.52 – 3.38 (m, 1H), 2.05 – 1.87 (m, 2H), 1.63 – 1.42 (m, 2H), 0.84 (s, 3H), 0.79 (s, 3H). ^{19}F NMR (376 MHz, $CDCl_3$) δ -70.3 (d,

J = 9.7 Hz). ^{13}C NMR (101 MHz, $CDCl_3$) δ 168.1, 134.8, 133.9, 133.8, 132.0, 130.6, 128.8, 126.8 (q, J = 281.3 Hz), 123.0, 45.7 (q, J = 26.6 Hz), 39.8, 39.7, 33.7, 32.7, 27.3, 27.1. MS (DART): m/z (%) 424.1 ([M+H]$^+$), 441.2 ([M+NH_4]$^+$, 100). HRMS (DART): Calculated for $C_{22}H_{22}ClF_3NO_2$ ([M+H]$^+$): 424.1286; Found: 424.1283.

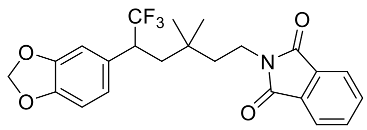

2-(5-(Benzo[d][1,3]dioxol-5-yl)-6,6,6-trifluoro-3,3-dimethylhexyl)isoindoline-1,3-dione (4o). The product (97 mg, 56% yield using **P2**) as a yellow oil was purified with silica gel chromatography (Hexane/Ethyl Acetate = 5/1). ^{1}H NMR (400 MHz, $CDCl_3$) δ 7.83 – 7.76 (m, 2H), 7.70 – 7.63 (m, 2H), 6.86 – 6.70 (m, 3H), 5.92 (s, 2H), 3.70 – 3.54 (m, 2H), 3.47 – 3.31 (m, 1H), 2.03 – 1.83 (m, 2H), 1.65 – 1.42 (m, 2H), 0.86 (s, 3H), 0.82 (s, 3H). ^{19}F NMR (376 MHz, $CDCl_3$) δ -70.5 (d, J = 9.7 Hz). ^{13}C NMR (101 MHz, $CDCl_3$) δ 1681, 147.8, 147.3, 133.8, 132.01, 129.8, 127.0 (q, J = 281.3 Hz), 123.0, 109.1, 108.2, 101.1, 45.9 (q, J = 26.5 Hz), 39.8, 39.7, 33.8, 32.7, 27.3, 27.1. MS (DART): m/z (%) 434.2 ([M+H]$^+$), 451.2 ([M+NH_4]$^+$, 100). HRMS (DART): Calculated for $C_{23}H_{23}F_3NO_4$ ([M+H]$^+$): 434.1574; Found: 434.1568.

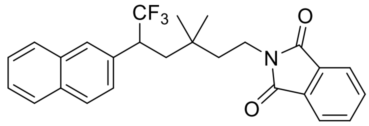

2-(6,6,6-Trifluoro-3,3-dimethyl-5-(naphthalen-2-yl)hexyl)isoindoline-1,3-dione (4p). The product (99 mg, 56% yield using **P1**) as a yellow oil was purified with silica gel chromatography (Hexane/Ethyl Acetate = 10/1). ^{1}H NMR (400 MHz, $CDCl_3$) δ 7.88 – 7.78 (m, 6H), 7.69 – 7.61 (m, 2H), 7.54 – 7.62 (m, 3H), 3.75 – 3.61 (m, 3H), 2.23 – 2.08 (m, 2H), 1.74 – 1.51 (m, 2H), 0.88 (s, 3H), 0.84 (s, 3H). ^{19}F NMR (376 MHz, $CDCl_3$) δ -69.8 (d, J = 9.8 Hz). ^{13}C NMR (101 MHz, $CDCl_3$) δ 168.1, 133.7, 133.1, 132.9, 132.0, 128.8, 128.3, 127.8, 127.5, 127.2 (q, J = 281.6 Hz), 126.6, 126.2, 126.1, 123.0, 46.4 (q, J = 26.4 Hz), 39.9, 39.8, 33.8, 32.8, 27.3, 27.0. MS (DART): m/z (%) 440.2 ([M+H]$^+$), 457.2 ([M+NH_4]$^+$, 100). HRMS (DART): Calculated for $C_{26}H_{25}F_3NO_2$ ([M+H]$^+$): 440.1832; Found: 440.1830.

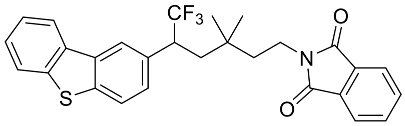

2-(5-(Dibenzo[b,d]thiophen-2-yl)-6,6,6-trifluoro-3,3-dimethylhexyl)isoindoline-1,3-dione (4q). The product (105 mg, 53% yield using **P1**) as a yellow viscous oil was purified with silica gel chromatography (Hexane/Ethyl Acetate = 10/1). ^{1}H NMR (400 MHz, $CDCl_3$) δ 8.23 – 8.13 (m, 2H), 7.86 – 7.79 (m, 2H), 7.79 – 7.73 (m, 2H), 7.66 – 7.56 (m, 2H), 7.53 – 7.39 (m, 3H), 3.78 – 3.59 (m, 3H), 2.17 (d, J = 5.8 Hz, 2H), 1.75 – 1.51 (m, 2H), 0.90 (s, 3H), 0.86 (s, 3H). ^{19}F NMR (376 MHz, $CDCl_3$) δ -69.9 (d, J = 9.7 Hz). ^{13}C NMR (101 MHz, $CDCl_3$) δ 168.0, 139.7, 139.1, 135.7, 135.0, 133.6, 132.7, 131.9, 127.7, 127.1 (q, J = 281.4 Hz), 126.8, 124.3, 122.9, 122.7, 122.3, 121.6, 46.3 (q, J = 26.5 Hz), 40.2, 39.8, 33.8, 32.8, 27.4, 27.1. MS (DART): m/z (%) 496.2 ([M+H]$^{+}$), 513.2 ([M+NH_4]$^{+}$, 100). HRMS (DART): Calculated for $C_{28}H_{25}F_3NO_2S$ ([M+H]$^{+}$): 496.1553; Found: 496.1549.

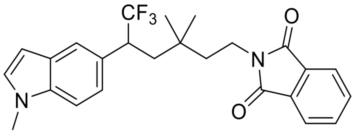

2-(6,6,6-Trifluoro-3,3-dimethyl-5-(1-methyl-1H-indol-5-yl)hexyl)isoindoline-1,3-dione (4r). The product (92 mg, 52% yield using **P2**) as a yellow solid (m.p. 92.0 – 93.2 °C) was purified with silica gel chromatography (Hexane/Ethyl Acetate = 5/1). ^{1}H NMR (400 MHz, $CDCl_3$) δ 7.86 – 7.79 (m, 2H), 7.71 – 7.62 (m, 3H), 7.33 – 7.19 (m, 2H), 7.03 (d, J = 3.0 Hz, 1H), 6.48 (d, J = 3.0 Hz, 1H), 3.73 (s, 3H), 3.69 (t, J = 8.3 Hz, 2H), 3.65 – 3.54 (m, 1H), 2.21 – 2.04 (m, 2H), 1.75 – 1.51 (m, 2H), 0.88 (s, 3H), 0.85 (s, 3H). ^{19}F NMR (376 MHz, $CDCl_3$) δ -70.2 (d, J = 9.9 Hz). ^{13}C NMR (101 MHz, $CDCl_3$) δ 168.0, 136.2, 133.7, 132.0, 129.3, 128.4, 127.6 (q, J = 281.4 Hz), 126.9, 122.9, 122.6, 121.7, 109.1, 100.8, 46.3 (q, J = 26.1 Hz), 40.2, 39.9, 33.9, 32.7, 32.6, 27.3, 26.9. MS (DART): m/z (%) 443.2 ([M+H]$^{+}$). HRMS (DART): Calculated for $C_{25}H_{26}F_3N_2O_2$ ([M+H]$^{+}$): 443.1941; Found: 443.1931.

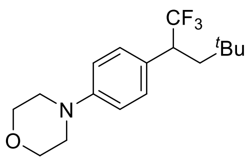

4-(4-(1,1,1-Trifluoro-4,4-dimethylpentan-2-yl)phenyl)morpholine (5a). The product (68 mg, 54% yield using **P2**) as a yellow oil was purified with silica gel chromatography (DCM). ^{1}H NMR (400 MHz, $CDCl_3$) δ 7.29 – 7.16 (m, 2H), 6.86 (d, J = 8.5 Hz, 2H), 3.90 – 3.77 (m, 4H), 3.33 – 3.19 (m, 1H), 3.18 – 3.10 (m, 4H), 1.91 – 1.83 (m, 2H), 0.80 (s, 9H). ^{19}F NMR (376 MHz, $CDCl_3$) δ -70.6 (d, J = 10.2 Hz). ^{13}C NMR (101 MHz, $CDCl_3$) δ 150.7, 130.0, 127.6, 127.4 (q, J = 281.1 Hz), 115.2, 66.8, 48.9, 46.0 (q, J

= 26.2 Hz), 41.8, 30.7, 29.7. MS (ESI): m/z (%) 316.2 ([M+H]$^+$). HRMS (ESI): Calculated for $C_{17}H_{25}F_3NO$ ([M+H]$^+$): 316.1883; Found: 316.1883.

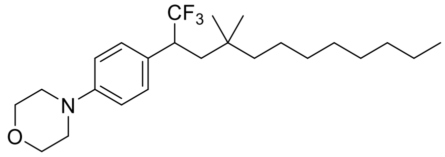

4-(4-(1,1,1-Trifluoro-4,4-dimethyldodecan-2-yl)phenyl)morpholine (5b). The product (65 mg, 39% yield using **P1** at 0 °C) as a yellow oil was purified with silica gel chromatography (DCM). ^{1}H NMR (400 MHz, $CDCl_3$) δ 7.22 (d, *J* = 8.4 Hz, 2H), 6.87 (d, *J* = 8.6 Hz, 2H), 3.89 – 3.82 (m, 4H), 3.30 – 3.19 (m, 1H), 3.19 – 3.12 (m, 4H), 1.93 – 1.80 (m, 2H), 1.31 – 1.02 (m, 14H), 0.89 (t, *J* = 6.9 Hz, 3H), 0.76 (s, 3H), 0.74 (s, 3H). ^{19}F NMR (376 MHz, $CDCl_3$) δ -70.6 (d, *J* = 10.2 Hz, 3F). ^{13}C NMR (101 MHz, $CDCl_3$) δ 150.7, 130.1, 127.8, 127.8, 127.5 (q, *J* = 281.0 Hz), 115.3, 66.8, 49.0, 45.7 (q, *J* = 26.3 Hz), 42.1, 39.7, 33.1, 31.9, 30.4, 29.6, 29.3, 27.7, 27.5, 23.8, 22.7, 14.1. MS (ESI): m/z (%) 414.3 ([M+H]$^+$). HRMS (ESI): Calculated for $C_{24}H_{39}F_3NO$ ([M+H]$^+$): 414.2978; Found: 414.2980.

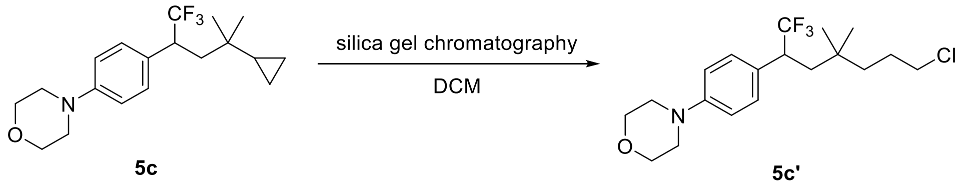

5c 5c'

4-(4-(7-Chloro-1,1,1-trifluoro-4,4-dimethylheptan-2-yl)phenyl)morpholine (5c'). The product (30 mg, 20% yield using **P1**) as a yellow oil was purified with silica gel chromatography (DCM). **5c'** was obtained from **5c** (reaction run with **2d** as alkyl iodide) during silica gel chromatography. **5c** can only be observed on the TLC. ^{1}H NMR (400 MHz, $CDCl_3$) δ 7.21 (d, *J* = 8.5 Hz, 2H), 6.87 (d, *J* = 8.6 Hz, 2H), 3.88 – 3.82 (m, 4H), 3.30 (t, *J* = 6.8 Hz, 2H), 3.27 – 3.20 (m, 1H), 3.19 – 3.13 (m, 4H), 1.95 – 1.79 (m, 2H), 1.71 – 1.59 (m, 1H), 1.59 – 1.46 (m, 1H), 1.32 – 1.21 (m, 1H), 1.20 – 1.10 (m, 1H), 0.79 (s, 3H), 0.78 (s, 3H). ^{19}F NMR (376 MHz, $CDCl_3$) δ -70.6 (d, *J* = 10.0 Hz, 3F). ^{13}C NMR (126 MHz, $CDCl_3$) δ 151.0, 130.1, 127.5, 127.5 (q, *J* = 280.0 Hz), 115.5, 66.9, 49.1, 45.7 (q, *J* = 26.5 Hz), 45.6, 39.7, 39.2, 33.1, 27.7, 27.5, 27.5. MS (ESI): m/z (%) 378.1 ([M+H]$^+$). HRMS (ESI): Calculated for $C_{19}H_{28}ClF_3NO$ ([M+H]$^+$): 378.1806; Found: 378.1804.

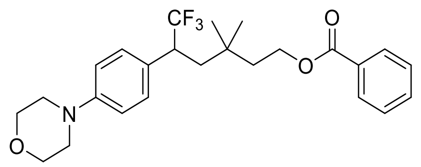

6,6,6-Trifluoro-3,3-dimethyl-5-(4-morpholinophenyl)hexyl benzoate (5d). The product (95 mg, 53% yield using **P2**) as a brown oil was purified with silica gel chromatography (Hexane/Ethyl Acetate = 10/1). ^{1}H NMR (400 MHz, $CDCl_3$) δ 8.0 (d, *J* = 7.5 Hz, 2H), 7.58 – 7.51 (m, 1H), 7.46 – 7.39 (m, 2H), 7.23 (d, *J* = 8.5 Hz, 2H), 6.85 (d, *J* = 8.6 Hz, 2H), 4.35 – 4.15 (m, 2H), 3.83 – 3.77 (m, 4H), 3.39 – 3.24 (m, 1H), 3.12 – 3.06 (m, 4H), 2.06 – 1.92 (m, 2H), 1.71 – 1.55 (m, 2H), 0.91 (s, 3H), 0.87 (s, 3H). ^{19}F NMR (376 MHz, $CDCl_3$) δ -70.5 (d, *J* = 9.8 Hz, 3F). ^{13}C NMR (101 MHz, $CDCl_3$) δ 166.3, 150.7, 132.7, 130.2, 129.9, 129.4, 128.2, 127.2 (q, *J* = 281.3 Hz), 127.0, 115.2, 66.6, 61.7, 48.6, 45.5 (q, *J* = 26.4 Hz), 40.2, 39.9, 32.6, 27.4, 27.3. MS (ESI): m/z (%) 450.2 ($[M+H]^+$). HRMS (ESI): Calculated for $C_{25}H_{31}F_3NO_3$ ($[M+H]^+$): 450.2251; Found: 450.2253.

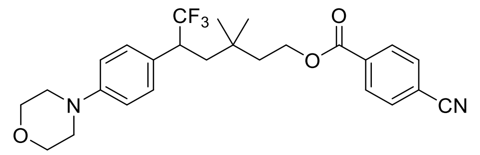

6,6,6-Trifluoro-3,3-dimethyl-5-(4-morpholinophenyl)hexyl 4-cyanobenzoate (5e). The product (118 mg, 62% yield using **P1**) as a yellow oil was purified with silica gel chromatography (Hexane/Ethyl Acetate = 5/1). ^{1}H NMR (400 MHz, $CDCl_3$) δ 8.04 (d, *J* = 8.2 Hz, 2H), 7.67 (d, *J* = 8.2 Hz, 2H), 7.20 (d, *J* = 8.4 Hz, 2H), 6.82 (d, *J* = 8.5 Hz, 2H), 4.34 – 4.25 (m, 1H), 4.24 – 4.15 (m, 1H), 3.83 – 3.71 (m, 4H), 3.36 – 3.21 (m, 1H), 3.12 – 2.99 (m, 4H), 2.01 – 1.88 (m, 2H), 1.69 – 1.52 (m, 2H), 0.87 (s, 3H), 0.83 (s, 3H). ^{19}F NMR (376 MHz, $CDCl_3$) δ -70.4 (d, *J* = 9.9 Hz, 3F). ^{13}C NMR (126 MHz, $CDCl_3$) δ 164.7, 150.8, 134.0, 132.1, 129.9, 127.2 (q, *J* = 279.8 Hz), 126.9, 66.6, 62.5, 48.6, 45.5 (q, *J* = 26.4 Hz), 40.2, 39.8, 32.6, 27.5, 27.3. MS (ESI): m/z (%) 475.1 ($[M+H]^+$). HRMS (ESI): Calculated for $C_{26}H_{30}F_3N_2O_3$ ($[M+H]^+$): 475.2203; Found: 475.2201.

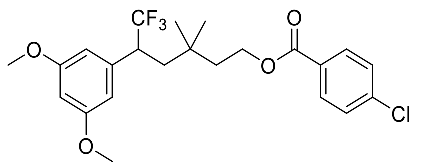

5-(3,5-Dimethoxyphenyl)-6,6,6-trifluoro-3,3-dimethylhexyl 4-chlorobenzoate (5f). The product (125 mg, 68% yield using **P2**) as a white solid (m.p. 71.8 – 73.0 °C) was purified with silica gel chromatography (Hexane/Ethyl Acetate = 10/1). ^{1}H NMR (400 MHz, $CDCl_3$) δ 7.91 (d, *J* = 8.3 Hz, 2H), 7.38 (d, *J* = 8.3 Hz, 2H), 6.50 (s, 2H), 6.37 (s, 1H), 4.36 – 4.14 (m, 2H), 3.74 (s, 6H), 3.38 – 3.24 (m, 1H), 2.07 – 1.90 (m, 2H), 1.71 – 1.56 (m, 2H), 0.91 (s, 3H), 0.88 (s,

3H). ^{19}F NMR (376 MHz, Chloroform-d) δ -70.0 – -70.1 (m, 3F). ^{13}C NMR (101 MHz, $CDCl_3$) δ 165.4, 160.7, 139.1, 138.4, 130.8, 128.7, 128.5, 127.0 (q, *J* = 281.4 Hz), 107.6, 99.3, 61.9, 55.0, 46.6 (q, *J* = 26.5 Hz), 40.3, 39.8, 32.6, 27.3, 27.2. MS (ESI): m/z (%) 459.1 ([M+H]$^+$). HRMS (ESI): Calculated for $C_{23}H_{27}ClF_3O_4$ ([M+H]$^+$): 459.1544; Found: 459.1549.

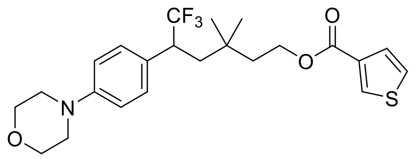

6,6,6-Trifluoro-3,3-dimethyl-5-(4-morpholinophenyl)hexyl thiophene-3-carboxylate (5g). The product (120 mg, 66% yield using **P2**) as a purple oil was purified with silica gel chromatography (Hexane/Ethyl Acetate = 10/1). ^{1}H NMR (400 MHz, $CDCl_3$) δ 8.05 – 8.02 (m, 1H), 7.51 – 7.45 (m, 1H), 7.28 – 7.24 (m, 1H), 7.22 (d, *J* = 8.5 Hz, 2H), 6.84 (d, *J* = 8.7 Hz, 2H), 4.30 – 4.09 (m, 2H), 3.83 – 3.77 (m, 4H), 3.38 – 3.22 (m, 1H), 3.12 – 3.06 (m, 4H), 2.04 – 1.89 (m, 2H), 1.67 – 1.49 (m, 2H), 0.88 (s, 3H), 0.84 (s, 3H). ^{19}F NMR (376 MHz, $CDCl_3$) δ -70.4 (d, *J* = 10.2 Hz). ^{13}C NMR (101 MHz, $CDCl_3$) δ 162.4, 150.7, 133.6, 132.3, 129.8, 127.7, 127.1 (q, *J* = 281.3 Hz) 126.9, 125.8, 115.1, 66.6, 61.3, 48.6, 45.5 (q, *J* = 26.5 Hz), 40.1, 39.8, 32.5, 27.4, 27.3. MS (ESI): m/z (%) 456.1 ([M+H]$^+$). HRMS (ESI): Calculated for $C_{23}H_{29}F_3NO_3S$ ([M+H]$^+$): 456.1815; Found: 456.1816.

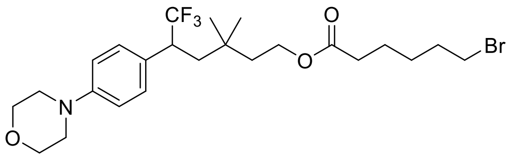

6,6,6-Trifluoro-3,3-dimethyl-5-(4-morpholinophenyl)hexyl 6-bromohexanoate (5h). The product (129 mg, 62% yield using **P2**) as a colorless oil was purified with silica gel chromatography (Hexane/Ethyl Acetate = 10/1). ^{1}H NMR (400 MHz, $CDCl_3$) δ 7.17 (d, *J* = 8.4 Hz, 2H), 6.83 (d, *J* = 8.5 Hz, 2H), 4.08 – 3.90 (m, 2H), 3.84 – 3.75 (m, 4H), 3.48 (t, *J* = 6.6 Hz, 2H), 3.31 – 3.18 (m, 1H), 3.15 – 3.06 (m, 4H), 2.23 (t, *J* = 7.4 Hz, 2H), 1.92 – 1.84 (m, 2H), 1.78 – 1.68 (m, 2H), 1.64 – 1.54 (m, 2H), 1.52 – 1.36 (m, 4H), 0.79 (s, 3H), 0.75 (s, 3H). ^{19}F NMR (376 MHz, $CDCl_3$) δ -70.5 (d, J = 9.9 Hz, 3F). ^{13}C NMR (101 MHz, $CDCl_3$) δ 173.1, 150.6, 129.8, 127.1 (q, J = 281.3 Hz), 126.9, 115.1, 66.6, 60.9, 48.6, 45.3 (q, *J* = 26.3 Hz), 44.5, 40.1, 39.8, 33.8, 32.4, 31.9, 27.3, 27.1, 26.1, 23.9. MS (ESI): m/z (%) 478.2 (100), 524.1 ([M+H]$^+$). HRMS (ESI): Calculated for $C_{24}H_{36}BrF_3NO_3$ ([M+H]$^+$): 522.1825; Found: 522.1829.

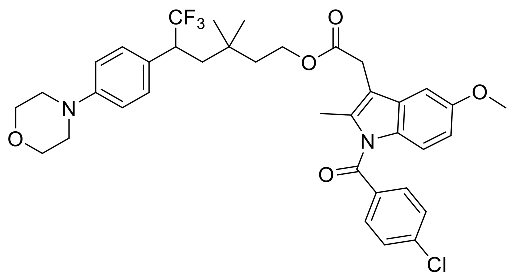

6,6,6-Trifluoro-3,3-dimethyl-5-(4-morpholinophenyl)hexyl 2-(1-(4-chlorobenzoyl)-5-methoxy-2-methyl-1H-indol-3-yl)acetate (5i). The product (113 mg, 41% yield using **P2**) as a yellow viscous oil was purified with silica gel chromatography (Hexane/Ethyl Acetate = 5/1). ^{1}H NMR (400 MHz, $CDCl_3$) δ 7.64 (d, *J* = 8.3 Hz, 2H), 7.44 (d, *J* = 8.3 Hz, 2H), 7.17 (d, *J* = 8.4 Hz, 2H), 6.97 (d, *J* = 2.0 Hz, 1H), 6.91 – 6.80 (m, 3H), 6.71 – 6.64 (m, 1H), 4.15 – 3.97 (m, 2H), 3.85 – 3.78 (m, 7H), 3.63 (s, 2H), 3.31 – 3.18 (m, 1H), 3.16 – 3.08 (m, 4H), 2.37 (s, 3H), 1.93 – 1.85 (m, 2H), 1.59 – 1.42 (m, J = 7.3 Hz, 2H), 0.79 (s, 3H), 0.76 (s, 3H). ^{19}F NMR (376 MHz, $CDCl_3$) δ -70.5 (d, *J* = 9.8 Hz). ^{13}C NMR (101 MHz, $CDCl_3$) δ 170.6, 168.0, 155.9, 150.7, 139.0, 135.7, 133.7, 131.0, 130.6, 130.5, 129.8, 128.9, 127.1 (q, *J* = 281.4 Hz), 126.9, 115.1, 114.8, 112.4, 111.4, 101.2, 66.6, 61.7, 55.4, 48.6, 45.4 (q, *J* = 26.5 Hz), 40.3, 39.8, 32.5, 30.2, 27.2, 27.1, 13.2. MS (ESI): m/z (%) 685.2 ([M+H]$^{+}$, 100). HRMS (ESI): Calculated for $C_{37}H_{41}ClF_3N_2O_5$ ([M+H]$^{+}$): 685.2651; Found: 685.2658.

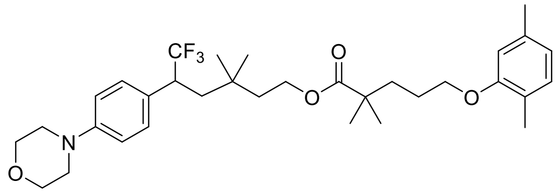

6,6,6-Trifluoro-3,3-dimethyl-5-(4-morpholinophenyl)hexyl 5-(2,5-dimethylphenoxy)-2,2-dimethylpentanoate (5j). The product (113 mg, 49% yield using **P1**) as a colorless viscous oil was purified with silica gel chromatography (Hexane/Ethyl Acetate = 5/1). ^{1}H NMR (400 MHz, $CDCl_3$) δ 7.23 (d, *J* = 8.4 Hz, 2H), 7.04 (d, *J* = 7.4 Hz, 1H), 6.87 (d, *J* = 8.6 Hz, 2H), 6.69 (d, *J* = 7.5 Hz, 1H), 6.64 (s, 1H), 4.14 – 3.97 (m, 2H), 3.97 – 3.91 (m, 2H), 3.89 – 3.83 (m, 4H), 3.38 – 3.23 (m, 1H), 3.20 – 3.12 (m, 4H), 2.34 (s, 3H), 2.21 (s, 3H), 1.99 – 1.91 (m, 2H), 1.78 – 1.69 (m, 4H), 1.57 – 1.44 (m, 2H), 1.22 (s, 6H), 0.89 (s, 3H), 0.82 (s, 3H). ^{19}F NMR (376 MHz, $CDCl_3$) δ -70.5 (d, *J* = 9.8 Hz). ^{13}C NMR (101 MHz, $CDCl_3$) δ 177.6, 156.8, 150.7, 136.3, 130.2, 129.9, 127.2 (q, *J* = 281.2 Hz), 127.1, 123.4, 120.6, 115.2, 111.8, 67.7, 66.7, 61.2, 48.7, 45.5 (q, *J* = 26.3 Hz), 41.8, 40.3, 40.2, 36.9, 32.6, 27.3, 27.2, 25.1, 25.0, 21.3, 15.7. MS (ESI): m/z (%) 578.3 ([M+H]$^{+}$, 100). HRMS (ESI): Calculated for $C_{33}H_{47}F_3NO_4$ ([M+H]$^{+}$): 578.3452; Found: 578.3440.

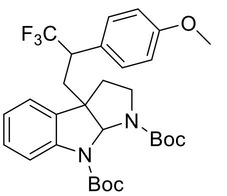

Di-*tert*-butyl 3a-(3,3,3-trifluoro-2-(4-methoxyphenyl)propyl)-2,3,3a,8a-tetrahydropyrrolo[2,3-b]indole-1,8-dicarboxylate (5k). The product (115 mg, 51% yield using **P2**) as a colorless viscous oil was purified with silica gel chromatography (Hexane/Ethyl Acetate = 10/1). ^{1}H NMR (400 MHz, $CDCl_3$) δ 7.28 – 7.19 (m, 2H), 7.12 – 7.01 (m, 2H), 6.91 (d, *J* = 8.4 Hz, 2H), 6.82 (d, *J* = 8.6 Hz, 2H), 3.81 – 3.70 (m, 4H), 2.98 – 2.84 (m, 1H), 2.68 – 2.51 (m, 2H), 2.42 – 2.27 (m, 1H), 2.00 – 1.92 (m, 1H), 1.84 – 1.74 (m, 1H), 1.58 – 1.53 (m, 2H), 1.48 (s, 2H), 1.43 (s, 15H). ^{19}F NMR (376 MHz, $CDCl_3$) δ -70.4 (d, *J* = 9.4 Hz). ^{13}C NMR (101 MHz, $CDCl_3$) δ 159.5, 153.9, 151.5, 143.7, 131.8, 130.0, 128.7, 126.8 (q, *J* = 281.7 Hz), 125.2, 123.0, 122.8, 114.7, 114.2, 81.0, 79.7, 79.1, 54.8, 54.7, 46.7 (q, *J* = 27.1 Hz), 44.5, 41.9, 37.0, 28.2, 28.1. MS (ESI): m/z (%) 451.1 (100), 563.2 ($[M+H]^+$). HRMS (ESI): Calculated for $C_{30}H_{38}F_3N_2O_5$ ($[M+H]^+$): 563.2727; Found: 563.2736.

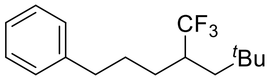

(6,6-Dimethyl-4-(trifluoromethyl)heptyl)benzene (5l). The reaction was conducted using $NiCl_2{\cdot}DME$ as the catalyst without **L3** and phosophine ligand. (***Note:*** the standard reaction conditions failed to provide the desired product). The product (28.3 mg, 26% yield) as a colorless viscous oil was purified with silica gel chromatography (Hexane). ^{1}H NMR (400 MHz, $CDCl_3$) δ 7.37 – 7.28 (m, 2H), 7.25 – 7.18 (m, 3H), 2.69 – 2.61 (m, 2H), 2.15 – 2.00 (m, 1H), 1.90 – 1.66 (m, 3H), 1.65 – 1.58 (m, 1H), 1.58 – 1.46 (m, 1H), 1.26 – 1.17 (m, 1H), 0.93 (s, 9H). ^{19}F NMR (376 MHz, $CDCl_3$) δ -70.2 (d, *J* = 10.0 Hz, 3F). ^{13}C NMR (126 MHz, $CDCl_3$) δ 141.9, 129.0 (q, *J* = 279.8 Hz), 128.4, 125.9, 41.8 (d, *J* = 1.9 Hz), 39.2 (q, *J* = 24.9 Hz), 36.1, 30.9 (q, *J* = 2.2 Hz), 30.7, 29.4, 28.9. MS (FI): m/z (%) 272 ($[M]^+$). HRMS (FI): Calculated for $C_{16}H_{23}F_3$ ($[M]^+$): 272.1750; Found: 272.1746.

7. Transformations of Compound 4a

Deprotection of compound 4a

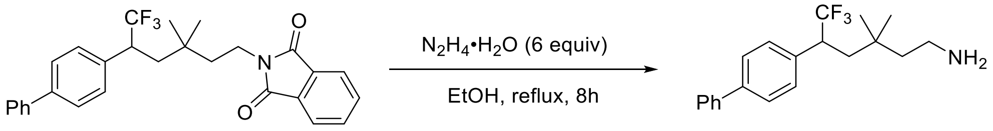

4a **8**

5-([1,1'-Biphenyl]-4-yl)-6,6,6-trifluoro-3,3-dimethylhexan-1-amine (8). To a suspension of **4a** (6 mmol, 1 equiv) in 40 mL ethanol was added hydrazine monohydrate (2 mL, 18 mmol, 6 equiv) at room temperature. The mixture was heated under reflux for 8 hours. The reaction was cooled to room temperature, and the resulting precipitate was filtered off and washed by ethyl acetate. The filtrate was concentrated and the residue was purified with silica gel chromatography (DCM/MeOH = 30/1) to give compound **8** as a yellow solid (1.44g, 72% yield, m.p. 77.0 – 78.0 °C). ^{1}H NMR (400 MHz, $CDCl_3$) δ 7.69 – 7.52 (m, 4H), 7.50 – 7.30 (m, 5H), 5.01 (s, 2H), 3.52 – 3.36 (m, 1H), 2.91 – 2.62 (m, 2H), 2.05 – 1.89 (m, 2H), 1.67 – 1.37 (m, 2H), 0.82 (s, 3H), 0.79 (s, 3H). ^{19}F NMR (376 MHz, $CDCl_3$) δ -70.0 (d, J = 9.5 Hz). ^{13}C NMR (126 MHz, $CDCl_3$) δ 141.0, 140.4, 129.8, 128.9, 127.5, 127.3, 127.3 (q, J = 279.9 Hz), 127.1, 46.1 (q, J = 26.7 Hz), 43.0, 40.1, 36.8, 32.9, 27.6, 27.2. MS (ESI): m/z (%) 336.1 ($[M+H]^+$). HRMS (ESI): Calculated for $C_{20}H_{25}F_3N$ ($[M+H]^+$): 336.1934; Found: 336.1932.

Synthesis of compound 10a

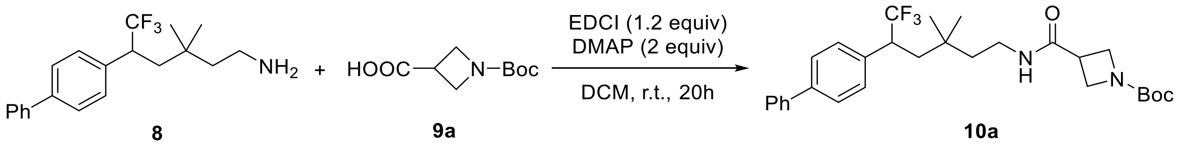

8 **9a** **10a**

***tert*-Butyl 3-((5-([1,1'-biphenyl]-4-yl)-6,6,6-trifluoro-3,3-dimethylhexyl)carbamoyl)azetidine-1-carboxylate (10a).** To a mixture of 1-(*t*-butyloxycarbonyl)-azetidine-3-carboxylic acid (1.2 mmol, 1.2 equiv) and 4-(dimethylamino)pyridine (2 mmol, 2 equiv) in 5 mL anhydrous CH_2Cl_2 was added *N*-(3-dimethylaminopropyl)-*N'*-ethylcarbodiimide hydrochloride (EDCI·HCl) (1.2 mmol, 1.2 equiv) and amine **6** (1 mmol, 1 equiv) at room temperature. The mixture was then stirred at room temperature for 20 h. The solution was diluted with ethyl acetate and washed with water and brine. The organic layer was dried over anhydrous Na_2SO_4, filtered, and concentrated. The residue was purified with silica gel chromatography (Hexane/Ethyl Acetate = 2/1) to give compound **10a** as a viscous colorless oil (451

mg, 87% yield). ^{1}H NMR (400 MHz, $CDCl_3$) δ 7.60 – 7.50 (m, 4H), 7.44 – 7.27 (m, 5H), 6.20 (s, 1H), 4.10 – 3.98 (m, 2H), 3.97 – 3.84 (m, 2H), 3.46 – 3.32 (m, 1H), 3.30 – 3.16 (m, 1H), 3.12 – 2.98 (m, 2H), 2.02 – 1.88 (m, 2H), 1.40 (s, 9H), 1.34 – 1.23 (m, 2H), 0.80 (s, 3H), 0.77 (s, 3H). ^{19}F NMR (376 MHz, $CDCl_3$) δ -70.0 (d, J = 9.7 Hz). ^{13}C NMR (126 MHz, $CDCl_3$) δ 171.6, 156.1, 140.7, 140.1, 135.4, 129.7, 128.8, 127.5, 127.1, 127.1 (q, J = 280.0 Hz), 126.8, 79.6, 52.1 (br), 46.0 (q, J = 26.3 Hz), 41.1, 40.0, 35.6, 33.0, 32.7, 28.2, 27.4, 27.0. MS (ESI): m/z (%) 541.2 ([M+Na]$^+$, 100). HRMS (ESI): Calculated for $C_{29}H_{37}F_3N_2NaO_3$ ([M+Na]$^+$): 541.2648; Found: 541.2646.

Synthesis of compound 10b

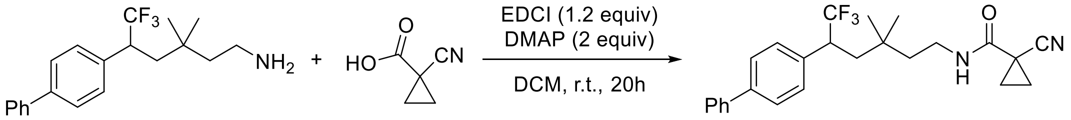

8 **9b** **10b**

***N*-(5-([1,1'-biphenyl]-4-yl)-6,6,6-trifluoro-3,3-dimethylhexyl)-1-cyanocyclopropane-1-carboxamide (10b).** To a mixture of 1-cyano-cyclopropanecarboxylic acid (1.2 mmol, 1.2 equiv) and 4-(dimethylamino)pyridine (2 mmol, 2 equiv) in 5 mL anhydrous CH_2Cl_2 was added *N*-(3-dimethylaminopropyl)-*N'*-ethylcarbodiimide hydrochloride (EDCI·HCl) (1.2 mmol, 1.2 equiv) and amine **6** (1 mmol, 1 equiv) at room temperature. The mixture then was stirred at room temperature for 20 h. The solution was diluted with ethyl acetate and washed with water and brine. The organic layer was dried over anhydrous Na_2SO_4, filtered, and concentrated. The residue was purified with silica gel chromatography (Hexane/Ethyl Acetate = 2/1) to give compound **10b** as a white solid (293 mg, 68% yield, m.p. 96.7 – 97.8 °C). ^{1}H NMR (400 MHz, $CDCl_3$) δ 7.66 – 7.58 (m, 4H), 7.48 – 7.32 (m, 5H), 6.37 (t, J = 5.0 Hz, 1H), 3.51 – 3.37 (m, 1H), 3.33 – 3.21 (m, 1H), 3.18 – 3.06 (m, 1H), 2.08 – 1.94 (m, 2H), 1.68 – 1.55 (m, 2H), 1.49 – 1.32 (m, 4H), 0.85 (s, 6H). ^{19}F NMR (376 MHz, $CDCl_3$) δ -70.0 (d, J = 9.8 Hz). ^{13}C NMR (126 MHz, $CDCl_3$) δ 165.0, 140.9, 140.2, 135.3, 129.7, 128.8, 127.5, 127.2, 127.2 (q, J = 279.6 Hz), 127.0, 120.2, 46.1 (q, J = 26.1 Hz), 40.8, 39.9, 36.5, 32.8, 27.5, 27.2, 17.3, 17.3, 13.4. MS (ESI): m/z (%) 429.2 ([M+H]$^+$, 100), 451.1 ([M+Na]$^+$). HRMS (ESI): Calculated for $C_{25}H_{28}F_3N_2O$ ([M+H]$^+$): 429.2148; Found: 429.2148.

Synthesis of compound 10c

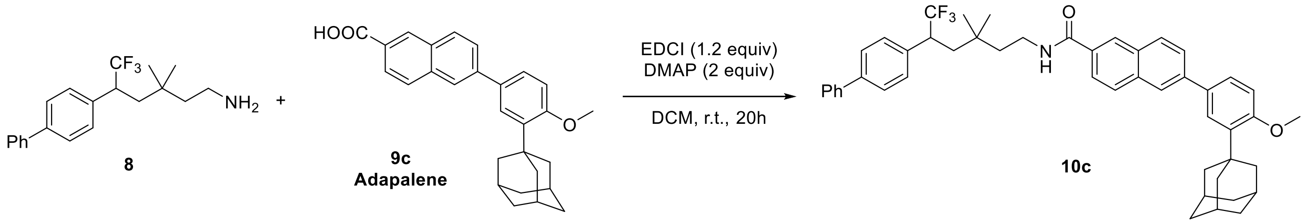

***N*-(5-([1,1'-biphenyl]-4-yl)-6,6,6-trifluoro-3,3-dimethylhexyl)-6-(3-((3*r*,5*r*,7*r*)-adamantan-1-yl)-4-methoxyphenyl)-2-naphthamide (10c).** To a mixture of adapalene (1.2 mmol, 1.2 equiv) and 4-(dimethylamino)pyridine (2 mmol, 2 equiv) in 5 mL anhydrous CH_2Cl_2 was added *N*-(3-dimethylaminopropyl)-*N'*-ethylcarbodiimide hydrochloride (EDCI·HCl) (1.2 mmol, 1.2 equiv) and amine **6** (1 mmol, 1 equiv) at room temperature. The mixture then was stirred at room temperature for 20 h. The solution was diluted with ethyl acetate and washed with water and brine. The organic layer was dried over anhydrous Na_2SO_4, filtered, and concentrated. The residue was purified with silica gel chromatography (Hexane/Ethyl Acetate = 5/1) to give compound **10c** as a white solid (673 mg, 92% yield, m.p. 141.5 – 142.8 °C). ^{1}H NMR (400 MHz, $CDCl_3$) δ 8.24 (s, 1H), 8.02 (s, 1H), 7.82 (s, 2H), 7.78 (s, 2H), 7.71 – 7.65 (m, 1H), 7.62 – 7.52 (m, 5H), 7.47 – 7.32 (m, 5H), 7.01 (d, *J* = 8.5 Hz, 1H), 6.45 (t, *J* = 5.2 Hz, 1H), 3.91 (s, 3H), 3.64 – 3.43 (m, 2H), 3.31 – 3.18 (m, 1H), 2.28 (s, 6H), 2.19 (s, 3H), 1.90 (s, 6H), 1.68 – 1.56 (m, 1H), 1.49 – 1.28 (m, 2H), 0.93 (s, 6H). ^{19}F NMR (376 MHz, $CDCl_3$) δ -69.8 (d, *J* = 9.8 Hz). ^{13}C NMR (126 MHz, $CDCl_3$) δ 167.6, 158.8, 140.9, 140.6, 140.0, 138.9, 135.4, 135.0, 132.5, 131.3, 131.3, 129.9, 129.3, 128.8, 128.4, 127.5, 127.2 (q, *J* = 279.9 Hz), 127.2, 127.1, 126.9, 126.4, 125.8, 125.7, 124.6, 123.9, 112.1, 55.1, 46.3 (q, *J* = 26.7 Hz), 40.9, 40.6, 39.7, 37.2, 37.1, 36.1, 34.1, 32.9, 29.1, 27.6, 27.4, 22.4, 14.1. MS (ESI): m/z (%) 730.3 ($[M+H]^+$, 100). HRMS (ESI): Calculated for $C_{48}H_{51}F_3NO_2$ ($[M+H]^+$): 730.3866; Found: 730.3867.

8. Mechanistic Studies

Radical Inhibition Experiments

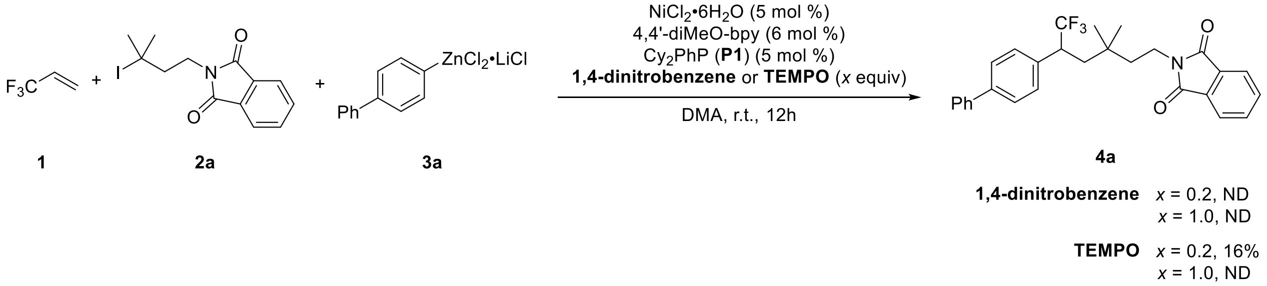

Procedure: To a 25 mL Schlenk tube were added **2a** (0.4 mmol, 1.0 equiv) 4,4'-diMeO-bpy (6 mol%), Cy_2PhP (5 mol%), 1,4-dinitrobenzene or TEMPO (0.2 equiv or 1.0 equiv) and $NiCl_2 \cdot 6H_2O$ (5 mol%) in the glovebox. Then **1** (1.6 mmol, 2.0 equiv, 2 mL) was added under Ar. The resulting mixture was stirred for 20 min at room temperature, and arylzinc reagent **3a** (0.6 mmol, 1.5 equiv) was added slowly within a period of 10 min, and the tube was sealed with a Teflon cap. After stirring for 12 h, the reaction mixture was quenched with aqueous NH_4Cl solution and diluted with EtOAc. The yield was determined by ^{19}F-NMR with benzotrifluoride as the internal standard. When 0.2 equiv or 1.0 equiv of 1,4-dinitrobenzene was added, the product **4a** was not detected. When 0.2 equiv of TEMPO was added, the product **4a** was obtained in 16% yield; when 1.0 equiv TEMPO was added, the product **4a** was not detected. In the case of using 1.0 equiv of TEMPO as the radical trapping reagent, compound 11 was detected by LC-MS. Compound **11**: MS (ESI): m/z (%) 373 ($[M+H]^+$).

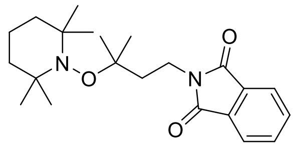

11, detected by LC-MS, $[M+H]^+$ = 373

Supplementary Figure 3 LC-MS of TEMPO Trapping Product **11**

Synthesis of aryl nickel complex B1

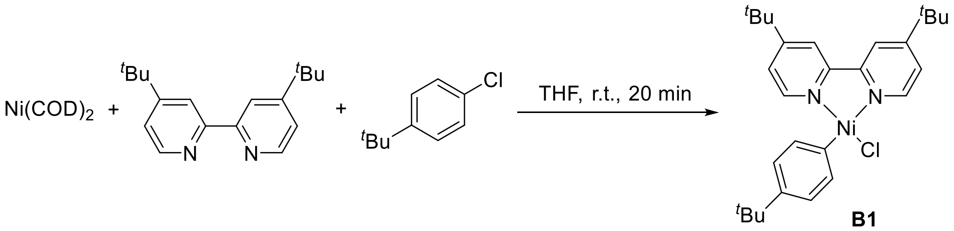

Procedure: Complex **B1** is a known compound and the preparation is according to the literature.[12] In a argon filled glove box, a 50 mL round bottom flask containing a stirring bar was charged with $Ni(COD)_2$ (550.0 mg, 2.0 mmol, 1.0 equiv), 4,4′-di-*tert*-butyl-2,2′-pyridine **L2** (537.0 mg, 2.0 mmol, 1.0 equiv) and dry THF (5 mL). The resulting dark purple mixture was stirred for 1h at room temperature. Then 1-chloro-4-(1,1-dimethylethyl)benzene (8.6 mL, 51.5 mmol, 25.0 equiv) was added and the reaction mixture was stirred for additional 20 minutes. The resulting dark red solution was triturated with pentane and the precipitate was collected on a frit, rinsed with pentane and dried under vacuum to give nickel complex (**B1**) as a light red powder (725 mg, 73% yield). The nickel complex was used in control experiments without further purification.

Control Experiments:

Stoichiometric Experiments

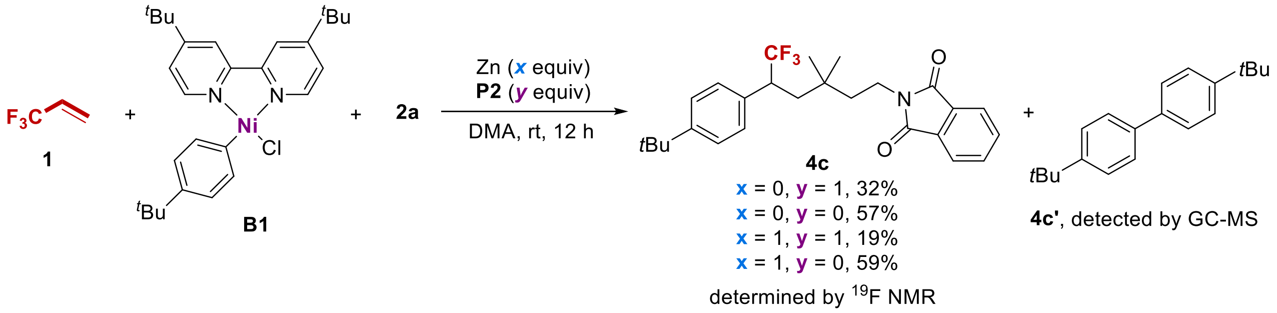

Procedure: To a 25 mL of Schlenck tube were added complex **B1** (0.2 mmol, 1.0 equiv), zinc powder (if needed, 0.2 mmol, 1.0 equiv) and Cy_2PhP (if needed, 0.2 mmol, 1.0 equiv). The tube was evacuated and backfilled with argon for 3 times, then anhydrous DMA was added (5 mL). The reaction mixture was stirred for 10 min. Then TFP solution (1 M in DMA, 5 mL) was added under Ar. The resulting mixture was stirred for additional 10 min at room temperature, and alkyl iodide **2a** (0.2 mmol, 1.0 equiv) was added in one portion, and the tube was sealed with a Teflon cap. After stirring for 12 h at room temperature, the reaction mixture was quenched with aqueous NH_4Cl solution and diluted with EtOAc. The yield was determined by ^{19}F-NMR with fluorobenzene as the internal standard. For all the control experiments, the biaryl **4c'** was detected by GC-MS. Compound **4c'**: MS (EI): m/z (%) 266 ($[M]^+$).

Complex B1 Catalyzed Reaction under Standard Reaction Conditions

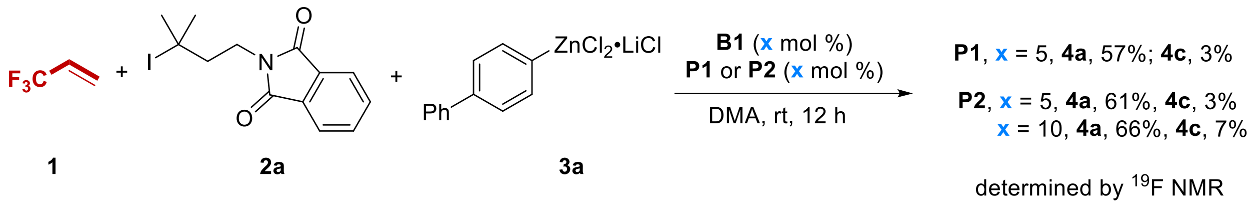

Procedure: To a 25 mL of Schlenck tube were added complex **B1** (x mol%) and the monodentate phosphine ligand (PCy_2Ph (**P1**) or $P^tBu_2Me{\cdot}HBF_4$ (**P2**), x mol%). The tube was evacuated and backfilled with argon for 3 times, then tertiary alkyl iodide **2a** (0.4 mmol, 1.0 equiv) and TFP solution (1 M in DMA, 1.6 mmol, 4.0 equiv, 2 mL) were added under Ar. The resulting mixture was stirred for 20 min at room temperature, and the corresponding arylzinc reagent **3a** (0.6 mmol, 1.5 equiv) was added slowly within a period of 10 min, and the tube was sealed with Teflon cap. After stirring for 12 h at room temperature, the reaction mixture was quenched with aqueous NH_4Cl solution and diluted with EtOAc. The yield was determined by ^{19}F-NMR using benzotrifluoride as the internal standard. For using **P1** as the co-ligand, **4a** was provided in 57% yield (x = 5) and **4c** was formed in 3% yield (x = 5); for using **P2** as the co-ligand, **4a** was provided in 61% yield (x =5) and 66% yield (x = 10), respectively; **4c** was formed in 3% yield (x = 5) and 7% yield (x = 10), respectively. The structure of **4c** was confirmed by its ^{19}F, ^{1}H, and ^{13}C NMR.

Supplementary Note

Supplementary Note 1: Characterization Spectra for Tertiary Alkyl Iodides 2

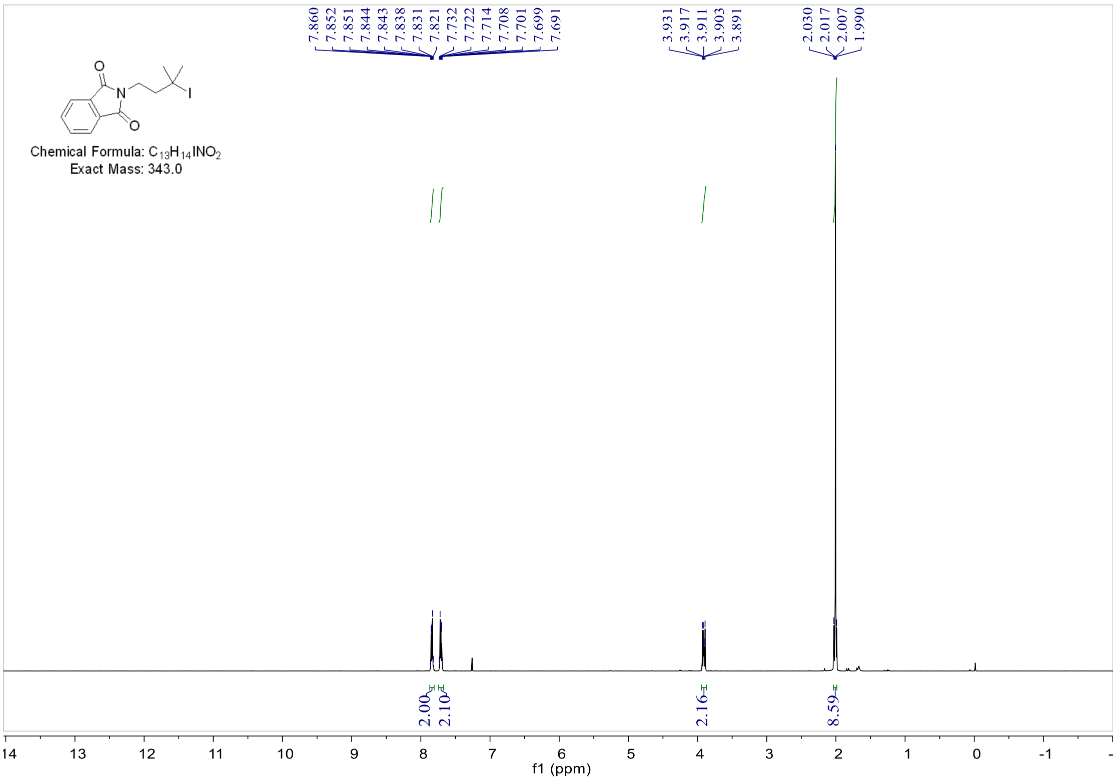

Supplementary Figure 4 ^{1}H NMR Spectrum of **2a**

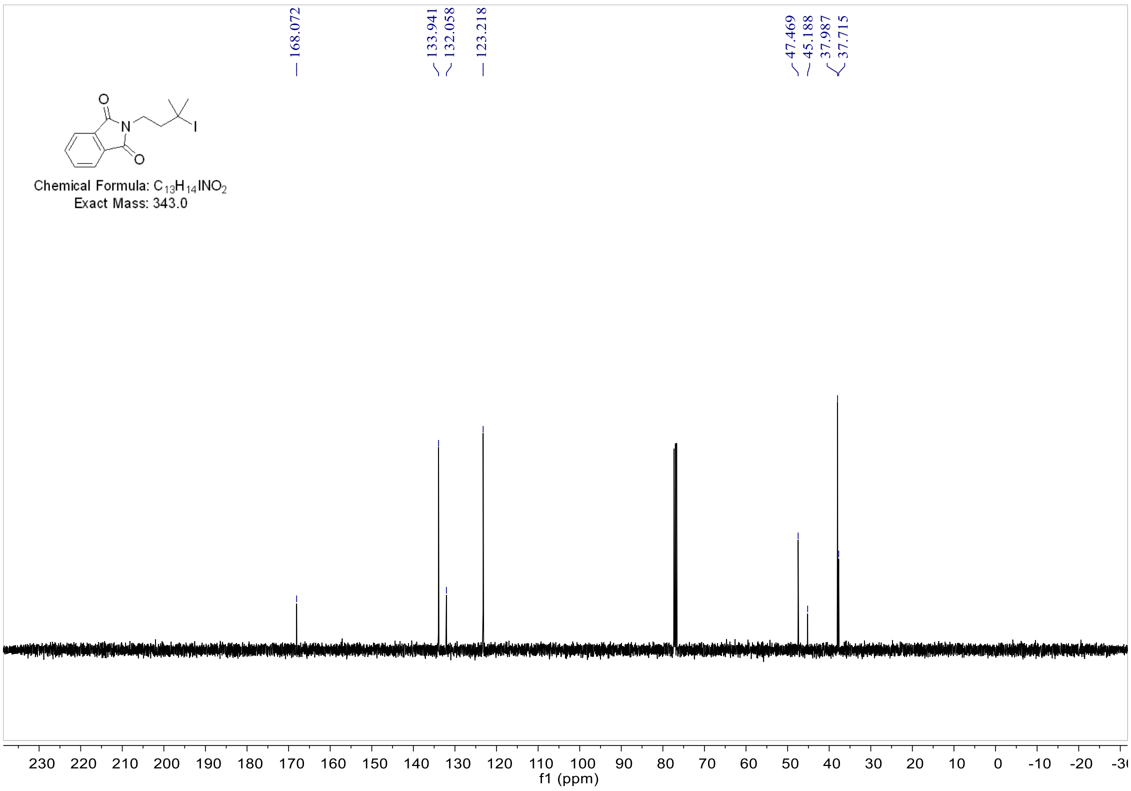

Supplementary Figure 5 ^{13}C NMR Spectrum of **2a**

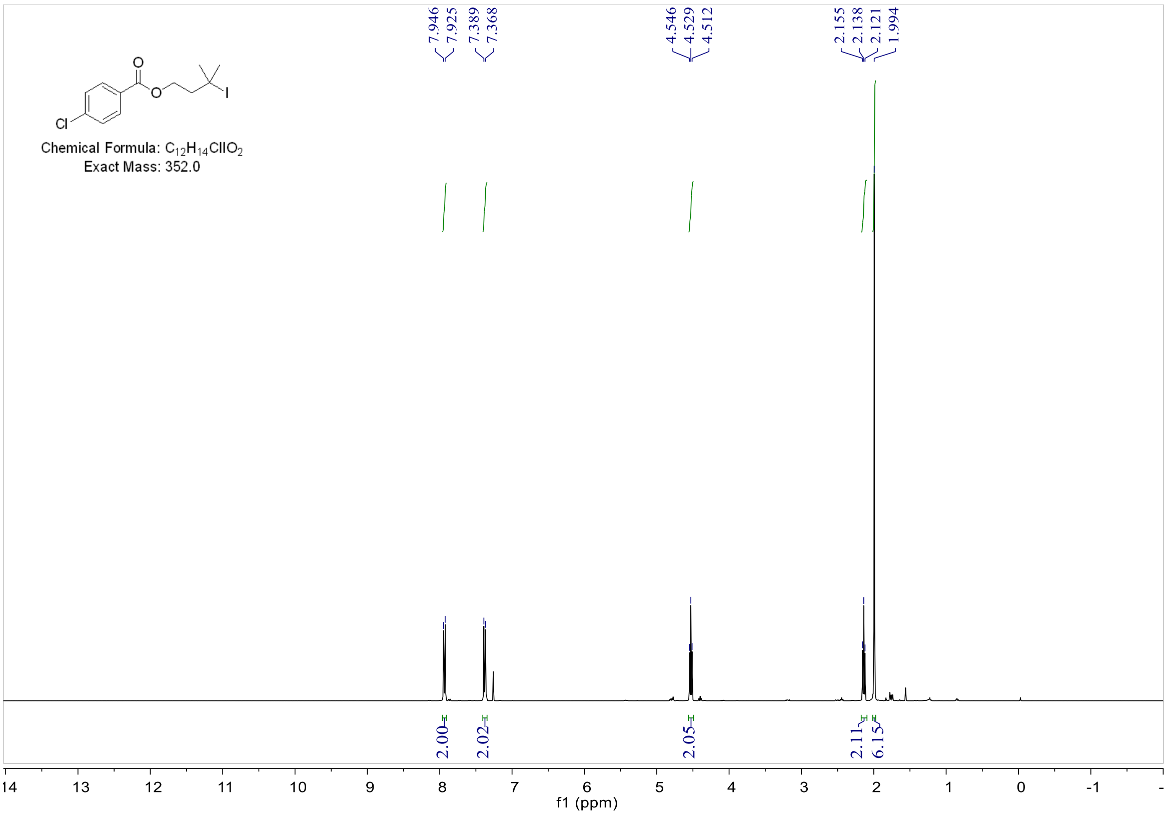

Supplementary Figure 6 ^{1}H NMR Spectrum of **2f**

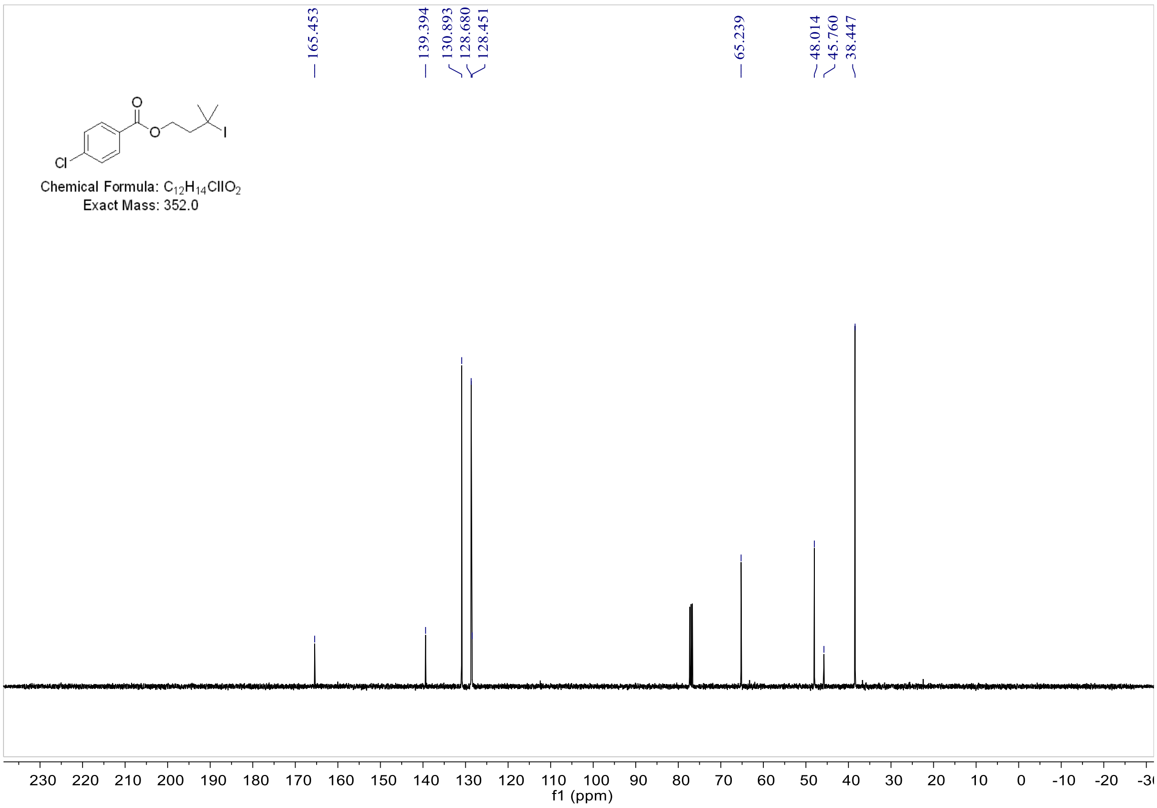

Supplementary Figure 7 ^{13}C NMR Spectrum of **2f**

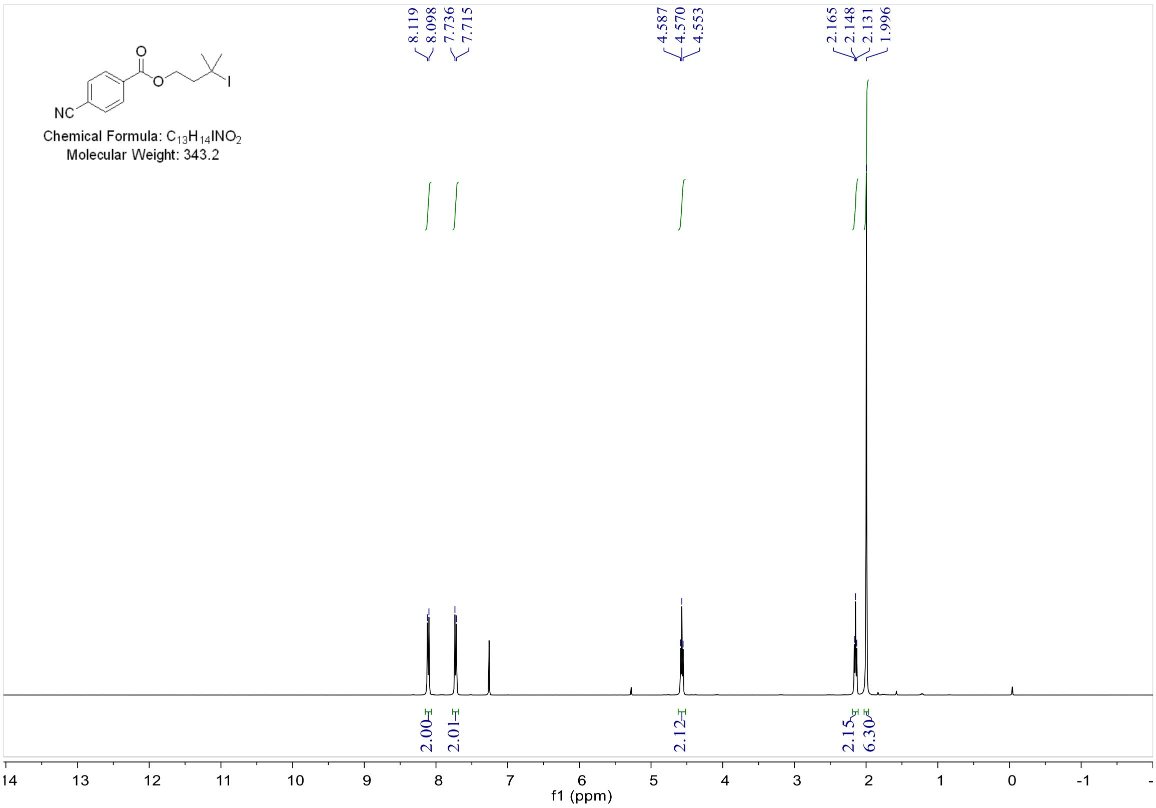

Supplementary Figure 8 ^{1}H NMR Spectrum of **2g**

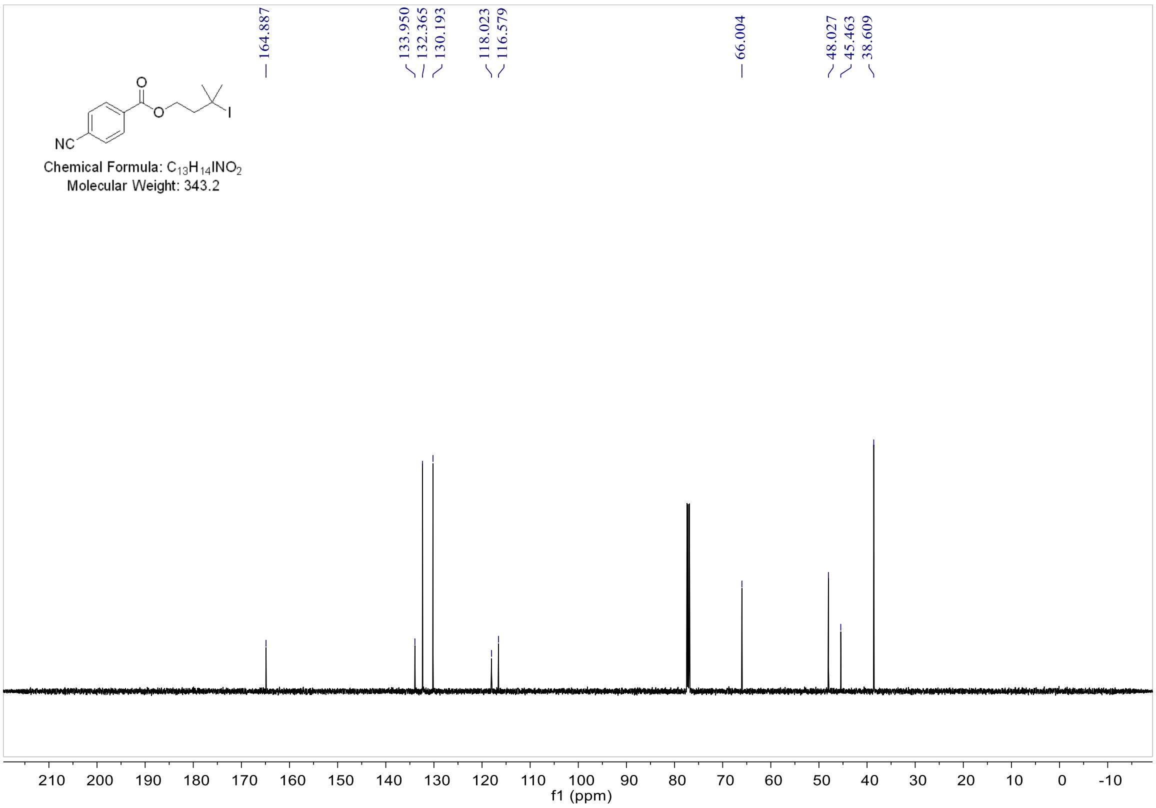

Supplementary Figure 9 ^{13}C NMR Spectrum of **2g**

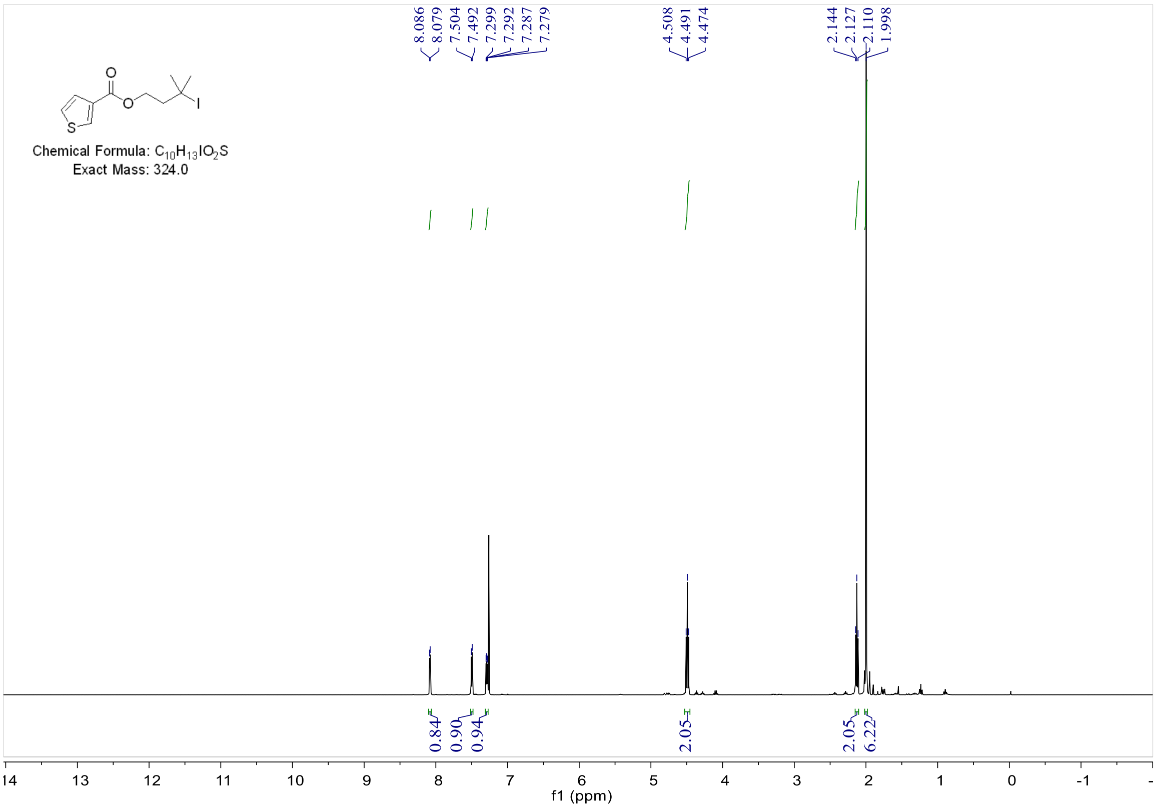

Supplementary Figure 10 ^{1}H NMR Spectrum of **2h**

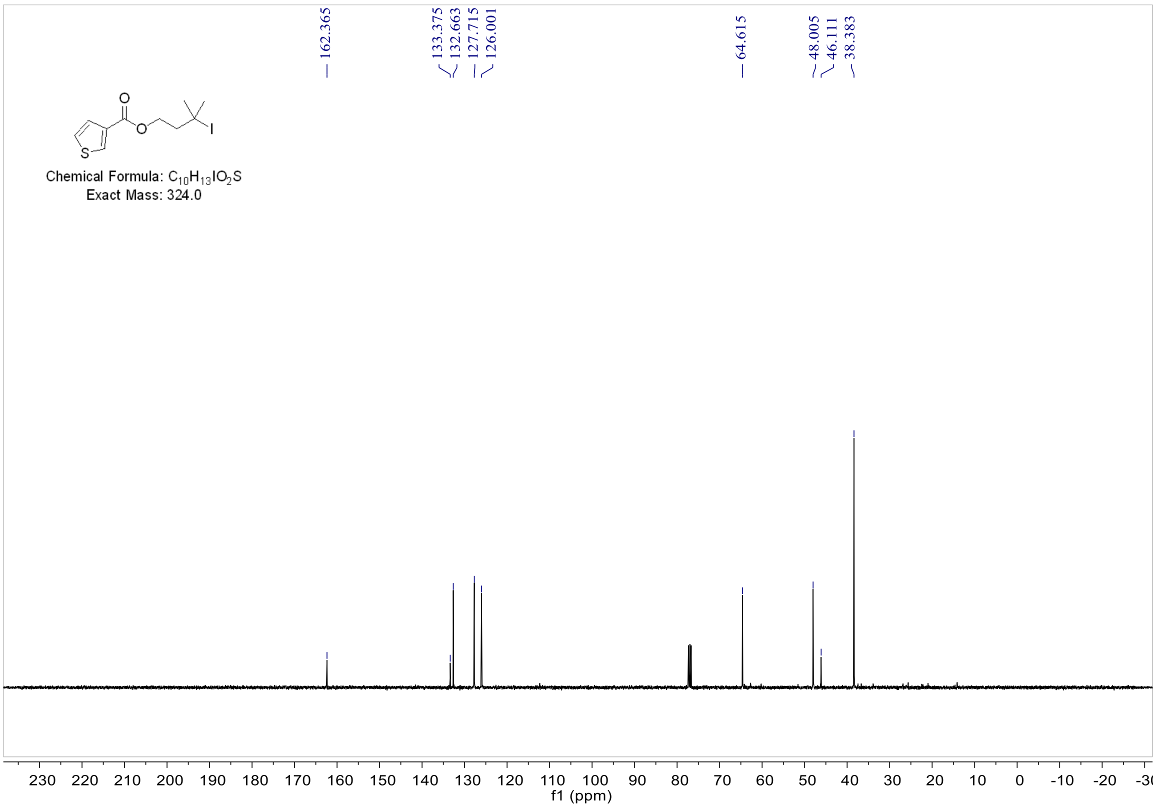

Supplementary Figure 11 ^{13}C NMR Spectrum of **2h**

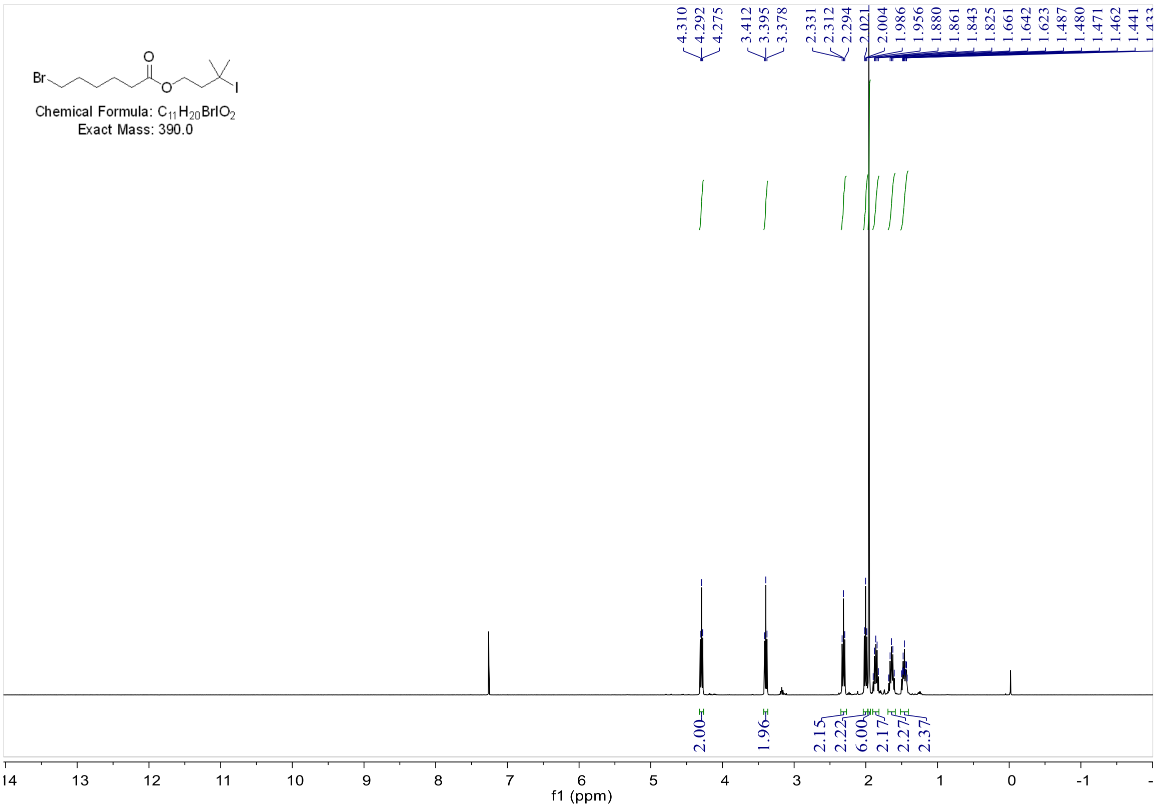

Supplementary Figure 12 ^{1}H NMR Spectrum of **2i**

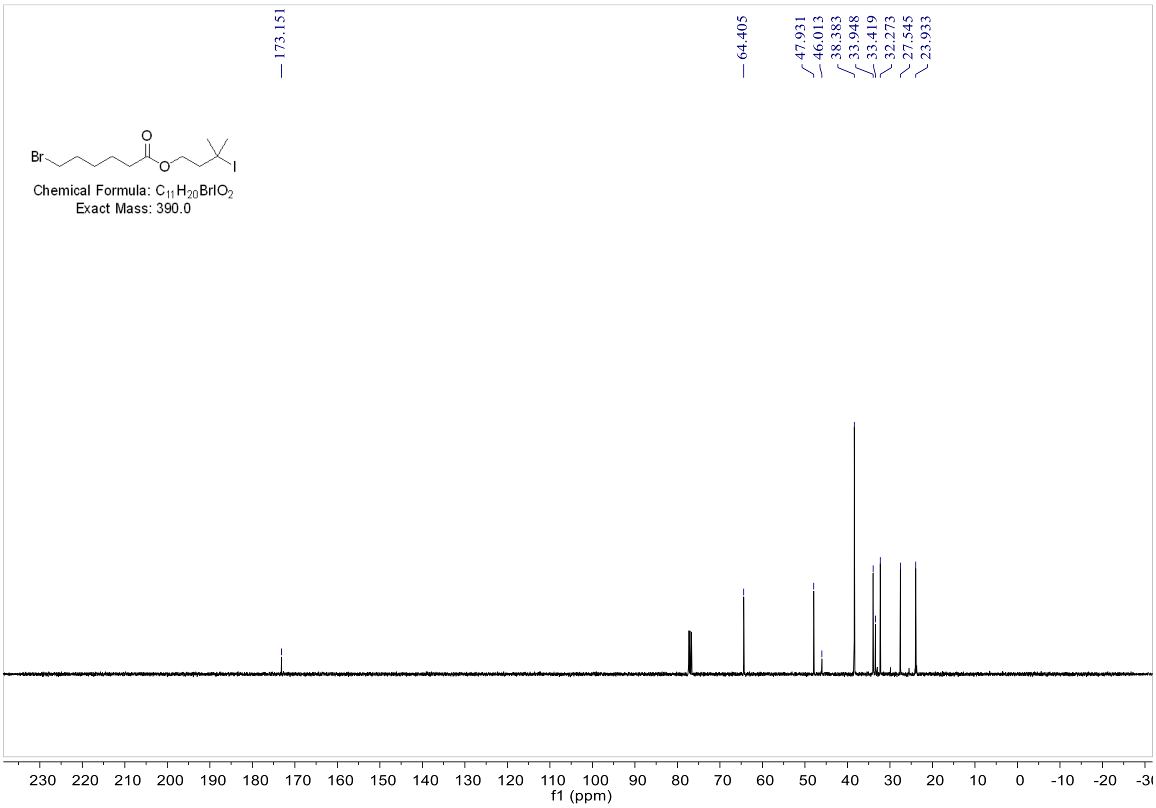

Supplementary Figure 13 ^{13}C NMR Spectrum of **2i**

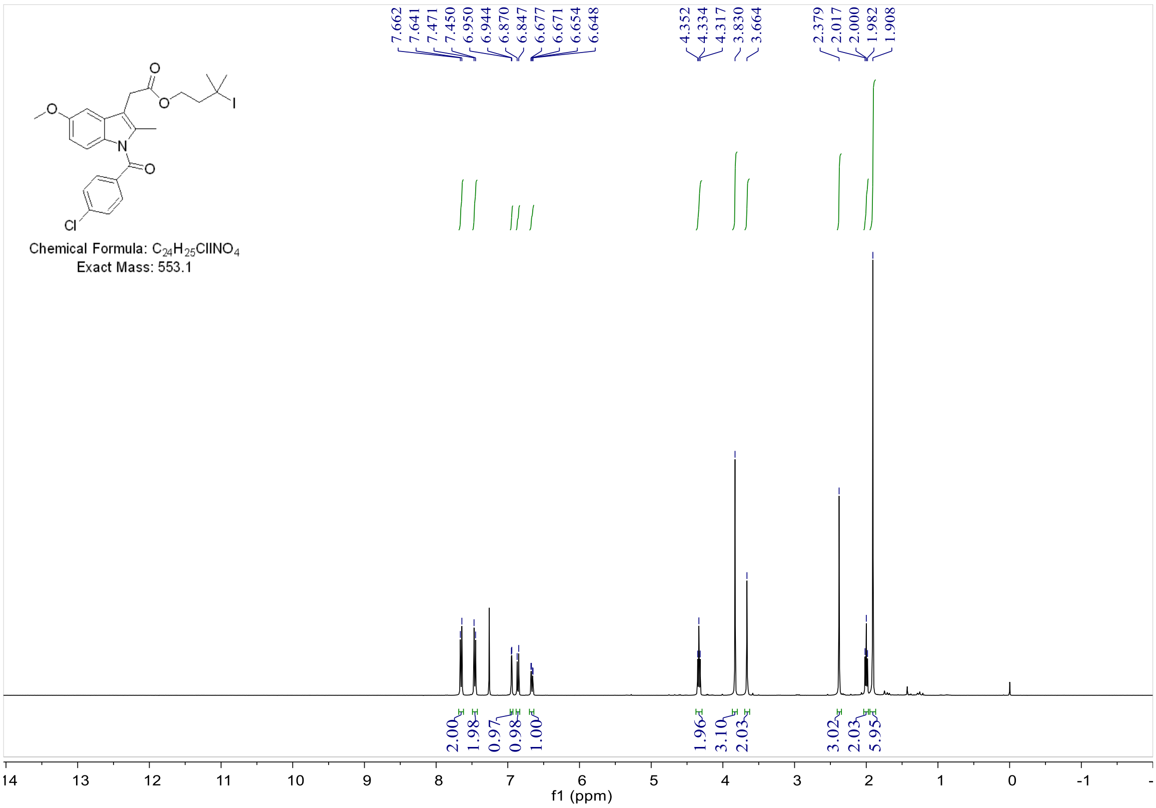

Supplementary Figure 14 ^{1}H NMR Spectrum of **2j**

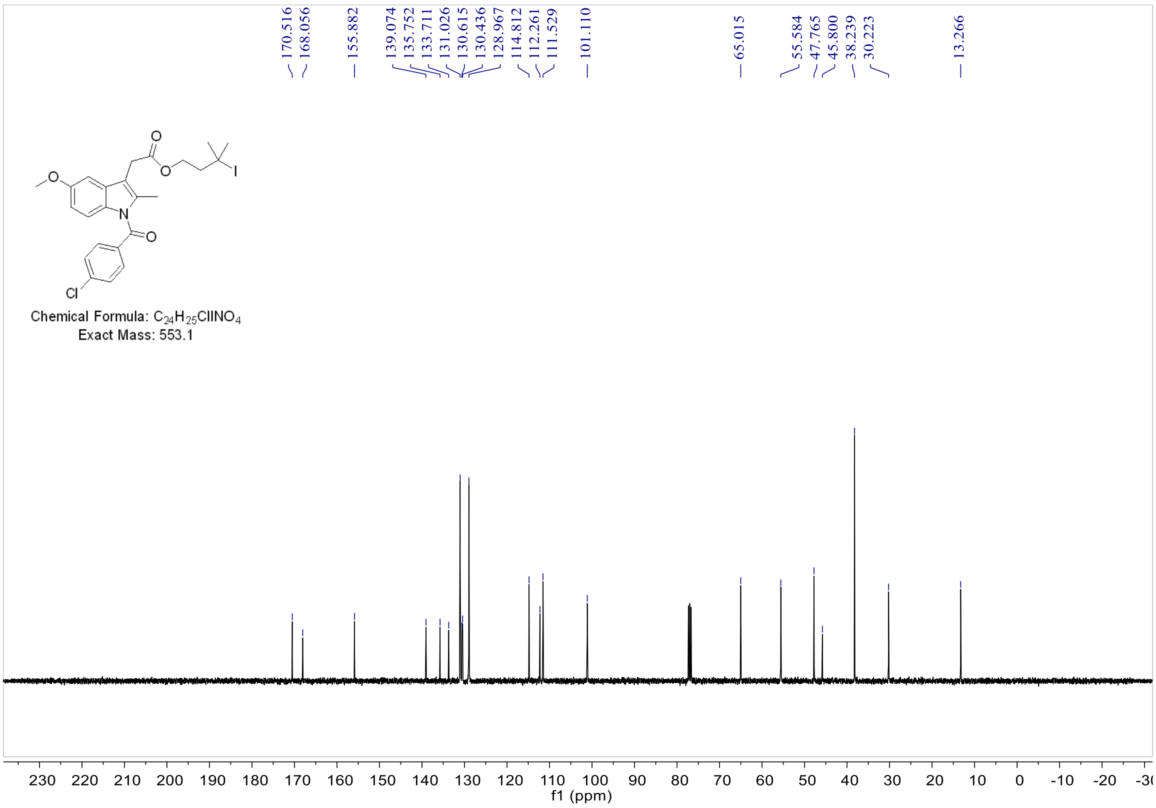

Supplementary Figure 15 ^{13}C NMR Spectrum of **2j**

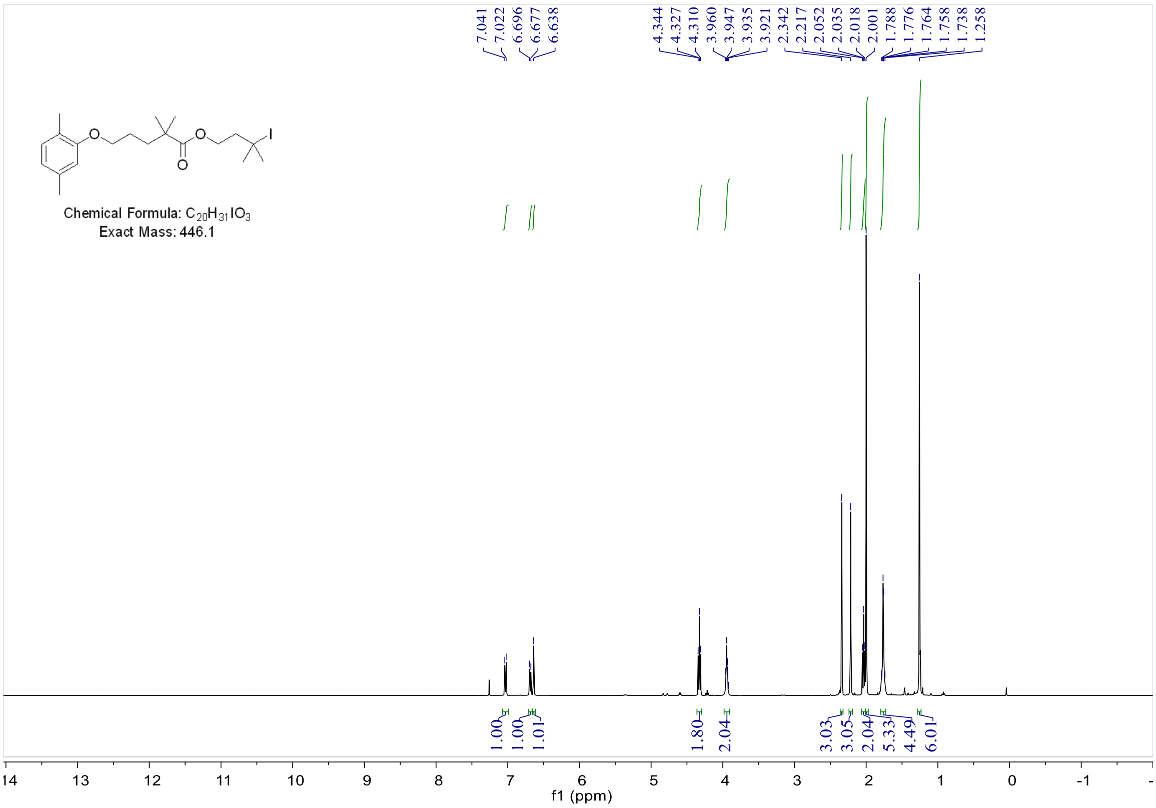

Supplementary Figure 16 ^{1}H NMR Spectrum of **2k**

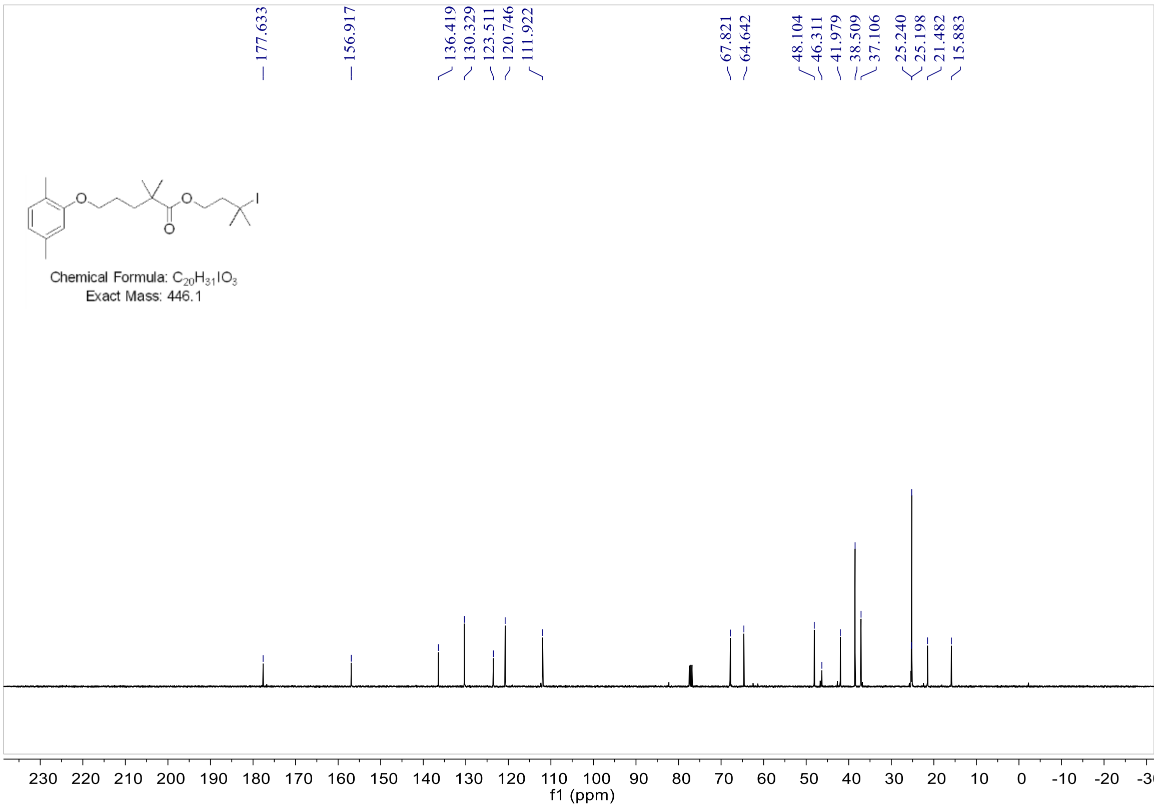

Supplementary Figure 17 ^{13}C NMR Spectrum of **2k**

Supplementary Note 2: Characterization Spectra for Compounds 4 and 5

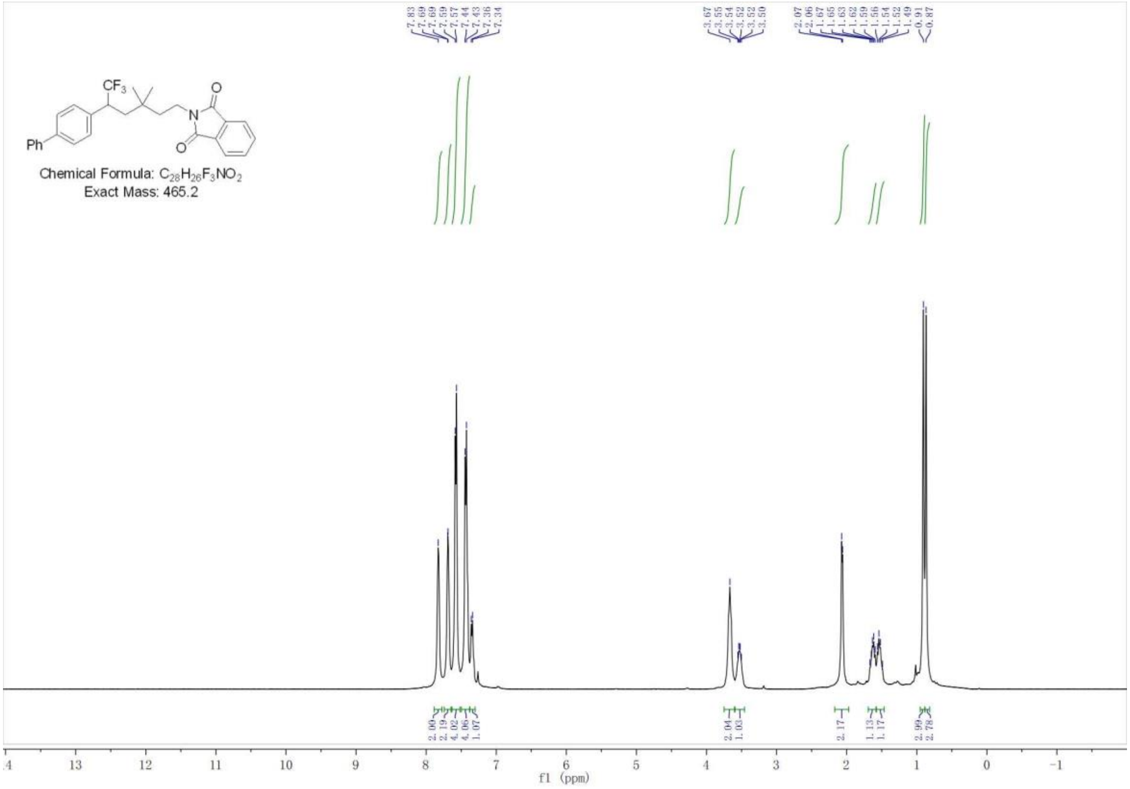

Supplementary Figure 18 ^{1}H NMR Spectrum of **4a**

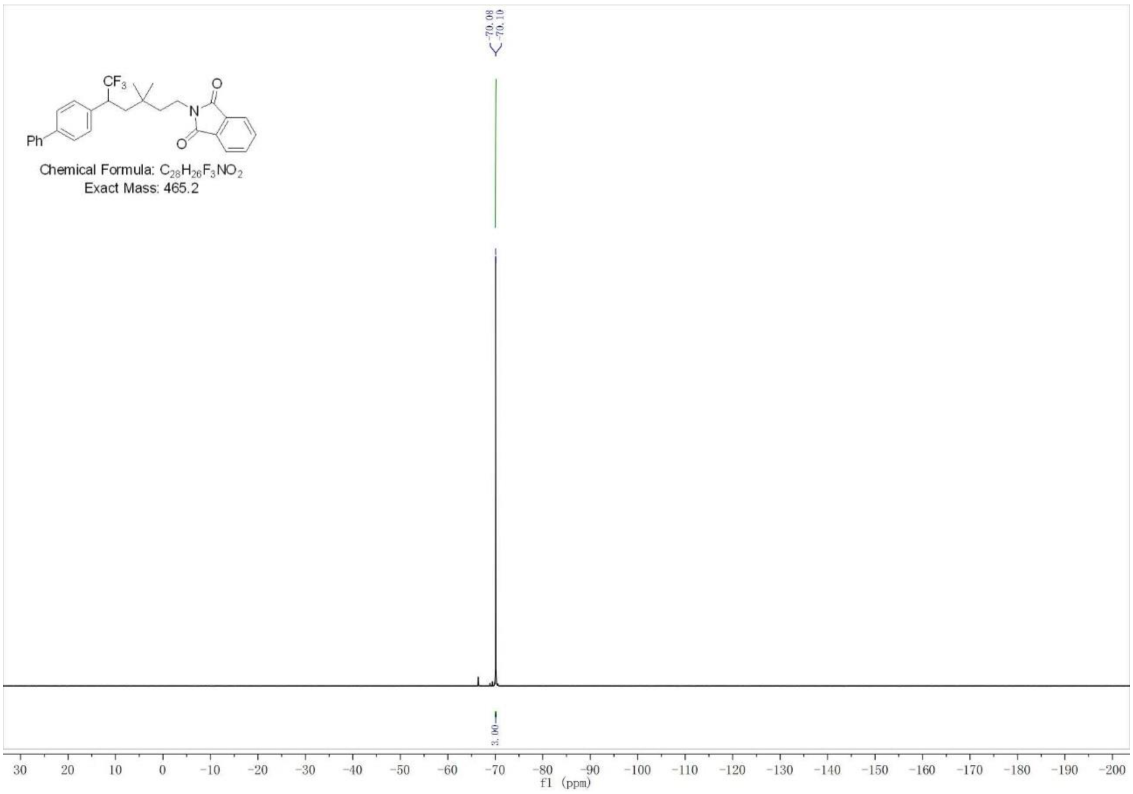

Supplementary Figure 19 ^{19}F NMR Spectrum of **4a**

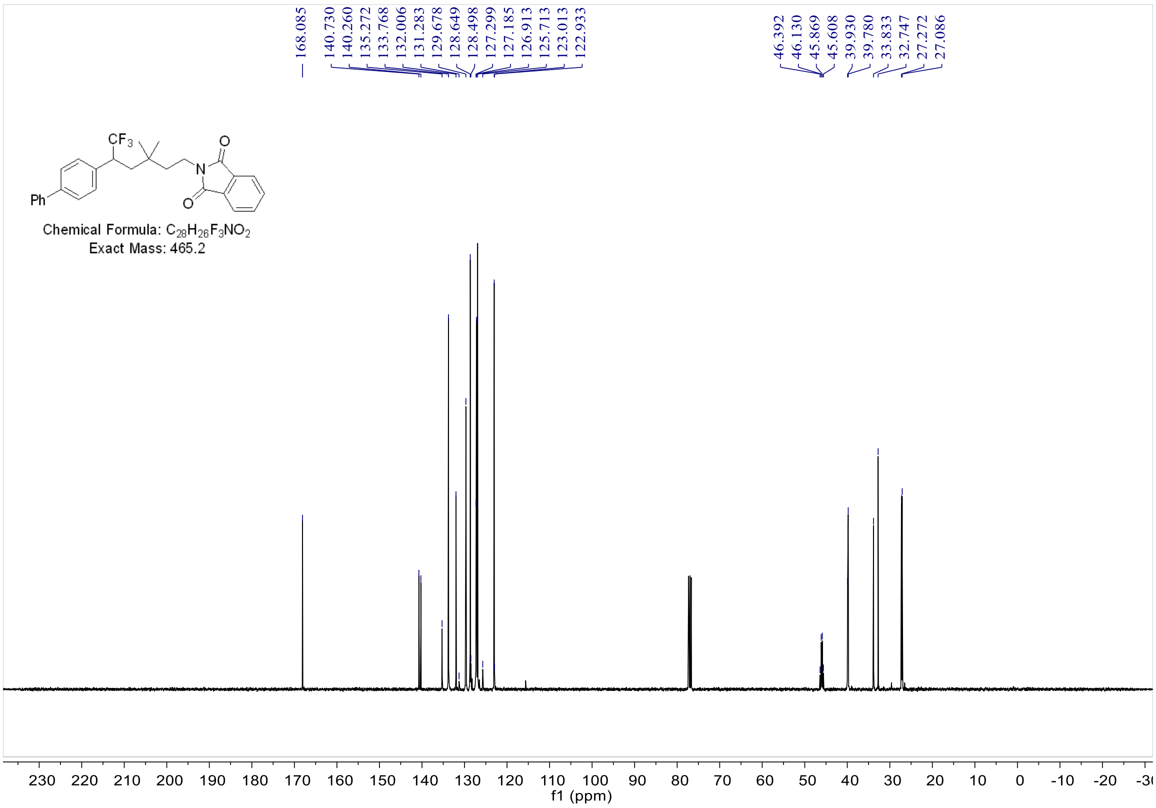

Supplementary Figure 20^{13}C NMR Spectrum of **4a**

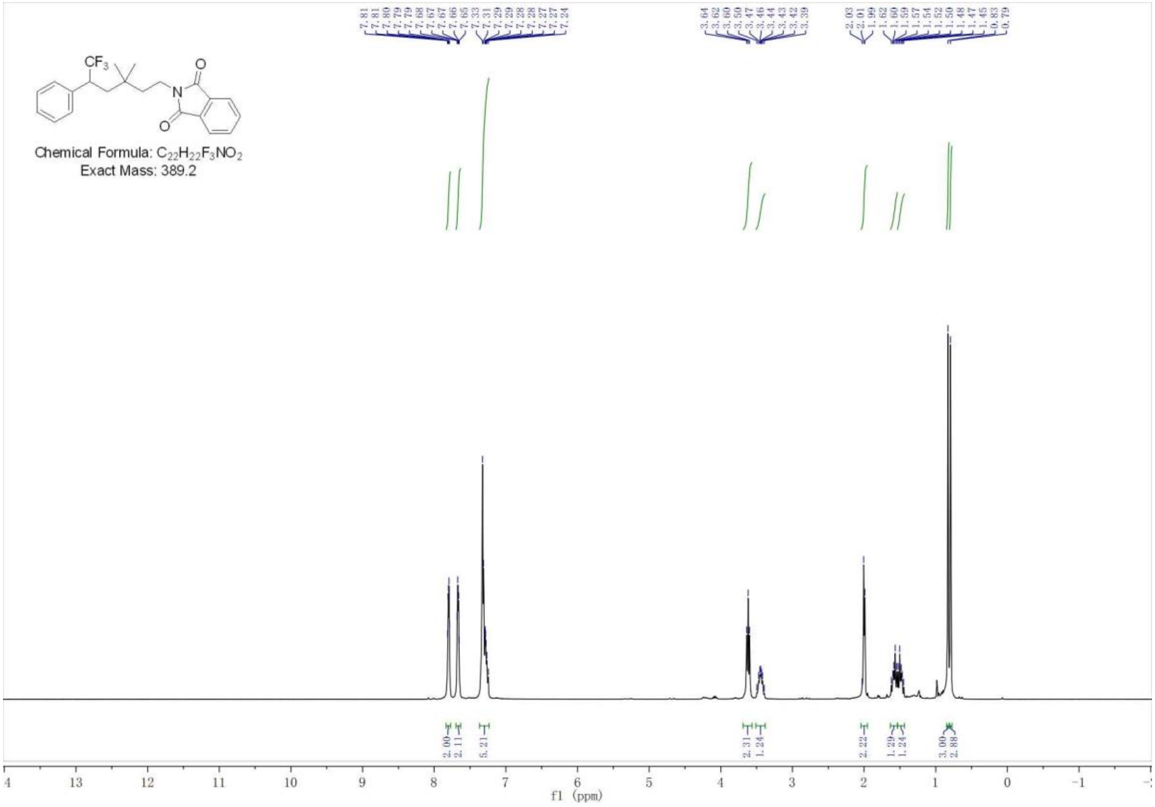

Supplementary Figure 21 ^{1}H NMR Spectrum of **4b**

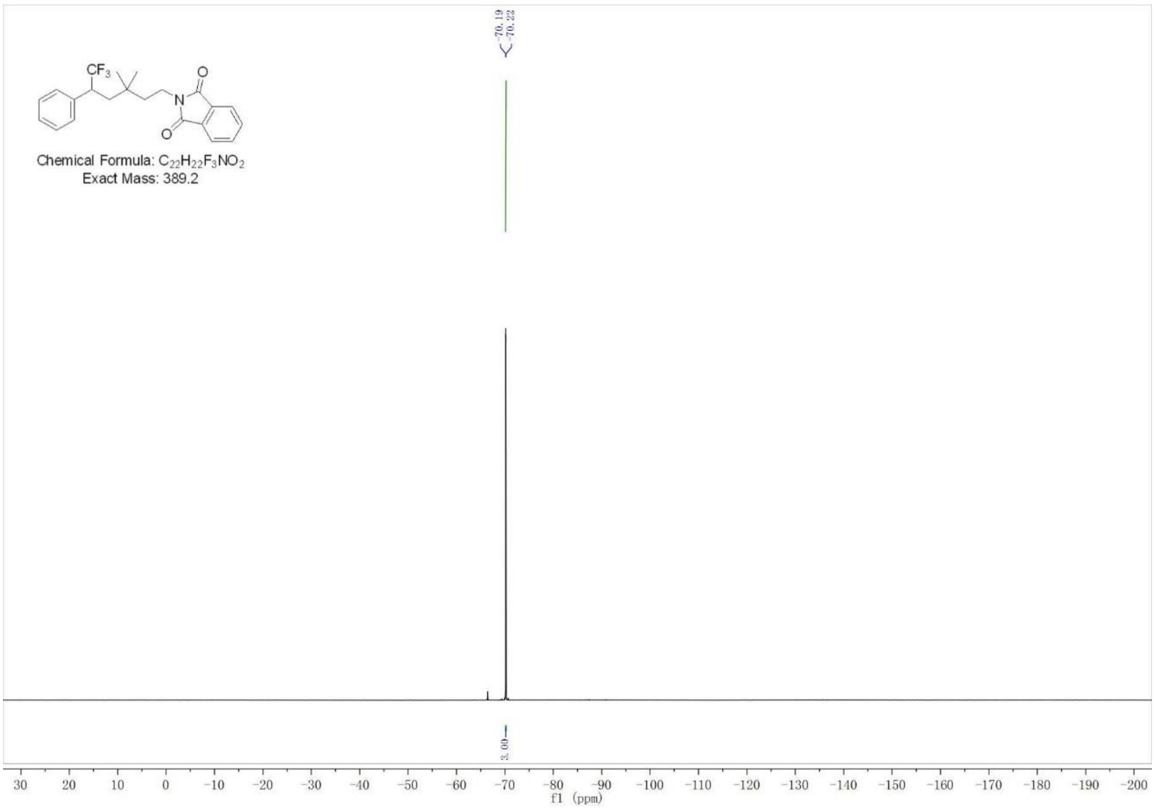

Supplementary Figure 22 ^{19}F NMR Spectrum of **4b**

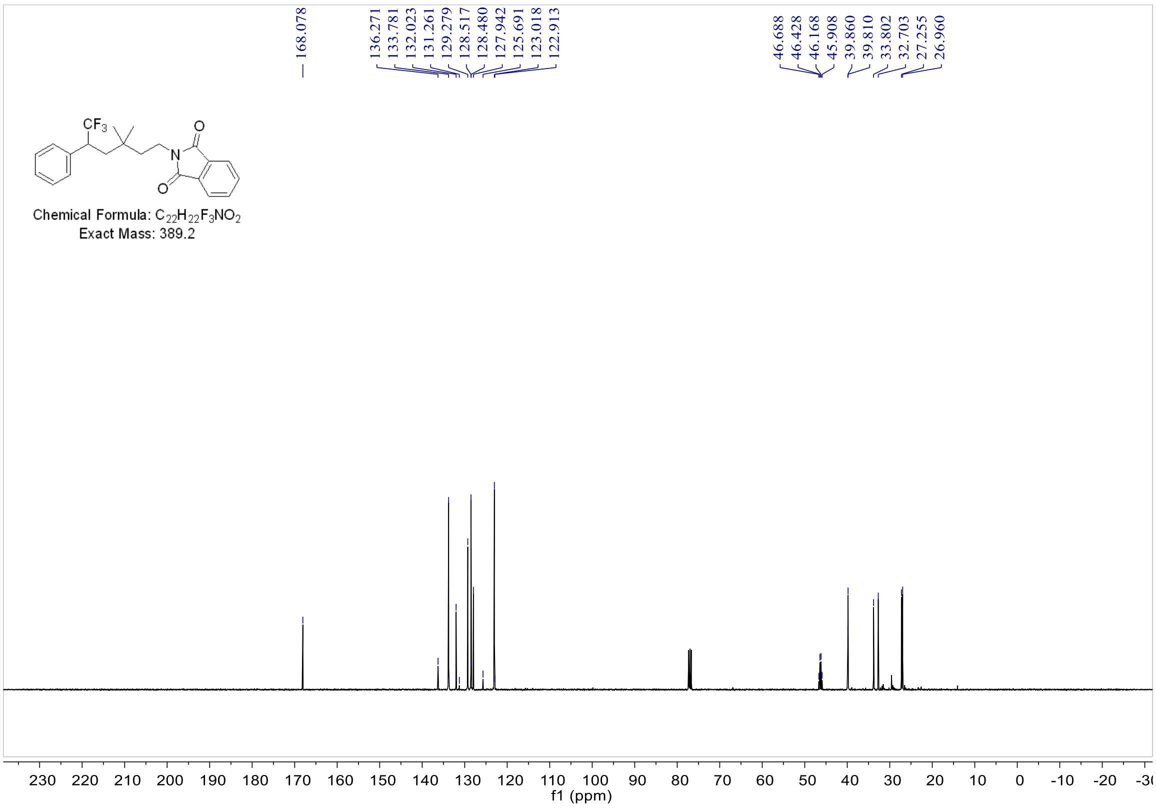

Supplementary Figure 23 ^{13}C NMR Spectrum of **4b**

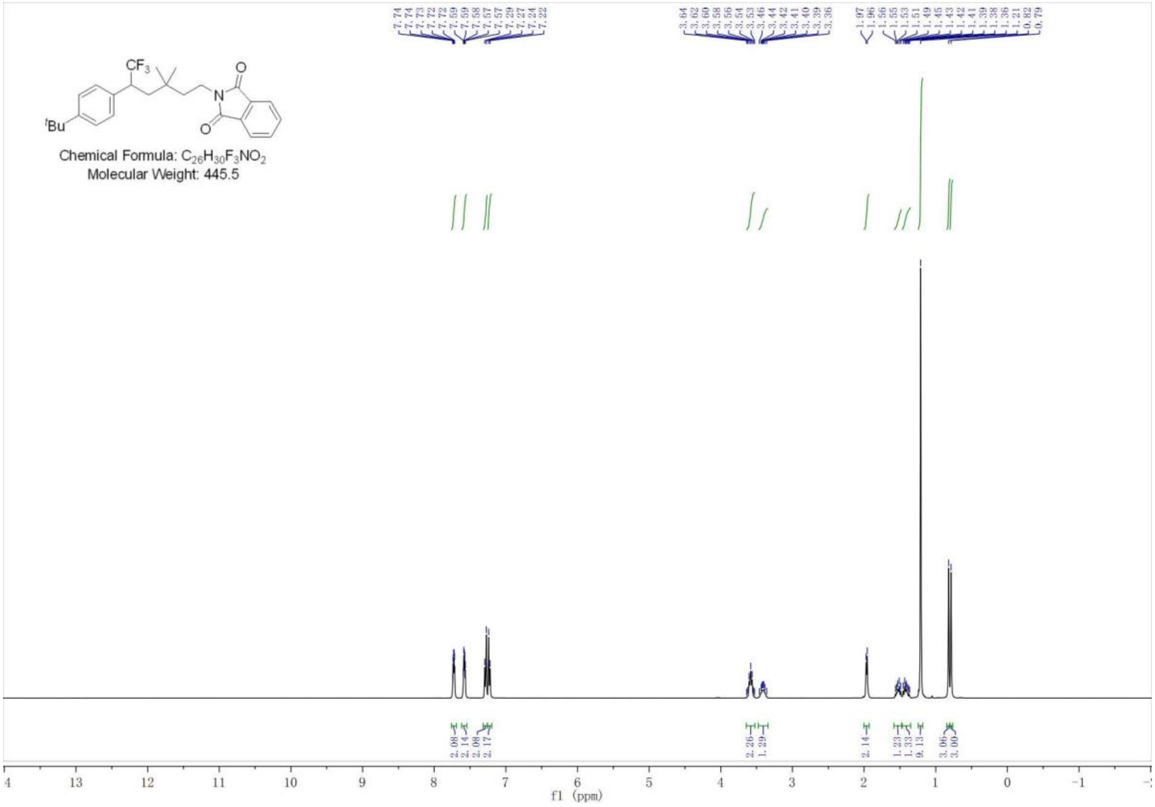

Supplementary Figure 24 ^{1}H NMR Spectrum of **4c**

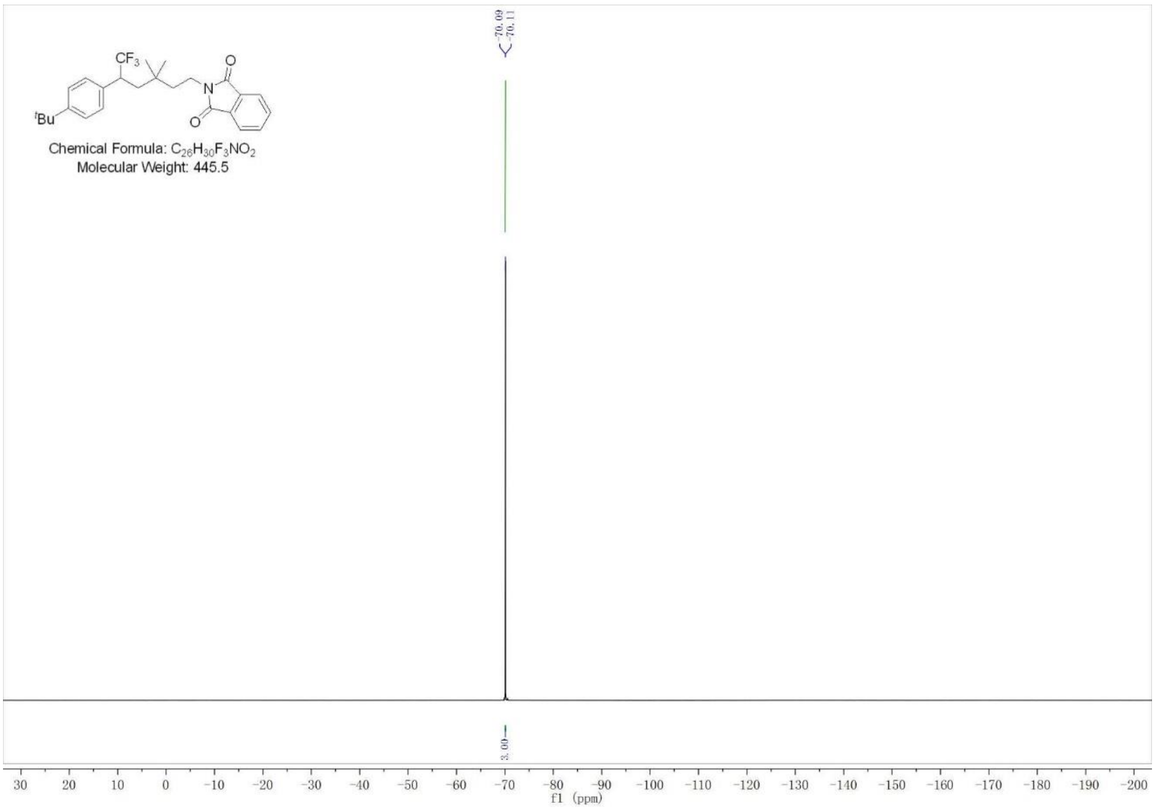

Supplementary Figure 25 ^{19}F NMR Spectrum of **4c**

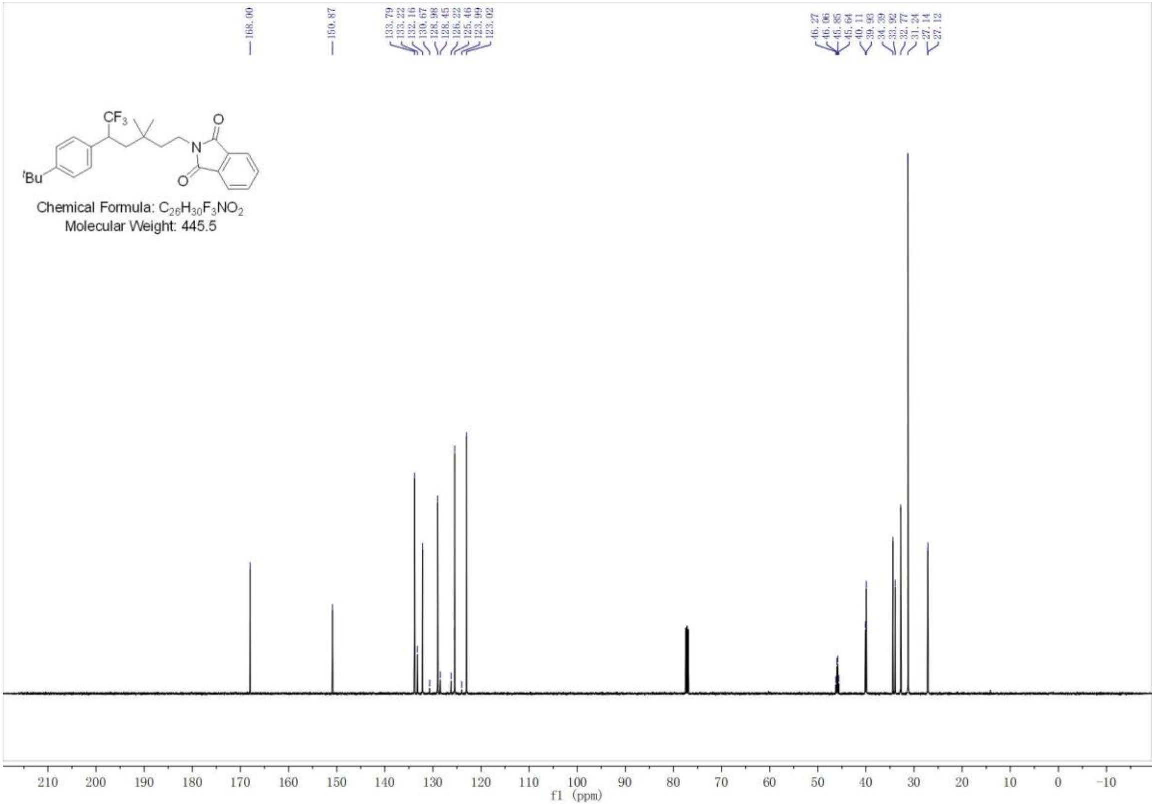

Supplementary Figure 26 ^{13}C NMR Spectrum of **4c**

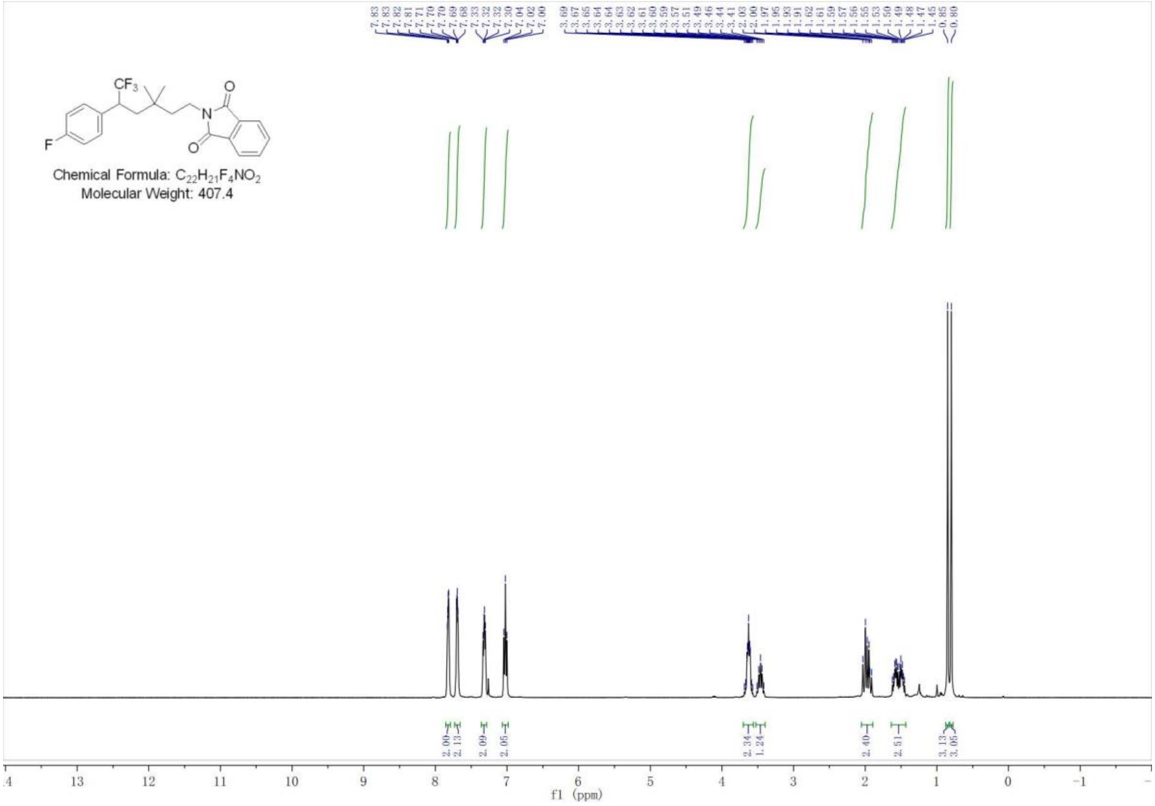

Supplementary Figure 27 ^{1}H NMR Spectrum of **4d**

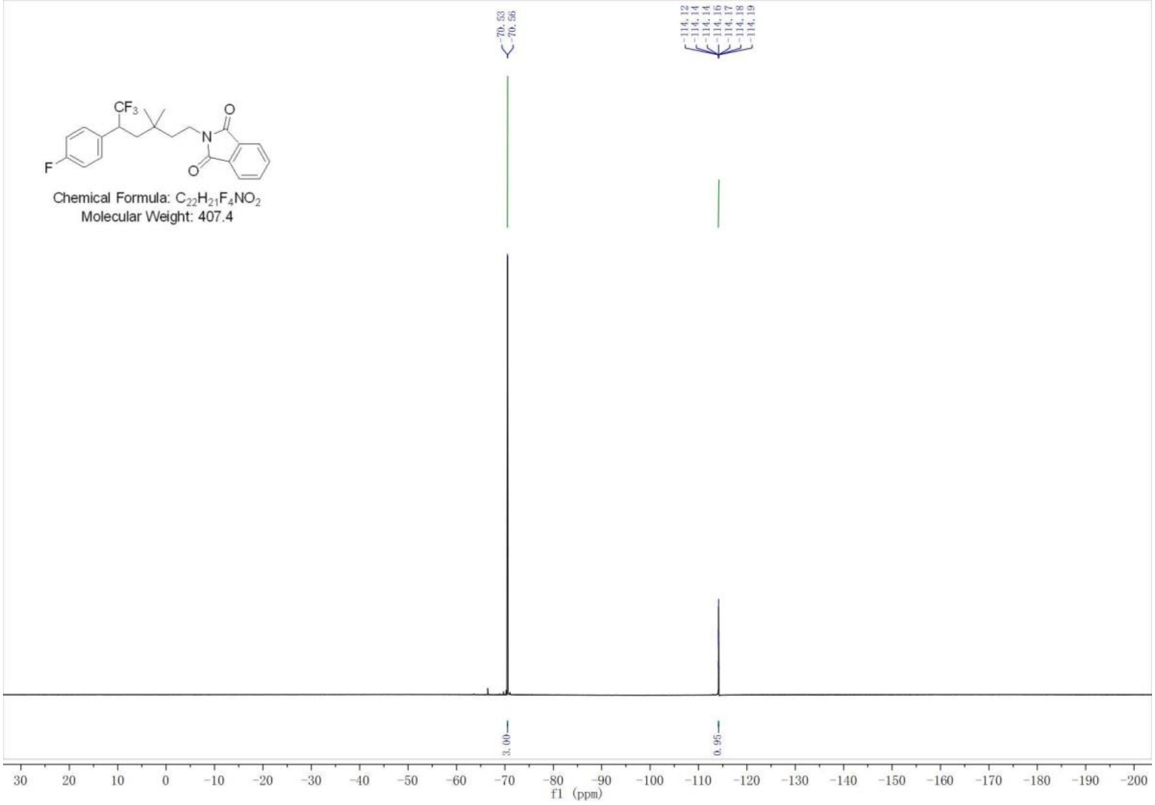

Supplementary Figure 28 ^{19}F NMR Spectrum of **4d**

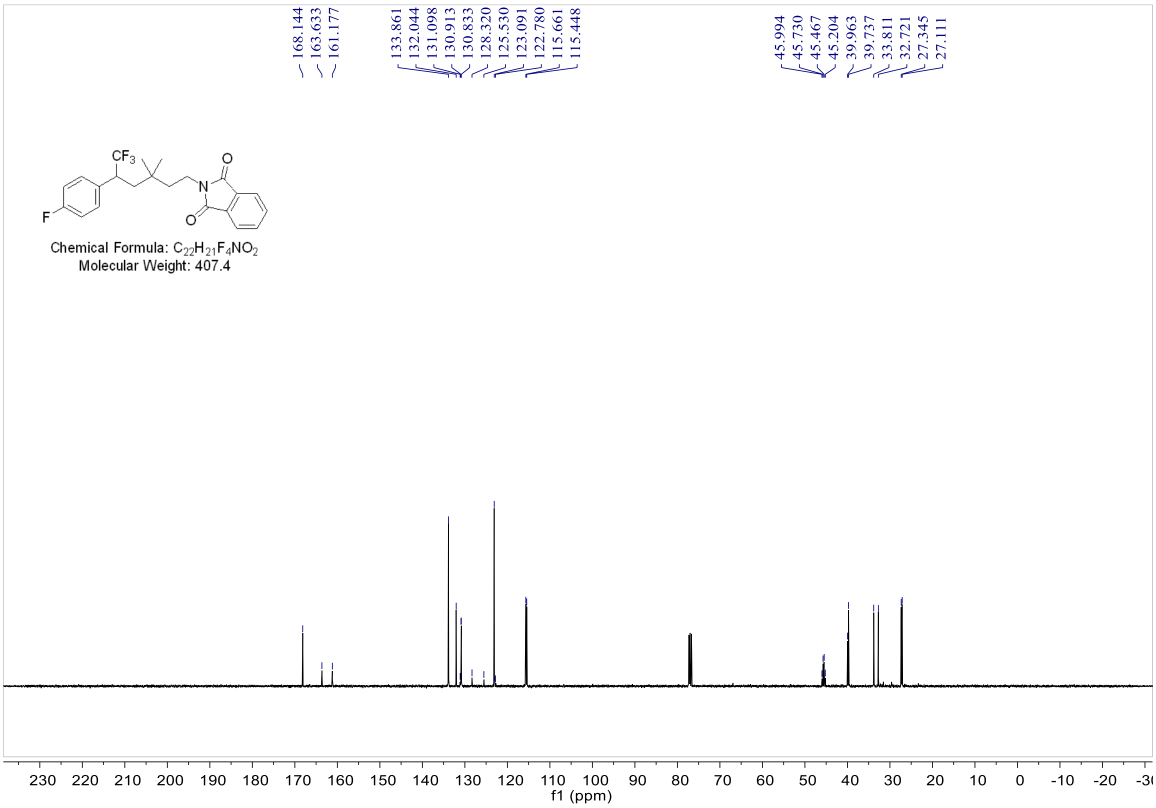

Supplementary Figure 29 ^{13}C NMR Spectrum of **4d**

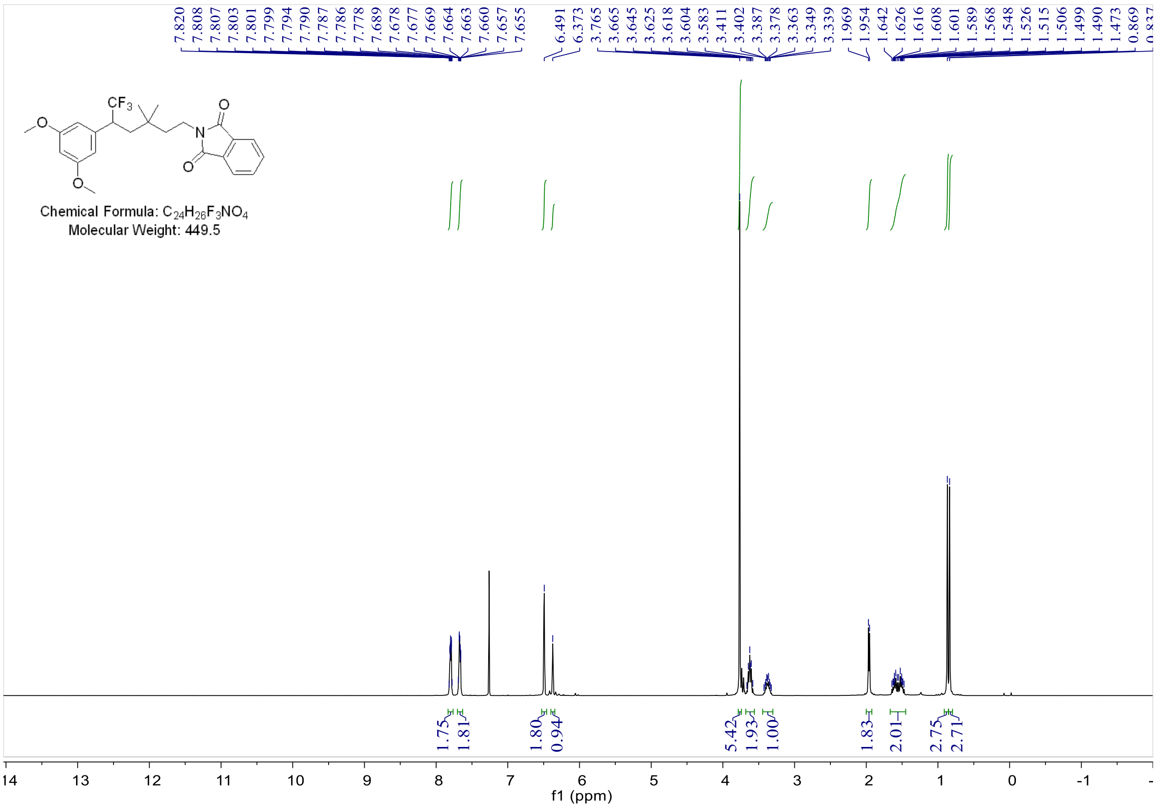

Supplementary Figure 30 ^{1}H NMR Spectrum of **4e**

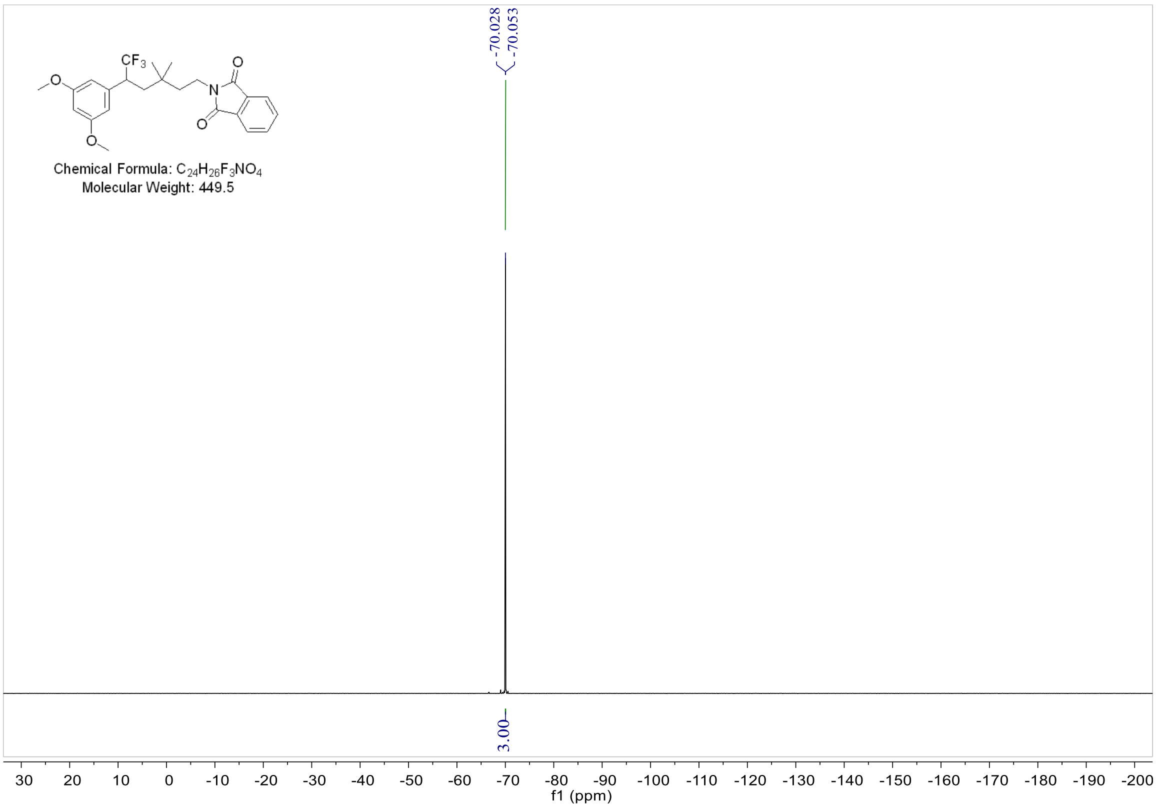

Supplementary Figure 31 ^{19}F NMR Spectrum of **4e**

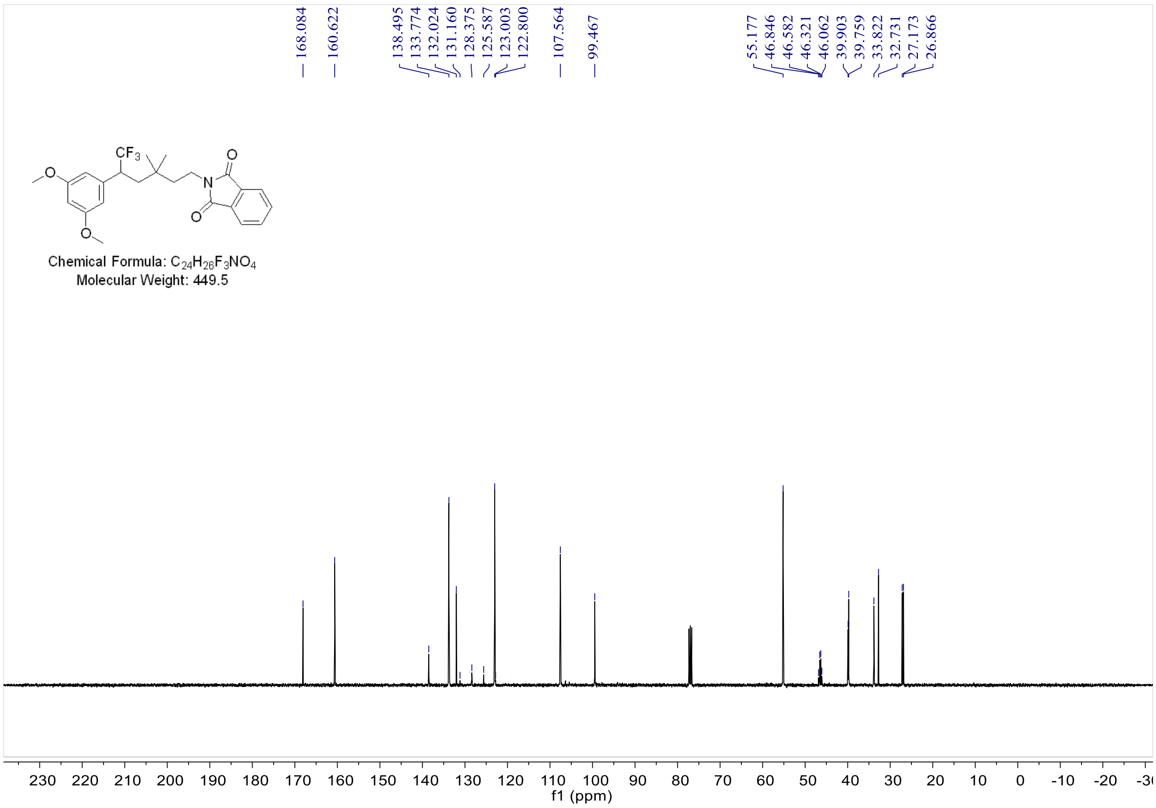

Supplementary Figure 32 ^{13}C NMR Spectrum of **4e**

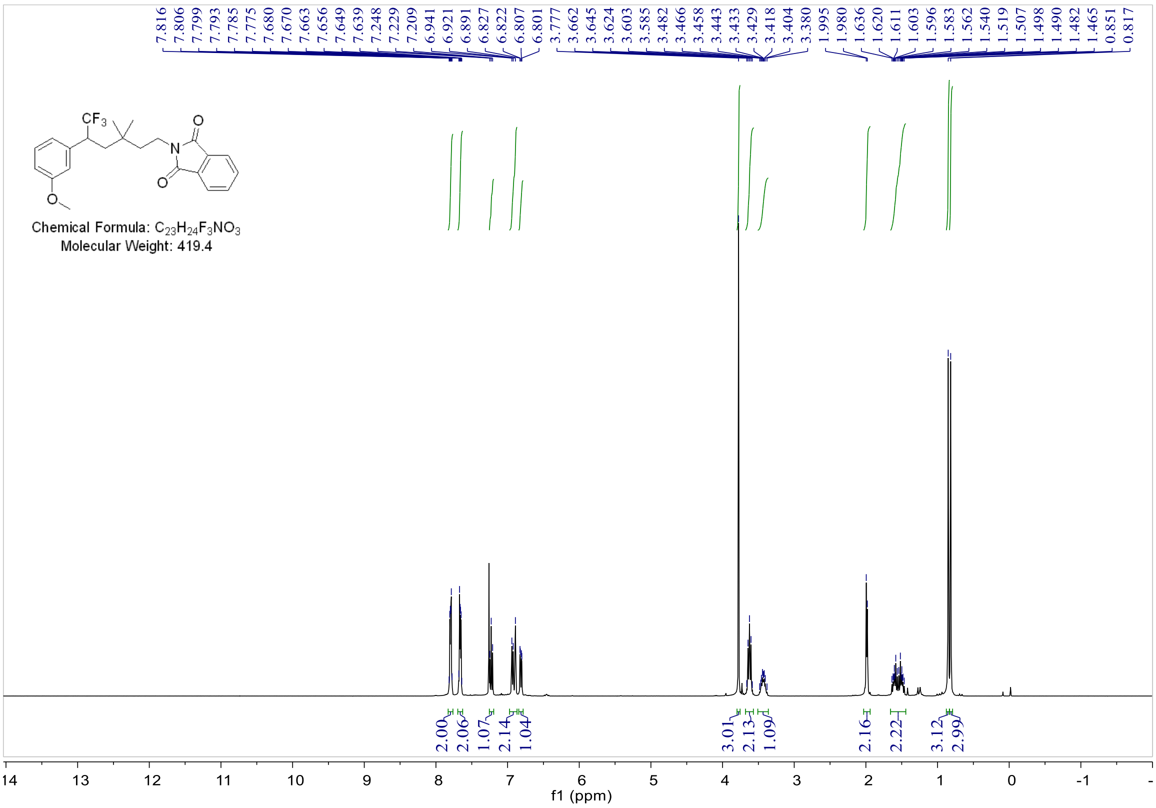

Supplementary Figure 33 ^{1}H NMR Spectrum of **4f**

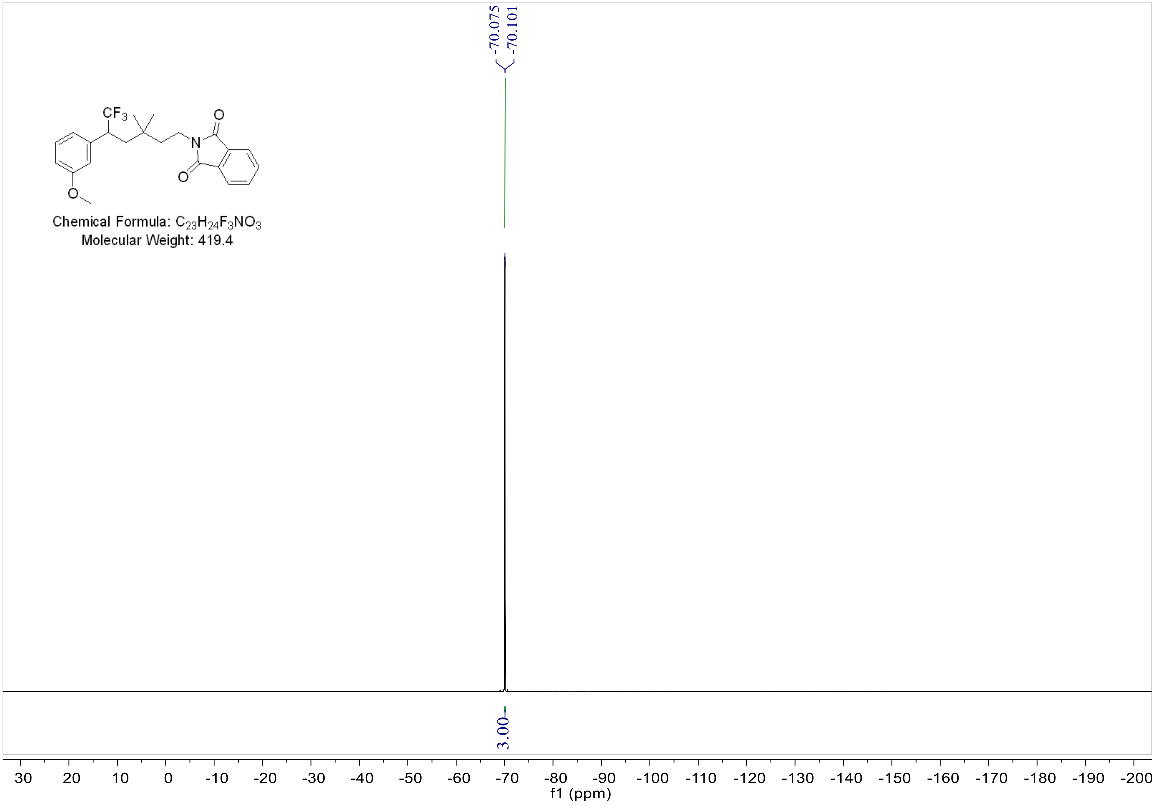

Supplementary Figure 34 ^{19}F NMR Spectrum of **4f**

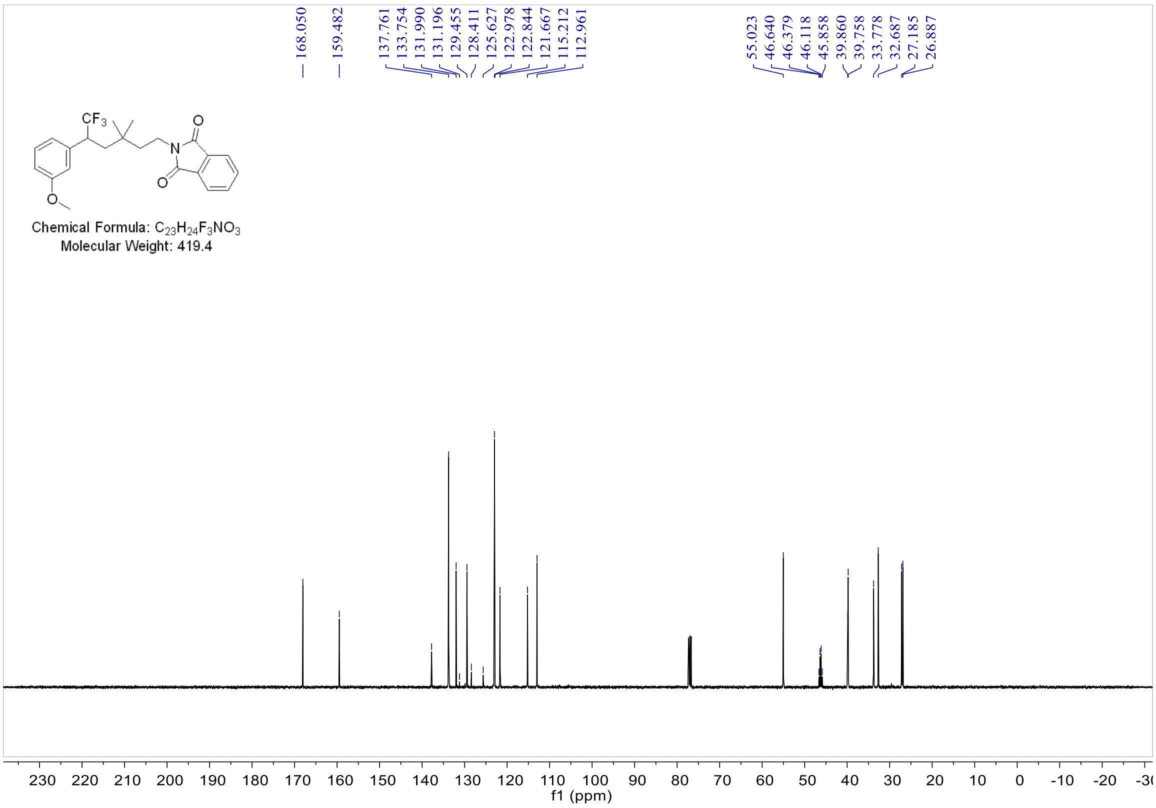

Supplementary Figure 35 ^{13}C NMR Spectrum of **4f**

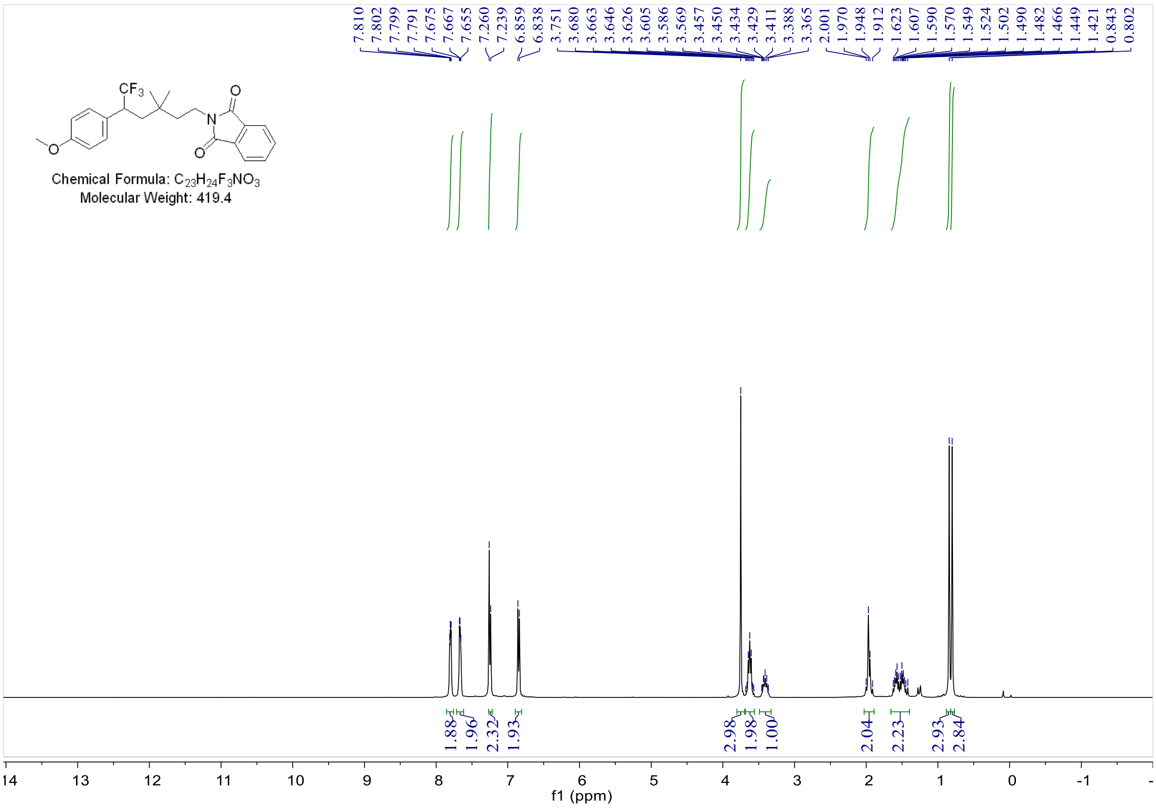

Supplementary Figure 36 ^{1}H NMR Spectrum of **4g**

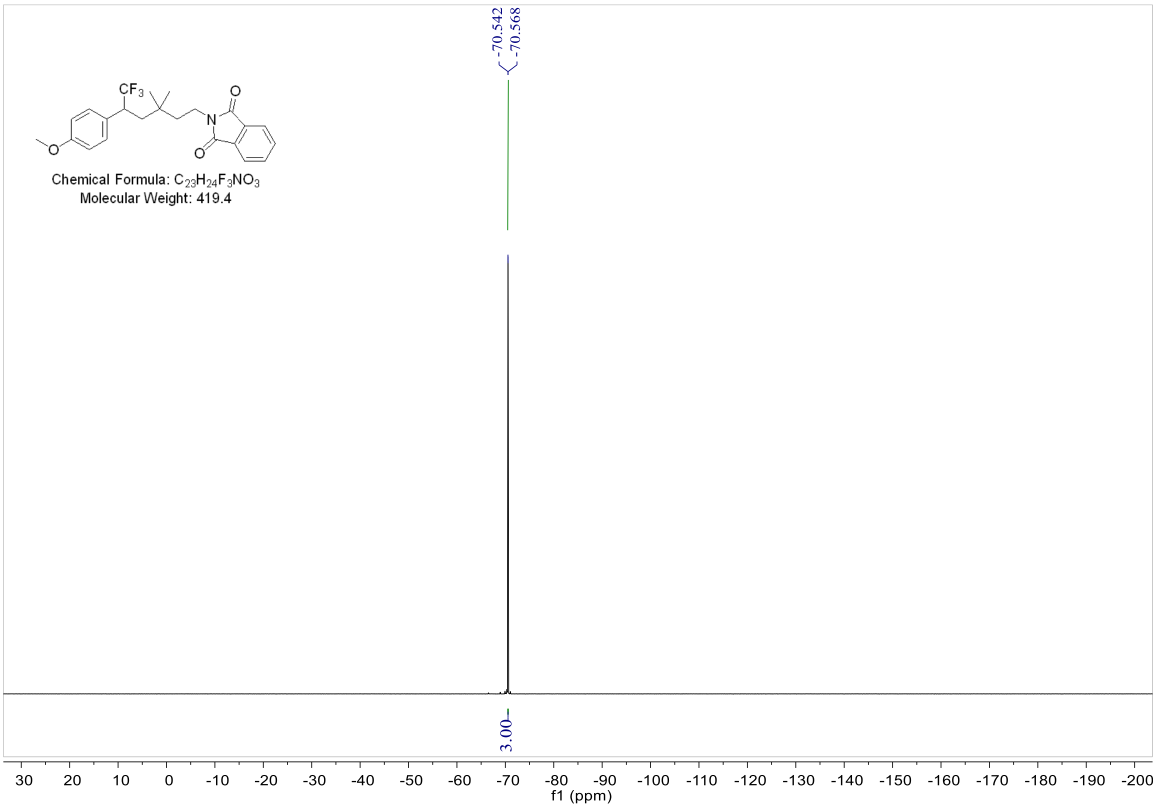

Supplementary Figure 37 ^{19}F NMR Spectrum of **4g**

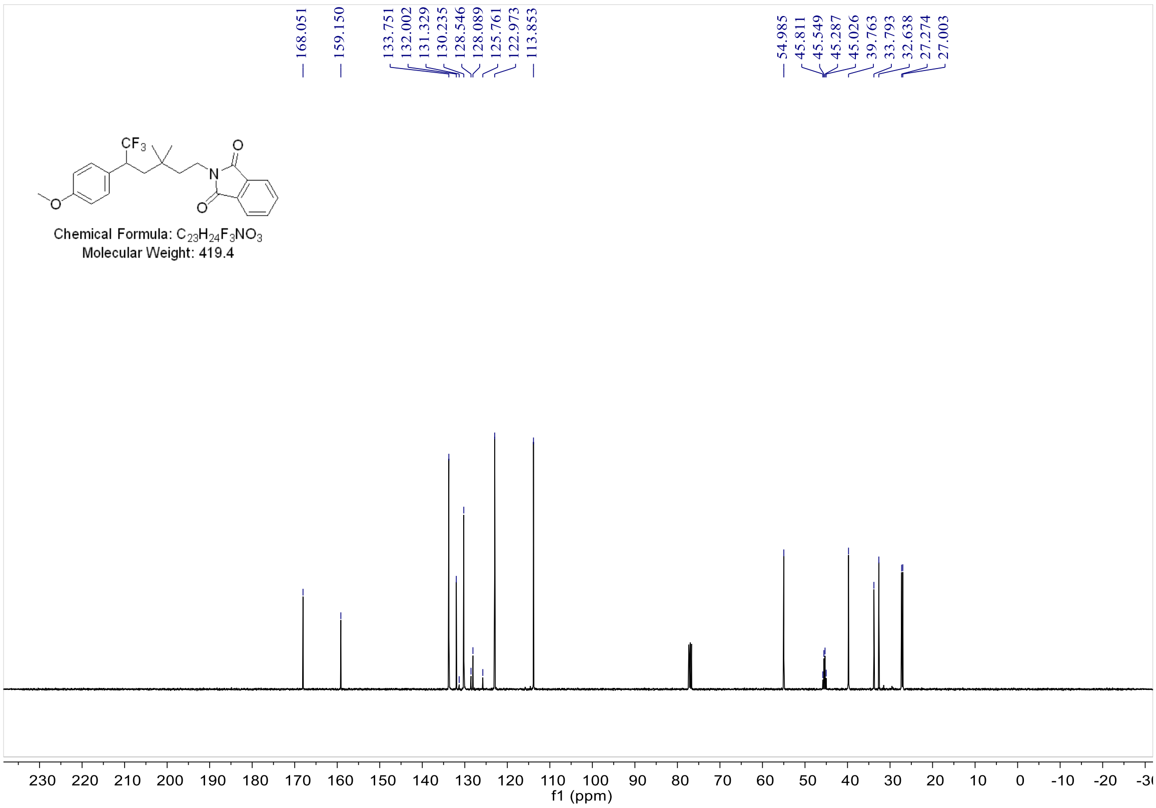

Supplementary Figure 38 ^{13}C NMR Spectrum of **4g**

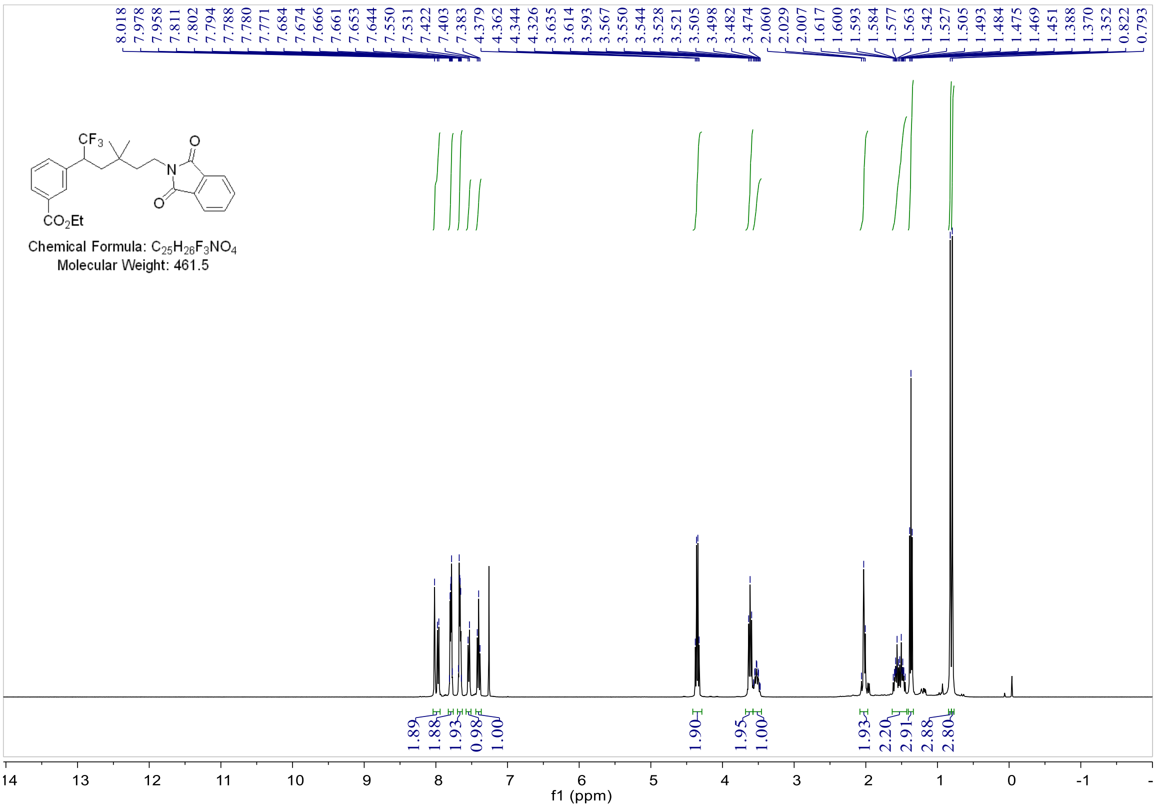

Supplementary Figure 39 ^{1}H NMR Spectrum of **4h**

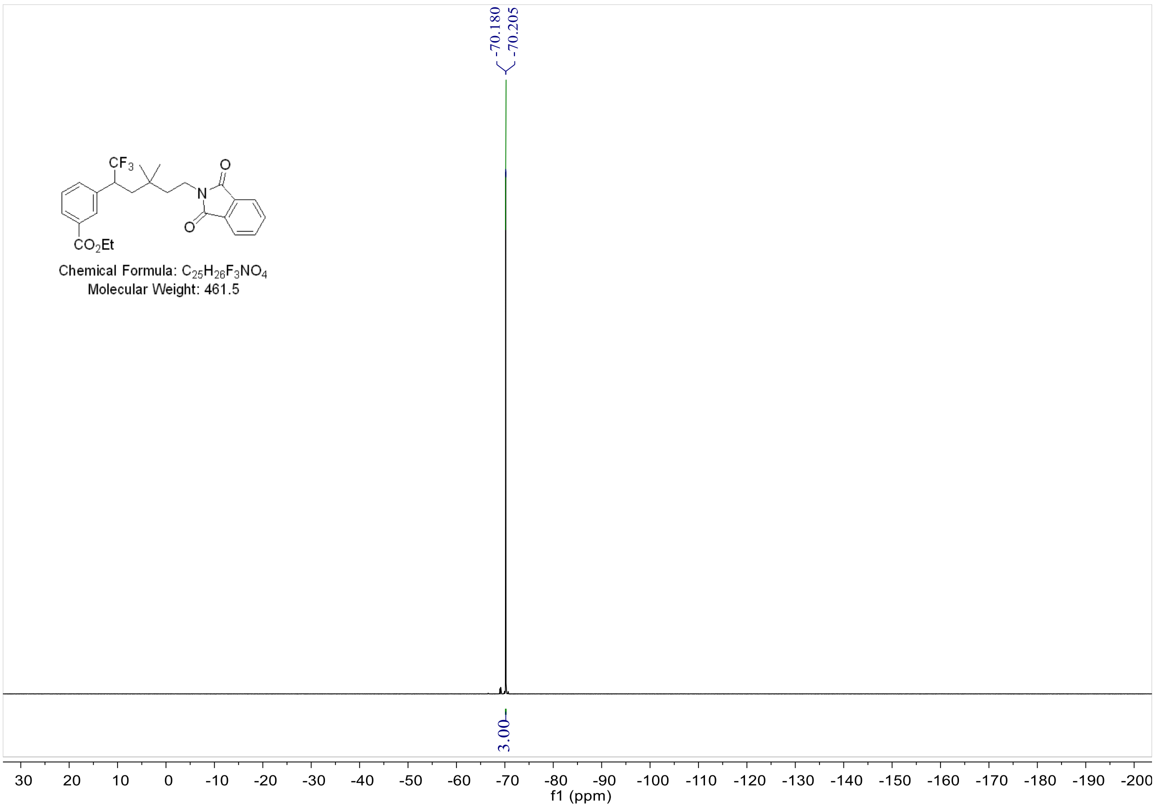

Supplementary Figure 40 ^{19}F NMR Spectrum of **4h**

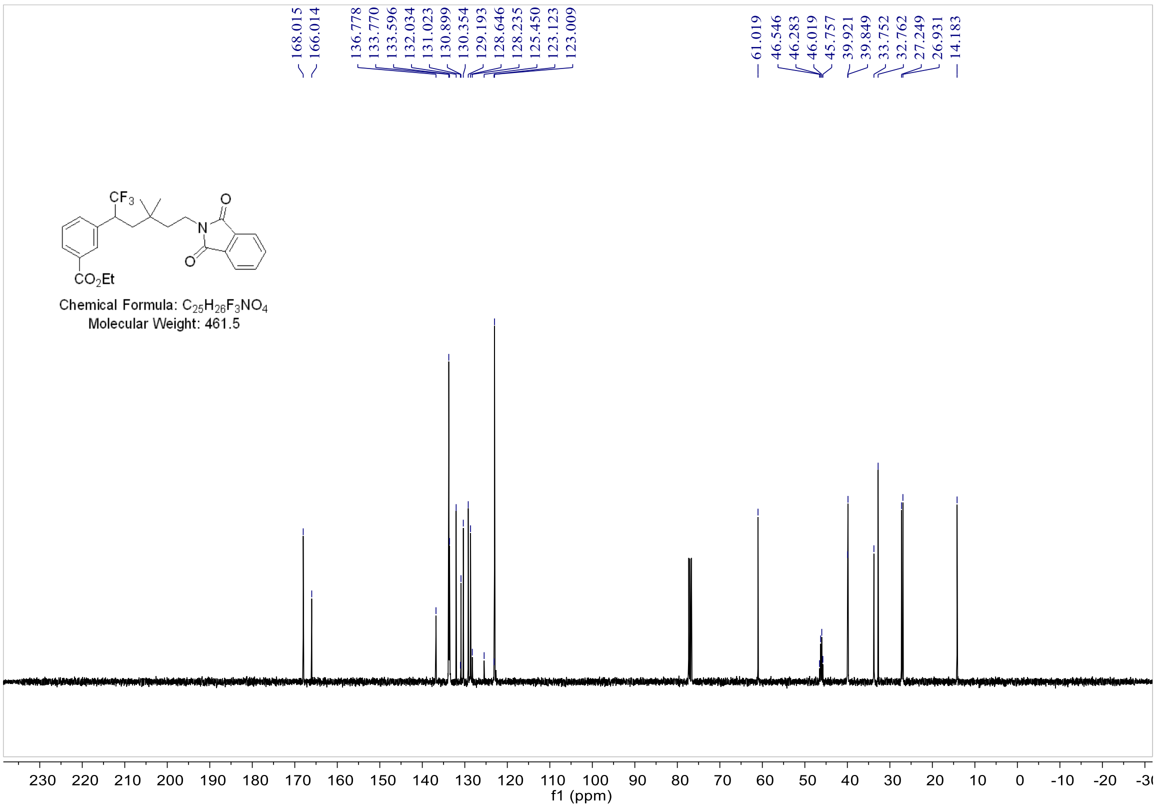

Supplementary Figure 41 ^{13}C NMR Spectrum of **4h**

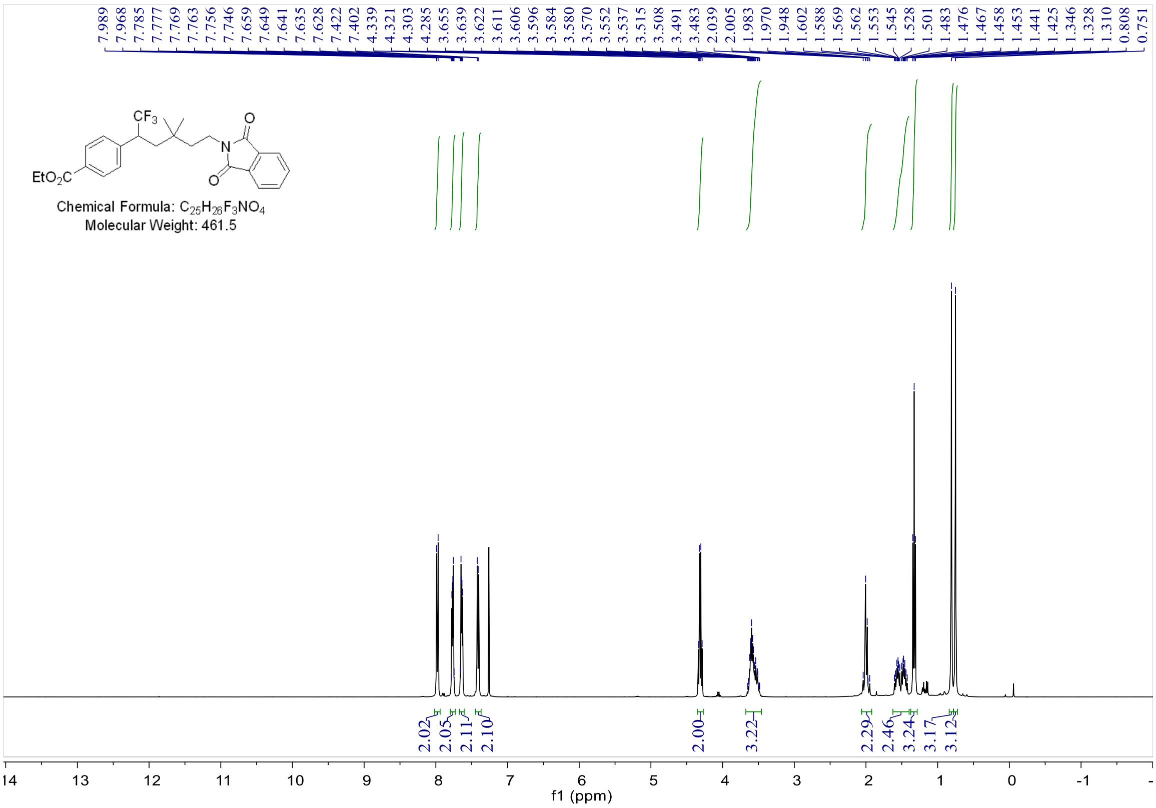

Supplementary Figure 42 ^{1}H NMR Spectrum of **4i**

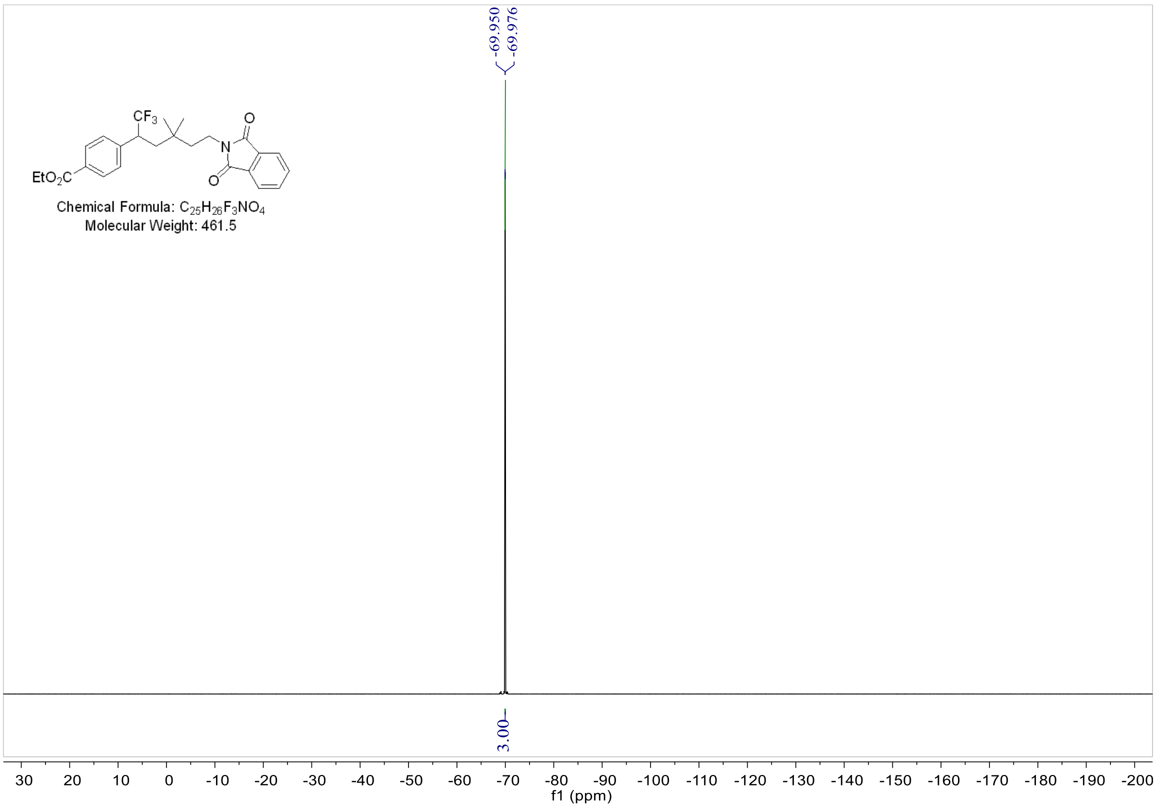

Supplementary Figure 43 ^{19}F NMR Spectrum of **4i**

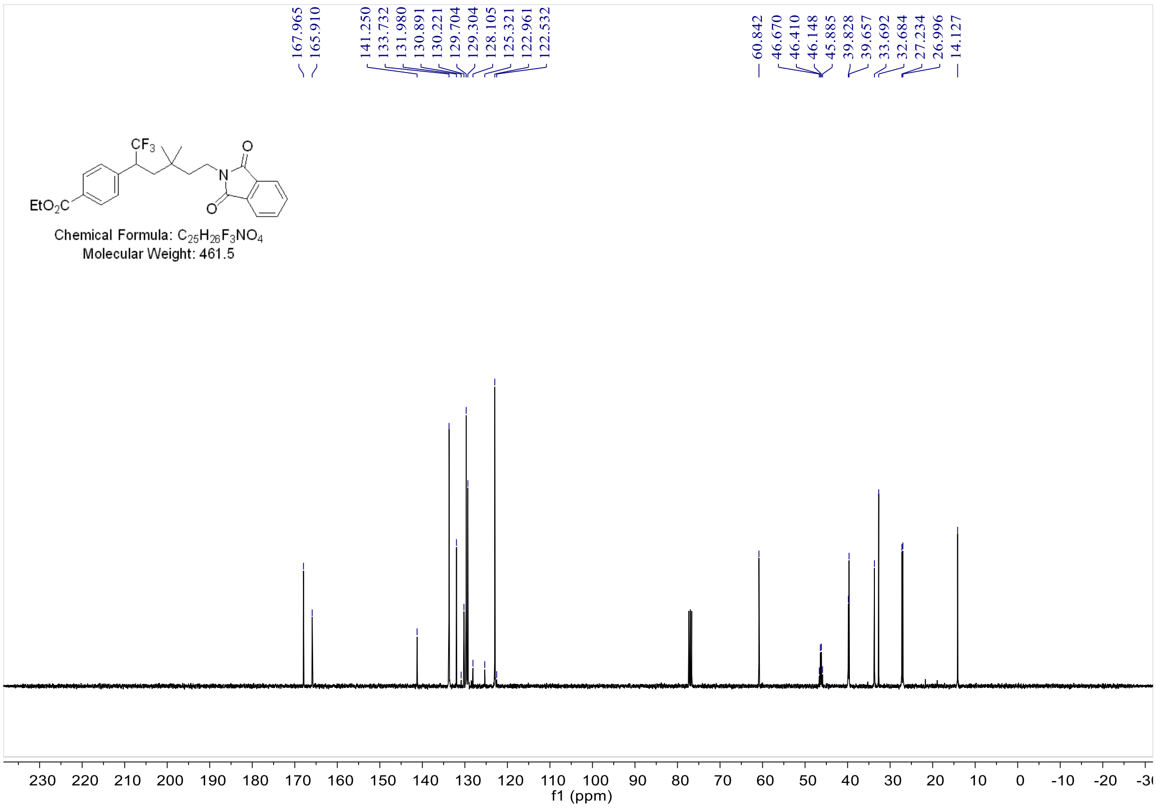

Supplementary Figure 44 ^{13}C NMR Spectrum of **4i**

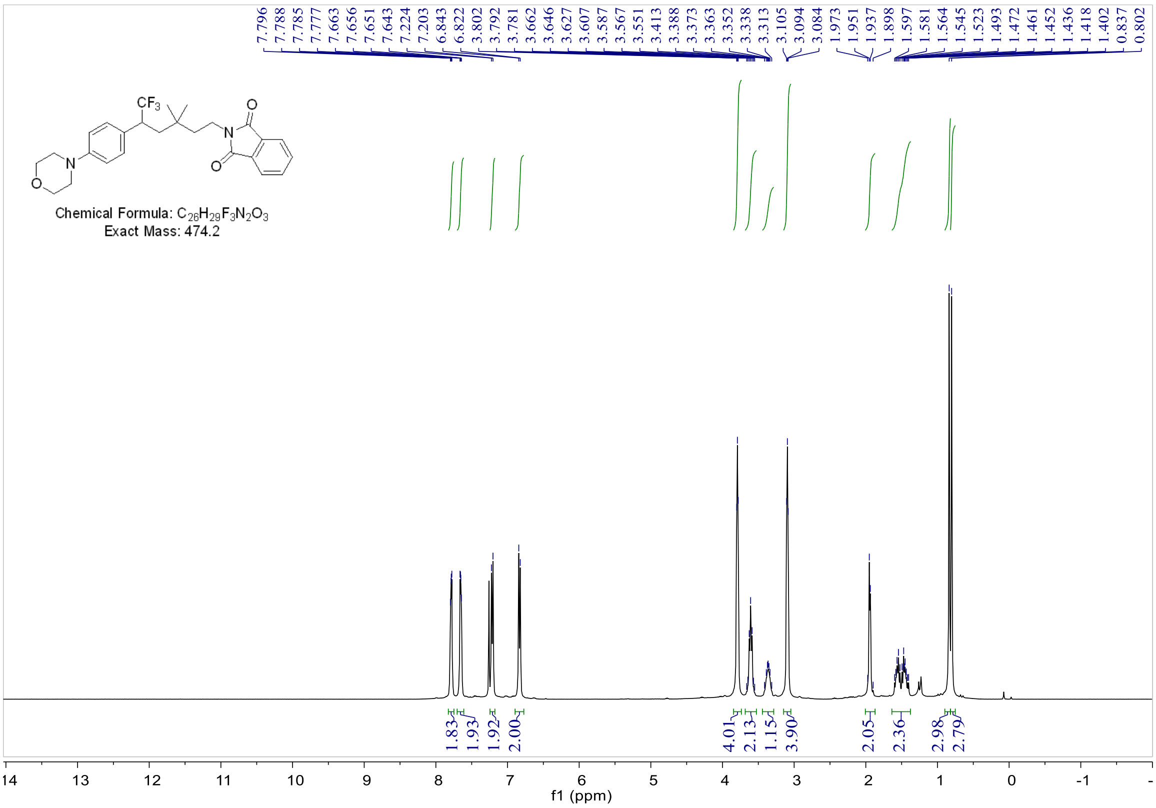

Supplementary Figure 45 ^{1}H NMR Spectrum of **4j**

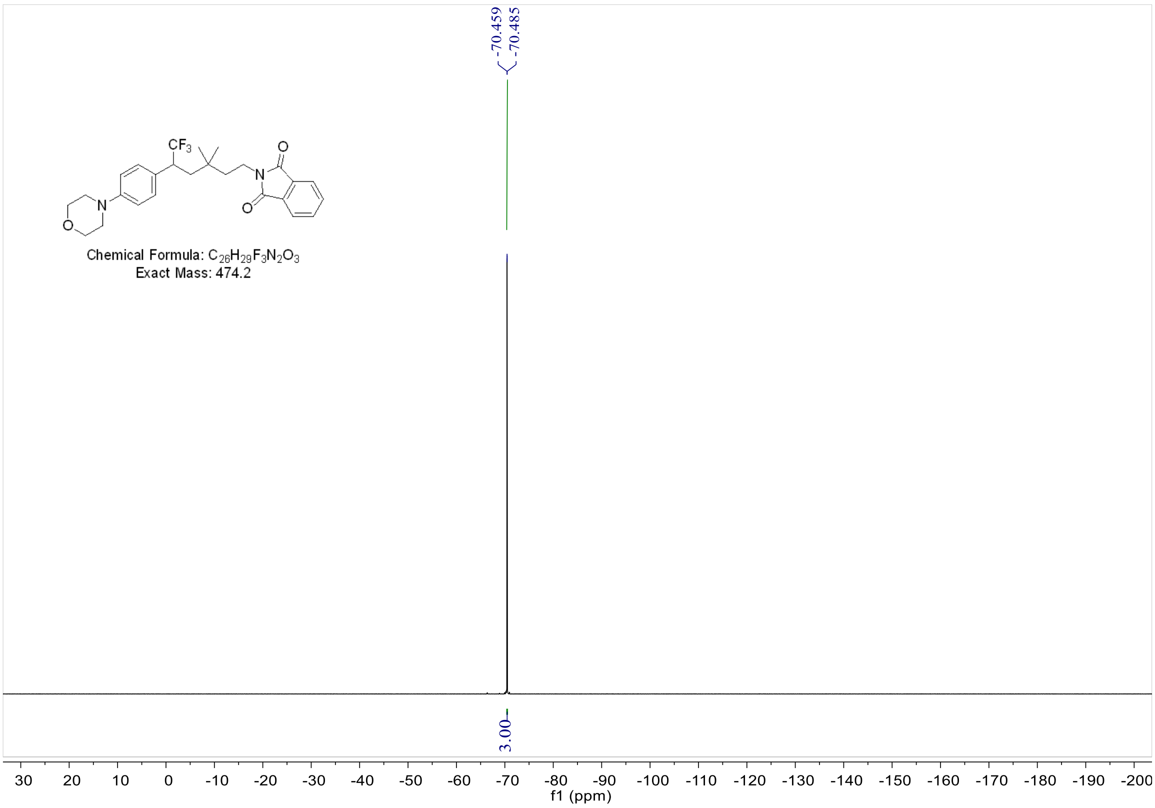

Supplementary Figure 46 ^{19}F NMR Spectrum of **4j**

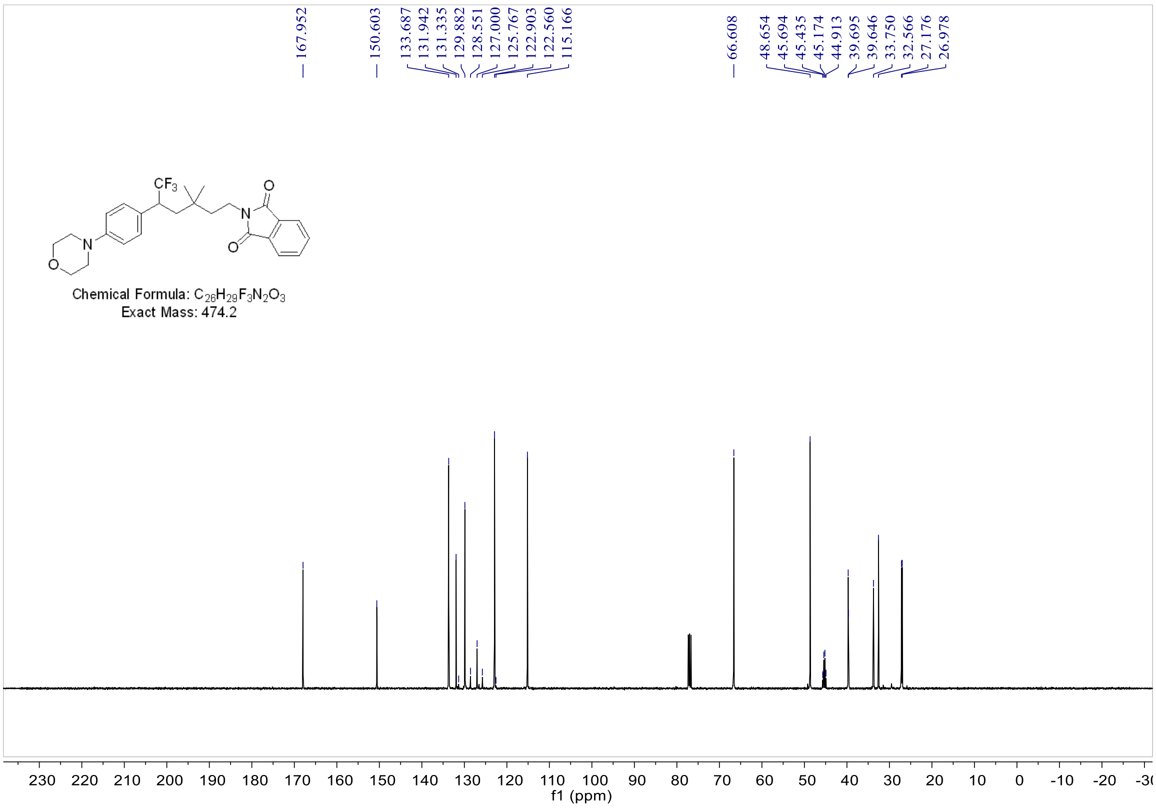

Supplementary Figure 47 ^{13}C NMR Spectrum of **4j**

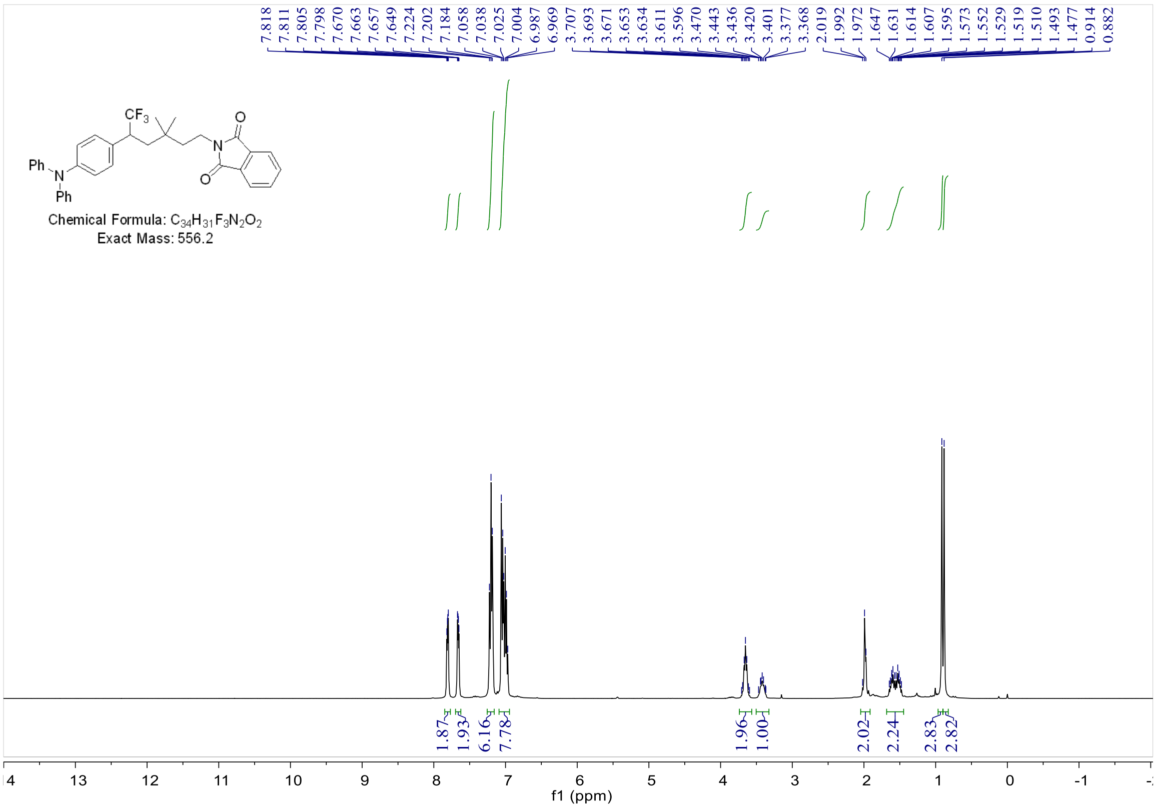

Supplementary Figure 48 ^{1}H NMR Spectrum of **4k**

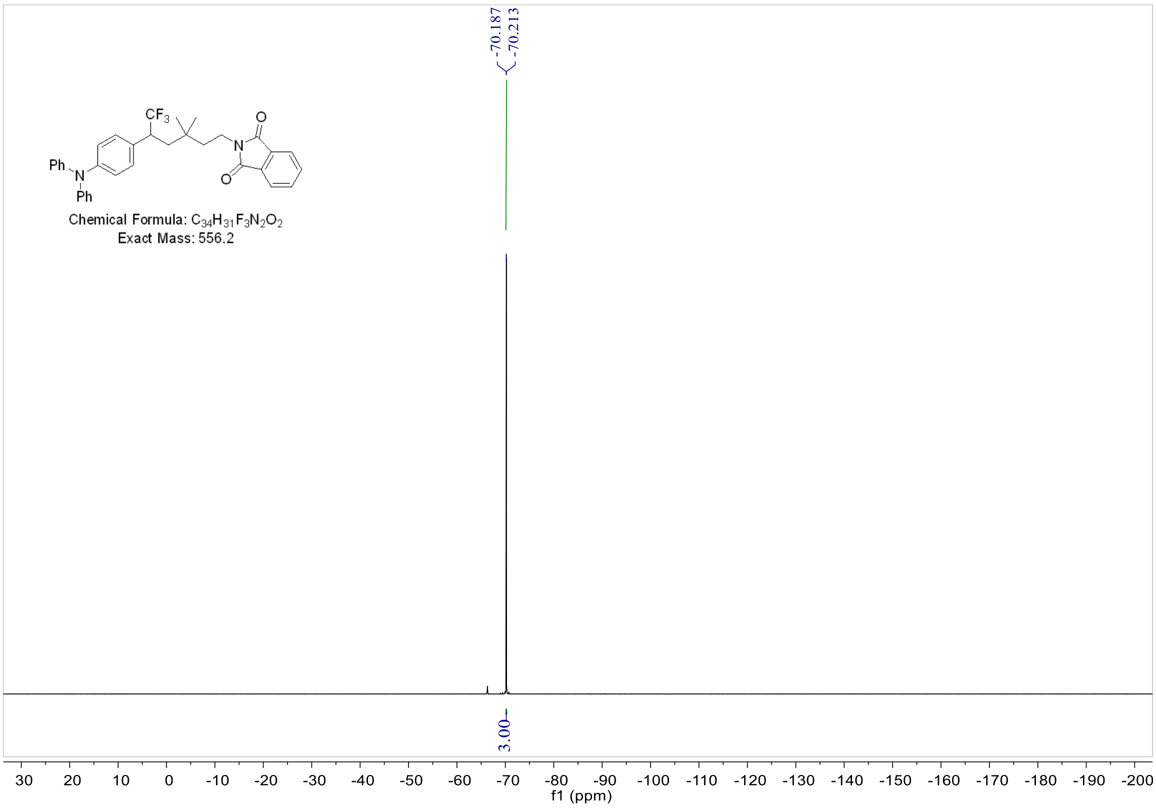

Supplementary Figure 49 ^{19}F NMR Spectrum of **4k**

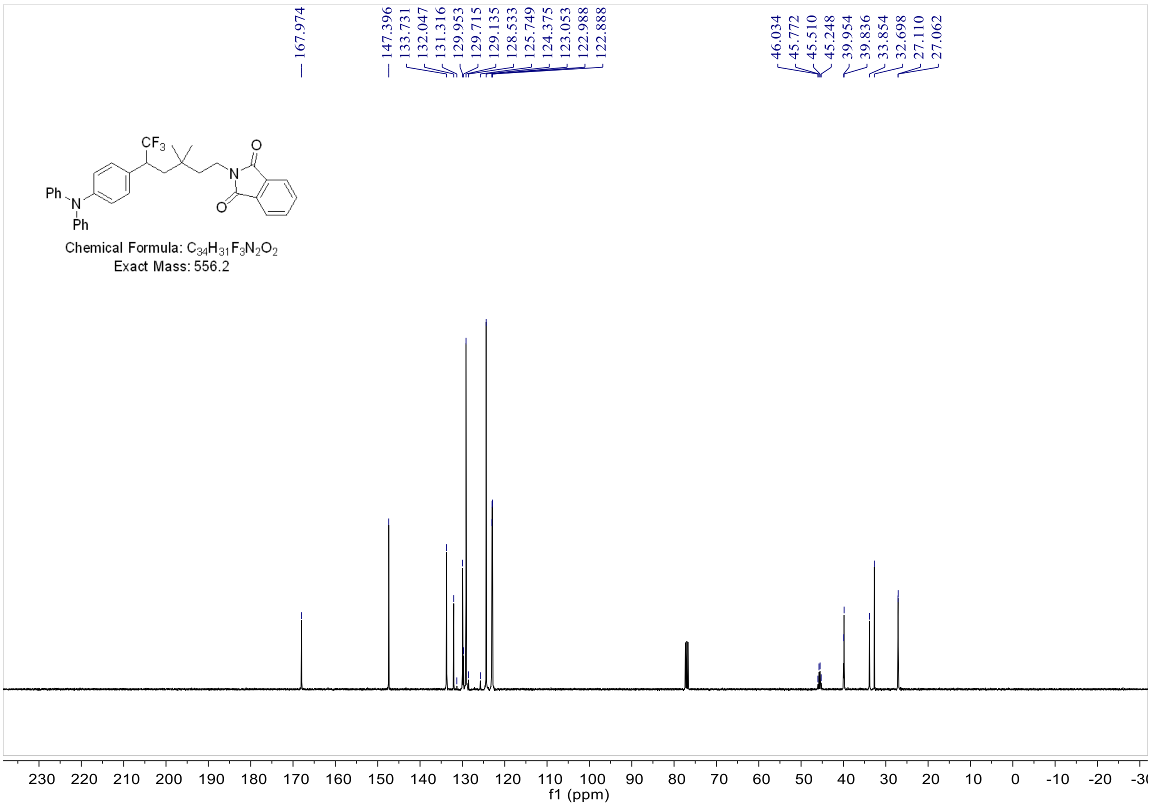

Supplementary Figure 50 ^{13}C NMR Spectrum of **4k**

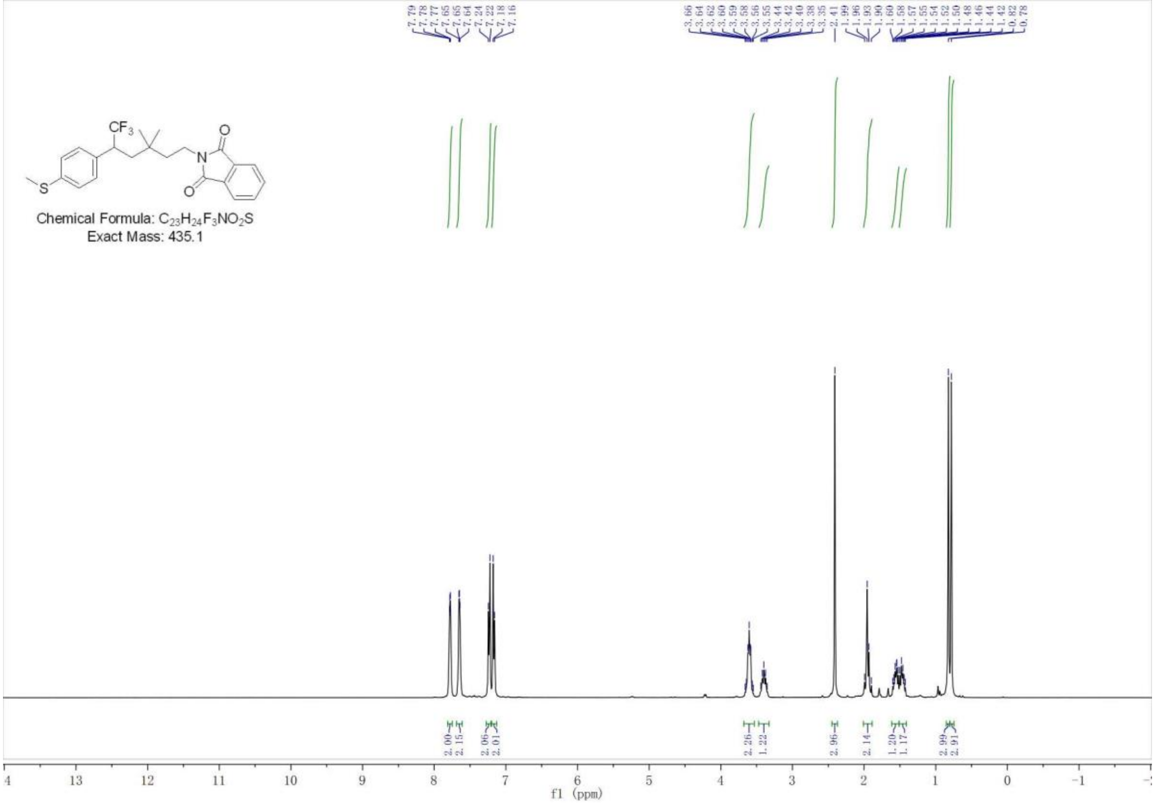

Supplementary Figure 51 ^{1}H NMR Spectrum of **4l**

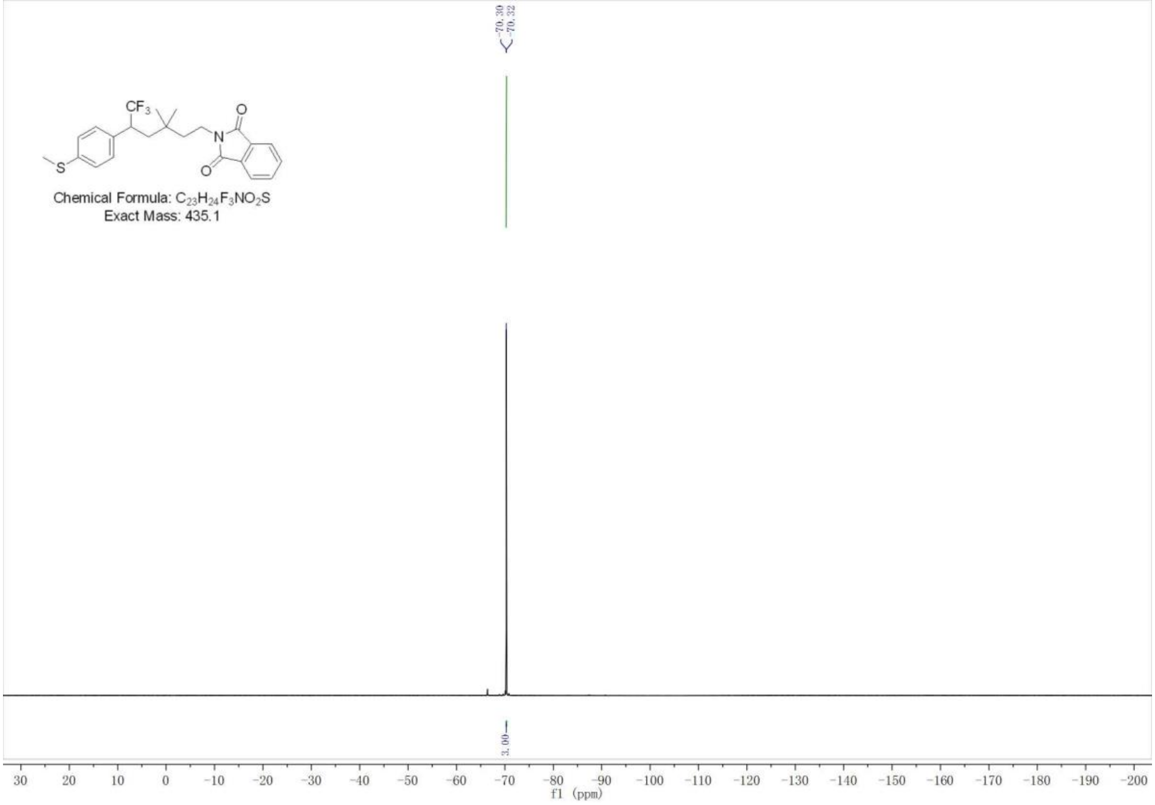

Supplementary Figure 52 ^{19}F NMR Spectrum of **4l**

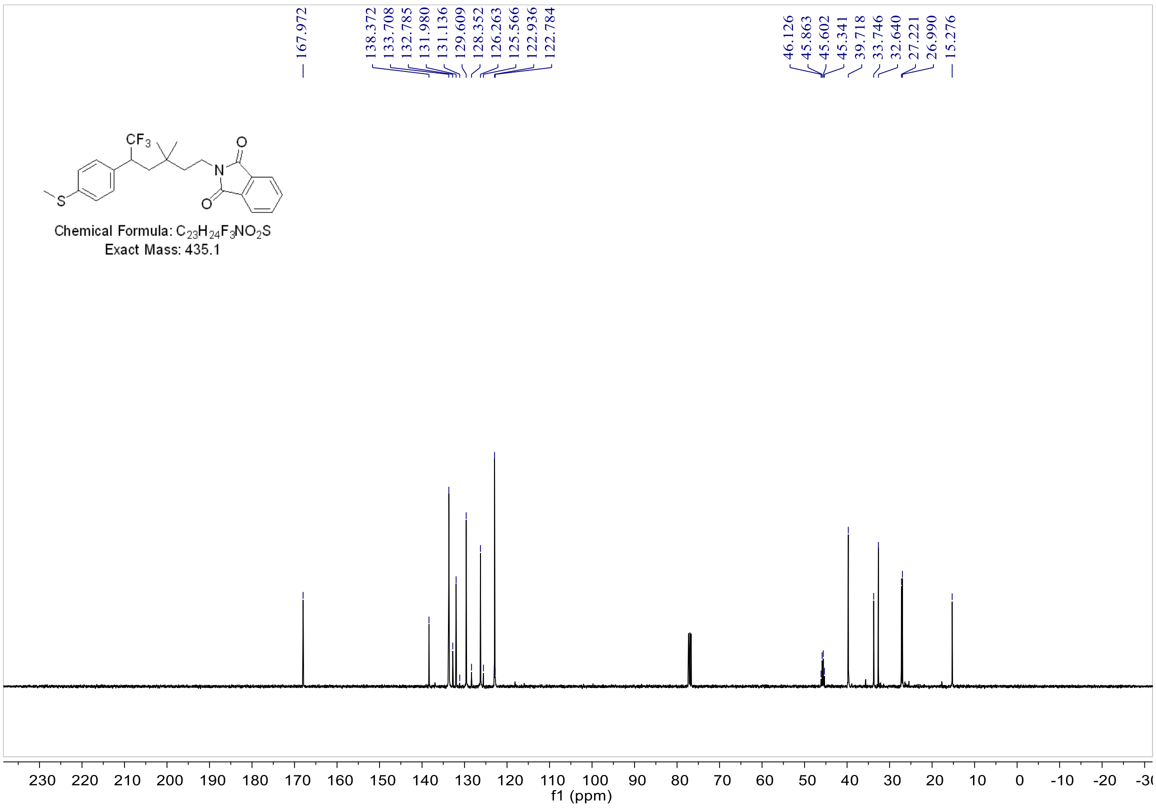

Supplementary Figure 53 ^{13}C NMR Spectrum of **4l**

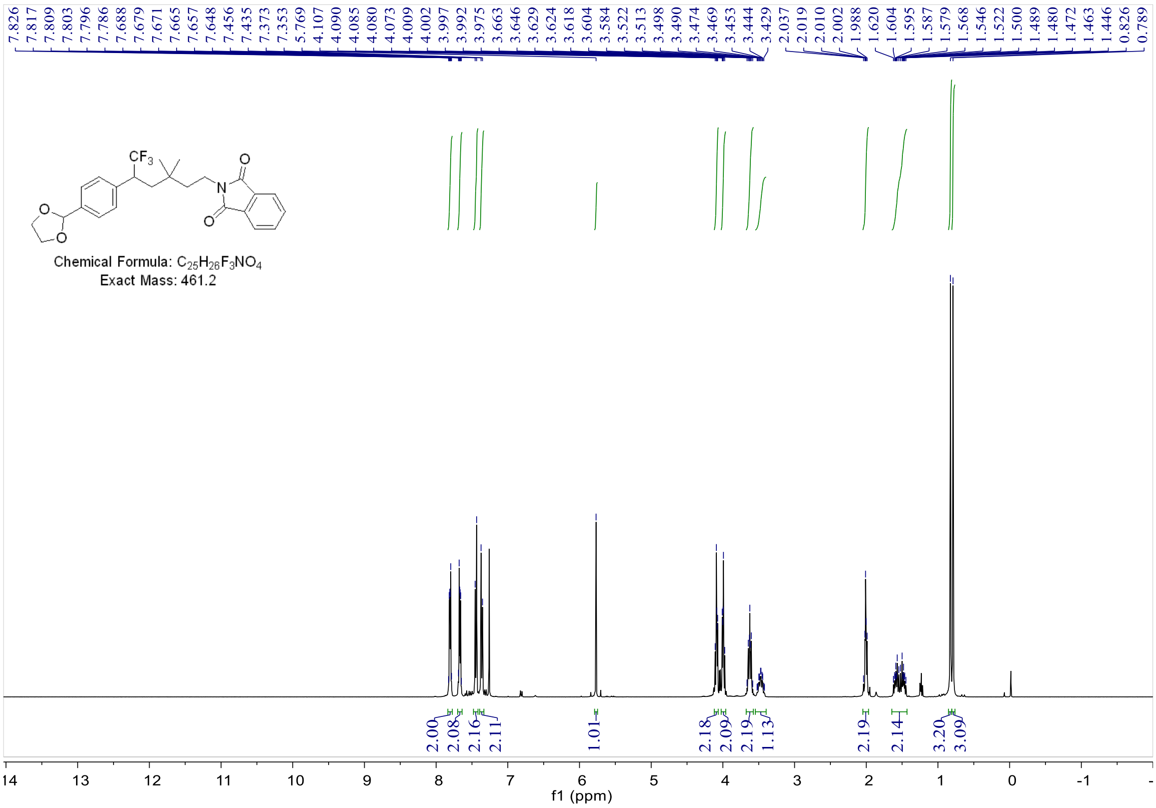

Supplementary Figure 54 ^{1}H NMR Spectrum of **4m**

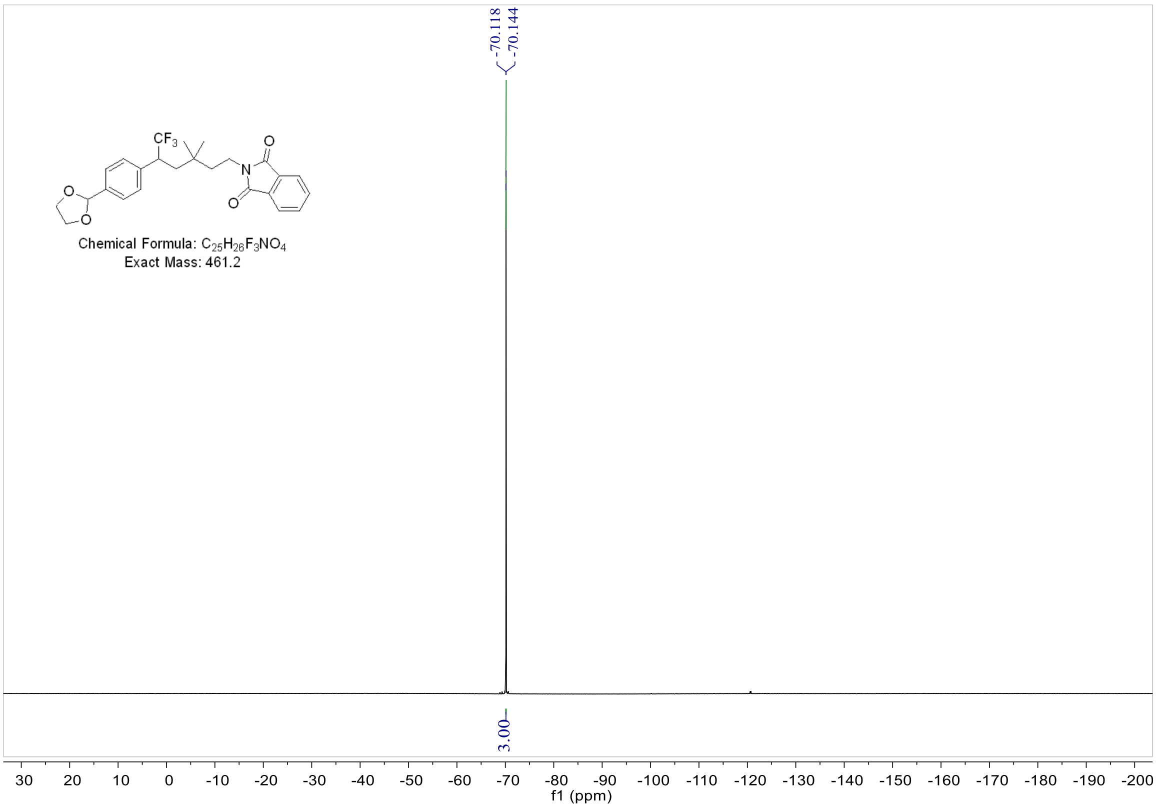

Supplementary Figure 55 ^{19}F NMR Spectrum of **4m**

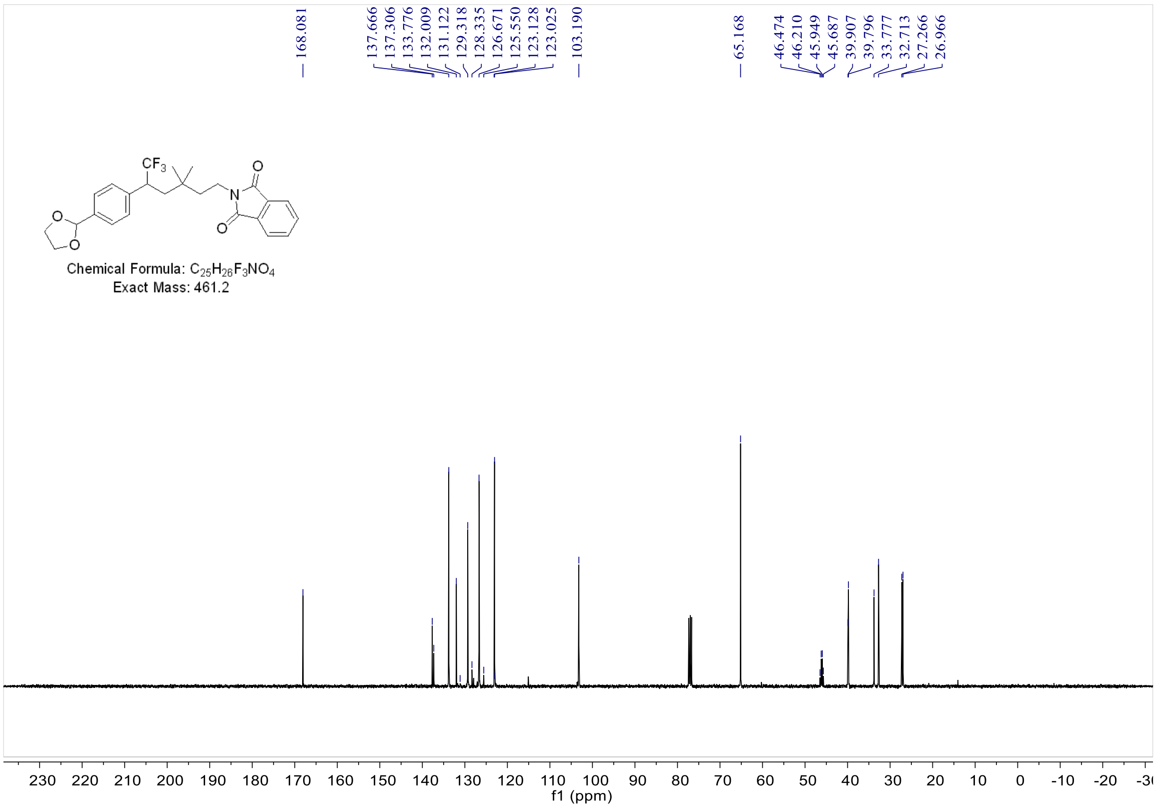

Supplementary Figure 56 ^{13}C NMR Spectrum of **4m**

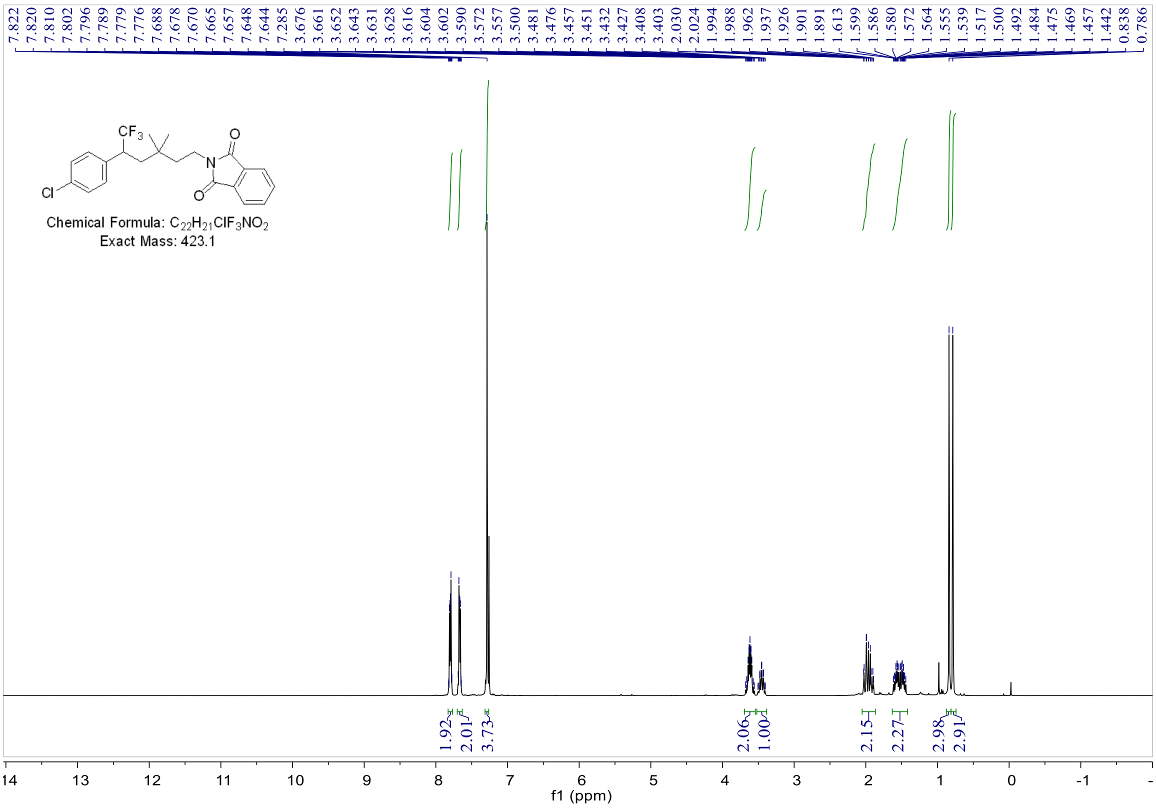

Supplementary Figure 57 ^{1}H NMR Spectrum of **4n**

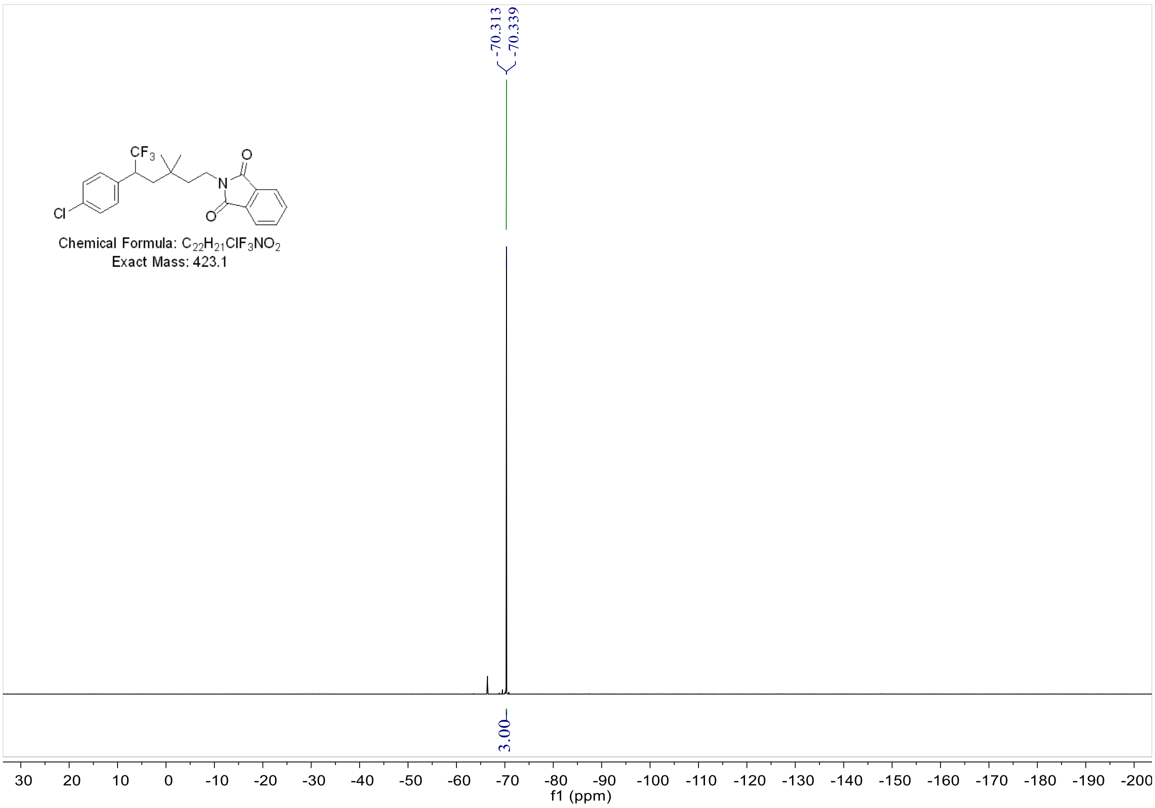

Supplementary Figure 58 ^{19}F NMR Spectrum of **4n**

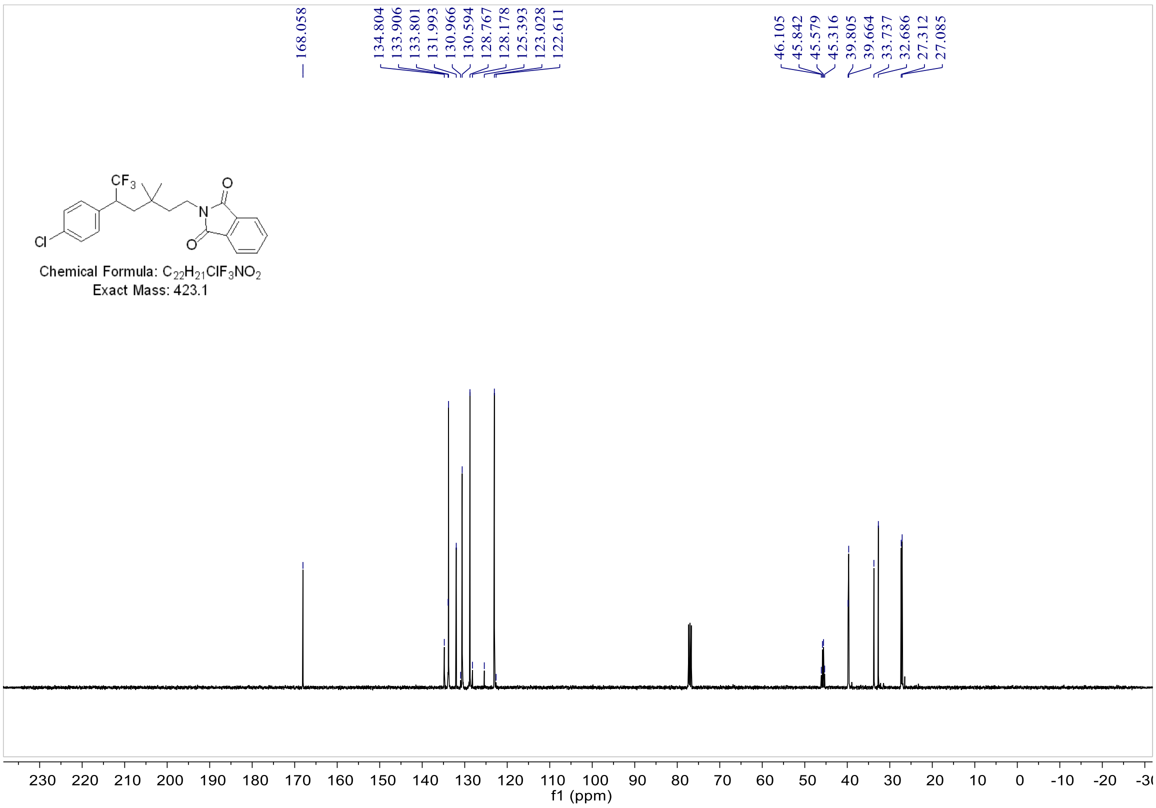

Supplementary Figure 59 ^{13}C NMR Spectrum of **4n**

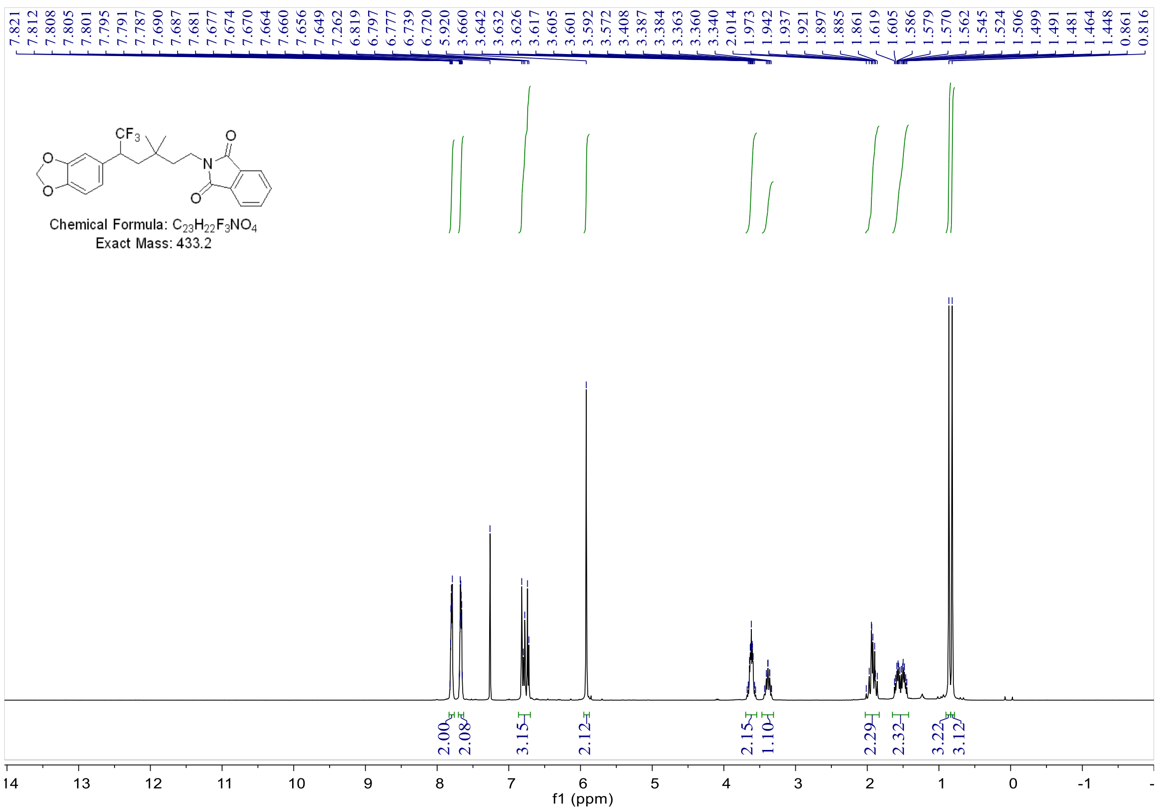

Supplementary Figure 60 ^{1}H NMR Spectrum of **4o**

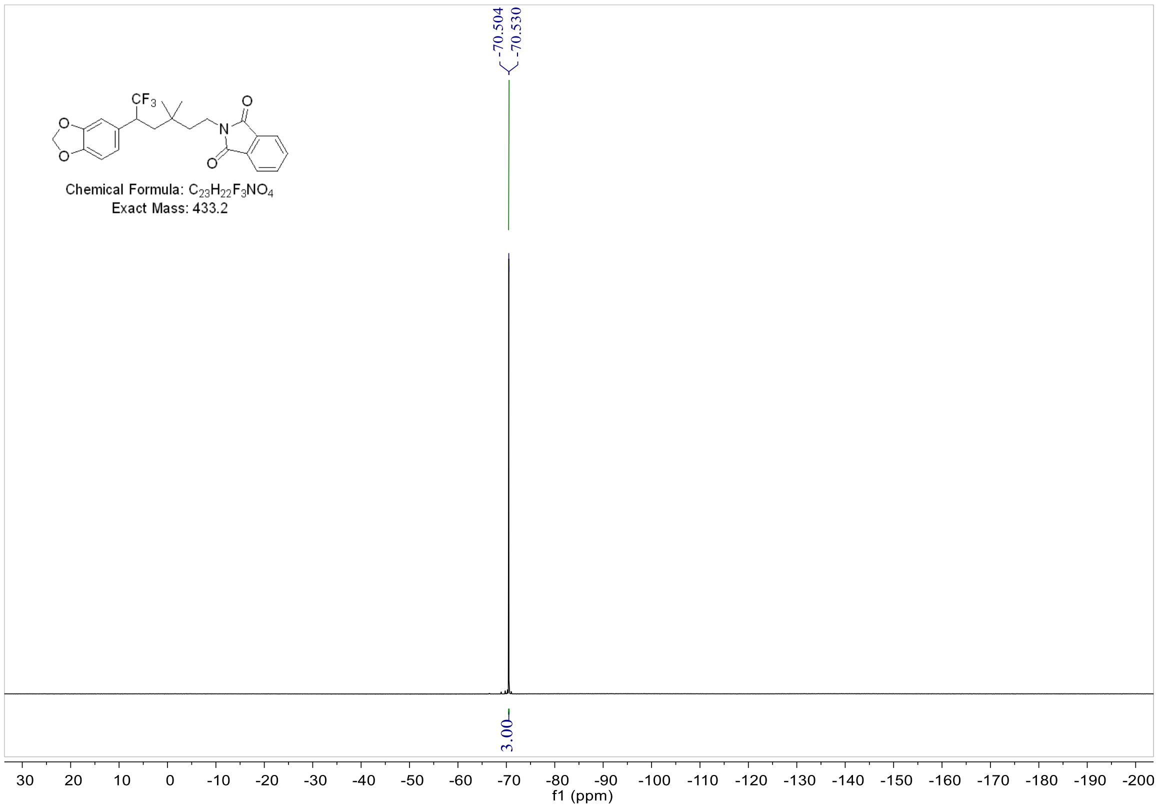

Supplementary Figure 61 ^{19}F NMR Spectrum of **4o**

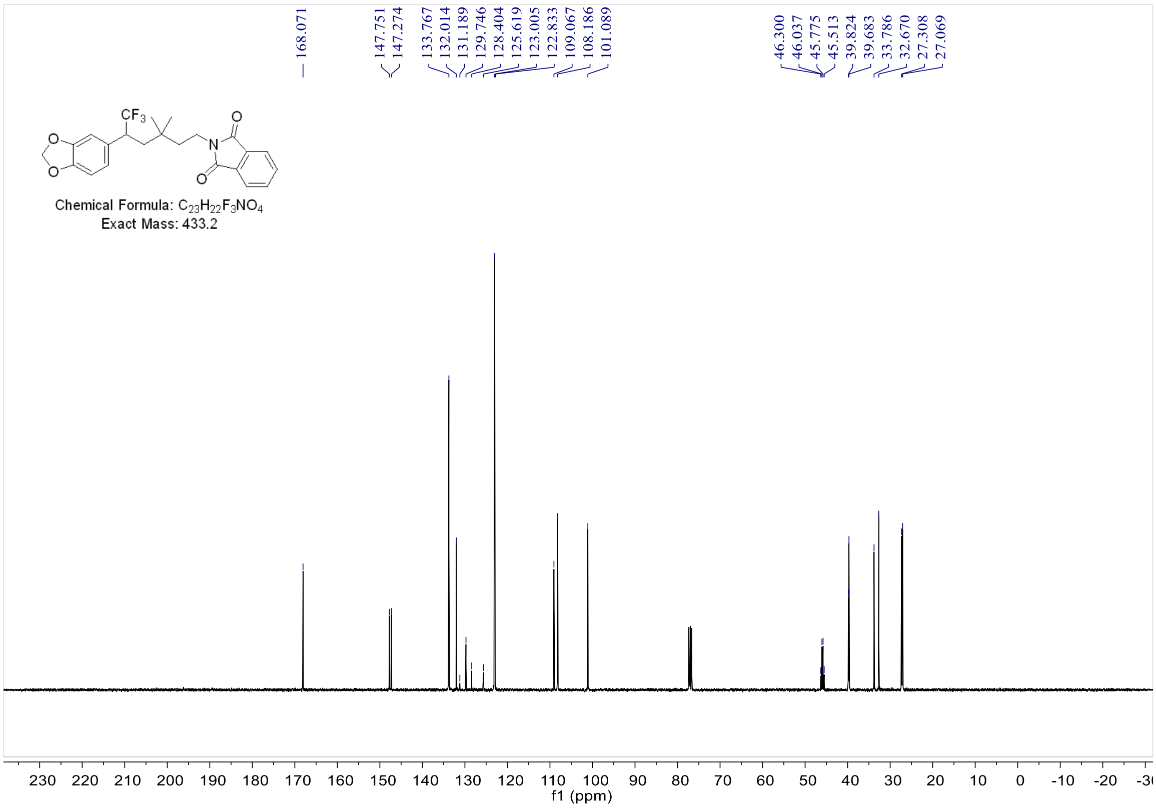

Supplementary Figure 62 ^{13}C NMR Spectrum of **4o**

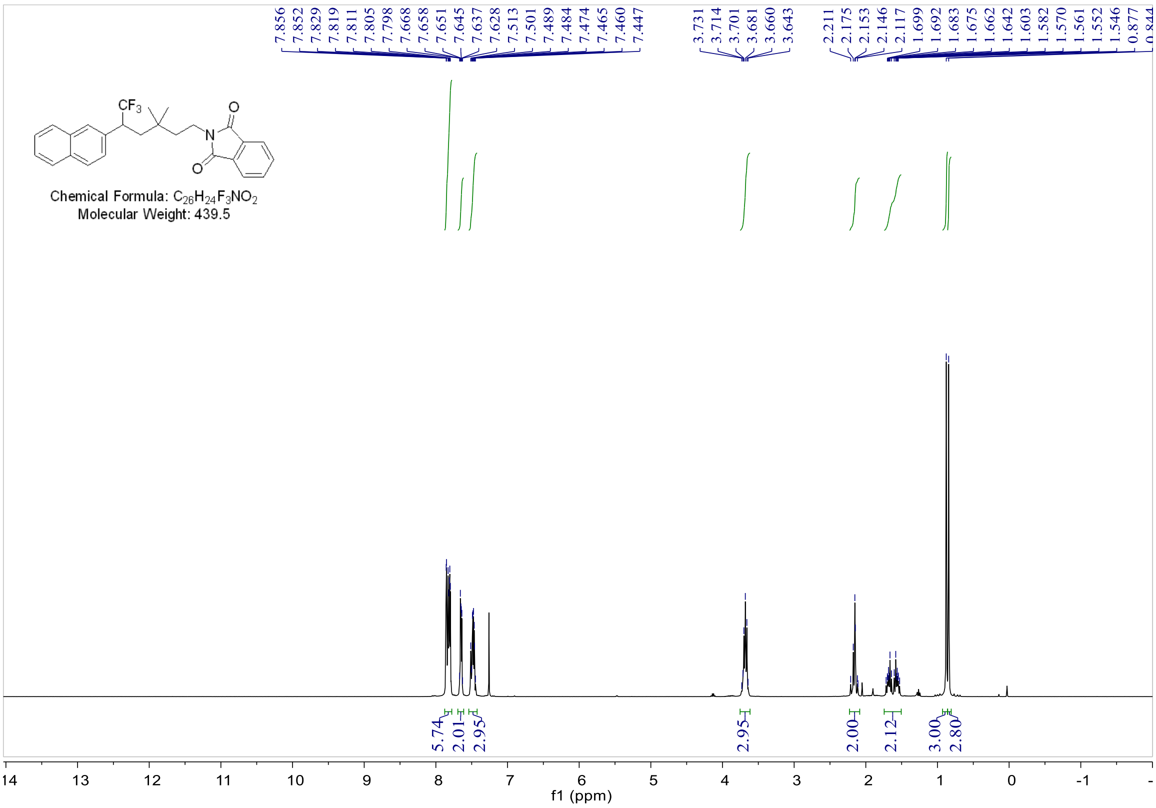

Supplementary Figure 63 ^{1}H NMR Spectrum of **4p**

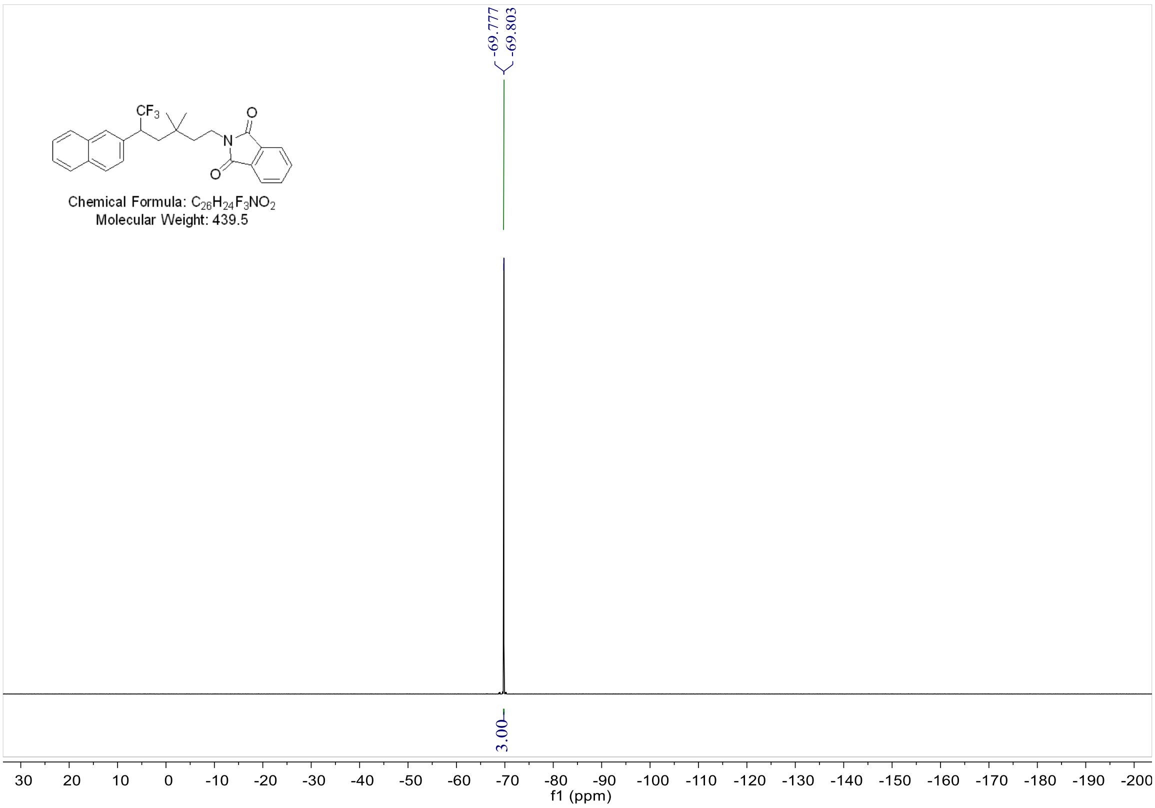

Supplementary Figure 64 ^{19}F NMR Spectrum of **4p**

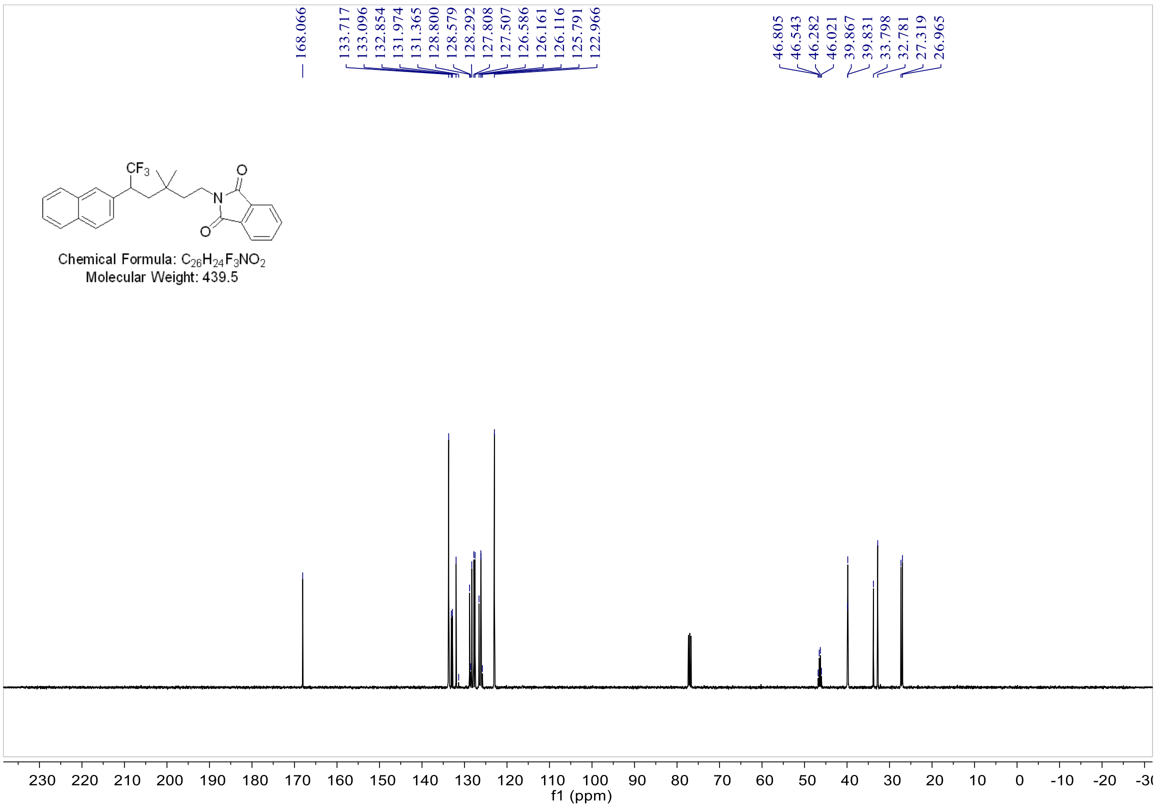

Supplementary Figure 65 ^{13}C NMR Spectrum of **4p**

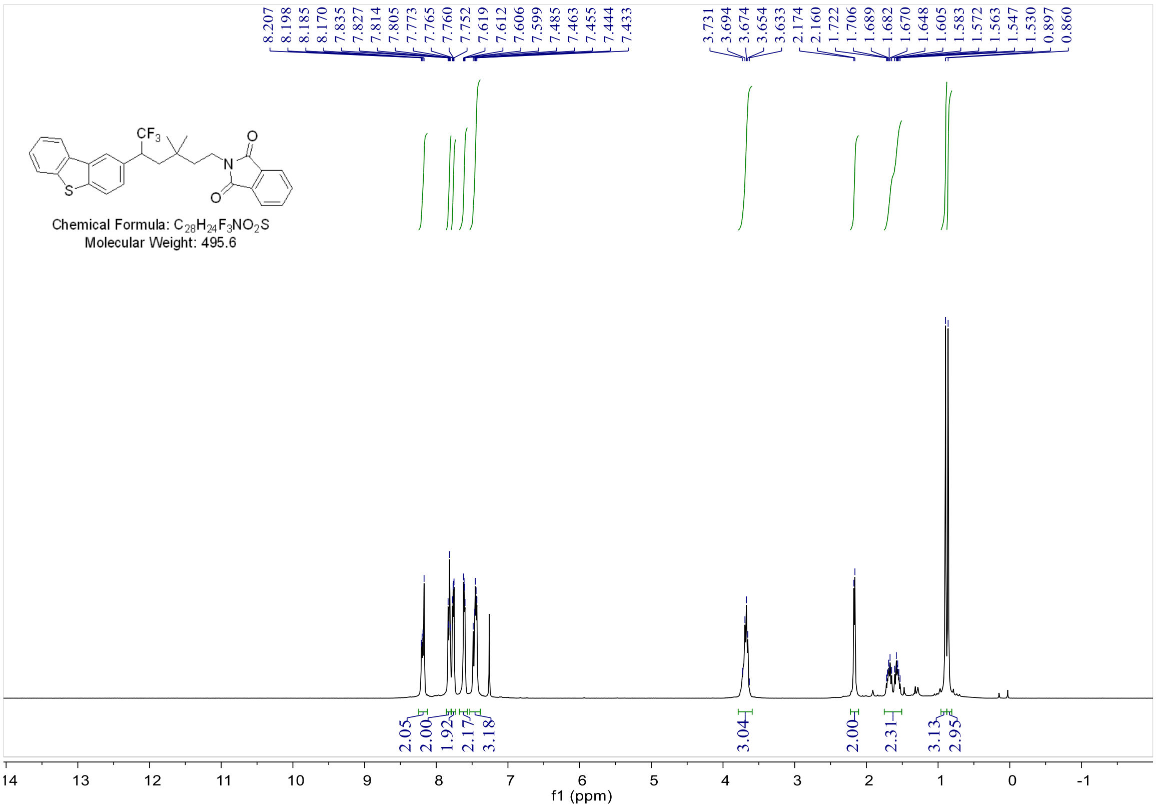

Supplementary Figure 66 ^{1}H NMR Spectrum of **4q**

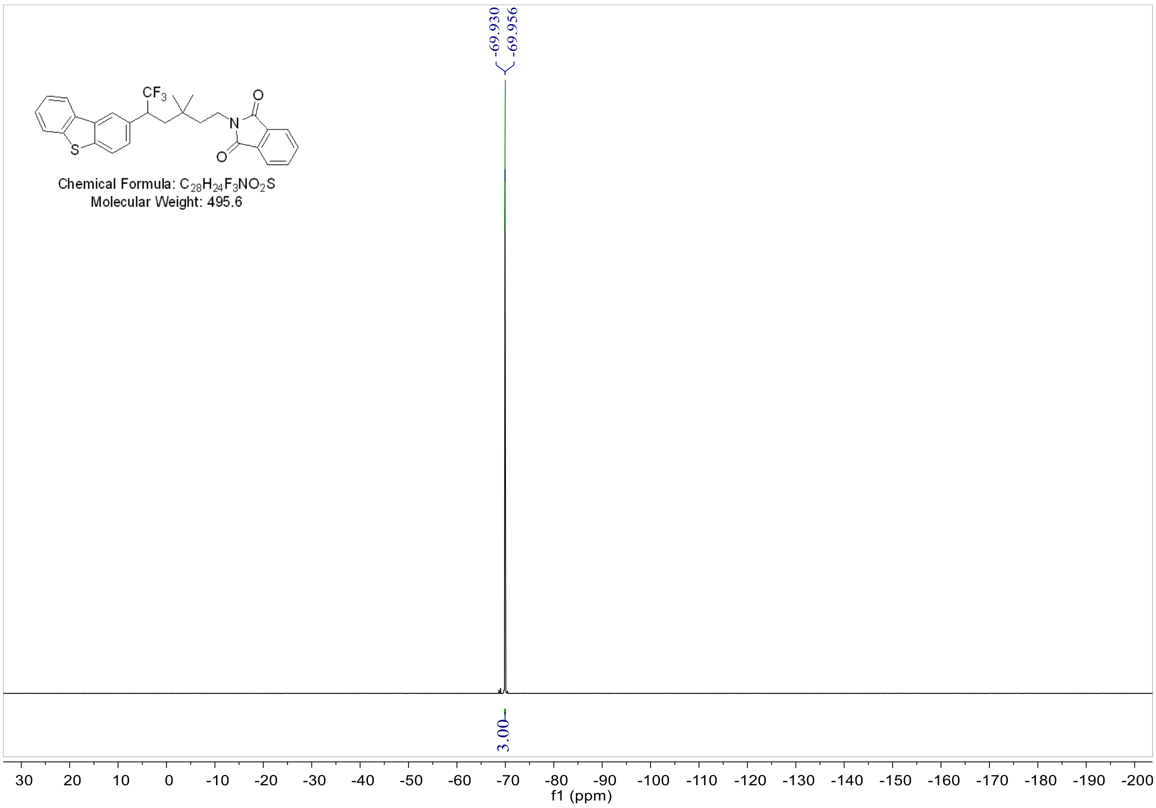

Supplementary Figure 67 ^{19}F NMR Spectrum of **4q**

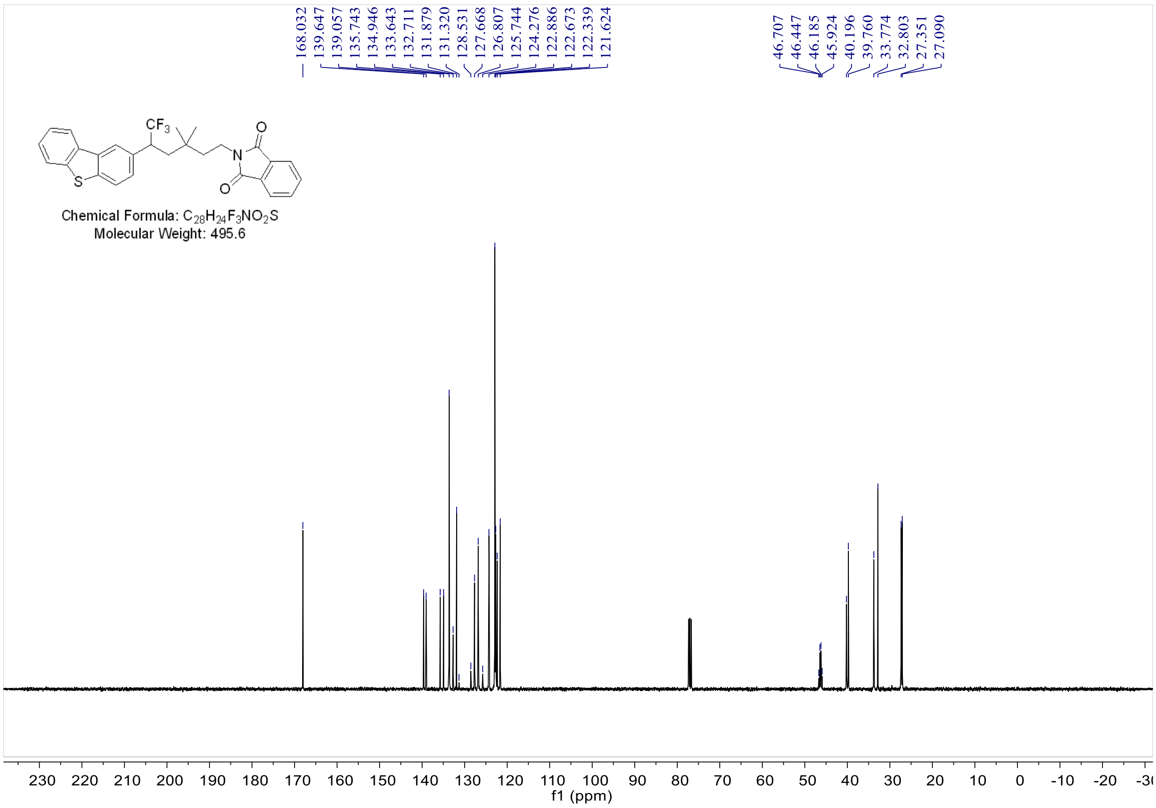

Supplementary Figure 68 ^{13}C NMR Spectrum of **4q**

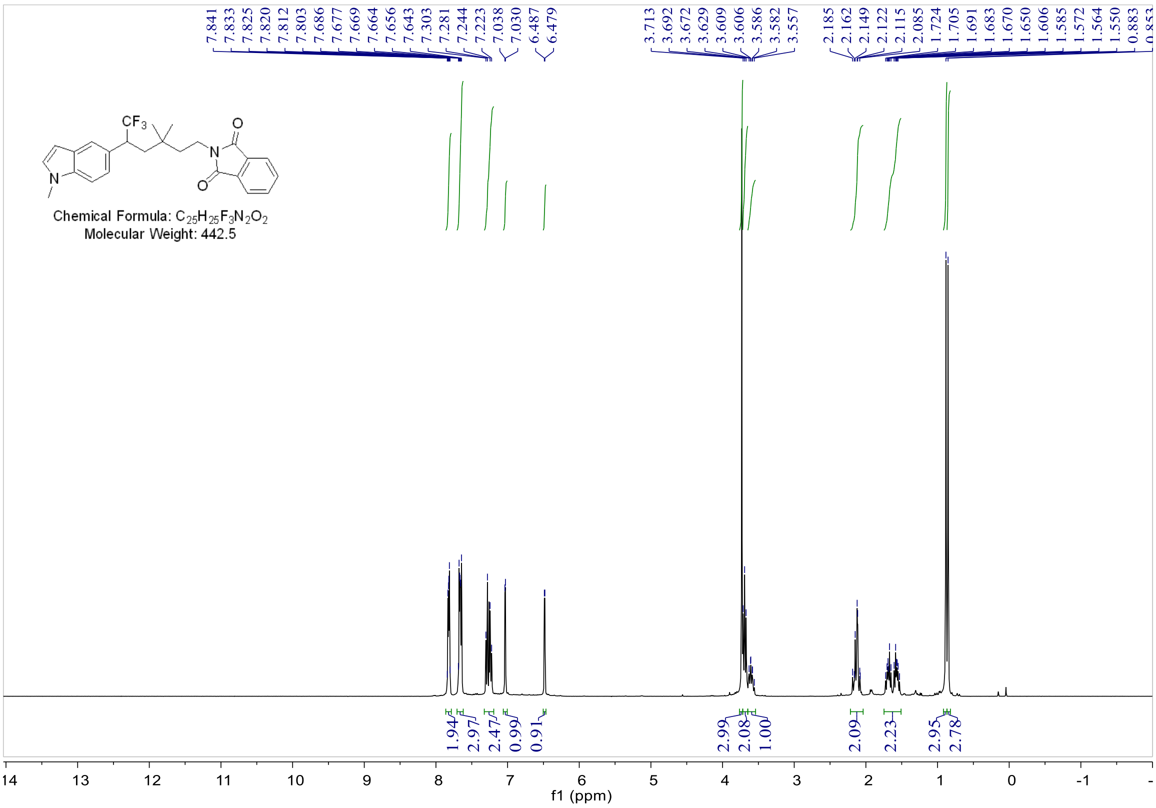

Supplementary Figure 69 ^{1}H NMR Spectrum of **4r**

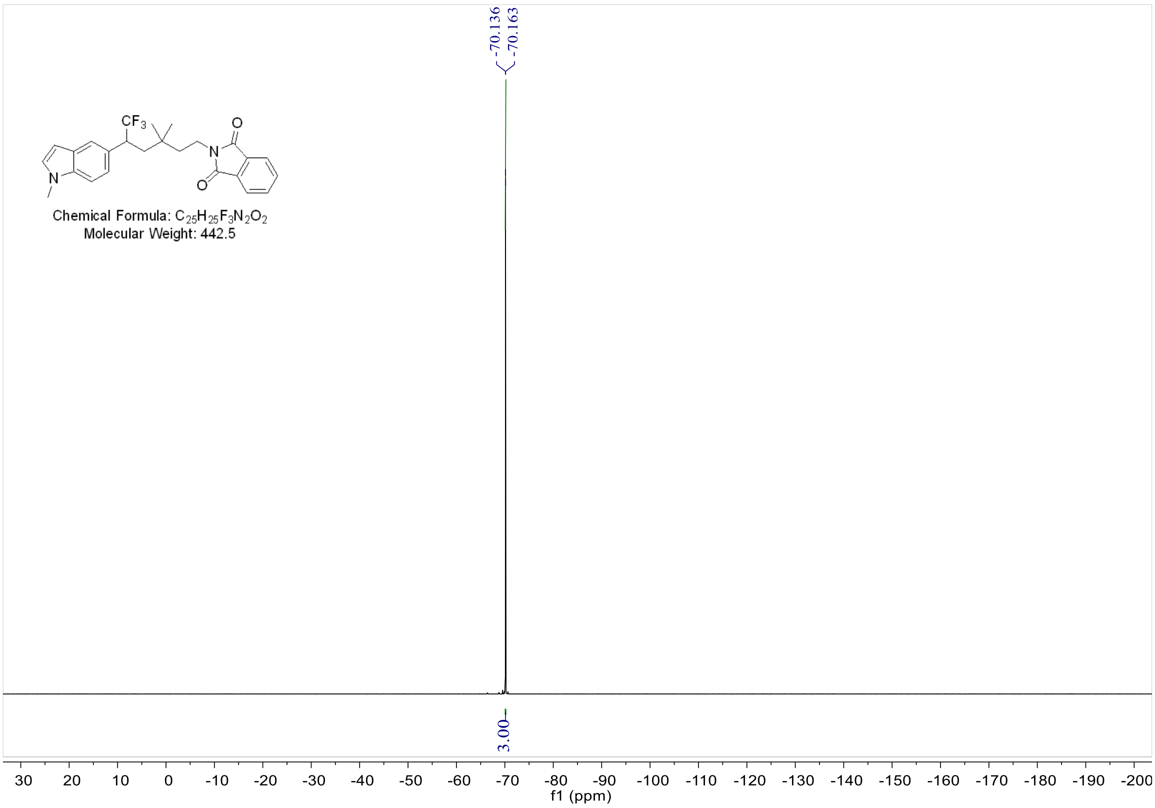

Supplementary Figure 70 ^{19}F NMR Spectrum of **4r**

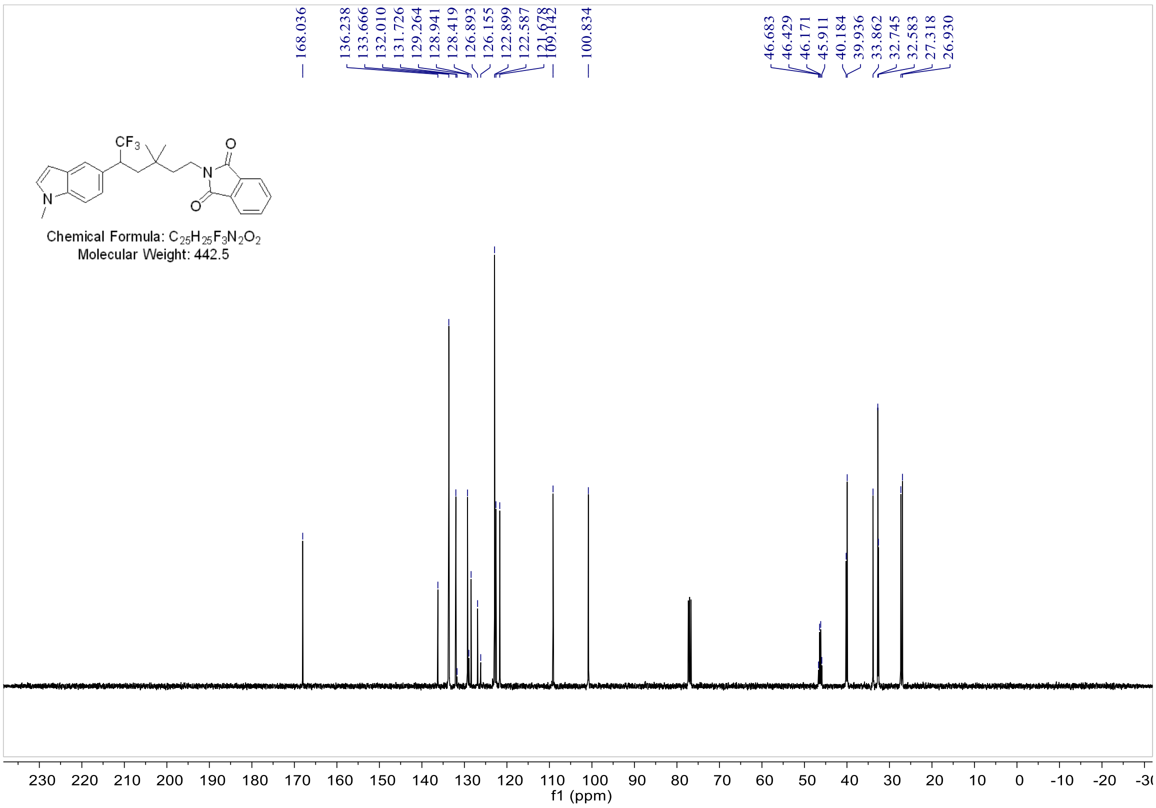

Supplementary Figure 71 ^{13}C NMR Spectrum of **4r**

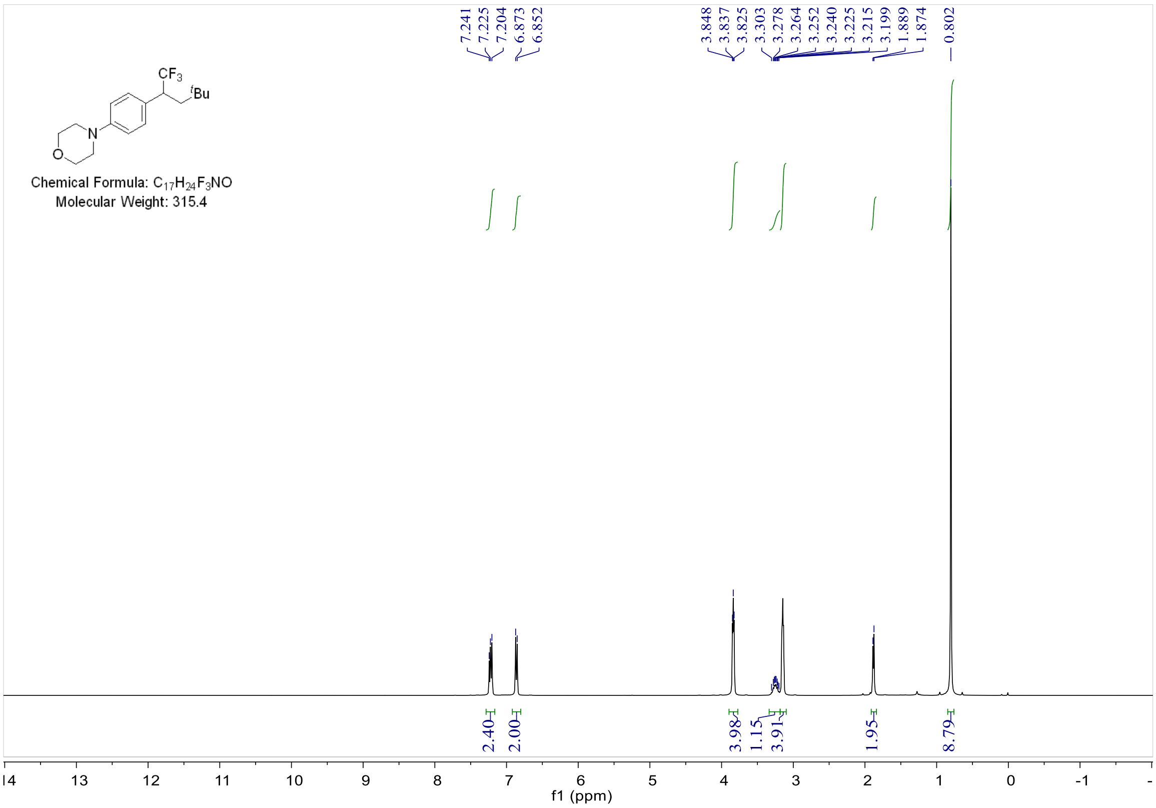

Supplementary Figure 72 ^{1}H NMR Spectrum of **5a**

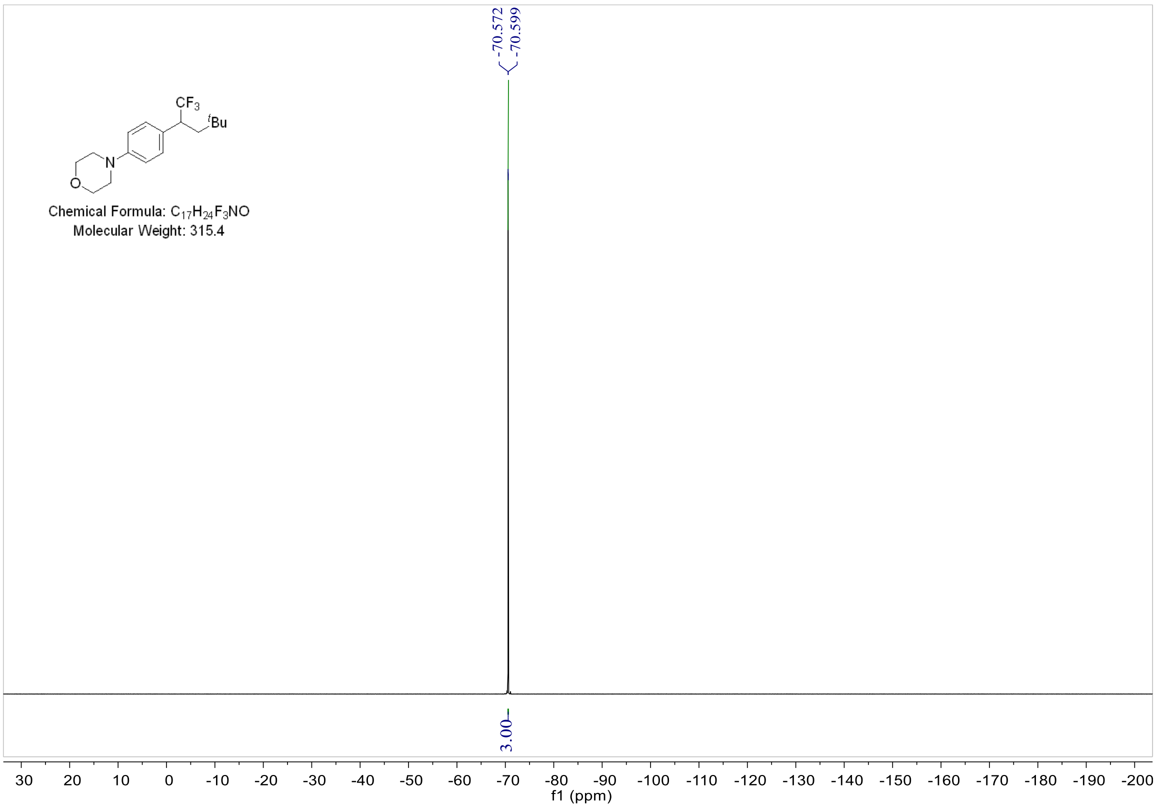

Supplementary Figure 73 ^{19}F NMR Spectrum of **5a**

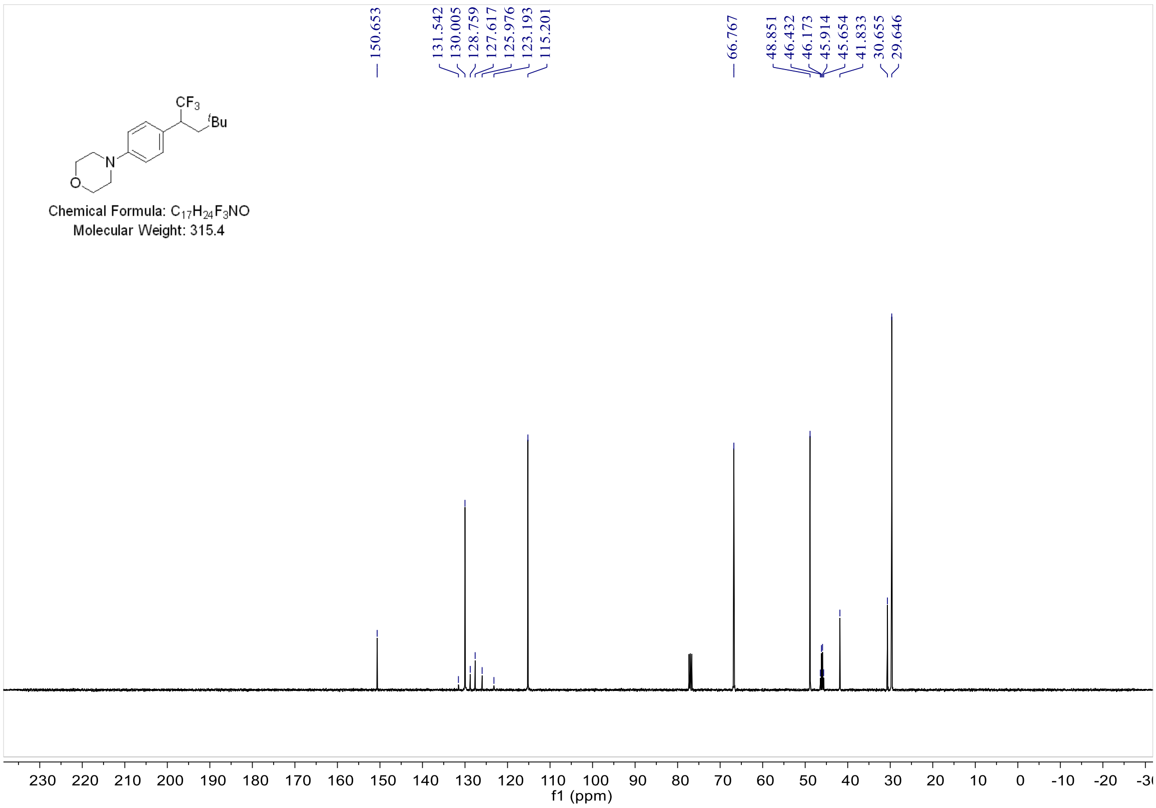

Supplementary Figure 74 ^{13}C NMR Spectrum of **5a**

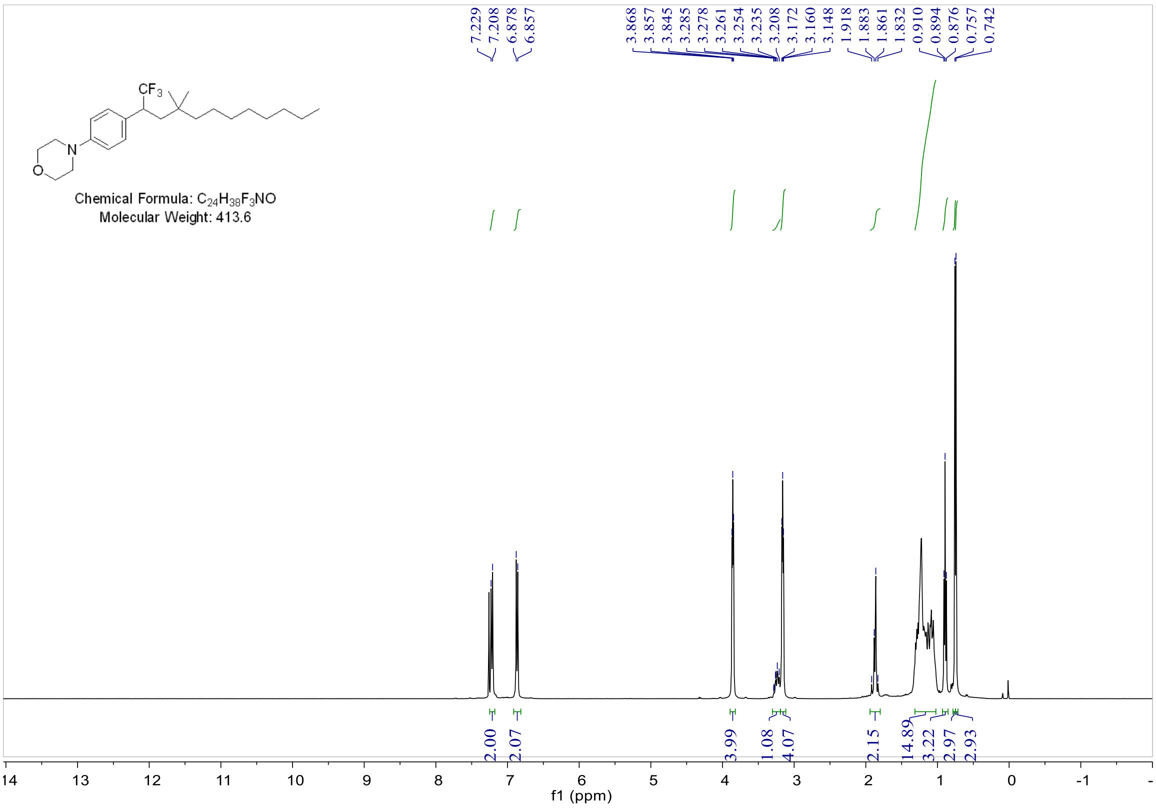

Supplementary Figure 75 ^{1}H NMR Spectrum of **5b**

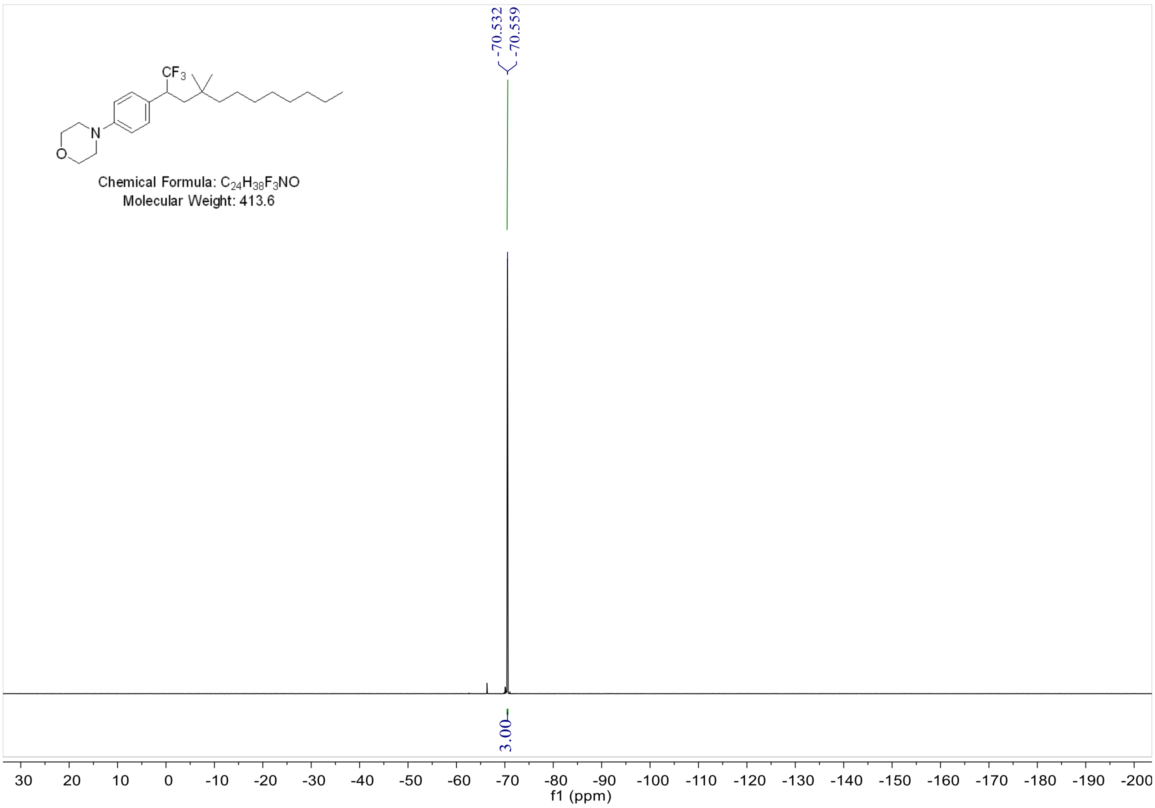

Supplementary Figure 76 ^{19}F NMR Spectrum of **5b**

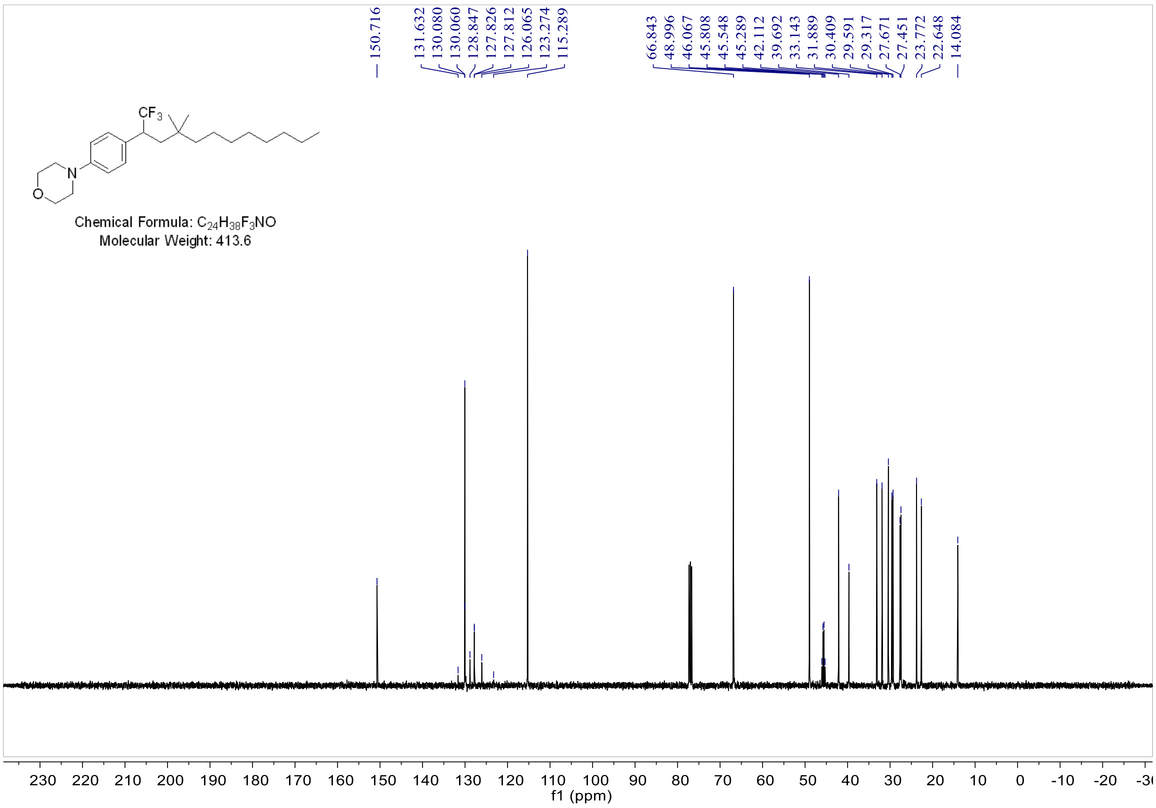

Supplementary Figure 77 ^{13}C NMR Spectrum of **5b**

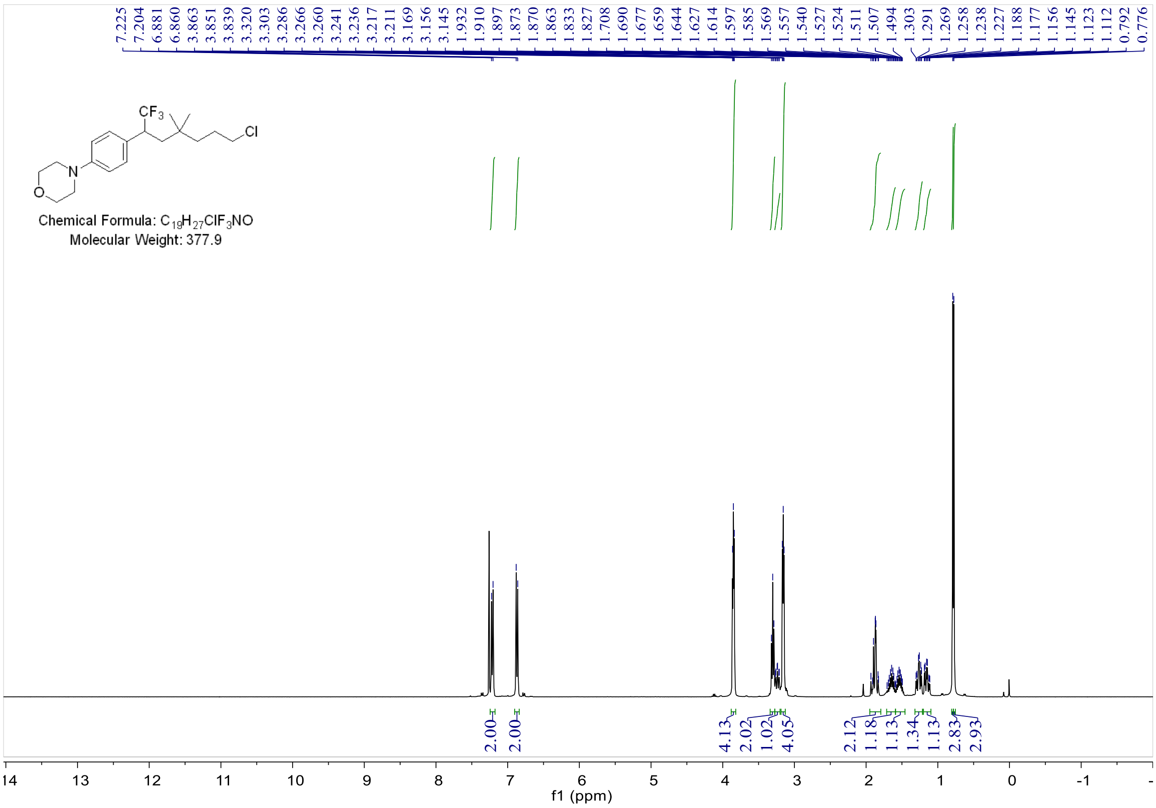

Supplementary Figure 78 ^{1}H NMR Spectrum of **5c'**

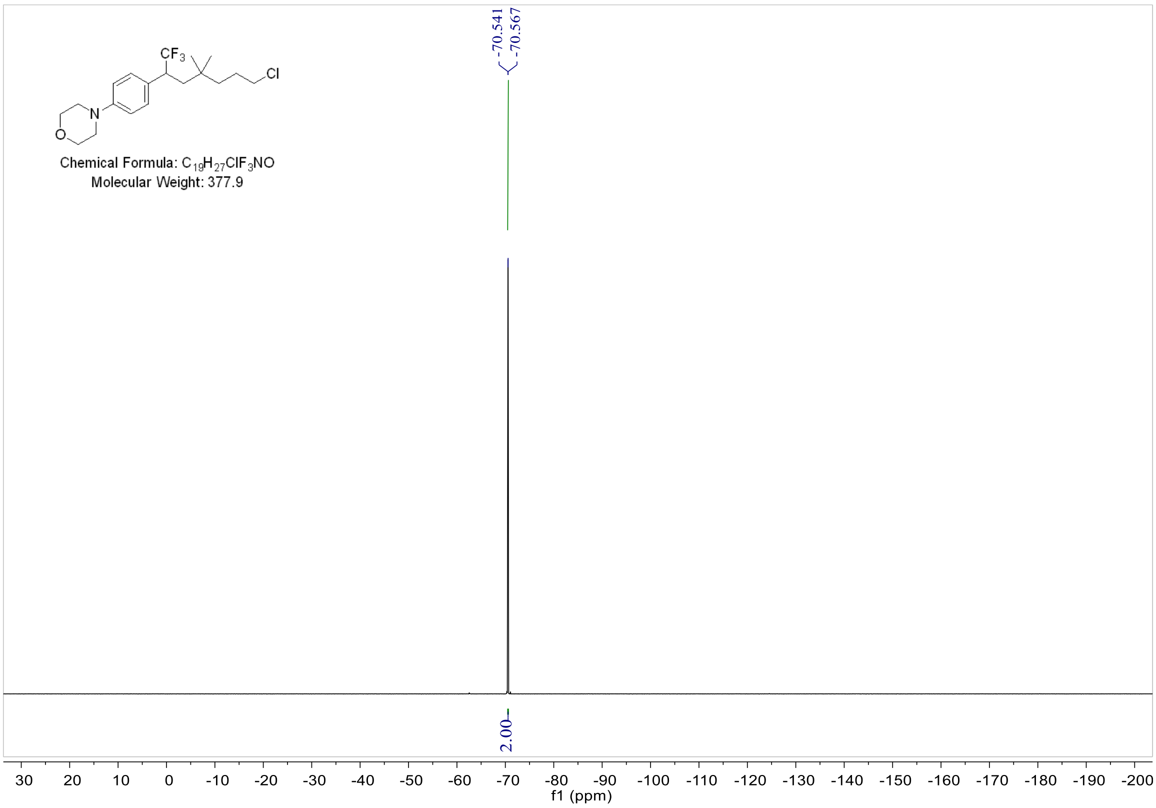

Supplementary Figure 79 ^{19}F NMR Spectrum of **5c'**

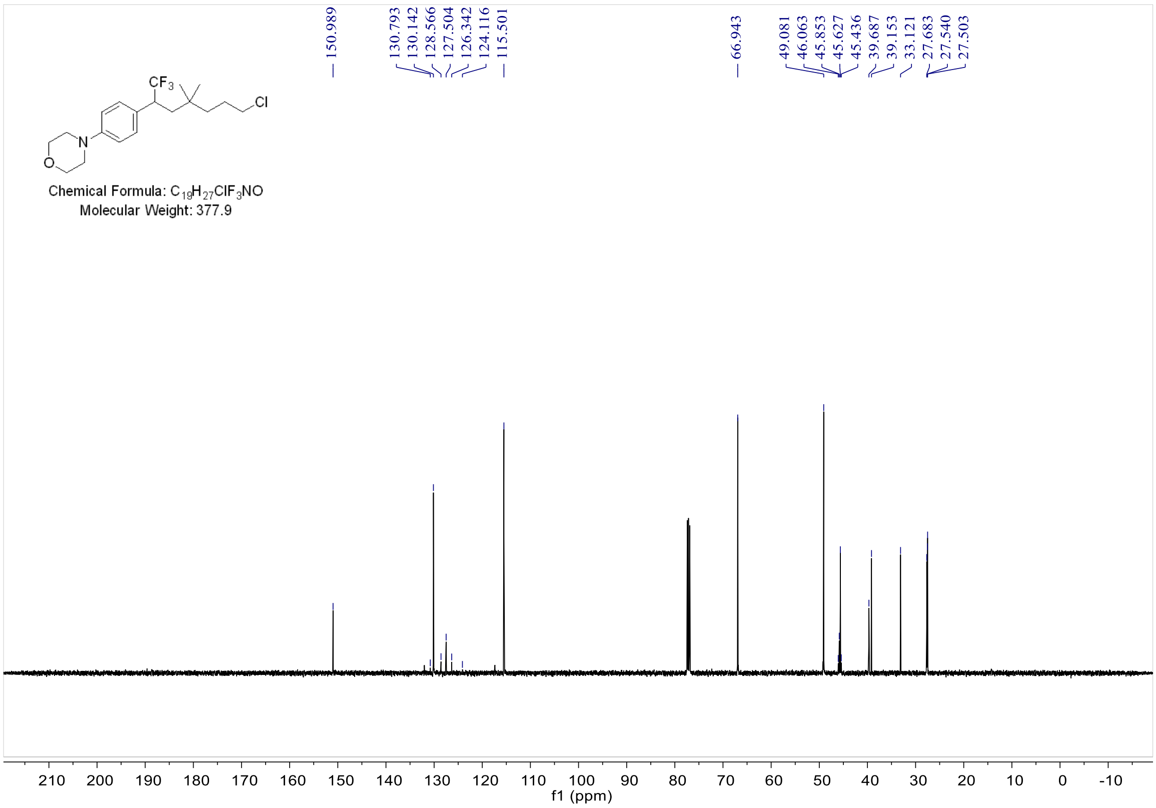

Supplementary Figure 80 ^{13}C NMR Spectrum of **5c'**

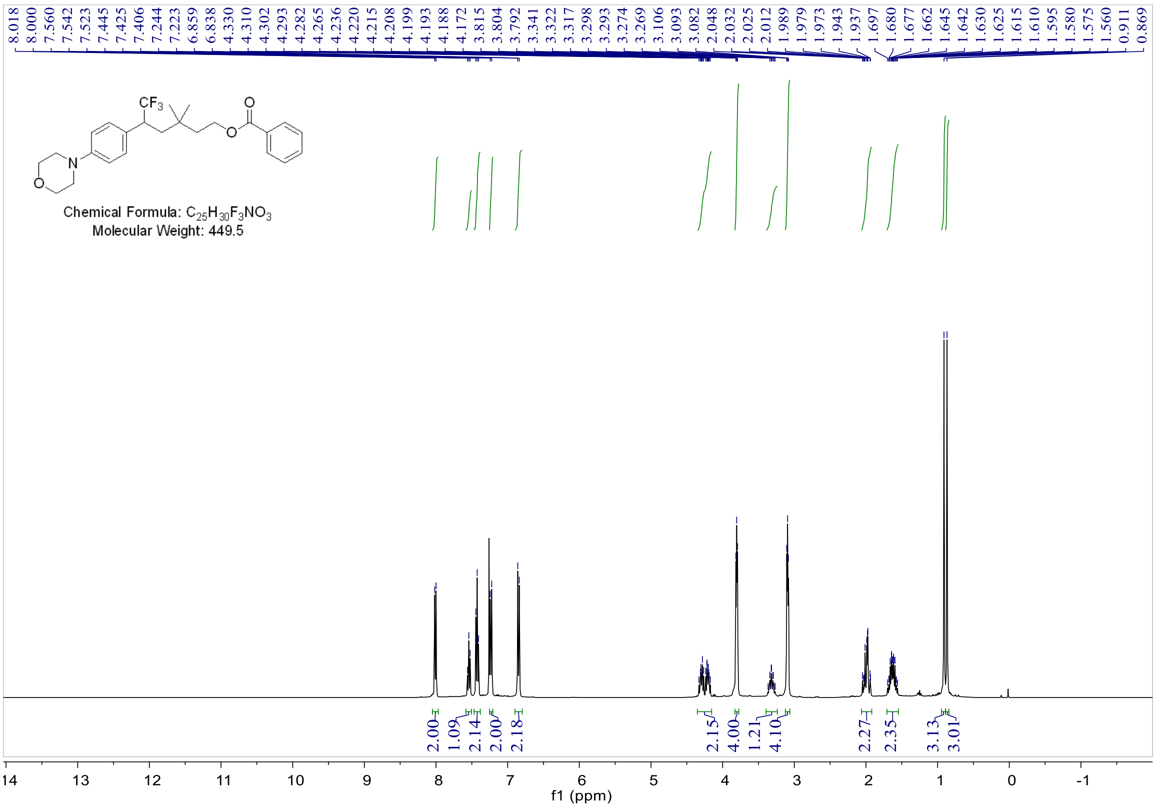

Supplementary Figure 81 ^{1}H NMR Spectrum of **5d**

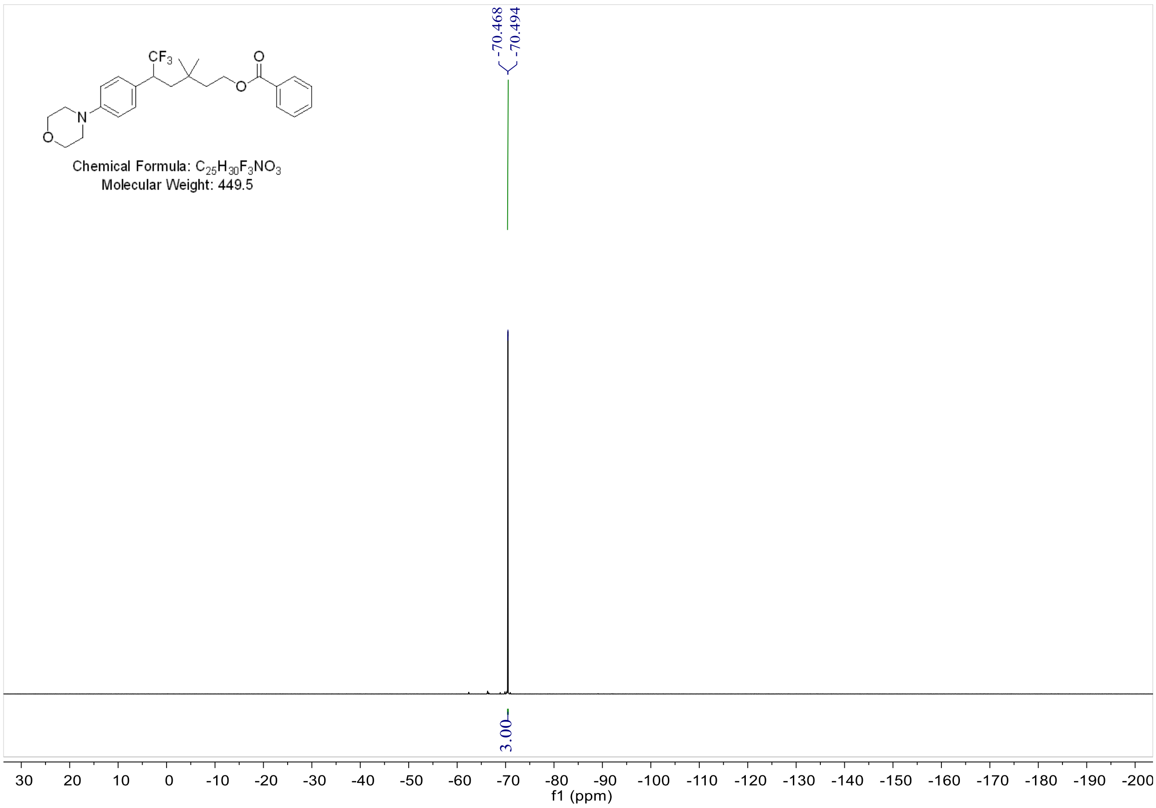

Supplementary Figure 82 ^{19}F NMR Spectrum of **5d**

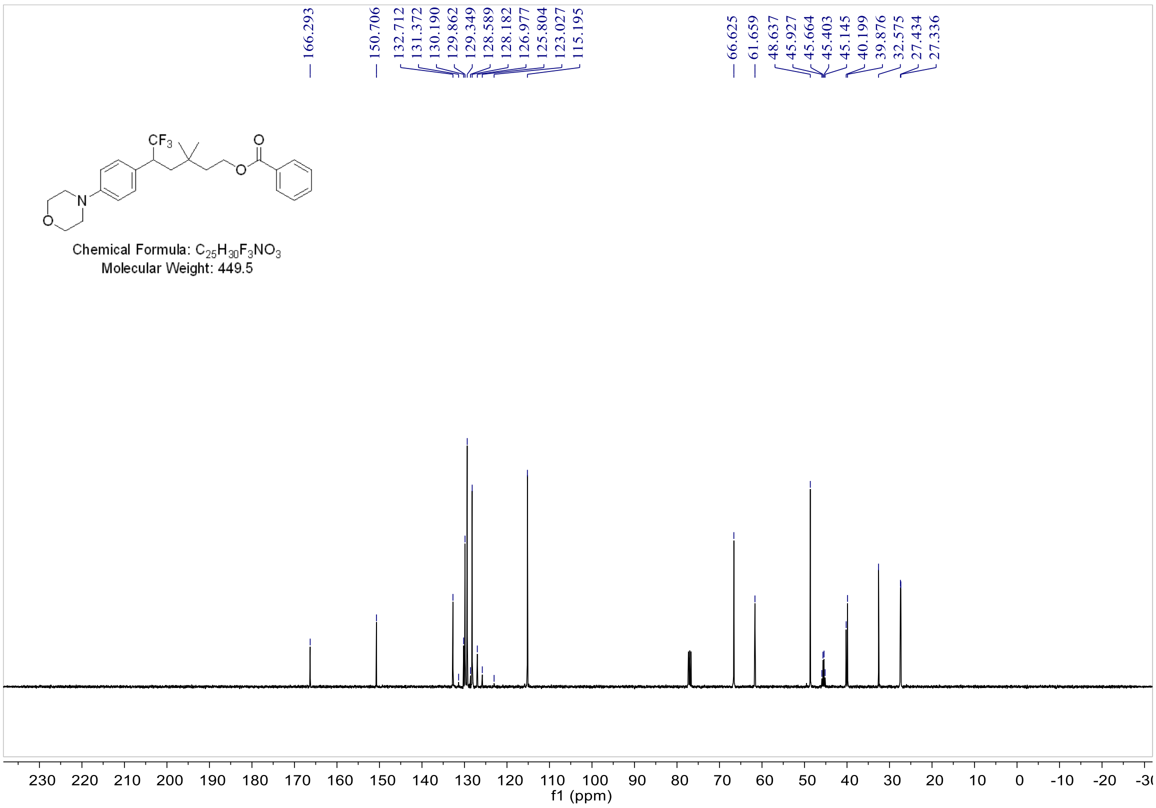

Supplementary Figure 83 ^{13}C NMR Spectrum of **5d**

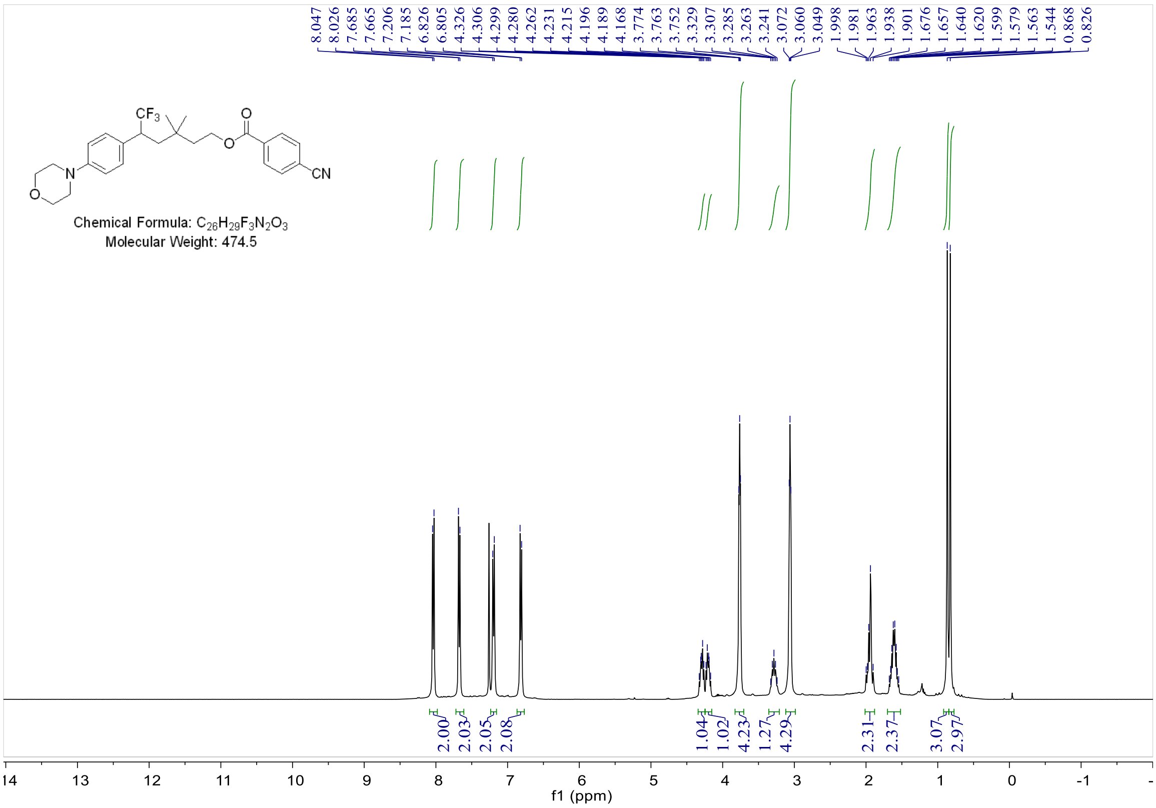

Supplementary Figure 84 ^{1}H NMR Spectrum of **5e**

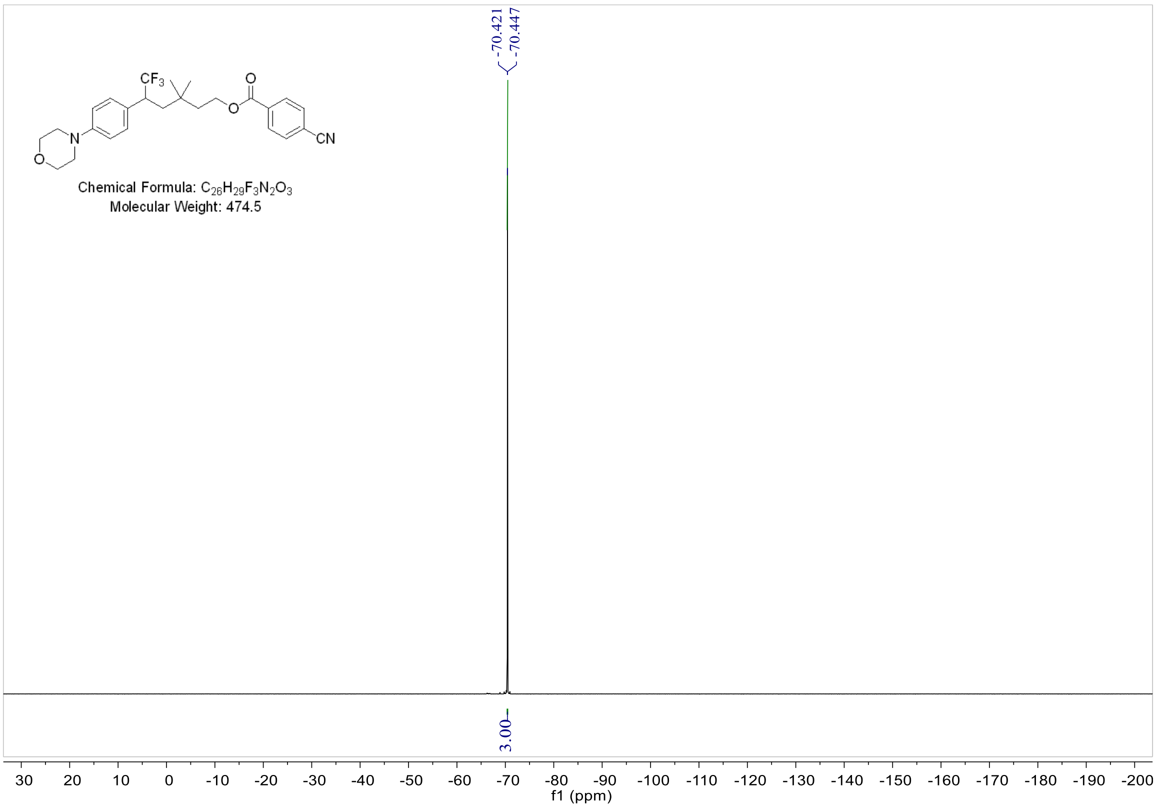

Supplementary Figure 85 ^{19}F NMR Spectrum of **5e**

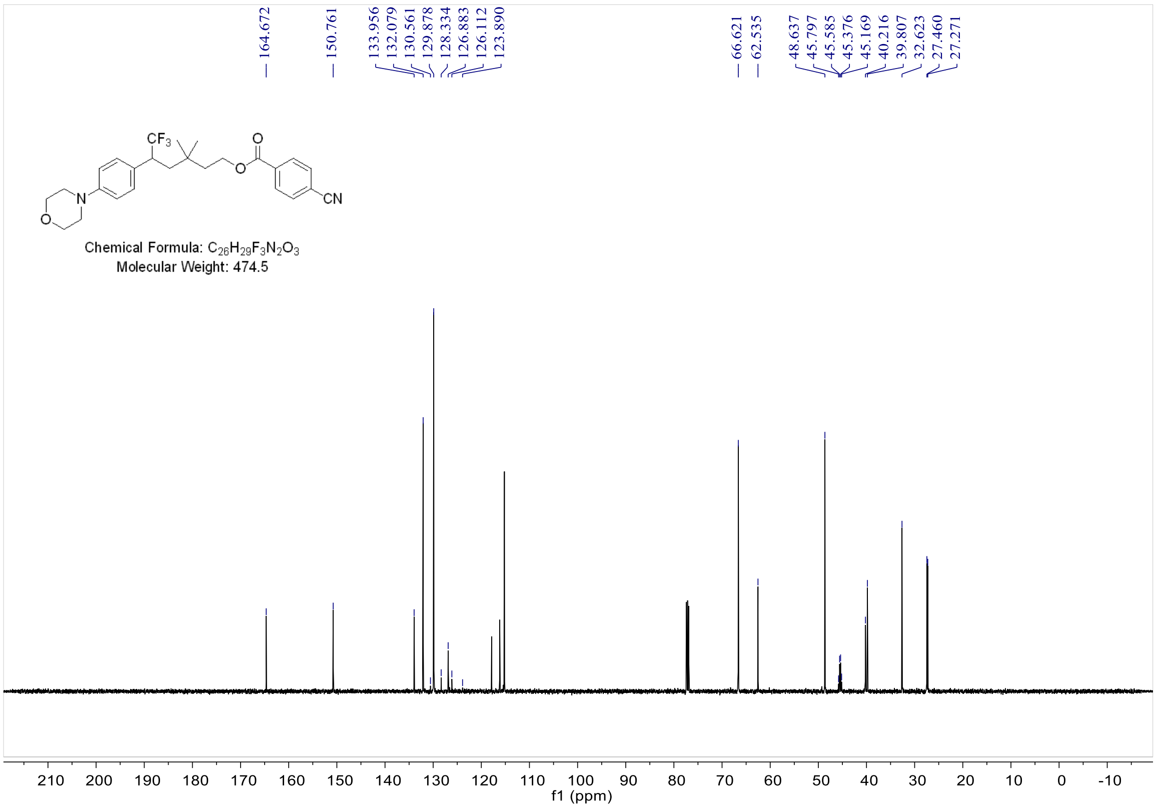

Supplementary Figure 86 ^{13}C NMR Spectrum of **5e**

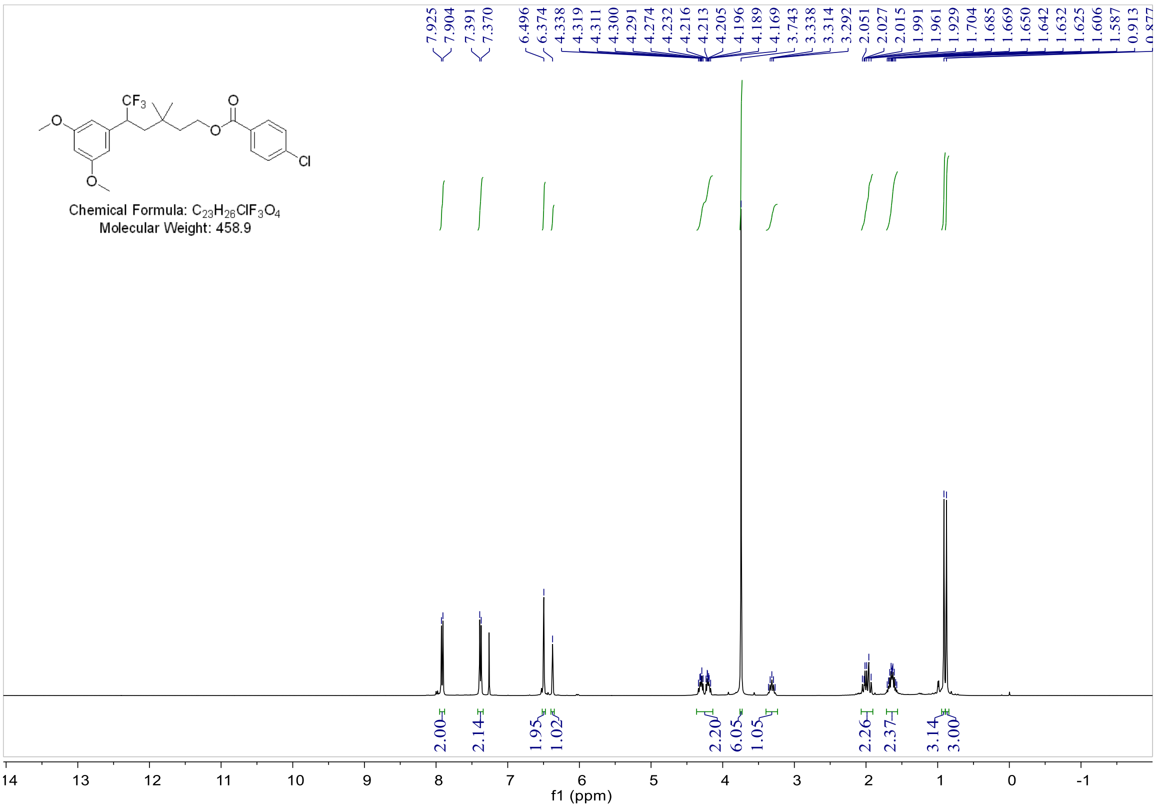

Supplementary Figure 87 ^{1}H NMR Spectrum of **5f**

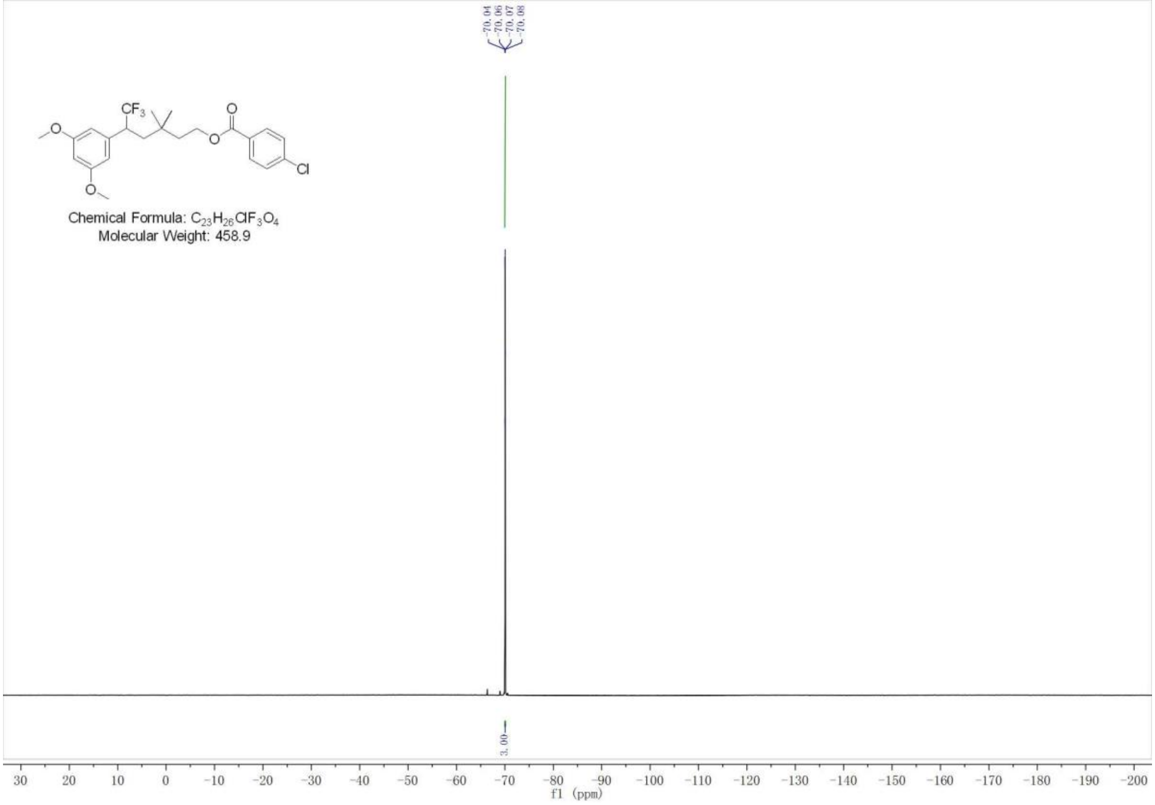

Supplementary Figure 88 ^{19}F NMR Spectrum of **5f**

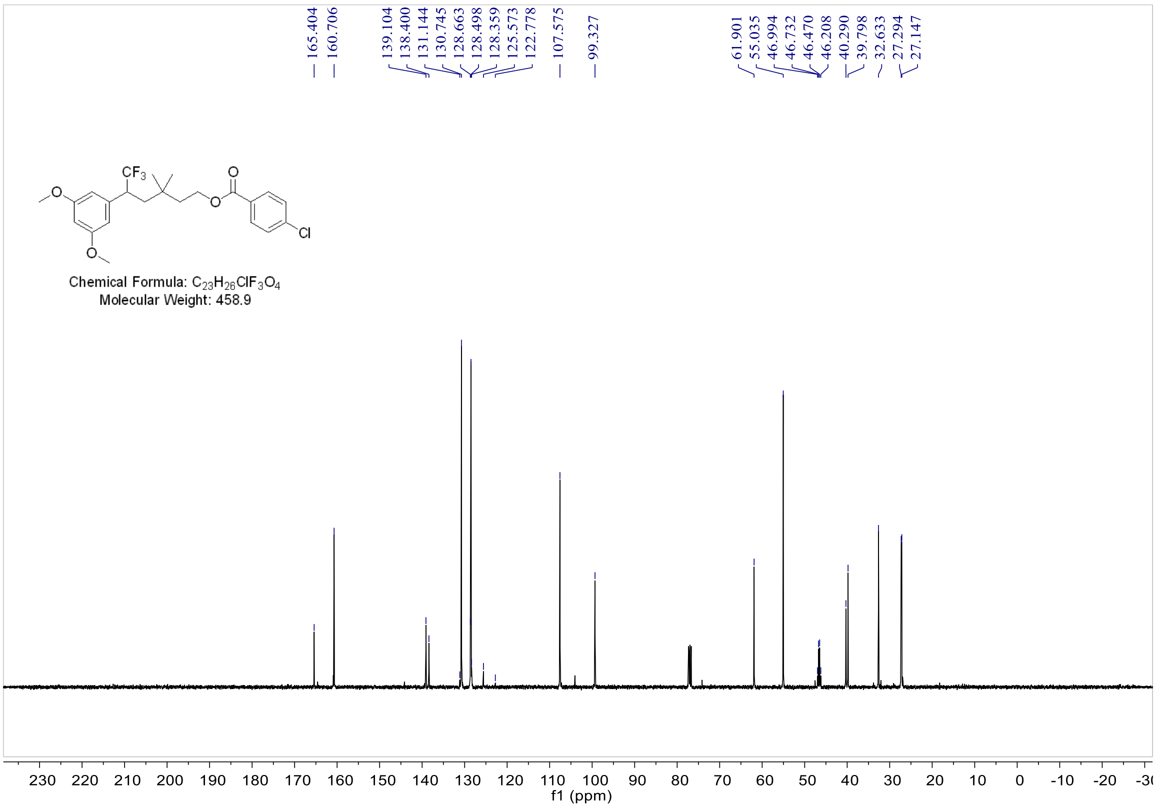

Supplementary Figure 89 ^{13}C NMR Spectrum of **5f**

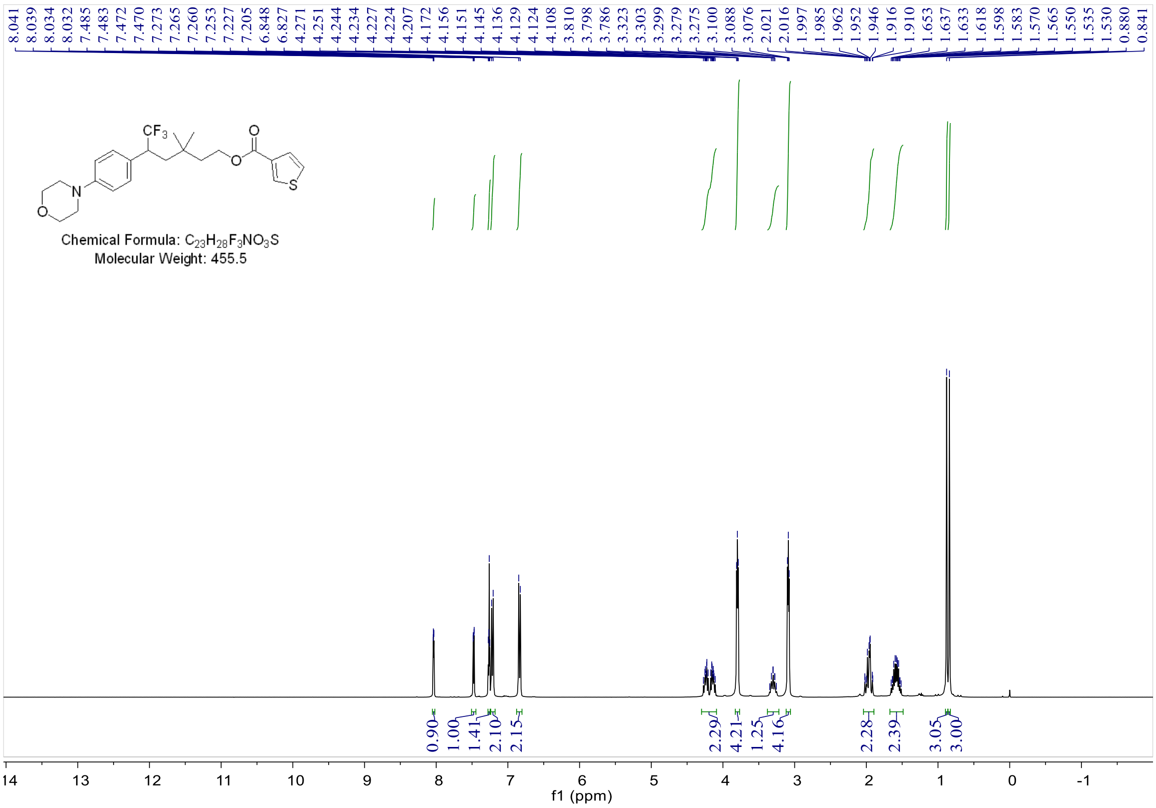

Supplementary Figure 90 ^{1}H NMR Spectrum of **5g**

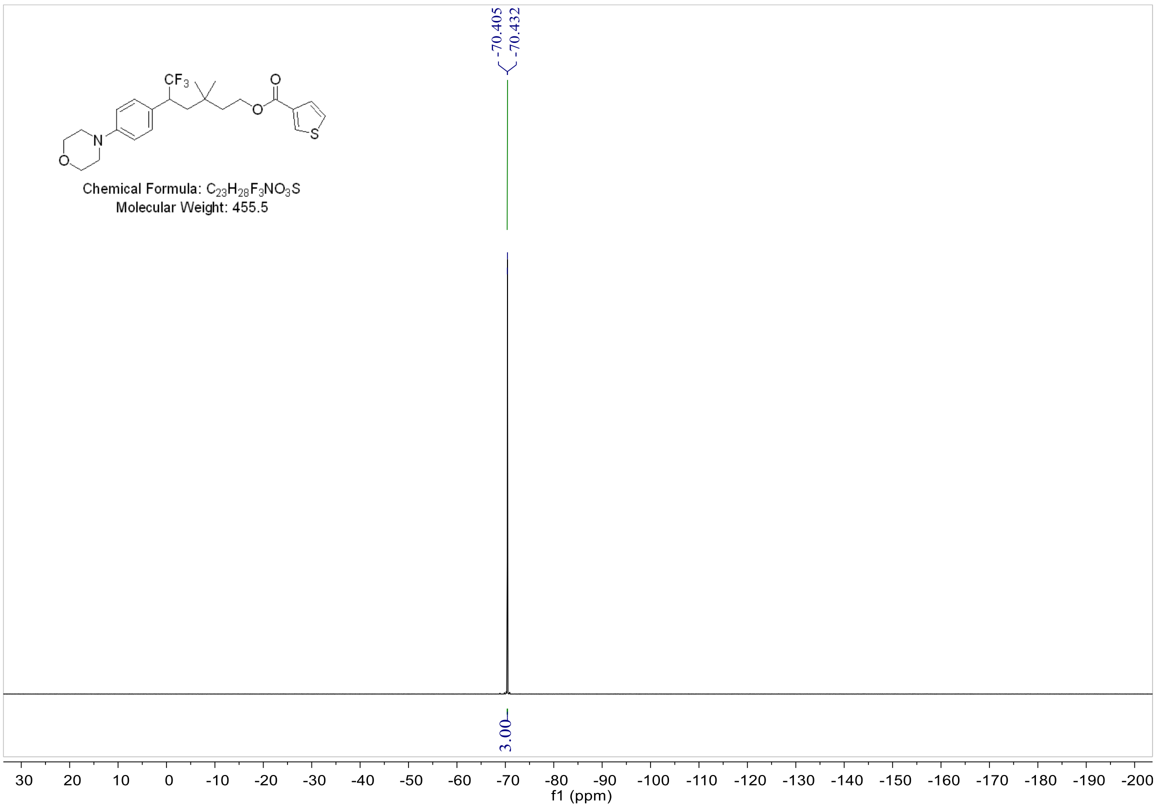

Supplementary Figure 91 ^{19}F NMR Spectrum of **5g**

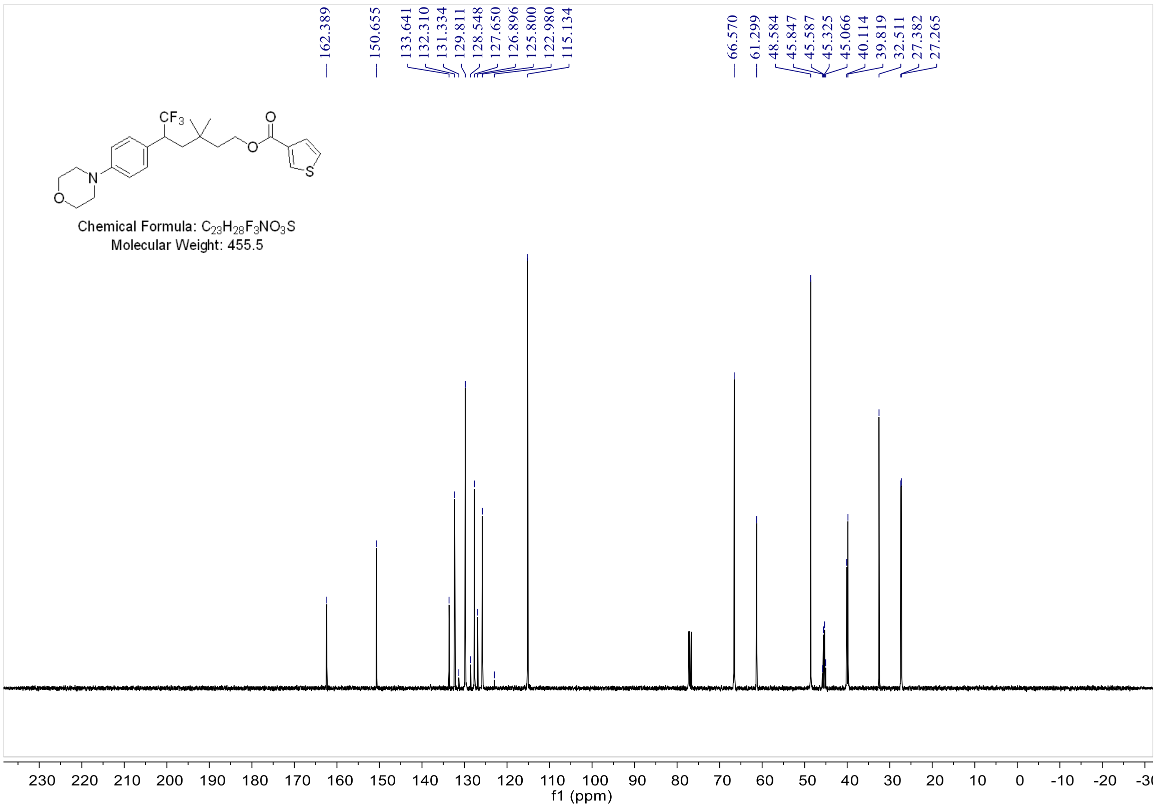

Supplementary Figure 92 ^{13}C NMR Spectrum of **5g**

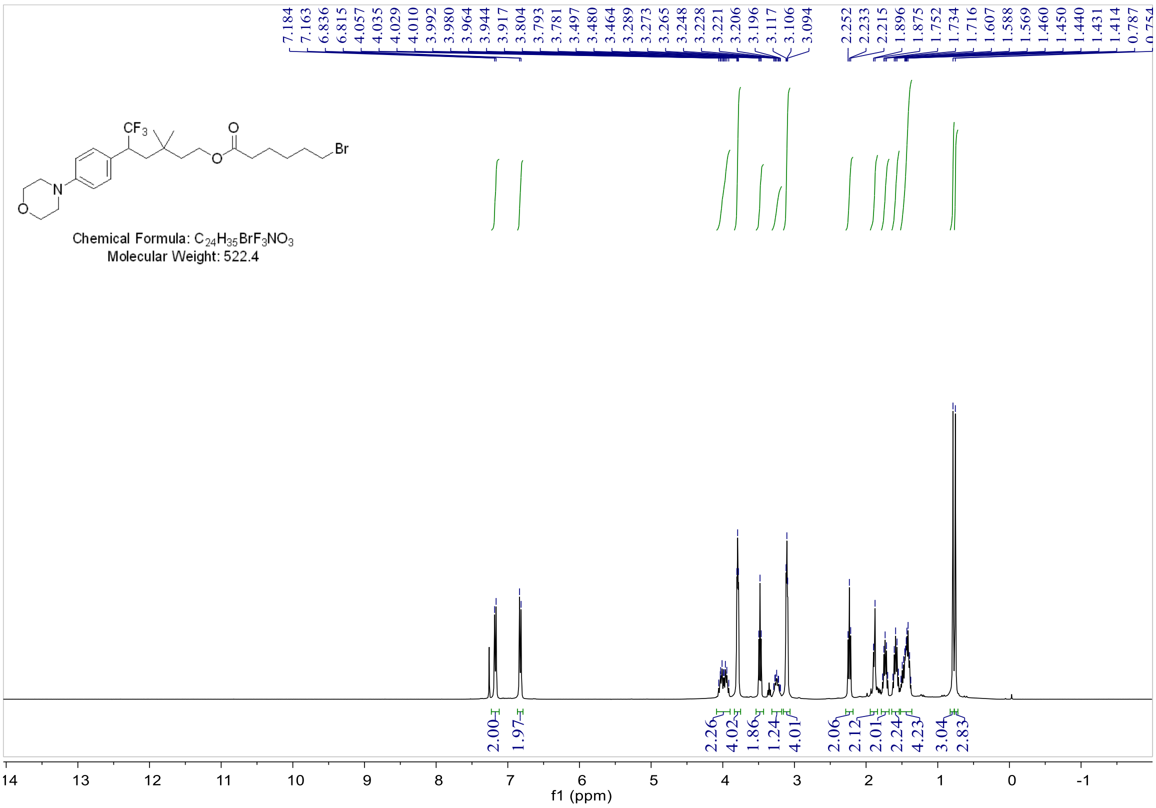

Supplementary Figure 93 ^{1}H NMR Spectrum of **5h**

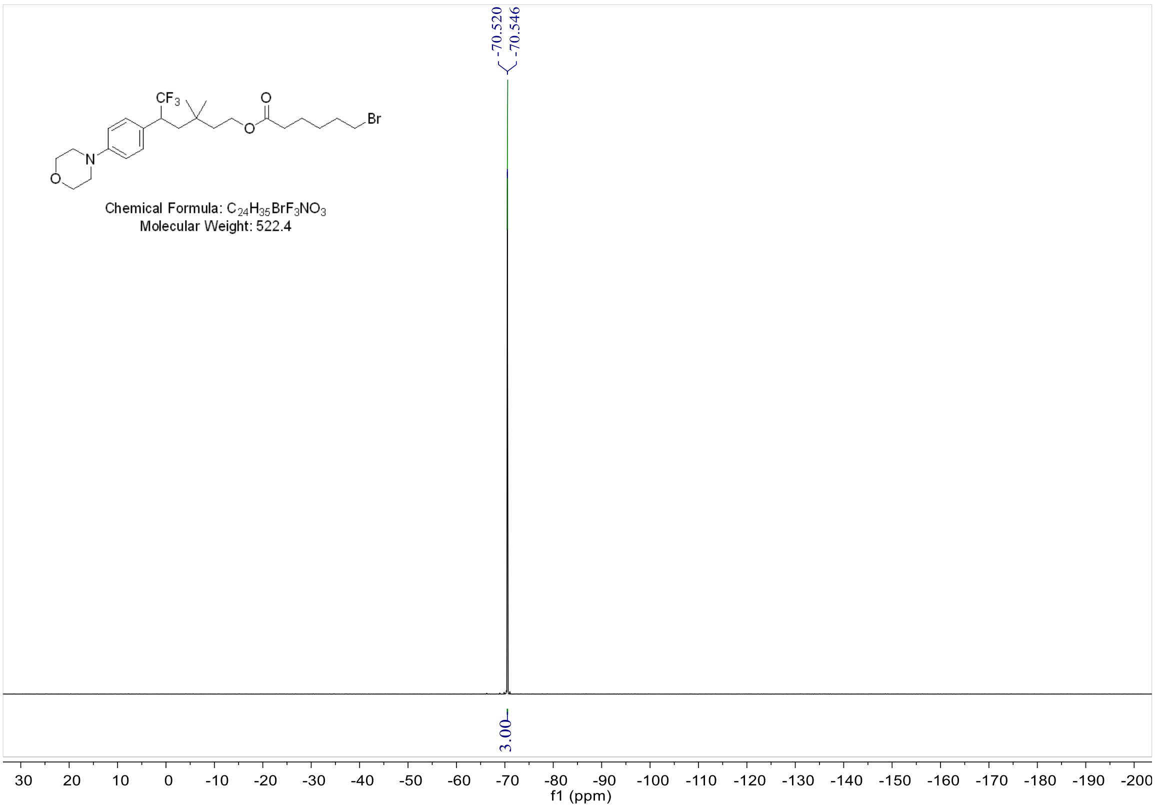

Supplementary Figure 94 ^{19}F NMR Spectrum of **5h**

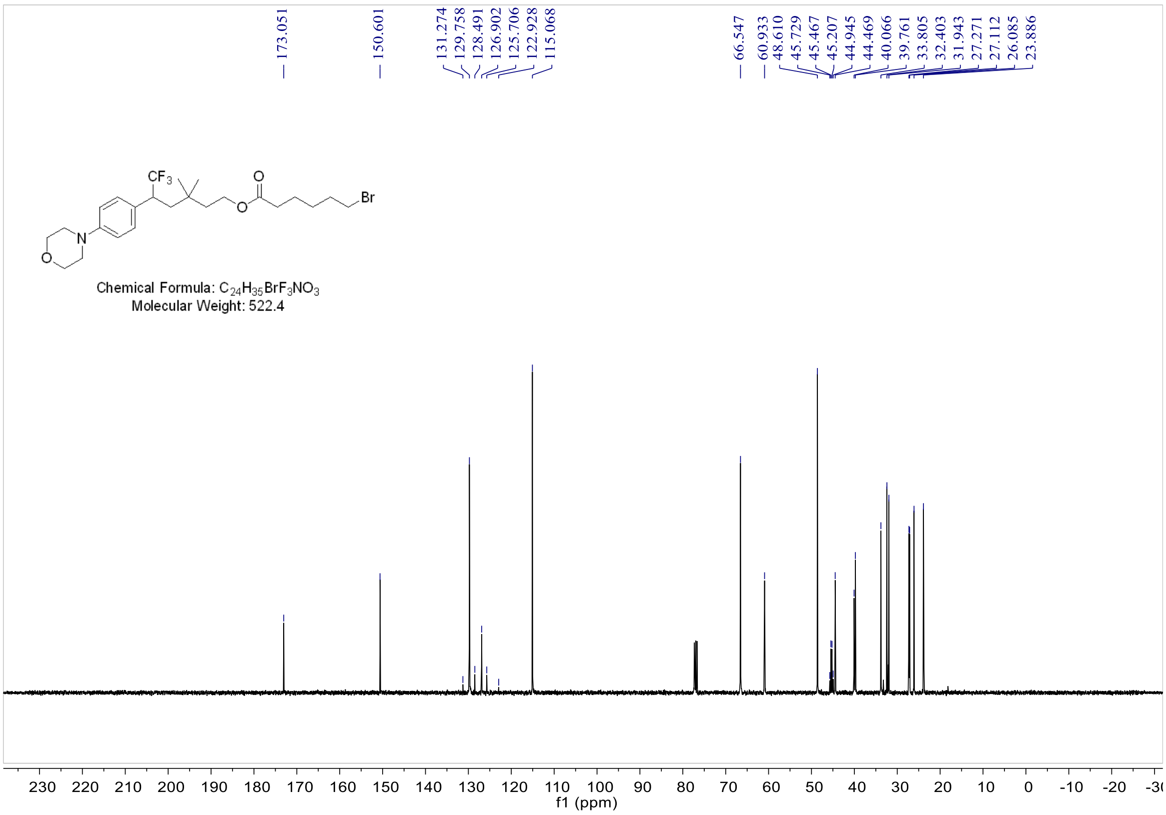

Supplementary Figure 95 ^{13}C NMR Spectrum of **5h**

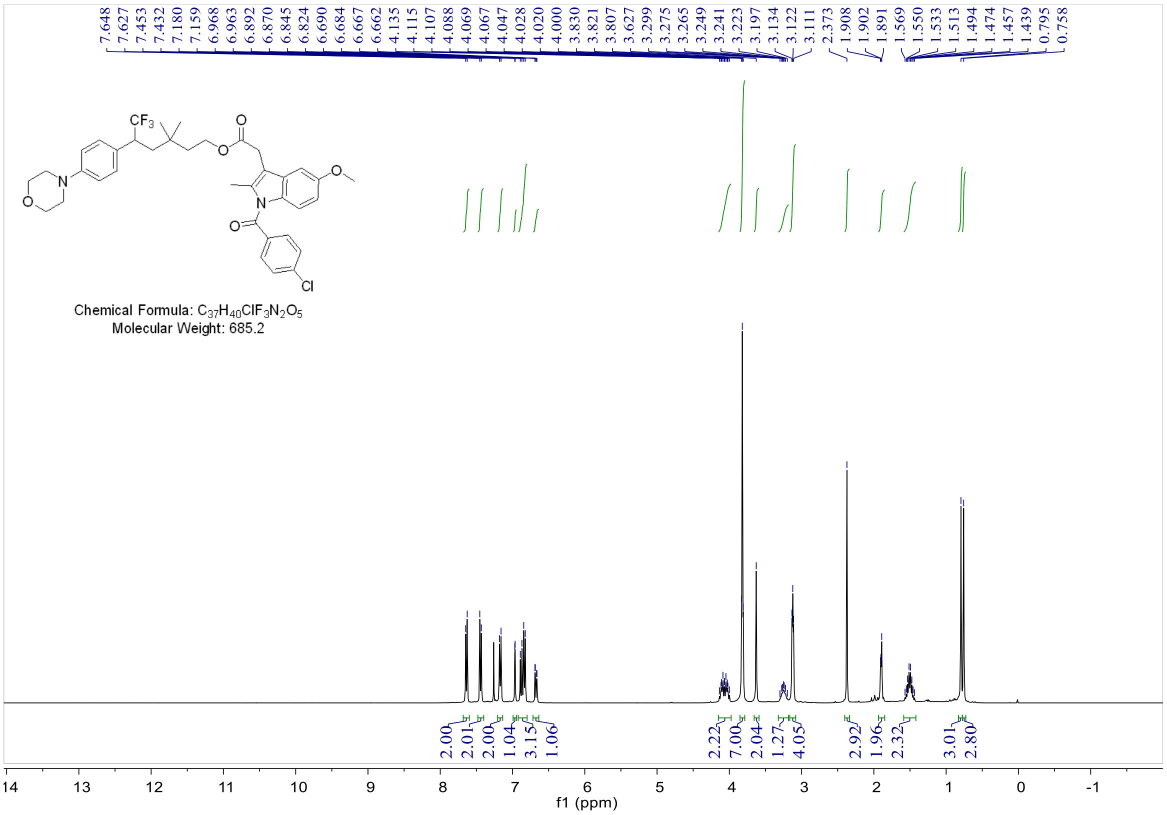

Supplementary Figure 96 ^{1}H NMR Spectrum of **5i**

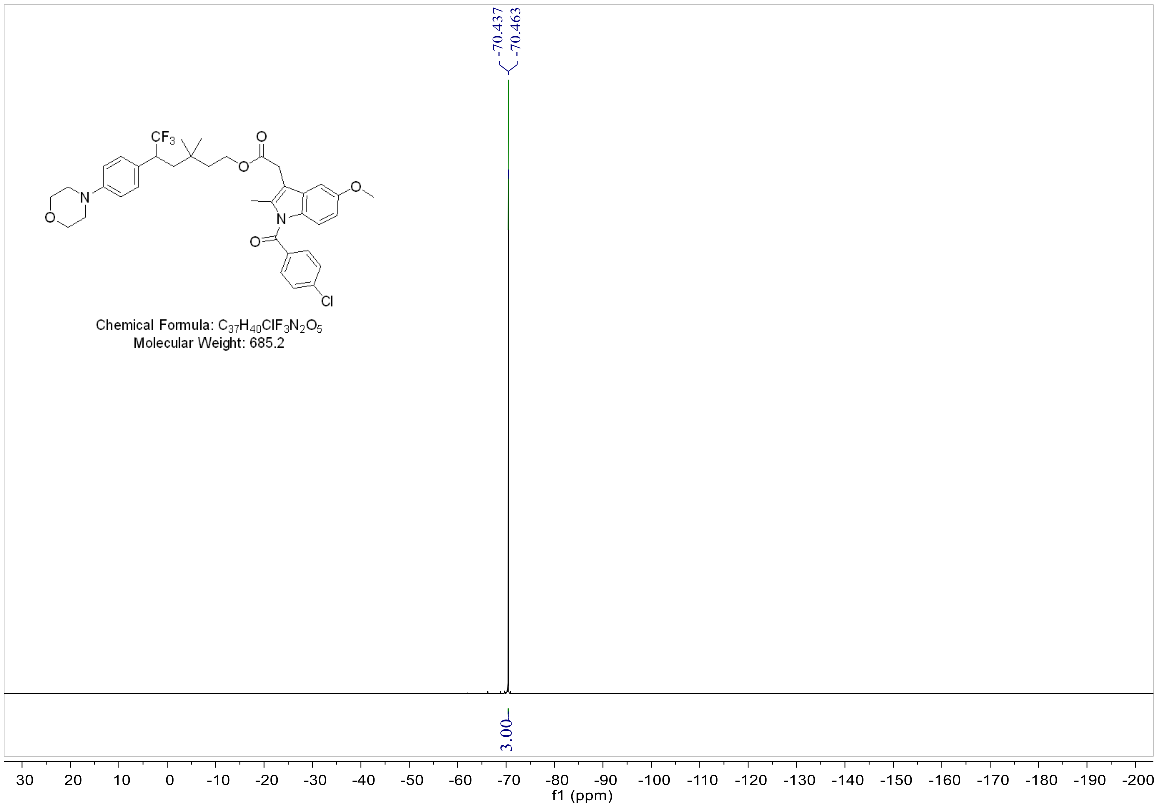

Supplementary Figure 97 ^{19}F NMR Spectrum of **5i**

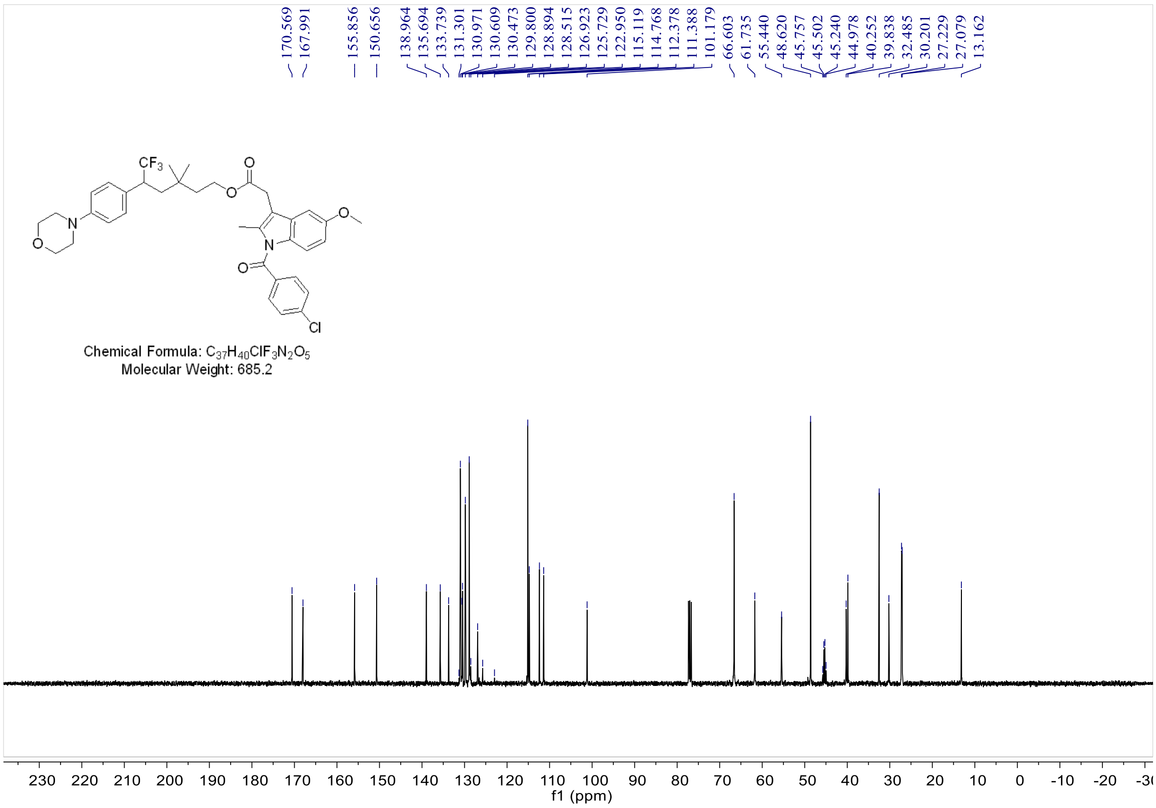

Supplementary Figure 98 ^{13}C NMR Spectrum of **5i**

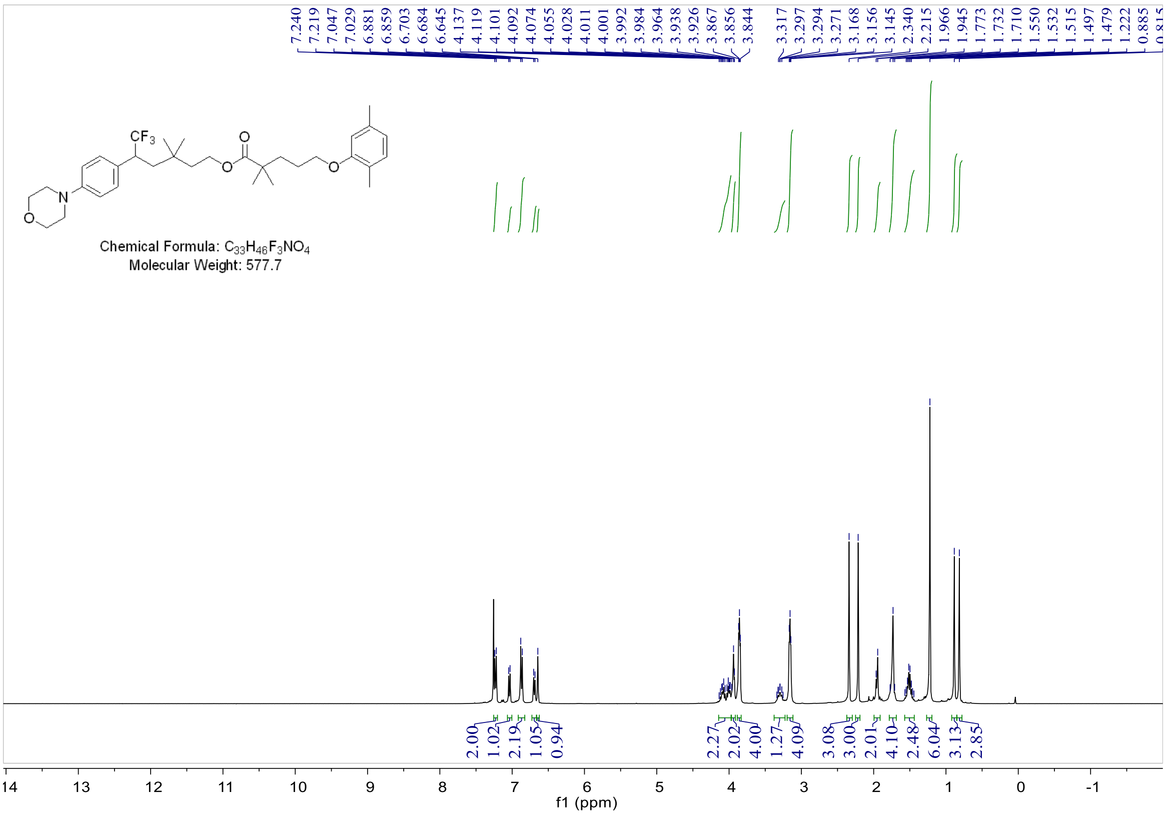

Supplementary Figure 99 ^{1}H NMR Spectrum of **5j**

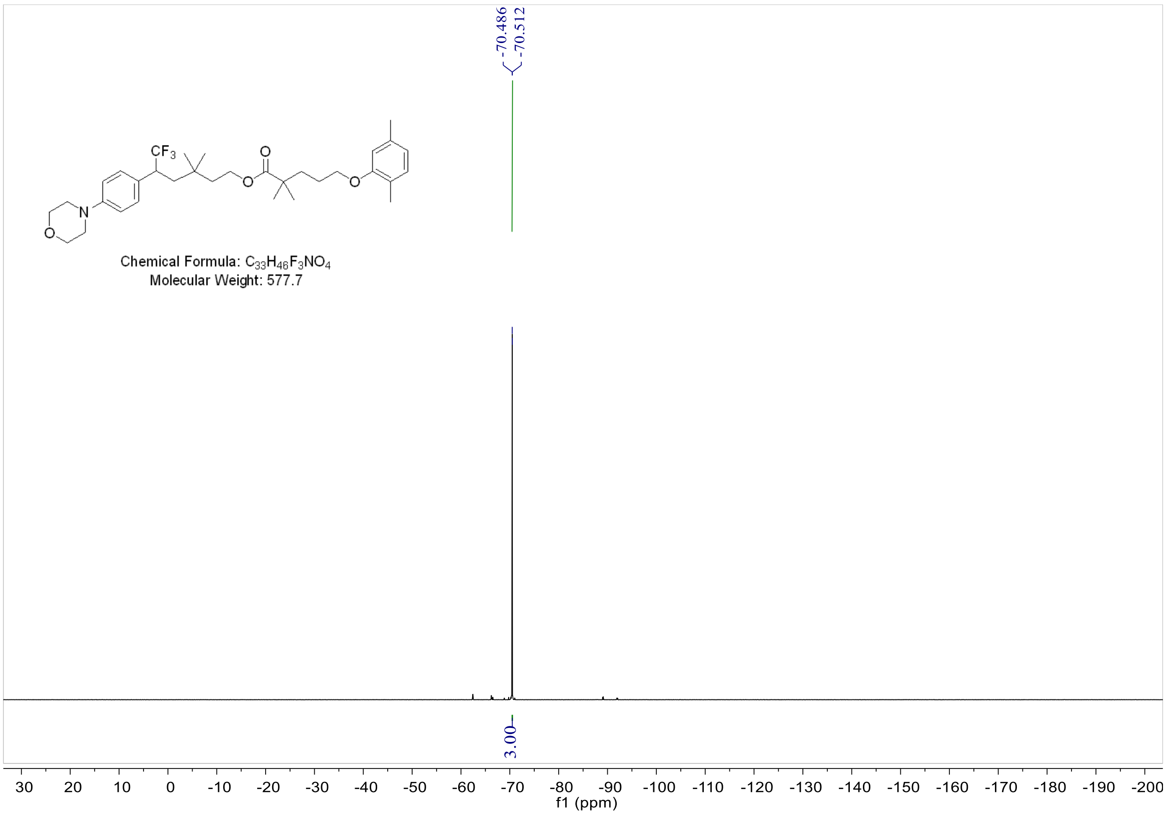

Supplementary Figure 100 ^{19}F NMR Spectrum of **5j**

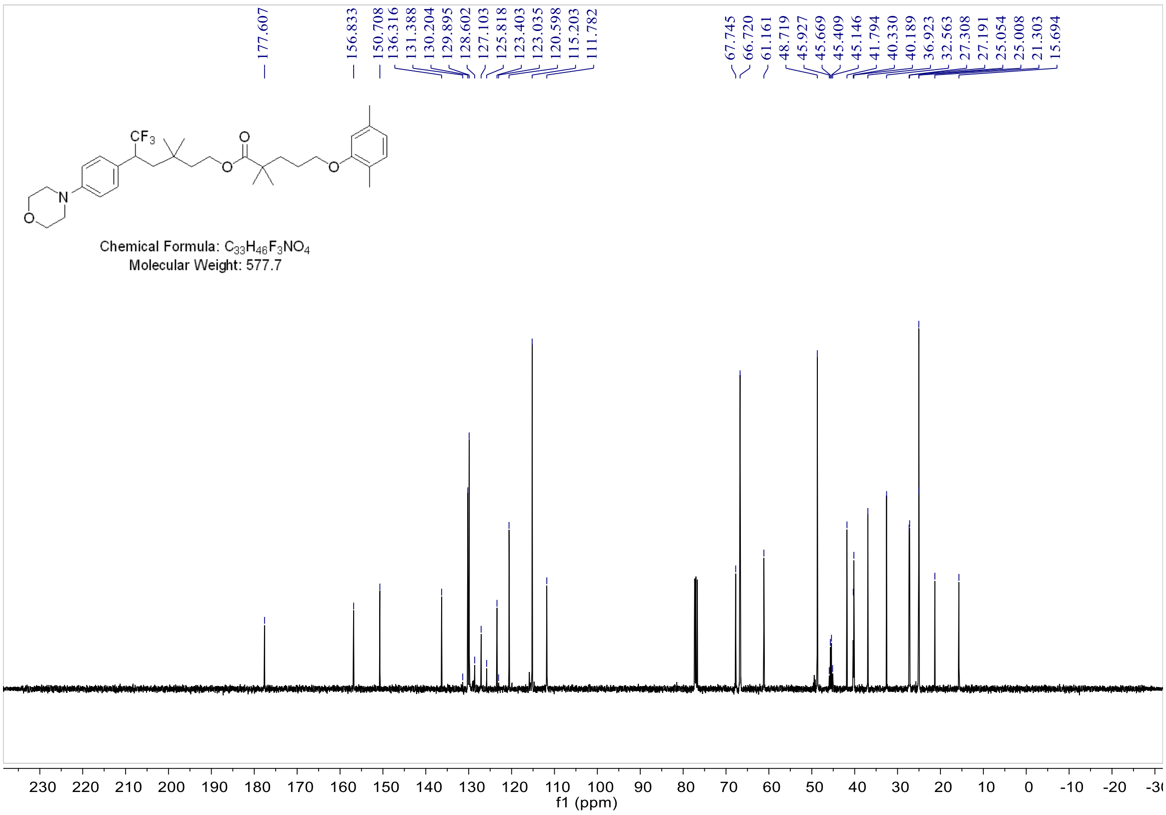

Supplementary Figure 101 ^{13}C NMR Spectrum of **5j**

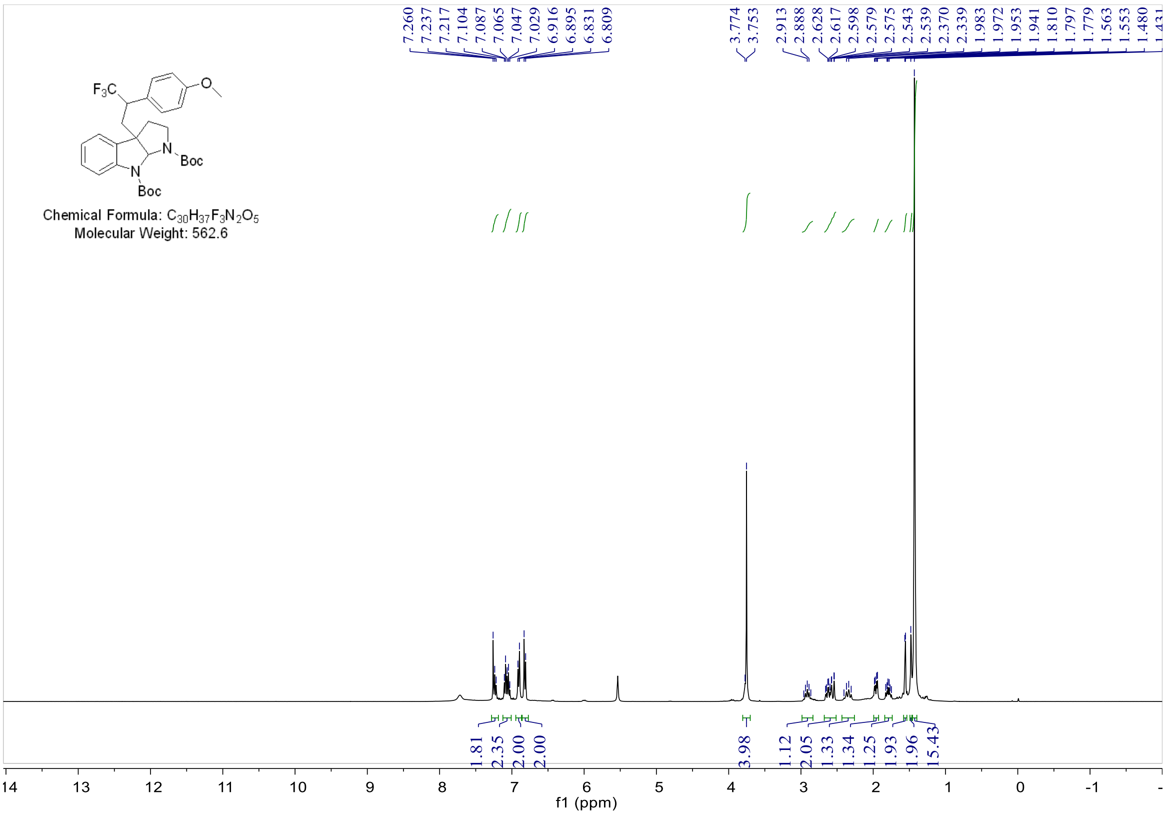

Supplementary Figure 102 ^{1}H NMR Spectrum of **5k**

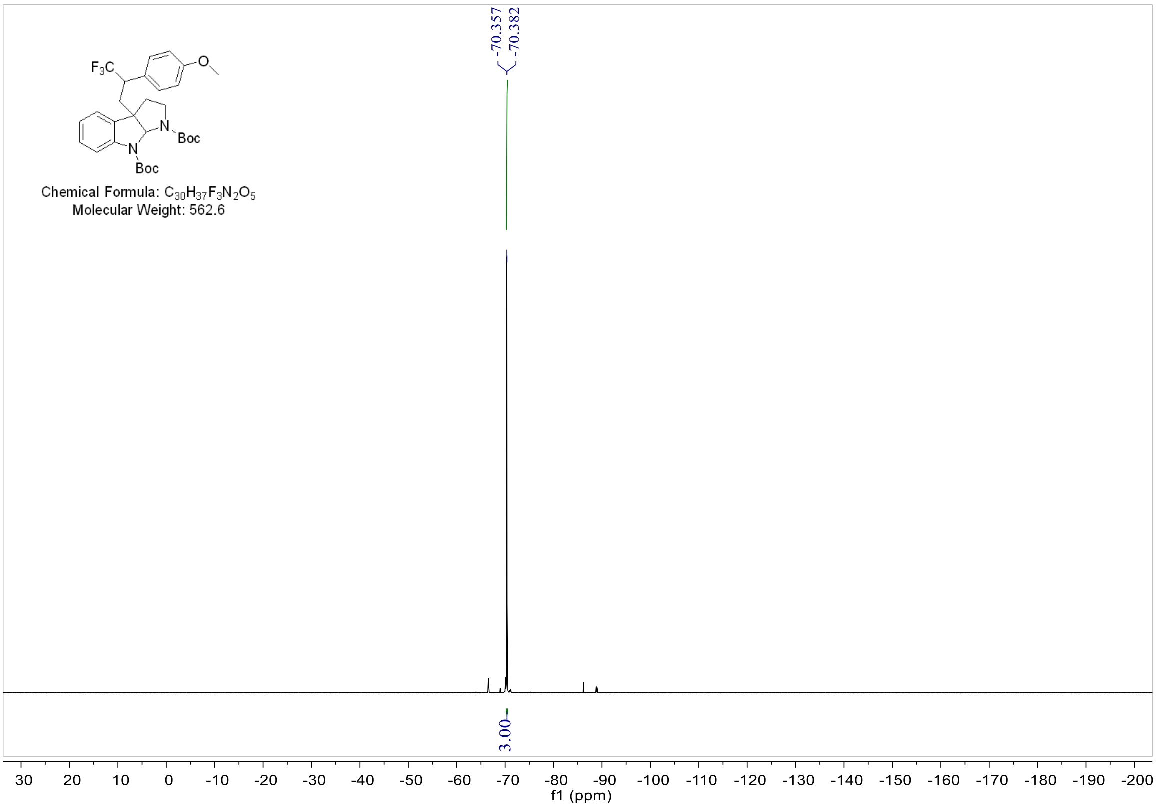

Supplementary Figure 103 ^{19}F NMR Spectrum of **5k**

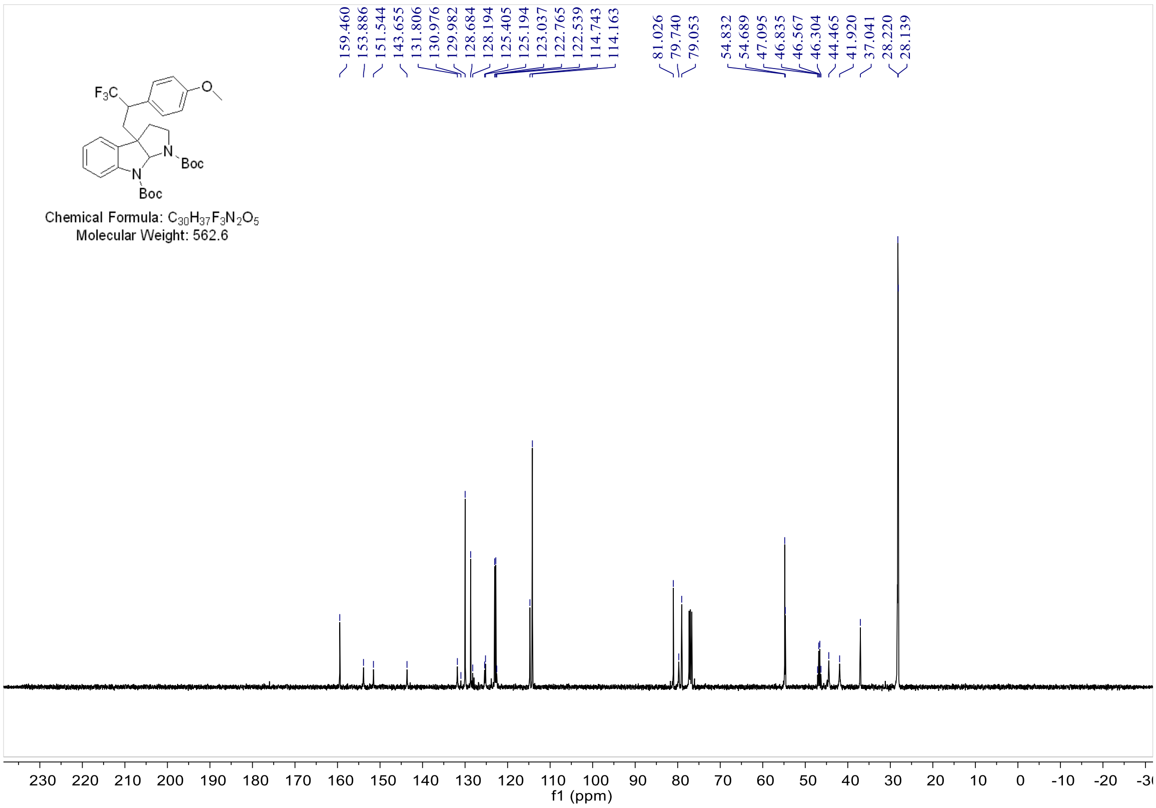

Supplementary Figure 104 ^{13}C NMR Spectrum of **5k**

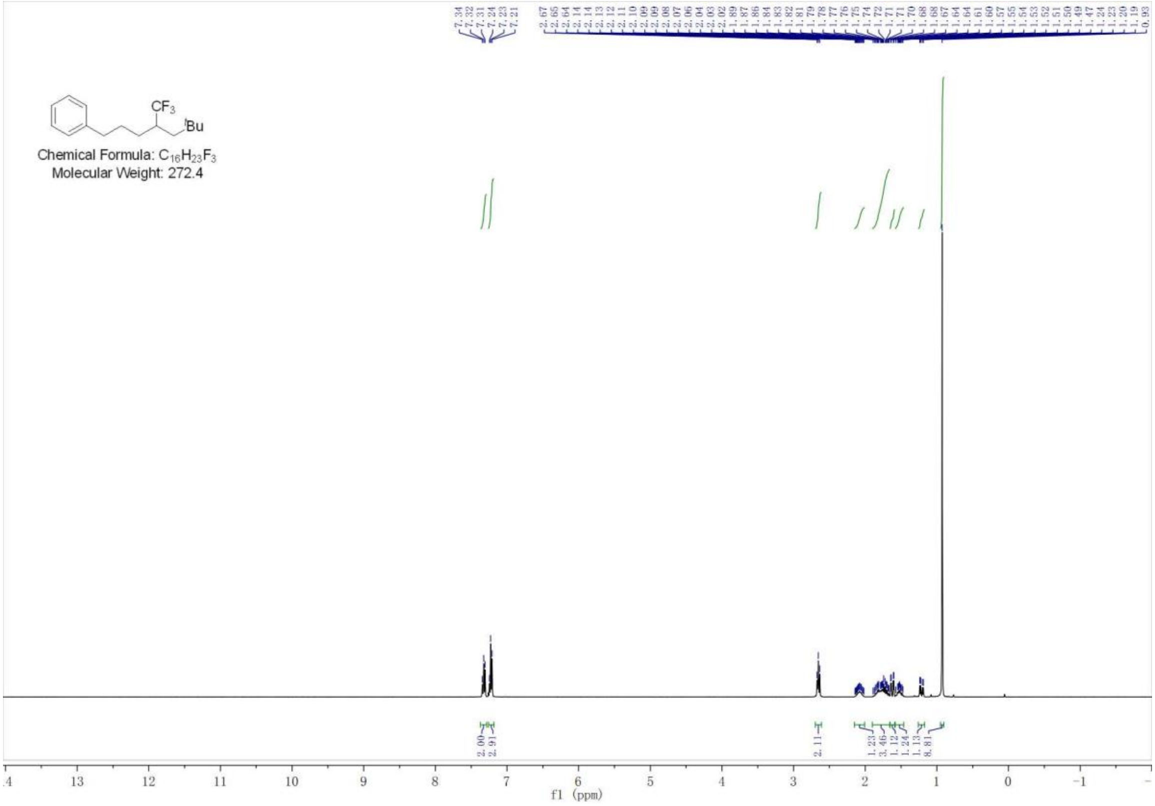

Supplementary Figure 105 ^{1}H NMR Spectrum of **5l**

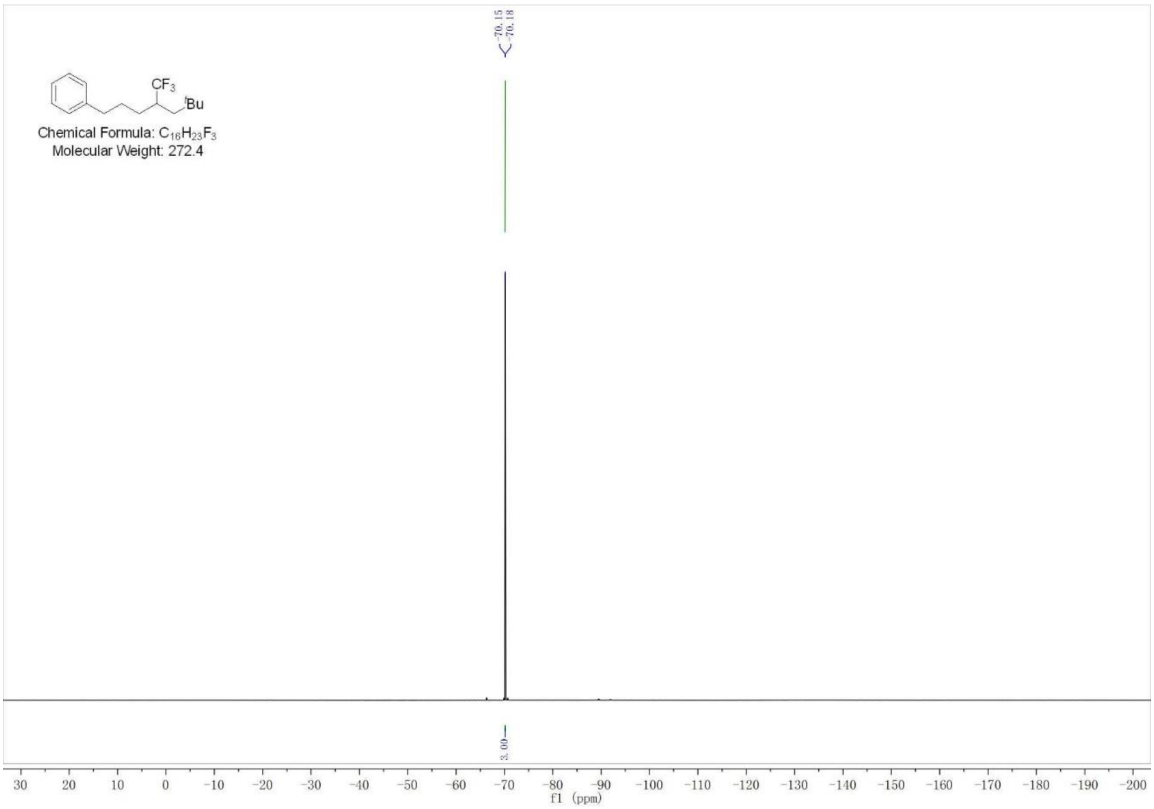

Supplementary Figure 106 ^{19}F NMR Spectrum of **5l**

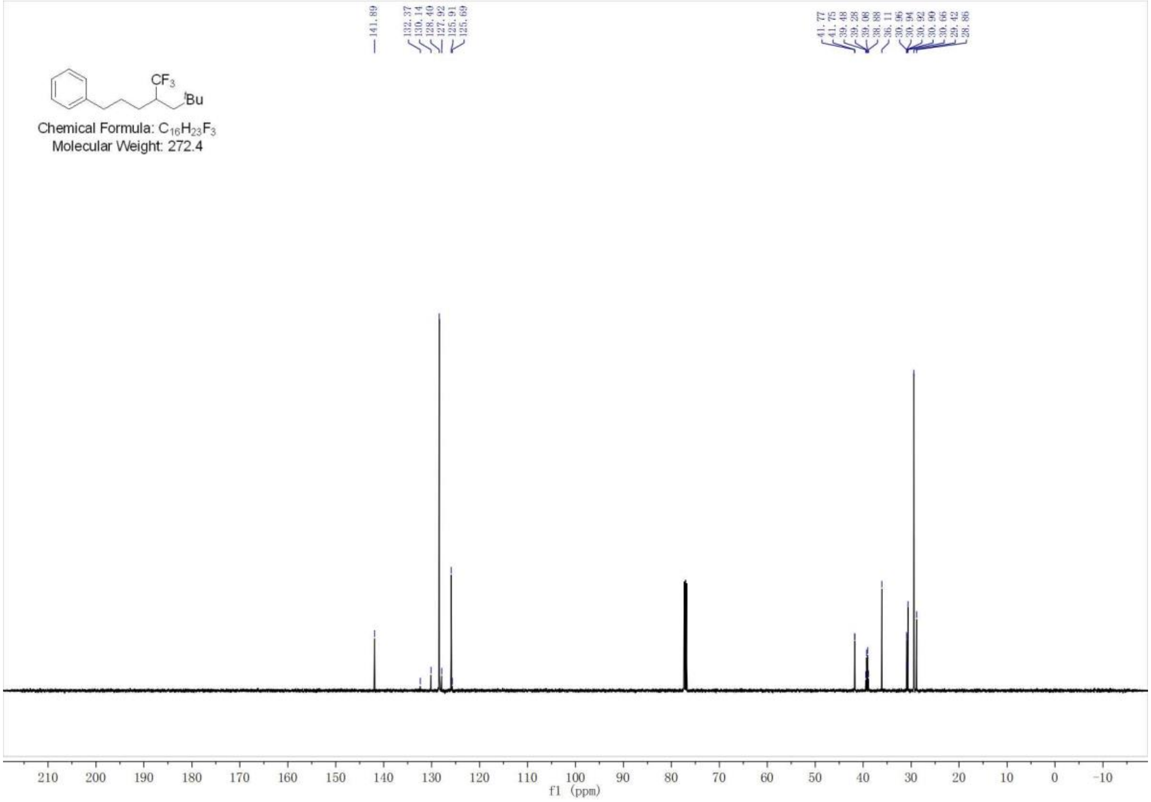

Supplementary Figure 107 ^{13}C NMR Spectrum of **5l**

Supplementary Note 3: Characterization Spectra for Compounds 8-10

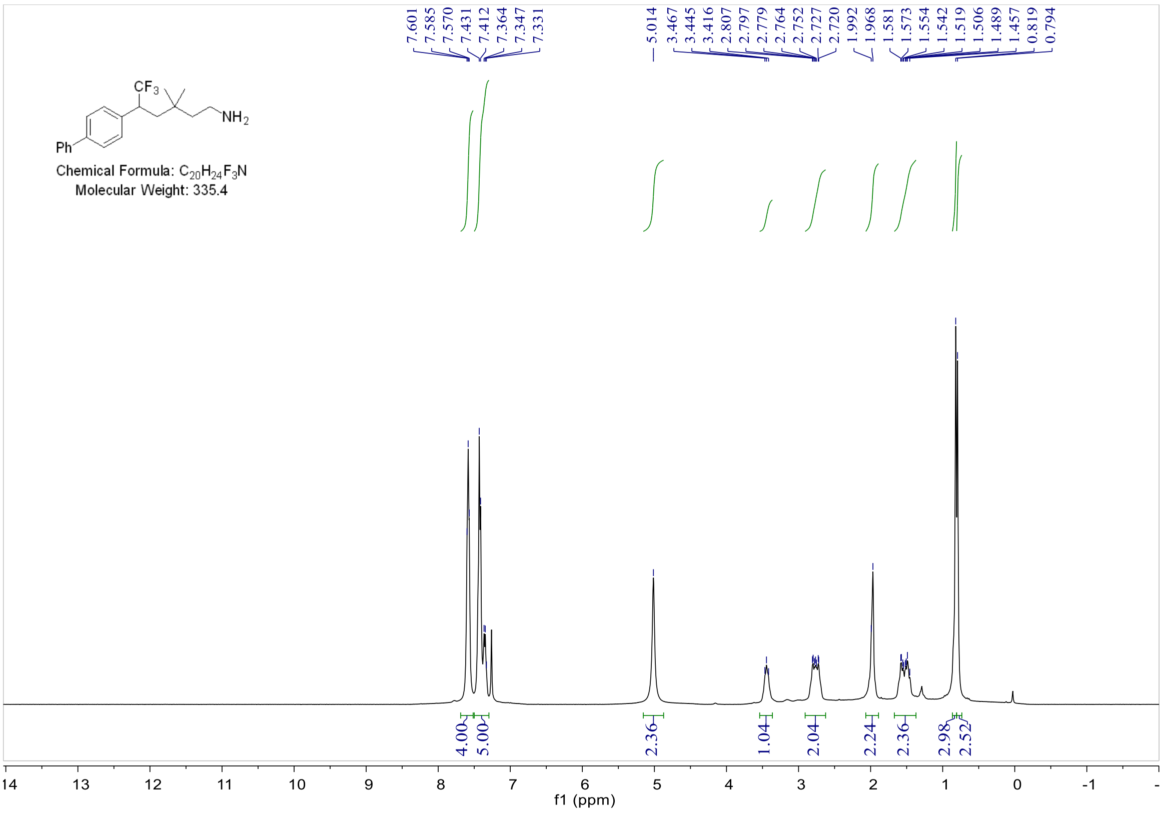

Supplementary Figure 108 ^{1}H NMR Spectrum of **8**

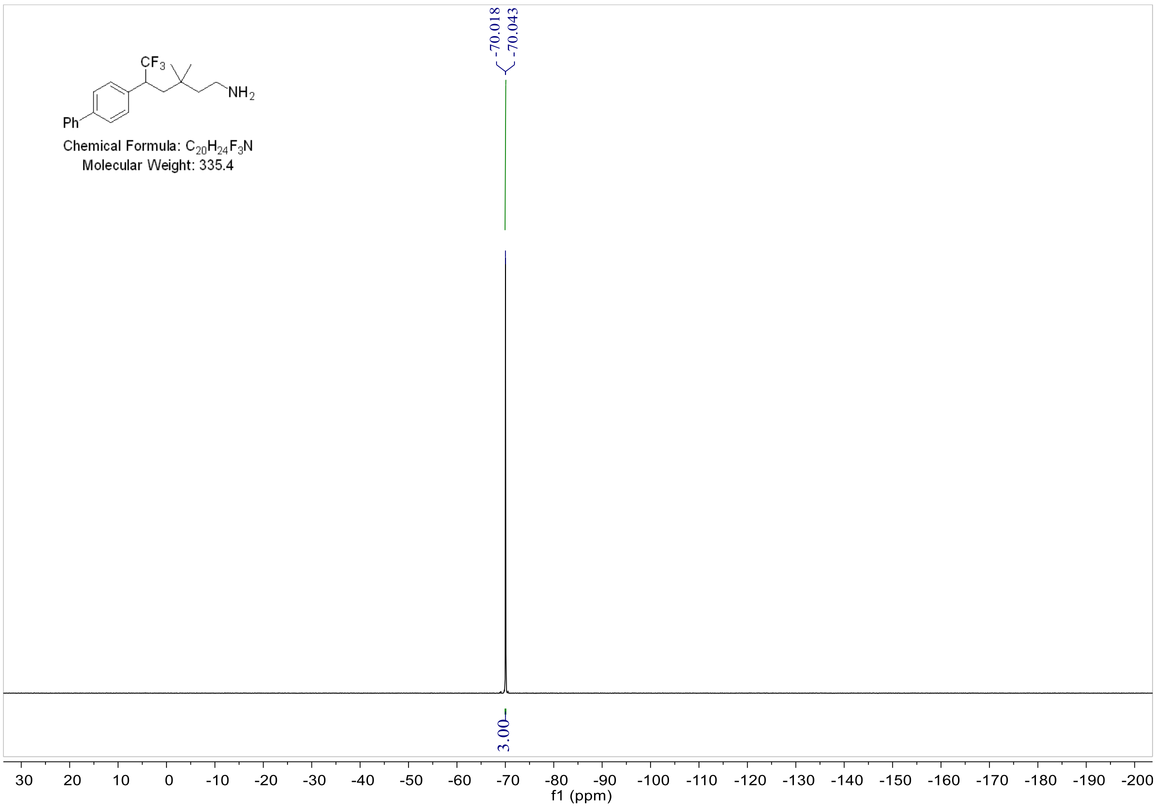

Supplementary Figure 109 ^{19}F NMR Spectrum of **8**

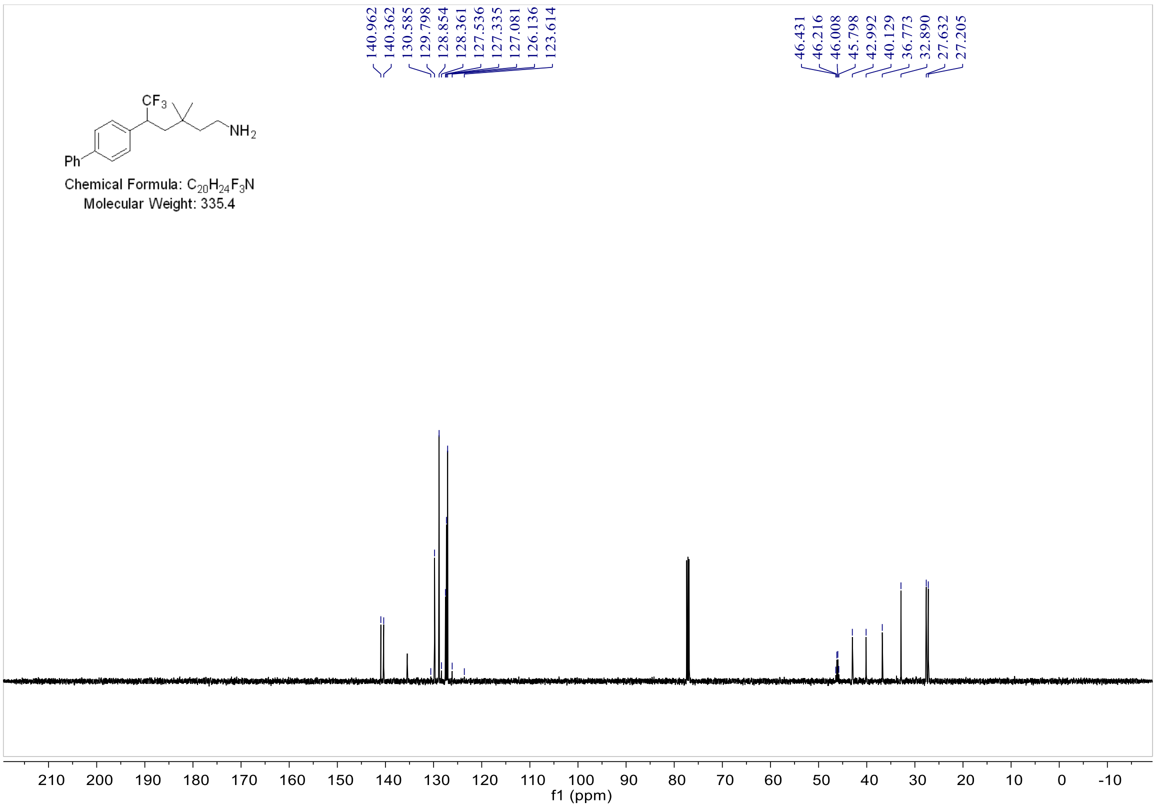

Supplementary Figure 110 ^{13}C NMR Spectrum of **8**

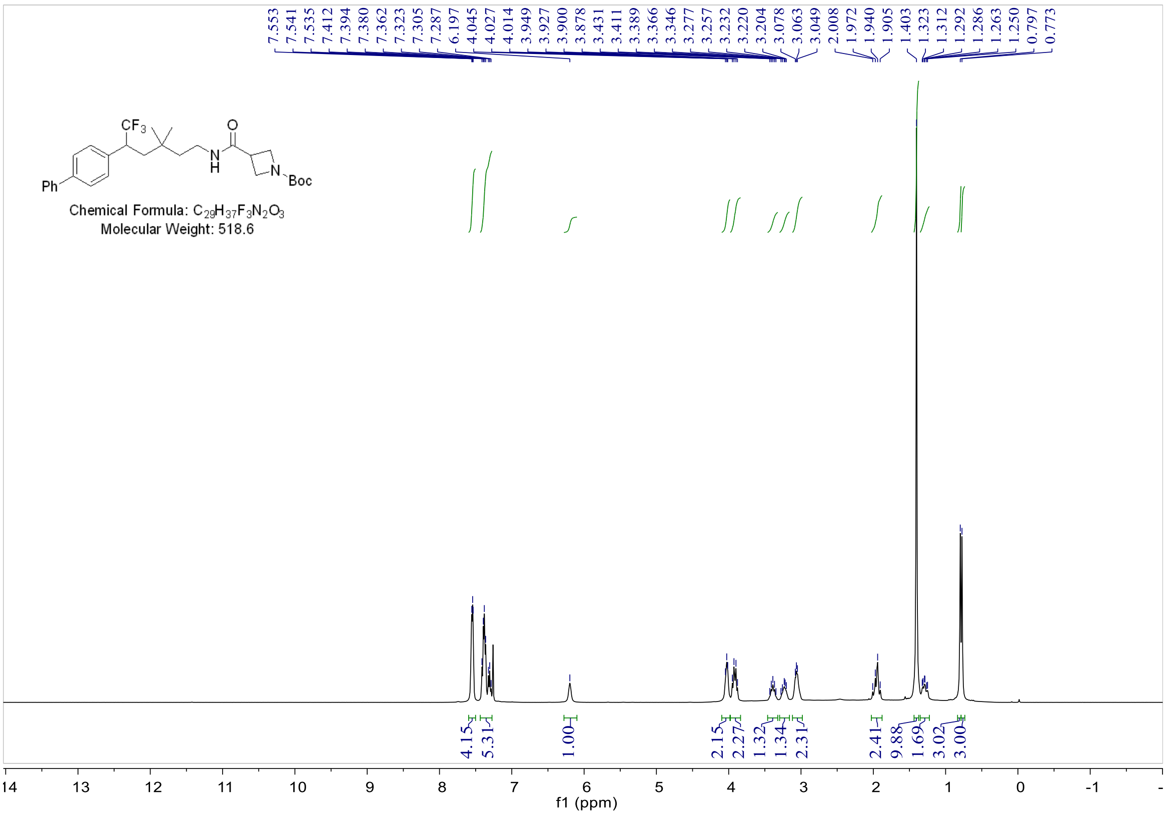

Supplementary Figure 111 ^{1}H NMR Spectrum of **10a**

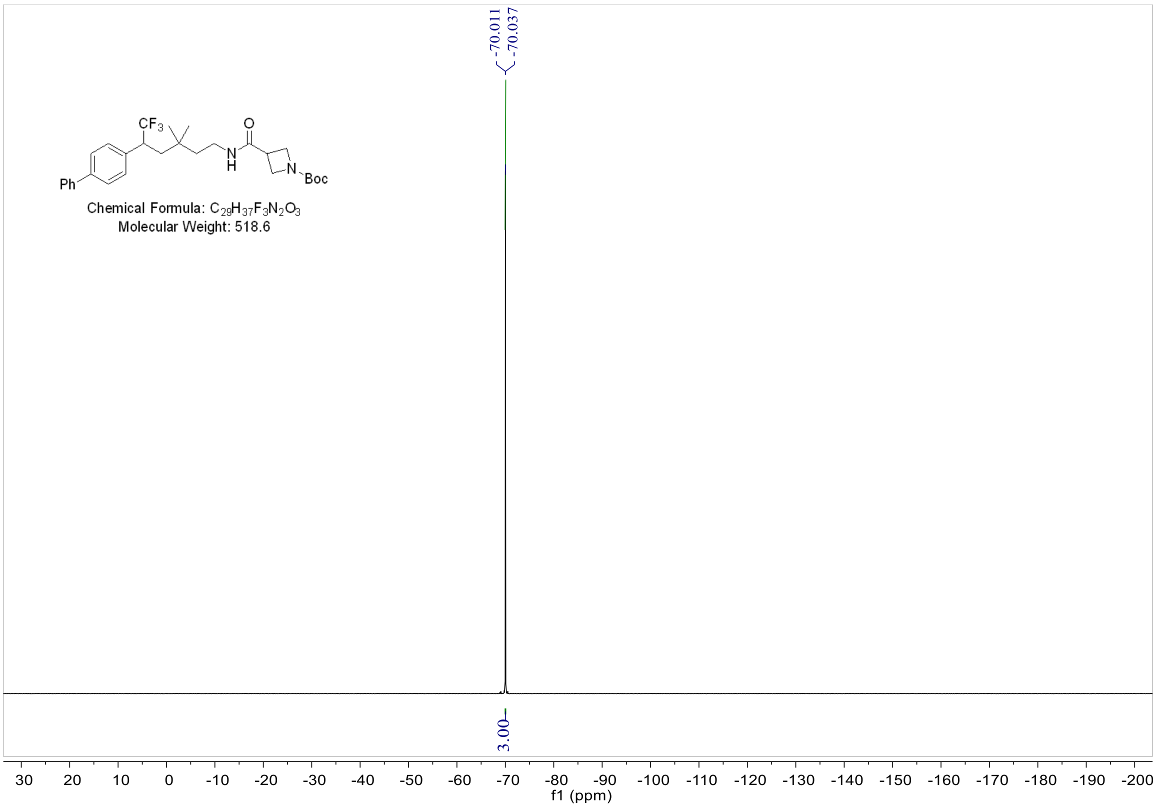

Supplementary Figure 112 ^{19}F NMR Spectrum of **10a**

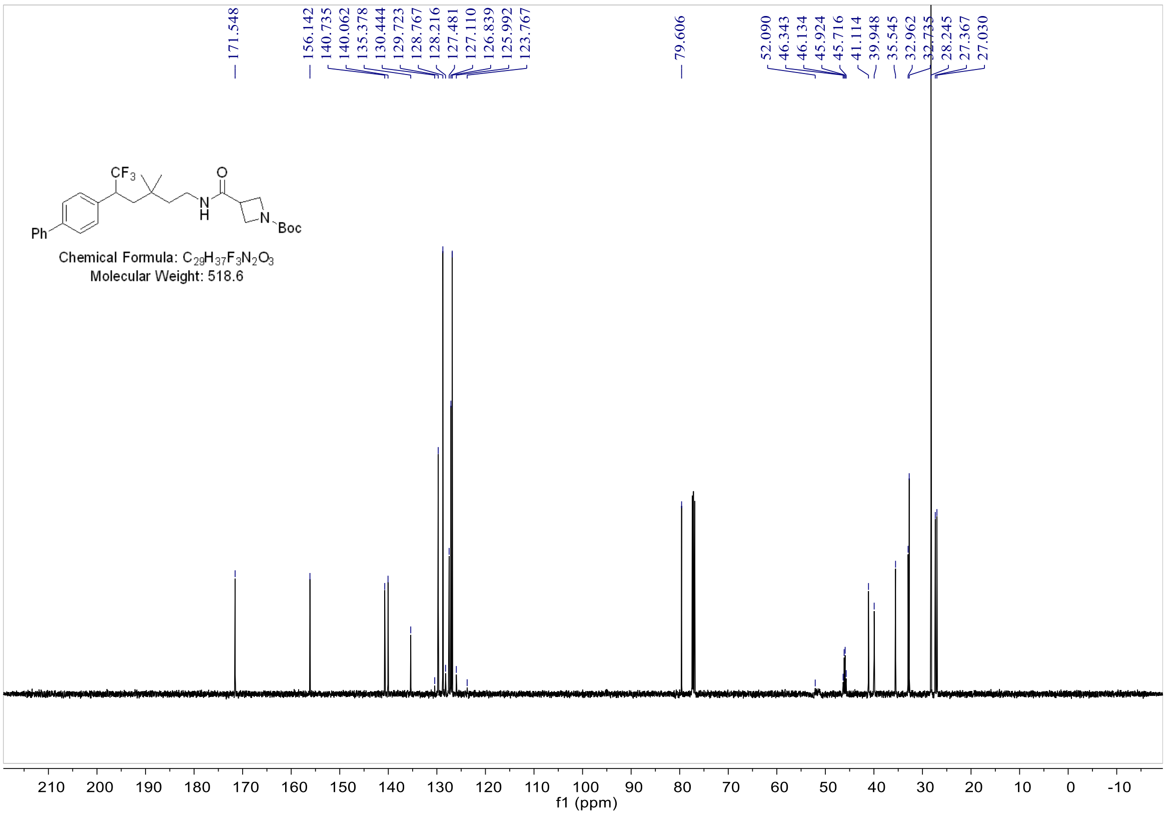

Supplementary Figure 113 ^{13}C NMR Spectrum of **10a**

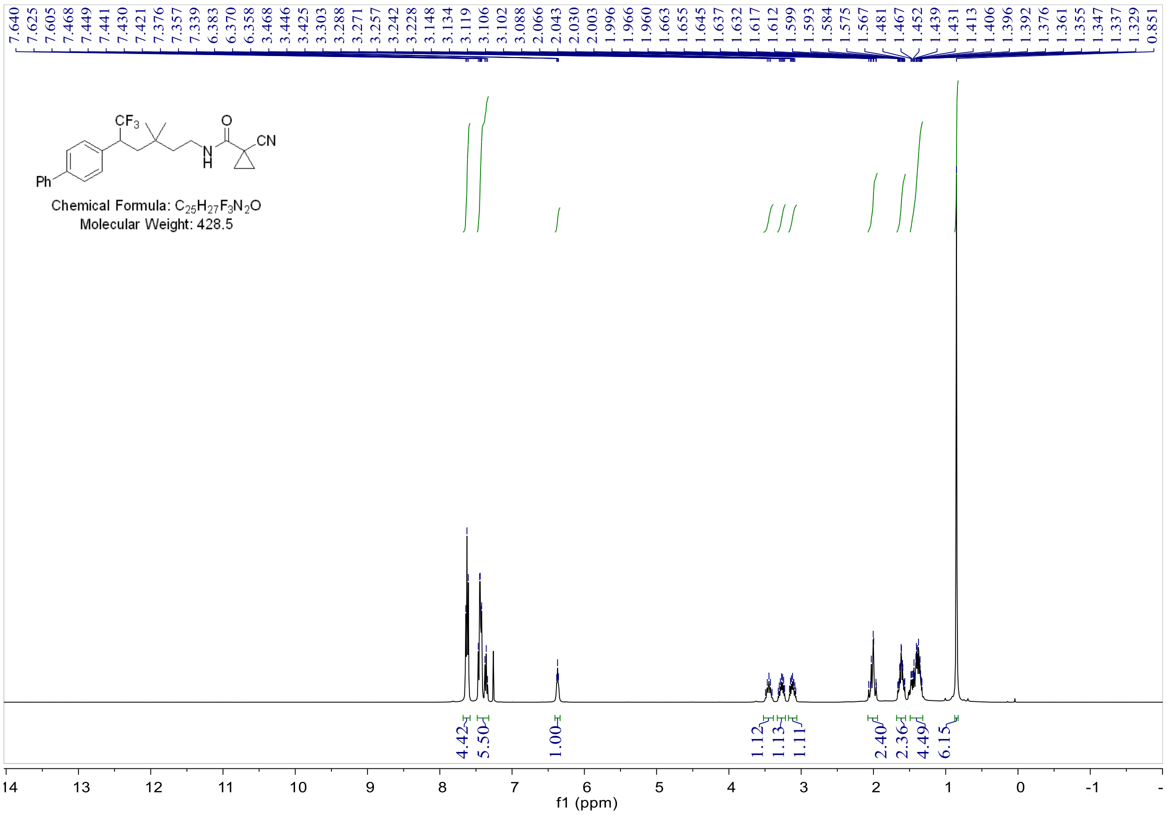

Supplementary Figure 114 ^{1}H NMR Spectrum of **10b**

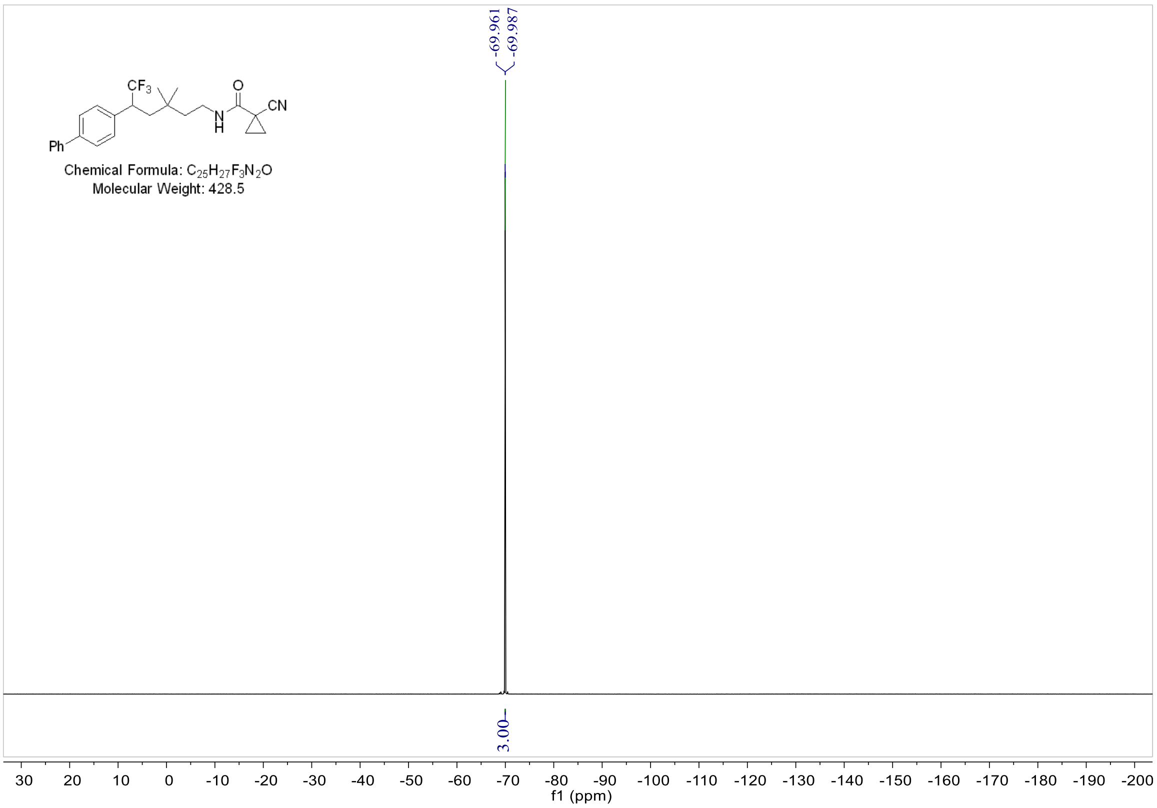

Supplementary Figure 115 ^{19}F NMR Spectrum of **10b**

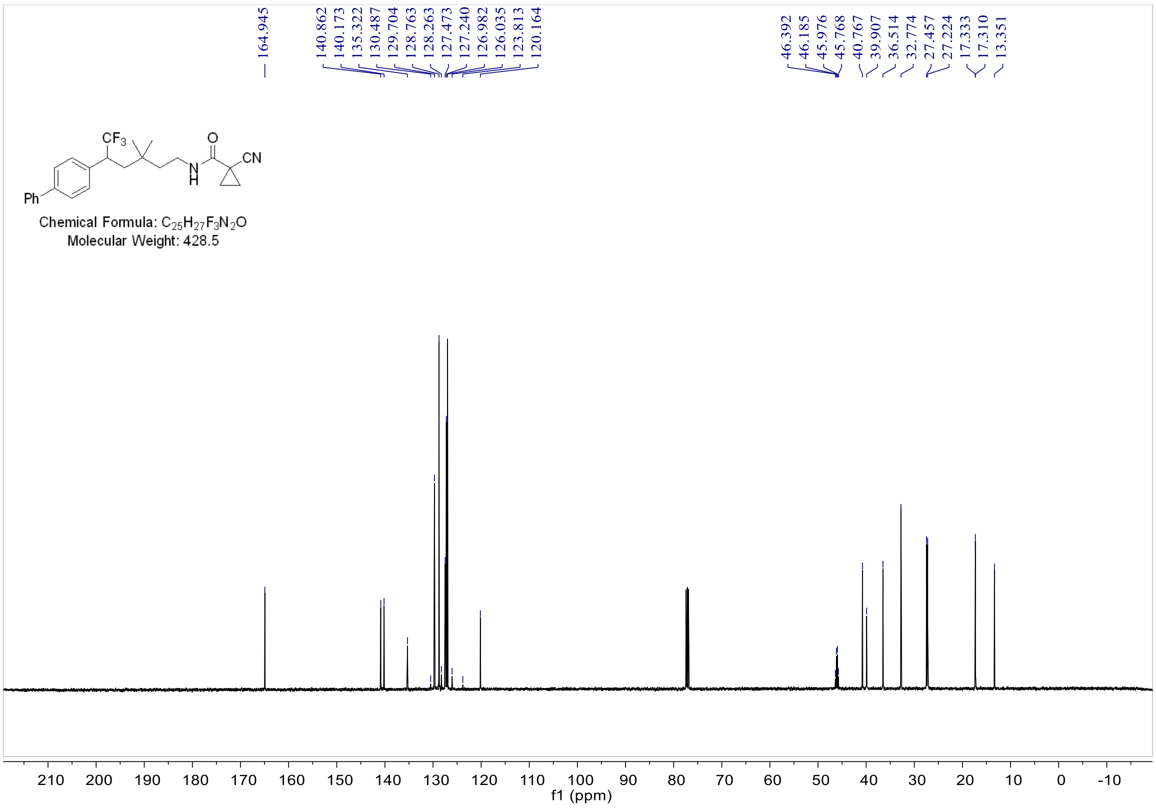

Supplementary Figure 116 ^{13}C NMR Spectrum of **10b**

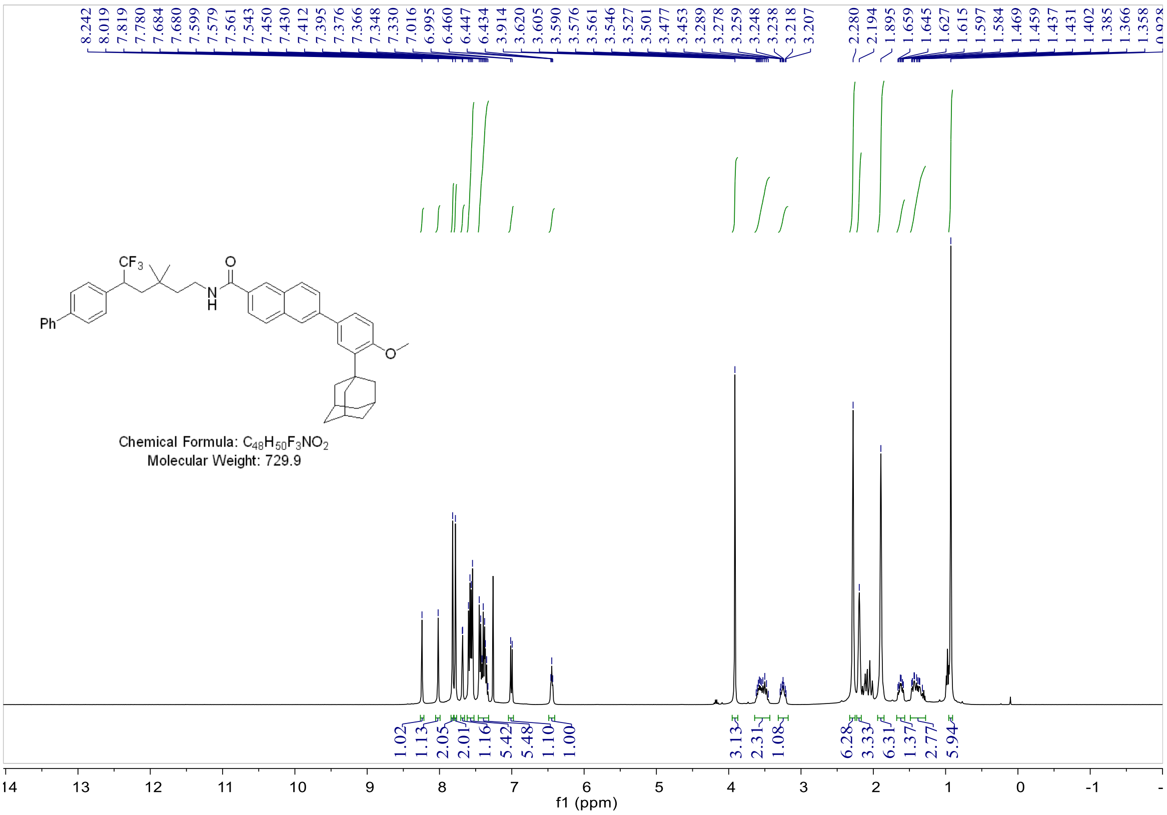

Supplementary Figure 117 ^{1}H NMR Spectrum of **10c**

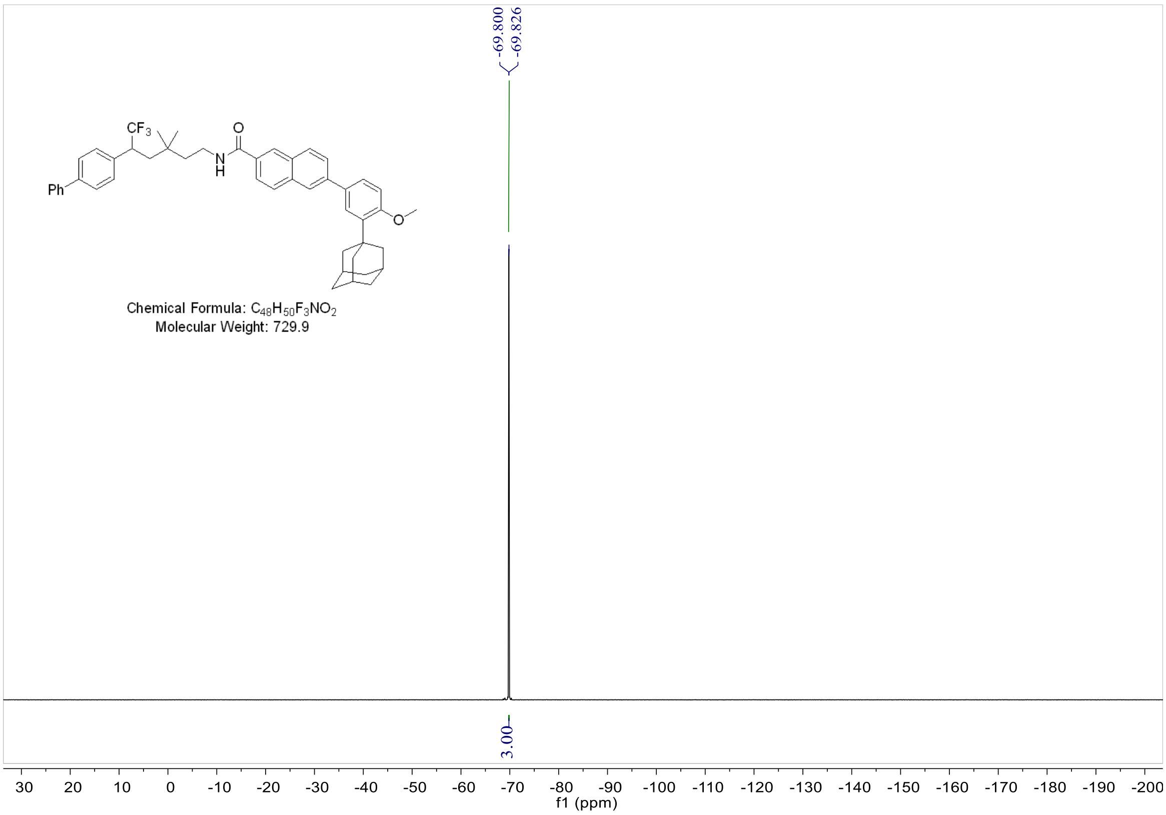

Supplementary Figure 118 ^{19}F NMR Spectrum of **10c**

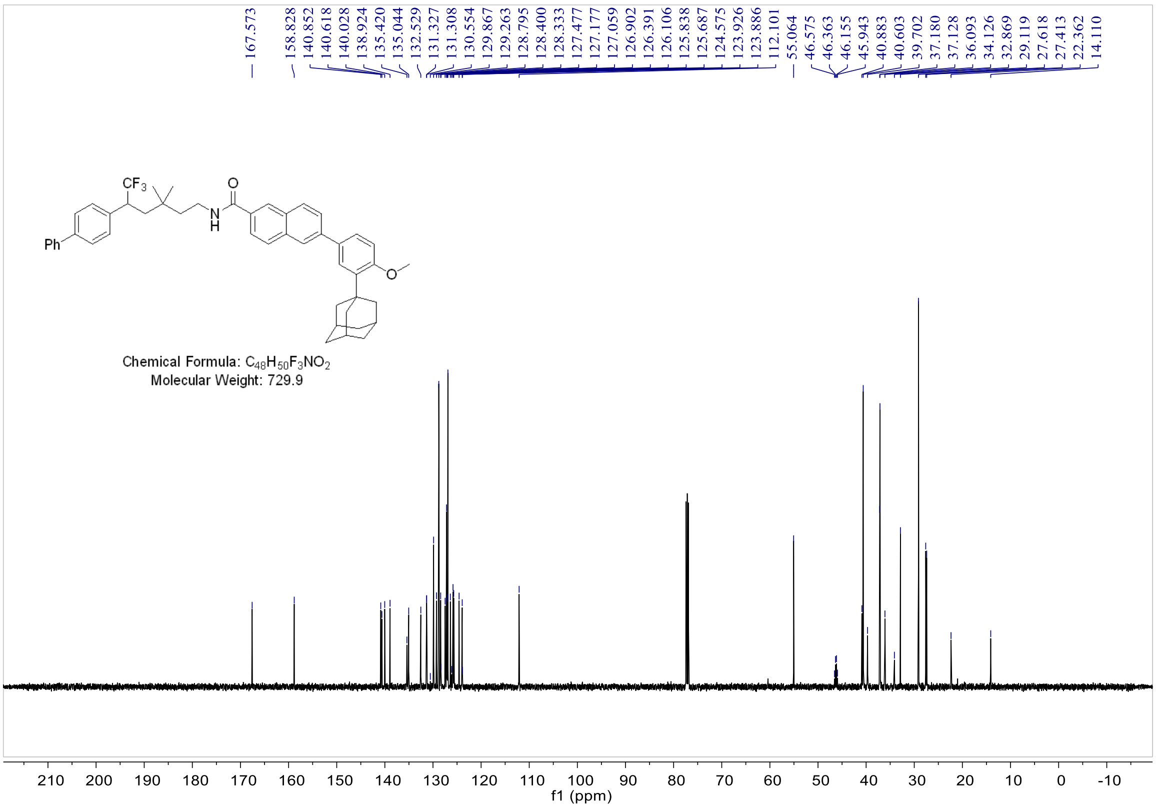

Supplementary Figure 119 ^{13}C NMR Spectrum of **10c**

Supplementary References

1. Kurandina, D., Rivas, M., Radzhabov, M. & Gevorgyan, V. Heck Reaction of Electronically Diverse Tertiary Alkyl Halides. *Org. Lett.* **20,** 357–360 (2018).
2. Shah, R., Farmer, L. A., Zilka, O., van Kessel, A. T. M. & Pratt, D. A. Beyond DPPH: Use of Fluorescence-Enabled Inhibited Autoxidation to Predict Oxidative Cell Death Rescue. *Cell Chem. Biol.* **26,** 1594-1607.e7 (2019).
3. Kiyokawa, K., Takemoto, K. & Minakata, S. Ritter-type amination of C-H bonds at tertiary carbon centers using iodic acid as an oxidant. *Chem. Commun.* **52,** 13082–13085 (2016).
4. Espejo, V. R. & Rainier, J. D. An expeditious synthesis of C(3)-N(1') heterodimeric indolines. *J. Am. Chem. Soc.* **130,** 12894–12895 (2008).
5. Dudnik, A. S. & Fu, G. C. Nickel-catalyzed coupling reactions of alkyl electrophiles, including unactivated tertiary halides, to generate carbon-boron bonds. *J. Am. Chem. Soc.* **134,** 10693–10697 (2012).
6. Piller, F. M., Appukkuttan, P., Gavryushin, A., Helm, M. & Knochel, P. Convenient preparation of polyfunctional aryl magnesium reagents by a direct magnesium insertion in the presence of LiCl. *Angew. Chem. Int. Ed.* **47,** 6802–6806 (2008).
7. Piller, F. M. *et al.* Preparation of polyfunctional arylmagnesium, arylzinc, and benzylic zinc reagents by using magnesium in the presence of LiCl. *Chem. Eur. J* **15,** 7192–7202 (2009).
8. Cornella, J. *et al.* Practical Ni-Catalyzed Aryl-Alkyl Cross-Coupling of Secondary Redox-Active Esters. *J. Am. Chem. Soc.* **138,** 2174–2177 (2016).
9. Cornella, J. *et al.* Practical Ni-Catalyzed Aryl-Alkyl Cross-Coupling of Secondary Redox-Active Esters. *J. Am. Chem. Soc.* **138,** 2174–2177 (2016).
10. Krasovskiy, A. & Knochel, P. Convenient Titration Method for Organometallic Zinc, Magnesium, and Lanthanide Reagents. *Synthesis* **2006,** 890–891 (2006).
11. Manolikakes, G. & Knochel, P. Radical catalysis of Kumada cross-coupling reactions using functionalized Grignard reagents. *Angew. Chem. Int. Ed.* **48,** 205–209 (2009).
12. Xu, C. et al. Difluoromethylation of (hetero)aryl chlorides with chlorodifluoromethane catalyzed by nickel. *Nat. Commun.* **9**, 1170 (2018).
